# LRBA regulates actin cytoskeleton dynamics through NMIIA during B cell immune responses

**DOI:** 10.1038/s44319-026-00831-3

**Published:** 2026-06-12

**Authors:** Elena Sindram, Juan Eduardo Montero-Hernández, Quentin Frenger, Hannah van Dijk, Anna Lang, Vanessa Zeidler, Rebecca Marsiske, Manuel Rhiel, Kerstin Geiger, Tatjana I Cornu, Emily M Mace, Volker Spindler, Joern Dengjel, Frédéric Gros, Bodo Grimbacher, Fernando E Sepulveda, Yolanda R Carrasco, Virginia Andreani, Laura Gámez-Díaz

**Affiliations:** 1 Institute for Immunodeficiency, Center for Chronic Immunodeficiency, Medical Center–University of Freiburg, Faculty of Medicine, University of Freiburg, Freiburg, Germany; 2 Spemann Graduate School of Biology and Medicine (SGBM), University of Freiburg, Freiburg, Germany; 3 Faculty of Biology, University of Freiburg, Freiburg, Germany; 4 Laboratory of Molecular Basis of Altered Immune Homeostasis Inserm UMR 1163, Institut Imagine, Paris, France; 5 Inserm UMR S 1109 – Immuno-Rheumatologie Moléculaire, University of Strasbourg, Strasbourg, France; 6 INSERM U1151, CNRS UMR8253, Institut Necker Enfants Malades, Université Paris Cité, Paris, France; 7 Institute for Transfusion Medicine and Gene Therapy, Medical Center-University of Freiburg, Freiburg, Germany; 8 Department of Pediatrics, Columbia University Vagelos College of Physicians and Surgeons, New York, NY USA; 9 Department of Biomedicine, University of Basel, Basel, Switzerland; 10 Institute of Anatomy and Experimental Morphology, University Clinic Hamburg-Eppendorf, Hamburg, Germany; 11 Department of Biology, University of Fribourg, Fribourg, Switzerland; 12 Clinic of Rheumatology and Clinical Immunology, Center for Chronic Immunodeficiency (CCI), Medical Center, Faculty of Medicine, University of Freiburg, Freiburg, Germany; 13 DZIF – German Center for Infection Research, Satellite Center Freiburg, Freiburg, Germany; 14 CIBSS – Centre for Integrative Biological Signalling Studies, University of Freiburg, Freiburg, Germany; 15 RESIST – Cluster of Excellence 2155, Hannover Medical School, Satellite Center Freiburg, Freiburg, Germany; 16 Centre national de la recherche scientifique - CNRS, Villejuif, France; 17 Department of Immunology and Oncology, Centro Nacional de Biotecnología (CNB)-CSIC, Madrid, Spain

**Keywords:** Cell Adhesion, Polarity & Cytoskeleton, Immunology, Signal Transduction

## Abstract

Patients with lipopolysaccharide-responsive beige-like anchor protein (LRBA) deficiency typically suffer from severe B cell dysfunction. However, the underlying mechanisms remain incompletely understood. In this study, we identify non-muscle myosin IIA (NMIIA) as an interaction partner of LRBA in B cells, and uncover a role for LRBA in regulating actin cytoskeleton dynamics during B cell activation. LRBA-deficient B cells exhibit abnormal migration, impaired F-actin polymerization, and reduced B cell receptor signalling and polarization upon activation. In addition, LRBA deficiency severely disrupts immune synapse formation as evidenced by diminished central SMAC formation, reduced microtubule organizing center translocation and disrupted BCR and lysosome polarization. Consistent with these defects, internalization of the BCR-antigen complex is also impaired. Mechanistically, NMIIA activation, assessed by myosin light chain (MLC) phosphorylation, is reduced in LRBA-deficient cells. In addition, LRBA co-localizes with active NMIIA during both migration and immune synapse formation. Collectively, our findings establish LRBA as an important regulator of cytoskeleton dynamics during B cell activation, which may contribute to the defective humoral immunity observed in LRBA-deficient patients.

## Synopsis

Lipopolysaccharide-responsive beige-like anchor protein (LRBA) interacts with non-muscle myosin IIA (NMIIA) to regulate actin cytoskeleton dynamics in B cells, providing a mechanistic basis for the defective humoral immune response in LRBA deficiency.

- Loss of LRBA disrupts F-actin and myosin front-to-rear polarity, leading to abnormal B cell migration.
- LRBA deficiency impairs BCR signaling and polarization, immune synapse formation and antigen internalization.
- This is likely driven by reduced F-actin polymerization and NMIIA activation.
- The disrupted LRBA-NMIIA interaction may contribute to the humoral immune defects observed in LRBA-deficient patients.

## Abstract (web summary)

Lipopolysaccharide-responsive beige-like anchor protein (LRBA) interacts with non-muscle myosin IIA (NMIIA) to regulate actin cytoskeleton dynamics in B cells, providing a mechanistic basis for the defective humoral immune response in LRBA deficiency.

## Graphical abstract

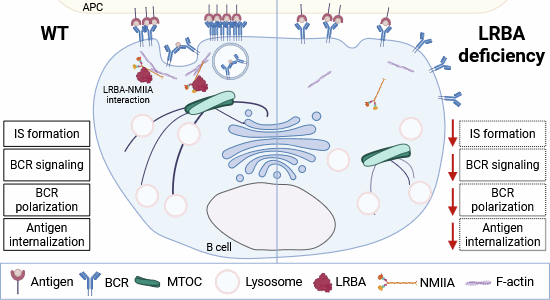

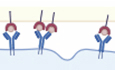

## Introduction

LRBA is known to regulate the recycling of cytotoxic T lymphocyte-associated protein 4 (CTLA-4) in regulatory T cells (Tregs), and to facilitate antigen presentation *via* autophagy through interactions with autophagy proteins, phosphoinositide-3-kinase regulatory subunit 4 (PIK3R4) and FYVE and coiled-coil domain adaptor 1 (FYCO1) in antigen-presenting cells (APC) (Janman et al, 2021; Lo et al, 2015; Sindram et al, 2025). This dual role of LRBA, controlling CTLA-4 trafficking and expression, and facilitating antigen presentation *via* autophagy, explains the T cell-driven autoimmunity and inflammation observed in patients with LRBA deficiency, a primary immunodeficiency syndrome caused by deleterious biallelic mutations in the *LRBA* gene (Alkhairy et al, 2016; Azizi et al, 2017; Gámez-Díaz et al, 2016; Habibi et al, 2019; Kostel Bal et al, 2017; Taghizade et al, 2023). Beyond T cell-mediated immune dysregulation symptoms, LRBA-deficient patients frequently present with recurrent infections and defective humoral response, characterized by hypogammaglobulinemia, poor vaccine responses, defective B cell activation, reduced switched memory B cells, and diminished plasma cells (Alkhairy et al, 2016; Azizi et al, 2017; Gámez-Díaz et al, 2016; Habibi et al, 2019; Kostel Bal et al, 2017; Taghizade et al, 2023). The latter has been previously associated with increased apoptosis in B cells likely due to impaired autophagy (Lopez-Herrera et al, 2012), which has been found essential for plasma cell maintenance (Arnold et al, 2016; Pengo et al, 2013; Pengo and Cenci, 2013). However, the precise mechanism underlying the dysfunction of B cell responses in LRBA deficiency has yet to be fully elucidated.

Cytoskeletal reorganization is pivotal for a proper B cell immune response *(*Harwood and Batista, 2010*;* Tolar, 2017*)*. Upon encountering an antigen, B cells rapidly reorganize their actin cytoskeleton to form the immune synapse (IS), a specialized integrin-rich supramolecular interface between the B cell and the APC (Carrasco, 2023; Wang et al, 2022). The IS serves as a platform for B cell receptor (BCR) clustering, which amplifies downstream signalling and facilitates antigen uptake for subsequent antigen processing and presentation (Batista et al, 2001; Song et al, 2013; Ulloa et al, 2022; Yuseff et al, 2011). Beyond IS formation and B cell activation, the cytoskeleton is crucial for cell adhesion and migration into lymphoid tissues and to sites of inflammation (Kamnev et al, 2021; Lub et al, 1997; Pearce et al, 2006). Cytoskeleton remodelling in B cells is tightly controlled through the action of actin-binding proteins and upstream regulators (Song et al, 2013; Tolar, 2017). Mutations in actin-regulating proteins can severely disrupt cytoskeletal dynamics and impair B cell functions, leading to immunodeficiency and defective humoral immunity (Dupré et al, 2026; Dupré and Prunier, 2023; He and Westerberg, 2019).

Non-muscle myosin IIA (NMIIA) is a motor protein composed of two heavy chains, two essential light chains (ELC) and two regulatory light chains (RLC, also referred to as myosin light chain (MLC)). Phosphorylation of the MLC regulates NMIIA activity, leading to the generation of contractile forces required for actin association and filament bundling (Pecci et al, 2018). In B cells, NMIIA has been associated with cell migration, immune synapse formation and antigen extraction (Hoogeboom et al, 2018; Seeley-Fallen et al, 2022; Wang et al, 2022). In this study, we describe a previously unrecognized interaction with NMIIA and reveal a novel role for LRBA in regulating cytoskeleton dynamics during B cell activation. We demonstrate that loss of LRBA results in abnormal migration, impaired F-actin organization, and reduced BCR polarization and BCR signalling responses following BCR stimulation. Furthermore, LRBA-deficient B cells fail to form a proper mature immune synapse and showed reduced internalization of the BCR and its antigen. Finally, our data suggest that LRBA is involved in the activation of NMIIA, as evidenced by reduced phosphorylation of MLC (pMLC) upon loss of LRBA. This newly identified role of LRBA in immune synapse formation and B cell activation may in part explain the poor humoral immune response observed in LRBA deficiency.

## Results

### LRBA associates with proteins involved in actin cytoskeleton dynamics and directly interacts with NMIIA in B lymphocytes

**Figure 1:**
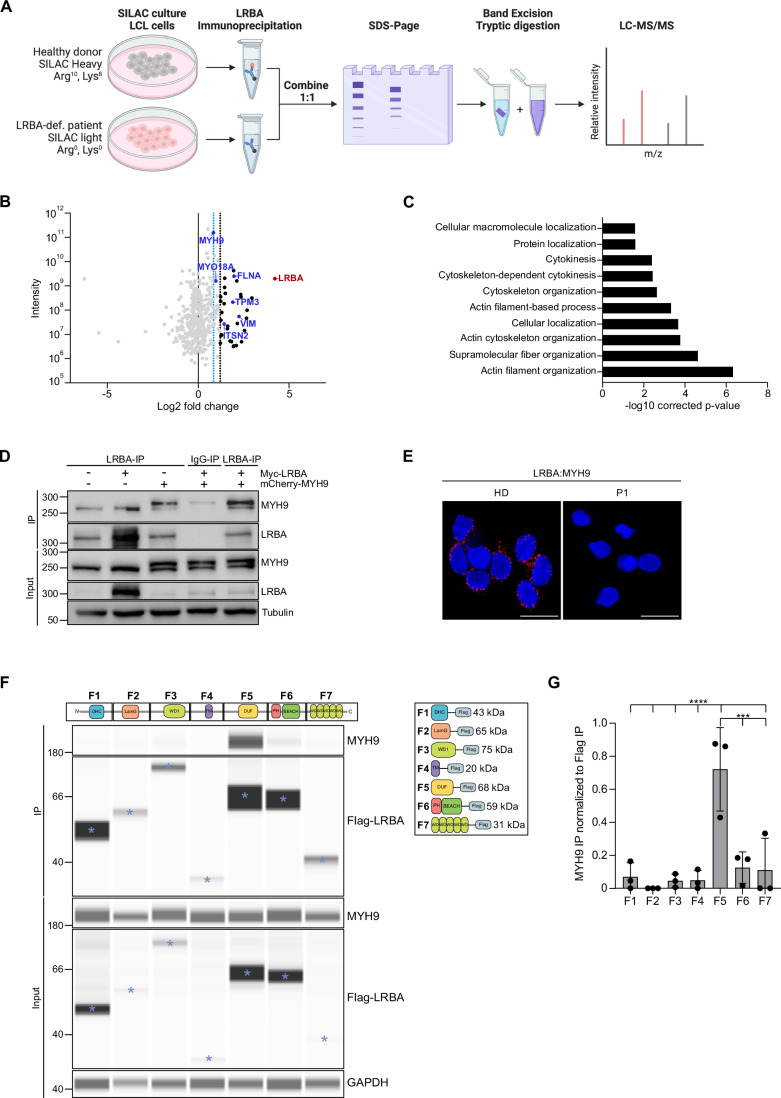
LRBA interacts with non-muscle myosin IIA. (**A**) Schematic representation of stable isotope labeling by amino acids in cell culture (SILAC)-immunoprecipitation (IP)-liquid chromatography-tandem mass spectrometry (LC-MS/MS) to identify potential interaction partners of LRBA in lymphoblastic B cell lines (LCL). (**B**) Scatter plot showing significantly enriched proteins in LCLs from a healthy donor (HD) compared to LRBA-P1. Protein abundance is plotted against the log2 fold change: HD/LRBA-P1. Significantly enriched proteins evaluated as potential LRBA interactors by co-IP are shown in blue font. The black dotted non-axial vertical line indicates the conventional significance threshold (*P* < 0.05) and is set at log2 fold change >1.2, while the light-blue dotted non-axial vertical line defines an empirically defined candidate region of interest with a log2 fold change >0.82 and significance of *P* < 0.13 that is enriched for cytoskeleton-associated proteins and was used for follow-up experiments. The plot was generated using *n* = 2 biological replicates. (**C**) Gene ontology (GO) enrichment analysis of the 33 significantly enriched proteins shown in (**B**). Bars show the negative log10 of the Bejamini–Hochberg false discovery rate (FDR)-corrected *P* value generated by the GO analysis tool. No additional statistical comparisons between GO terms were performed. (**D**) WT HEK293T cells were co-transfected with Myc-LRBA and mCherry-MYH9 plasmids. Pull-down was performed with anti-LRBA and immunoblotted for MYH9. Tubulin was used as a loading control for the input. *n* = 1 biological replicate. (**E**) Proximity ligation assay (PLA) in LCLs from a HD and LRBA-P1. Nuclear DAPI staining is shown in blue and LRBA-MYH9 interaction is shown as a red signal. Scale bar: 20 μm. *n* = 2 biological replicates. (**F**) WT HEK293T cells were co-transfected with a plasmid encoding full length, mCherry-tagged MYH9 and Flag-tagged LRBA protein domains (fragments 1–7). Flag-LRBA was immunoprecipitated with anti-Flag, and western blots were probed with anti-MYH9. Protein detection was performed by simple western automated western blot (WES) analysis. Tubulin loading control signals were obtained in separate capillaries within the same WES system. Schematic representation of plasmids containing the different LRBA protein domains is shown on the top and right. Asterisks in the western blots indicate the LRBA protein domains. (**G**) Ratio of MYH9/LRBA fragment detected by co-IP. Each dot represents one biological replicate, while bars represent the mean ± SD of *n* = 3 biological replicates. Statistical analysis was performed using two-way ANOVA with Tukey’s multiple comparison test (**G**), ***P < 0.001 (F5 vs. F6: *P* = 0.0002, F5 vs. F7: *P* = 0.0001), ****P < 0.0001. Source data are available online for this figure.

**Figure EV1:**
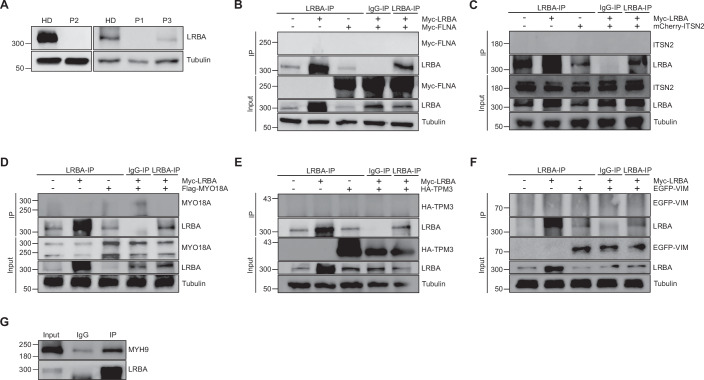
LRBA does not interact with FLNA, ITSN2, MYO18A, TPM3 and VIM. (**A**) Immunoblot analysis of LRBA protein expression in LCL cells from HD and LRBA-P1, P2 and P3. (**B**–**F**) WT HEK293T cells were co-transfected with Myc-LRBA plasmid and either (**B**) Myc-FLNA plasmid, (**C**) mCherry-ITSN2, (**D**) Flag-MYO18A, (**E**) HA-TPM3 or (**F**) EGFP-VIM plasmid. Immunoprecipitation was performed with anti-LRBA and immunoblotted for (**B**) FLNA using anti-Myc, (**C**) mCherry-ITSN2 using anti-ITSN2, (**D**) Flag-MYO18A using anti-MYO18A, (**E**) HA-TPM3 using anti-HA or (**F**) EGFP-VIM using anti-GFP. Tubulin was used as a loading control for the input. *n* = 1 biological replicate. (**G**) HD primary B cells were stimulated with 250 ng/ml CD40L and 10 µg/ml IL-21 for 24 h. Immunoprecipitation was performed using anti-LRBA and immunoblotted for MYH9. *n* = 1 biological replicate.

Using stable isotope labelling by amino acids in cell culture (SILAC) combined with immunoprecipitation (IP) and liquid chromatography-tandem mass spectrometry (LC-MS/MS) (Kaeser-Pebernard et al, 2022) in lymphoblastic B cell lines (LCL) from a healthy donor (HD) and an LRBA-deficient patient (Figs. 1A and EV1A), we identified 33 significantly enriched novel potential LRBA interactors (Fig. 1B; Dataset EV1). According to gene ontology (GO) term enrichment analysis, the enriched proteins are mostly involved in actin cytoskeleton dynamics (Fig. 1C). Significant potential interactors (*P* < 0.05) included filamin A (FLNA), intersectin-2 (ITSN2), tropomyosin 3 (TPM3) and vimentin (VIM). In addition, we considered proteins functionally linked to cytoskeleton dynamics that were strongly enriched with a *p*-value close to the significance threshold, including myosin heavy chain 9 (MYH9) and myosin 18 A (MYO18A) (Fig. 1B; Dataset EV1). All six enriched cytoskeleton-associated proteins were evaluated as potential LRBA interactors. Co-immunoprecipitation (co-IP) experiments in HEK293T cells revealed a specific interaction between LRBA and MYH9 (Fig. 1D), whereas no interaction was detected with FLNA, ITSN2, MYO18A, TPM3, and VIM (Fig. EV1B–F). This LRBA:MYH9 interaction was further validated at endogenous levels in HD cells using proximity ligation assay (PLA) in LCL cells and co-IP in primary B cells upon stimulation with CD40L and IL-21 (Figs. 1E and EV1G). To map the interaction domain, co-IP experiments were performed following co-expression of Flag-tagged LRBA fragments (F1 to F7) with mCherry-tagged full-length MYH9 in HEK293T cells (Fig. 1F,G). These analyses revealed an interaction between mCherry-MYH9 and fragment 5 (F5) of LRBA (Fig. 1F,G). Notably, LRBA-F5 contains the domain of unknown function (DUF) 1088. As *MYH9* encodes the heavy chain of non-muscle myosin IIA (NMIIA), a cytoplasmic myosin essential for mechanical force generation and cytoskeletal organization (Pecci et al, 2018), our findings suggest a role for LRBA in the regulation of the actin cytoskeleton.

### Loss of LRBA results in abnormal B cell migration

**Figure EV2:**
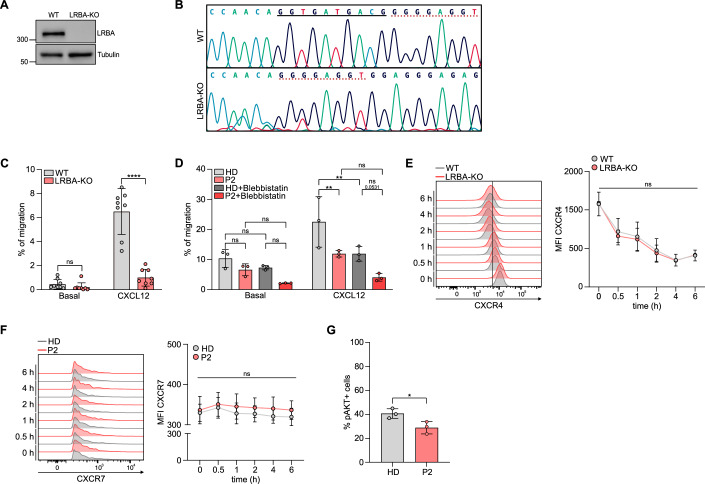
Loss of LRBA does not affect CXCR4 and CXCR7 expression. (**A**) Immunoblot analysis of LRBA protein expression in WT and LRBA-KO Ramos B cells generated using the CRISPR-Cas9 system showing absence of LRBA. (**B**) Sequencing analysis of *LRBA* exon 2 showing a deletion of 10 nucleotides in LRBA-KO cells compared to the WT sequence. (**C**) Percentage of chemokine-directed cell migration was evaluated in WT (grey) and LRBA-KO (red) Ramos B cells. Cells migrated through a 5 μm membrane towards either RPMI only (Basal) or 100 ng/ml CXCL12 for 6 h. Each dot represents one biological replicate, while bars represent mean ± SD of *n* = 8 biological replicates. (**D**) Percentage of chemokine-directed cell migration was evaluated in HD (grey) and LRBA-P2 (red) LCL cells. Cells were left untreated (light colors) or treated with 10 µM Blebbistatin (dark colors) and migrated through a 5 μm membrane towards either RPMI only (Basal) or 100 ng/ml CXCL12 for 6 h. Each dot represents one biological replicate, while bars represent mean ± SD of *n* = 3 biological replicates. (**E**) WT (grey) and LRBA-KO (red) Ramos B cells and (**F**) LCL cells from a HD (grey) and LRBA-P2 (red) were stimulated with 100 ng/ml CXCL12 for the indicated time points and analyzed for (**E**) CXCR4 and (**F**) CXCR7 expression. MFI of the total cell population is shown in (**E**) while MFI of CXCR7+ cells is shown in (**F**). Each dot represents the mean ± SEM from *n* = 3 biological replicates. (**G**) Percentage of pAKT+ HD (grey) and LRBA-P2 (red) LCL cells. Each dot represents the mean of *n* = 2 technical replicates, while bars represent mean ± SD of *n* = 3 biological replicates. Statistical analysis was performed using Welch’s *t* test (**G**) or two-way ANOVA with Tukey’s multiple comparison test (**C**–**F**), *P < 0.05 (**G**: *P* = 0.0371) **P < 0.01 (**D**: WT vs. KO: *P* = 0.0074, **D**: WT vs. WT-Blebbistatin: *P* = 0.0074), ****P < 0.0001, ns: not significant.

**Figure 2:**
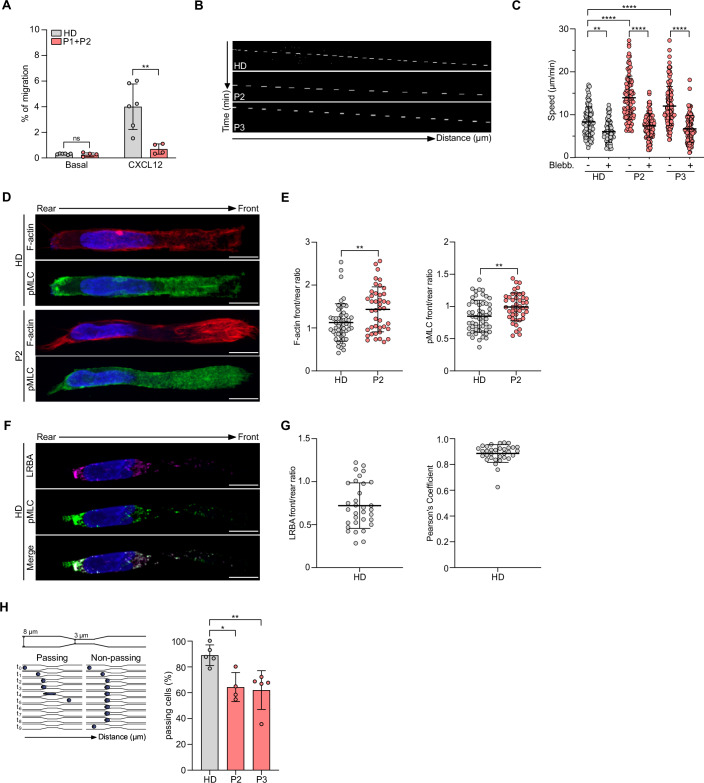
LRBA-deficient B cells show abnormal chemokine-directed and spontaneous cell migration. (**A**) Percentage of chemokine-directed cell migration was evaluated in LCL cells from a HD (grey) and LRBA-P1 and P2 (red). Cells migrated through a 5 μm membrane towards either RPMI only (Basal) or 100 ng/ml CXCL12 for 6 h. Each dot represents one biological replicate, while bars represent mean ± SD of HD: *n* = 6 and P1 + P2: *n* = 2 (data pooled) biological replicates. (**B**) Representative space-time kymographs showing the trajectory of a single HD (top) and LRBA-P2 (middle) and LRBA-P3 (bottom) LCL cell migrating in fibronectin-coated microchannels. Nuclear position of a representative single cell migrating along the microchannels (in white) is displayed over different time points along the field of view. The interval between images is 2 min; the slope of the trajectory reflects the migration speed. (**C**) Analysis of (**B**) based on the mean instantaneous speed of HD (grey) and LRBA-P2 and P3 (red) LCL cells untreated or treated with 25 µM Blebbistatin. Each dot represents one cell (HD: *n* = 92, HD-Blebb: *n* = 69, P2: *n* = 90, P2-Blebb: *n* = 94, P3: *n* = 119, P3-Blebb: *n* = 97) from *n* = 3 biological replicates, mean ± SD shown by the black line and error bars. (**D**) Representative confocal microscopy images of HD and LRBA-P2 LCL cells which were let to migrate in microchannels, fixed and stained for F-actin using Phalloidin (red), pMLC (green) and Hoechst (blue). Scale bar: 10 μm. (**E**) Analysis of F-actin (left) and pMLC (right) distribution between the front and the rear in HD (grey) and LRBA-P2 (red) LCL cells. Each dot represents one cell (F-actin: HD: *n* = 51, P2: *n* = 42; pMLC: HD: *n *= 54, P2: *n* = 44) from *n* = 3 biological replicates, mean ± SD shown by the black line and error bars. (**F**) Representative confocal microscopy images of HD LCL cells which were let to migrate in microchannels, fixed and stained for LRBA (magenta), pMLC (green) and Hoechst (blue). Scale bar: 10 μm. (**G**) Analysis of LRBA distribution between the front and rear in HD (grey) LCL cells (left) and co-localization of pMLC and LRBA shown as Pearson’s coefficient (right). Each dot represents one cell (Front/rear: *n* = 32, Pearson: *n* = 33) from *n* = 3 biological replicates, mean ± SD shown by the black line and error bars. (**H**) Left: Schematic representation of cell migration in microchannels containing 3 µm constrictions, illustrating the two observed behaviours: a passing cell that deforms and crosses the constriction, and a non‑passing cell that repeatedly attempts to access the constriction but fails to enter and eventually retreats. Right: Percentage of HD (grey) and LRBA-P2 and P3 LCL cells (red) that migrated through constrictions. Each dot represents the mean of one biological replicate, while bars represent mean ± SD of *n* = 4–5 biological replicates. Statistical analysis was performed using Welch’s *t* test (**E**) or two-way ANOVA with Tukey’s multiple comparisons test (**A**, **C**, **H**), *P < 0.05 (**H**: *P* = 0.0120), **P < 0.01 (**A**: *P* = 0.0051, **C**: *P* = 0.0029, **E**, F-actin: *P* = 0.0034, **E**, pMLC: *P* = 0.0028, **H**: *P* = 0.0072), ****P < 0.0001, ns: not significant. Source data are available online for this figure.

In B cells, NMIIA has been linked to cell migration, immune synapse formation and antigen extraction *via* the BCR (Hoogeboom et al, 2018; Seeley-Fallen et al, 2022; Wang et al, 2022). Given our observation that LRBA interacts with NMIIA and that *Myh9* knock-out (KO) murine B cells showed reduced migration towards CXCL12 (Hoogeboom et al, 2018), we investigated whether LRBA deficiency affects B cell chemotaxis and spontaneous migration in LCL cells from LRBA-deficient patients (Fig. EV1A) and LRBA-KO Ramos B cells generated with CRISPR-Cas9 system (Fig. EV2A,B). After 6 h of stimulation with CXCL12, LRBA-deficient LCL cells and LRBA-KO Ramos B cells showed significantly reduced migration in a transwell system towards the chemokine in comparison to control cells (Figs. 2A and EV2C). Similarly, HD LCL cells treated with Blebbistatin, a myosin II ATPase inhibitor (Straight et al, 2003), exhibited reduced migration towards CXCL12 (Fig. EV2D), phenocopying the migration defect observed in LRBA-deficient cells. CXCL12 recruits B cells through its interaction with CXCR4 or CXCR7, which in turn triggers cell migration (Beck et al, 2014). We did not detect any differences in CXCR4 surface expression and internalization between WT and LRBA-KO Ramos B cells, or in CXCR7 dynamics in LCL cells (as LCL cells do not express CXCR4), indicating that reduced cell migration is not due to abnormal CXCL12 receptor expression (Fig. EV2E,F). In contrast, in microchannels measuring spontaneous ameboid migration (Heuzé et al, 2011), LRBA-deficient LCL cells migrated faster than HD LCL cells (Fig. 2B,C). Blebbistatin treatment reduced the migration speed of LRBA-deficient cells to control levels, suggesting abnormalities in NMIIA regulation in the absence of LRBA (Fig. 2C). In addition, LRBA-deficient LCL cells showed reduced AKT activity (Fig. EV2G), which has been linked to decreased F-actin stabilization (Qian et al, 2004). In fact, Phalloidin staining revealed accumulation of F-actin at the cell front in LRBA-deficient LCL cells compared to HD cells, indicating a disrupted front-rear F-actin polarity during confined migration (Fig. 2D,E). In addition, spatial distribution analysis of pMLC identified an accumulation of pMLC at the cell rear in HD LCL cells, whereas LRBA-deficient LCL cells displayed a uniform pMLC distribution in microchannels (Fig. 2D,E). Interestingly, LRBA was found at the rear and perinuclear region of the cell, partially overlapping with pMLC (Fig. 2F,G), supporting a role of LRBA in organizing actomyosin polarity during migration. Finally, we evaluated the ability of LCL cells to navigate through complex confined spaces using microchannels with 3 µm constrictions mimicking tissue barriers (Heuzé et al, 2011; Vargas et al, 2014). LRBA-deficient LCL cells showed reduced ability to pass through confined spaces (Fig. 2H), likely due to impaired nuclear deformability. This is consistent with the observed reduced migration in transwell assays (Figs. 2A and EV2C,D). Altogether, our data indicate that LRBA is involved in the spatial organization of F-actin and NMIIA, which is required for proper B cell migration.

### LRBA deficiency reduces BCR-mediated cell activation and BCR polarization

**Figure 3:**
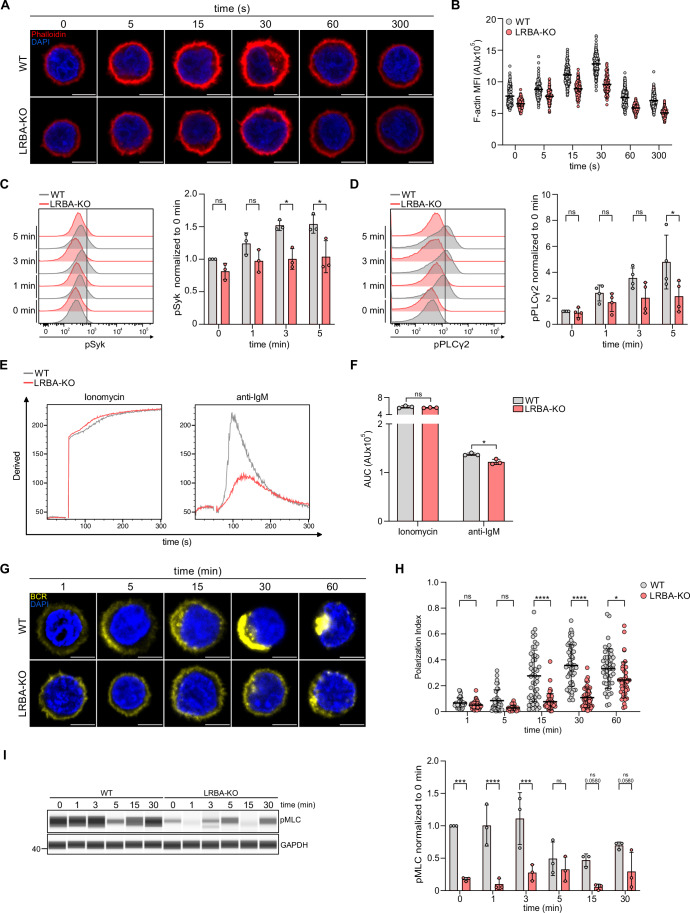
Reduced BCR downstream signalling in B cells lacking LRBA. (**A**) Representative confocal microscopy images of WT and LRBA-KO Ramos B cells stained for F-actin using Phalloidin (red) and DAPI (blue) after stimulation with 5 μg/ml anti-IgM for the indicated time points. Scale bar: 5 μm. (**B**) Quantification of F-actin mean fluorescence intensity (MFI) over time in WT (grey) and LRBA-KO (red) Ramos B cells. Each dot represents one cell (WT-0s: *n* = 241, KO-0s: *n* = 205, WT-5s: *n* = 171, KO-5s: *n* = 214, WT-15s: *n *= 222, KO-15s: *n* = 199, WT-30s: *n* = 287, KO-30s: *n* = 189, WT-60s: *n* = 341, KO-60s: *n* = 334, WT-300s: *n* = 336, KO-300s: *n* = 264), while the black horizontal line represents the mean from *n* = 2 biological replicates. (**C**, **D**) WT (grey) and LRBA-KO (red) Ramos B cells were stimulated with 5 μg/ml anti-IgM for the indicated time points and analyzed for (**C**) phosphorylation of Syk (pSyk). Left: Representative histograms of pSyk MFI of one experiment. Right: Analysis of MFI normalized to pSyk WT 0 min, and (**D**) phosphorylation of PLCy2 (pPLCy2) analyzed by flow cytometry. Left: Representative histograms of pPLCy2 MFI of one experiment. Right: Analysis of MFI normalized to pPLCy2 WT 0 min. Each dot represents one biological replicate, while bars represent mean ± SD of *n* = 3–4 biological replicates. (**E**, **F**) Calcium flux responses were evaluated in Ramos B cells after stimulation with either 2 μM Ionomycin or 0.5 μg/ml anti-IgM. (**E**) Representative flow cytometry histograms of WT (grey) and LRBA-KO (red) Ramos B cells. (**F**) Quantification of area under the curve (AUC) using FlowJo software. Each dot represents one biological replicate, while bars represent the mean ± SD of *n* = 3 biological replicates. (**G**) Representative confocal microscopy images of WT and LRBA-KO Ramos B cells stimulated with 5 μg/ml AlexaFluor647 anti-IgM for the indicated time points. BCR staining is shown in yellow and DAPI in blue. Scale bar: 5 μm. (**H**) Quantification of the BCR polarization index in WT (grey) and LRBA-KO (red) Ramos B cells, which measures the displacement of the fluorescence center of mass relative to the cell body. Positive values indicate polarization of the fluorescence signal towards one side of the cell, whereas values close to 0 indicate symmetric distribution. Each dot represents one cell (WT-1 min: *n* = 32, KO-1 min: *n* = 40, WT-5 min: *n* = 36, KO-5 min: *n* = 29, WT-15 min: *n* = 48, KO-15 min: *n* = 50, WT-30 min: *n* = 55, KO-30 min: *n* = 50, WT-60 min: *n* = 45, KO-60 min: *n* = 41) from *n* = 3 biological replicates, mean ± SD shown by the black line and error bars. (**I**) Phosphorylation of MLC was measured in WT (grey) and LRBA-KO (red) Ramos B cells after stimulation with 5 μg/ml anti-IgM for the indicated time points. Left: Representative immunoblot analyses of pMLC expression of one experiment. Protein detection was performed by Simple Western automated western blot (WES) analysis. GAPDH loading control signals were obtained in separate capillaries within the same WES system. Right: Densitometry analyses of pMLC immunoblots normalized first to GAPDH and then to pMLC WT 0 min. Each dot represents one biological replicate, while bars represent mean ± SD of *n* = 3 biological replicates. AU Arbitrary Units. Statistical analysis of all experiments was performed using Two-way ANOVA with Tukey's (**C**, **D**) or Holm–Sidak's (**H**, **I**) multiple comparison test or Welch’s *t* test (**F**). *P < 0.05 (**C**: 3 min: *P* = 0.0129, **C**: 5 min: *P* = 0.0161, **D**: *P* = 0.0277, **F**: *P* = 0.0217, **H**: *P* = 0.0402), ***P < 0.001 (**I**: 0 min: *P* = 0.0003, 3 min: *P* = 0.0002), ****P < 0.0001, ns: not significant. Source data are available online for this figure.

**Figure EV3:**
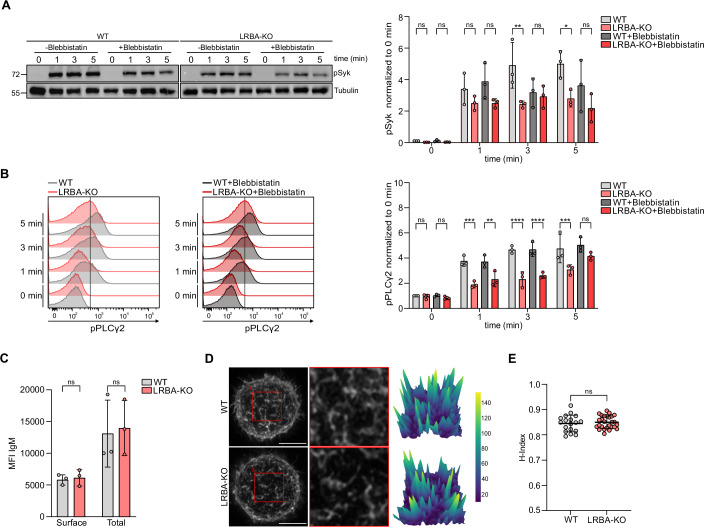
Blebbistatin treatment does not alter BCR downstream signaling. (**A**, **B**) WT (grey) and LRBA-KO (red) Ramos B cells were left untreated (light color) or treated with 50 μM Blebbistatin (dark color) for 30 min and stimulated with 5 μg/ml anti-IgM for the indicated time points. (**A**) Phosphorylation of Syk (pSyk) analyzed by western blot. Left: Representative immunoblot analyses of pSyk expression of one experiment. Tubulin was used as loading control. Right: Densitometry analyses of pSyk immunoblots normalized first to Tubulin and to then pSyk WT 0 min. (**B**) Phosphorylation of PLCy2 (pPLCy2) analyzed by flow cytometry. Left: Representative histograms of pPLCy2 MFI of one experiment. Right: Analysis of MFI normalized to pPLCy2 WT 0 min. Each dot represents one biological replicate, while bars represent mean ± SD of *n* = 3 biological replicates. (**C**) Surface and intracellular expression of IgM in WT (grey) and LRBA-KO (red) Ramos B cells. Each dot represents the mean of *n* = 2 technical replicates, while bars represent mean ± SD of *n* = 3 biological replicates. (**D**, **E**) WT and LRBA-KO Ramos B cells were stained with AF647-conjugated anti-IgM Fab fragments, fixed and imaged using confocal microscopy. (**D**) Representative images of BCR distribution after Airyscan processing and maximum intensity projection across the acquired z-stacks (left). Region of interest (ROI) in red square shown as a 2D image (center) and 3D surface plot generated from the ROI with the color-coded *z* axis representing the intensity values. Scale bar: 5 µm. (**E**) Quantification of the distribution of the BCR by the Hopkins (H) index in WT (grey) and LRBA-KO (red) Ramos B cells. Each dot represents one cell (WT: *n* = 18, KO: *n* = 27) from *n* = 3 biological replicates, mean ± SD shown by the black line and error bars. Statistical analysis of all experiments was performed using Welch’s *t* test (**C**, **E**) or two-way ANOVA with Tukey’s multiple comparisons test (**A**, **B**), *P < 0.05 (**A**: *P* = 0.011), **P < 0.01 (**A**: *P* = 0.0038, **B**: *P* = 0.0057), ***P < 0.001 (**B**, 1 min: *P* = 0.0002, **B**, 5 min: *P* = 0.0007), ****P < 0.0001. All other comparisons are ns: not significant.

In addition to cell migration, actin dynamics are also critical for effective BCR signalling *(*Bhanja et al, 2022*;* Liu et al, 2012*)*, as demonstrated in patients with mutations in DOCK8 and WASp (Bai et al, 2016; Gu et al, 2024). Upon antigen-BCR engagement, B cells undergo rapid F-actin polymerization to form BCR microclusters, which are essential for BCR downstream signalling and subsequent calcium release (Li et al, 2018). In fact, LRBA-KO Ramos B cells showed reduced F-actin polymerization compared to WT cells at all evaluated time points (0, 5, 15, 30, 60, 300 sec) after anti-IgM stimulation (Fig. 3A,B). Notably, the reduction in F-actin observed already at baseline (*t *= 0 s) suggests that LRBA contributes to constitutive F-actin homeostasis. In addition, LRBA-KO Ramos B cells showed a reduction in phosphorylation of Syk and PLCγ2 (Fig. 3C,D) as well as reduced intracellular calcium release upon anti-IgM stimulation (Fig. 3E,F), consistent with impaired upstream actin-dependent BCR signalling. BCR-independent calcium release using ionomycin, a calcium ionophore, showed no differences between WT and LRBA-KO Ramos B cells (Fig. 3E,F), indicating that the defects in calcium release are BCR-dependent. Interestingly, inhibition of NMIIA by Blebbistatin treatment of WT and LRBA-KO cells did not alter BCR downstream signalling compared to untreated controls (Fig. EV3A,B). This suggests that the impaired BCR signalling in the absence of LRBA is not driven by NMIIA motor activity, but rather by reduced F-actin polymerization. Proper B cell activation also requires BCR polarization (Arbogast et al, 2019). Therefore, we stimulated WT and LRBA-KO Ramos B cells with soluble AF647-conjugated anti-IgM for up to 60 min. WT Ramos B cells formed a polarized BCR cap from 15 min onward, whereas LRBA-KO Ramos B cells showed delayed polarization, becoming evident only after 60 min of stimulation (Fig. 3G,H). This BCR polarization is consistent with the observed defective F-actin polymerization, which is required for directional BCR distribution (Liu et al, 2012). To characterize NMIIA involvement, we measured phosphorylation of MLC, as a readout of NMIIA motor activity. LRBA-KO Ramos B cells showed markedly reduced basal pMLC levels compared to WT cells, indicating impaired basal NMIIA activation, which remained reduced post-stimulation (Fig. 3I). Together with the reduced F-actin polymerization (Fig. 3A,B), these data suggest a disrupted actomyosin homeostasis in LRBA-KO Ramos B cells. Of note, surface and intracellular IgM expression and BCR distribution at basal levels were similar between WT and LRBA-KO Ramos B cells (Fig. EV3C–E), excluding abnormal IgM expression and distribution as a cause of reduced BCR responses. Overall, these data indicate that LRBA is likely required for maintaining F-actin and actomyosin homeostasis both at baseline state and upon BCR stimulation, which may underlie efficient BCR downstream signaling and BCR polarization.

### LRBA-deficient B cells fail to form a proper immune synapse

**Figure 4:**
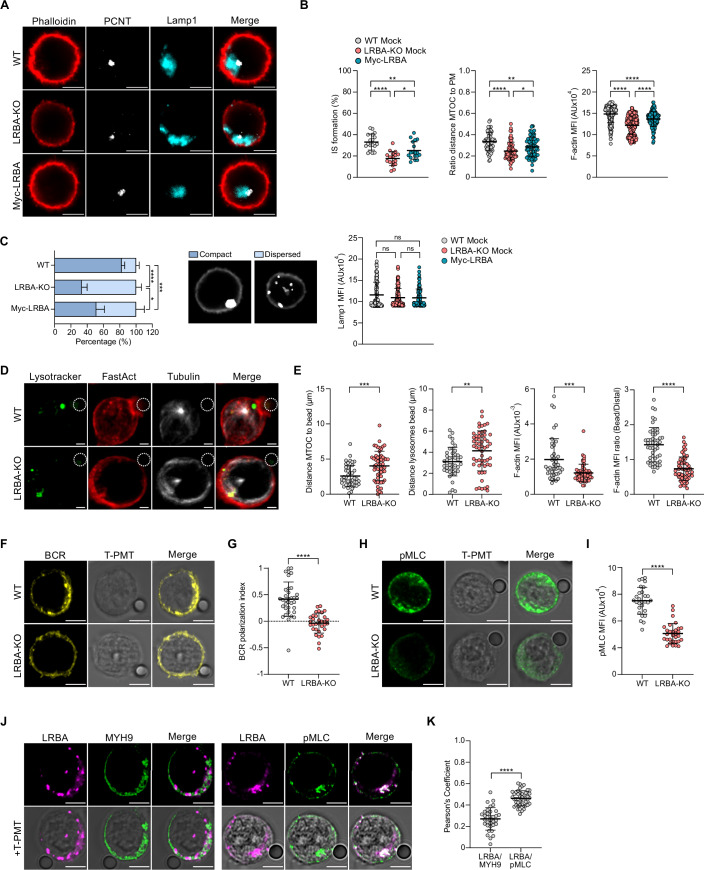
LRBA-deficient B lymphocytes show an abnormal immune synapse formation. (**A**–**C**) IS analysis of WT (grey), LRBA-KO (red) and reconstituted Myc-LRBA (teal) Ramos B cells. Cells were incubated on glass slides coated with 15 μg/ml anti-IgM for 15 min. (**A**) Representative confocal microscopy images of IS formation. F-actin was stained with Phalloidin (red), MTOC with anti-pericentrin (PCNT) (white), and lysosomes with anti-Lamp1 (cyan). Scale bar: 5 μm. (**B**) Analysis of IS formation frequency (left) (each dot represents one image, WT: *n* = 18, KO: *n* = 17, Myc: *n* = 18), ratio of the distance of MTOC to the plasma membrane (PM) (center) (each dot represents one cell, WT: *n* = 65, KO: *n* = 65, Myc: *n* = 69), and F-actin MFI (right) (each dot represents one cell, WT: *n* = 219, KO: *n* = 285, Myc: *n* = 135). *n* = 3 biological replicates, mean ± SD shown by the black line and error bars. (**C**) Analysis of lysosome distribution during IS formation. Percentage of cells with compact (dark-blue) or dispersed (light-blue) lysosome distribution (left) and MFI of Lamp1+ dots (right). Each dot represents one Lamp1+ dot (MFI) (WT: *n* = 107, KO: *n* = 154, Myc: *n* = 137), while bars and lines represent the mean ± SD of 45 cells from *n* = 3 biological replicates. (**D**–**K**) IS analysis of WT (grey) and LRBA-KO (red) Ramos B cells after incubation with anti-IgM-coated 3 μm beads for 30 min. (**D**) Representative confocal microscopy images of Ramos cells stained for lysosomes with lysotracker (green), F-actin with SPY555-FastAct (red) and Tubulin with SPY650-tubulin (white). Scale bar: 2 μm. (**E**) Quantification of the distance of the MTOC to the bead (left), the distance of lysosomes to the bead (center-left), F-actin MFI (center-right) and the F-actin MFI ratio at the bead vs the distal side of the cell (right). Each dot represents one cell (WT: *n* = 43-48, KO: *n* = 57) from *n* = 3 biological replicates, mean ± SD shown by the black line and error bars. (**F**) Representative confocal microscopy images of Ramos cells in transmitted-light (T-PMT) and stained for the BCR using anti-lambda (yellow). Scale bar: 5 μm. (**G**) Quantification of the BCR polarization index, calculated from the displacement of the fluorescence center of mass relative to the cell center toward the bead-contact site. Positive values indicate polarization toward the bead, values near 0 indicate a symmetric distribution, and negative values indicate polarization away from the bead. Each dot represents one cell (WT: *n* = 35, KO: *n* = 32) from *n *= 3 biological replicates, mean ± SD shown by the black line and error bars. (**H**) Representative confocal microscopy images of Ramos cells in transmitted-light (T-PMT) and stained for pMLC (green). Scale bar: 5 μm. (**I**) Analysis of pMLC MFI of WT (grey) and LRBA-KO (red) Ramos B cells. Each dot represents one cell (WT: *n* = 28, KO: *n *= 30) from *n *= 3 biological replicates, mean ± SD shown by the black line and error bars. (**J**) Representative confocal microscopy images of Ramos WT cells stained for MYH9 or pMLC (green) and for LRBA (magenta). Top panel is shown without and bottom panel with transmitted-light (T-PMT). Left: LRBA and MYH9. Right: LRBA and pMLC. Scale bar: 5 μm. (**K**) Co-localization of MYH9 and LRBA and pMLC and LRBA shown as Pearson’s coefficient. Each dot represents one cell (MYH9: *n* = 34, pMLC: *n* = 47) from *n* = 3 biological replicates, mean ± SD shown by the black line and error bars. AU Arbitrary Units. Statistical analysis was performed using Welch’s *t* test (**E**, **G**, **I**, **K**), one-way ANOVA with Tukey’s multiple comparison test (**B**, **C**-MFI) or two-way ANOVA with Tukey’s multiple comparisons test (**C**—lysosome distribution), *P < 0.05 (**B**, left: *P* = 0.0174, **B**, center: *P* = 0.0124, **C**: *P* = 0.0301), **P < 0.01 (**B**, left: *P* = 0.0083, **B**, center: *P* = 0.0034, **E**: *P* = 0.0022), ***P < 0.001 (**C**: *P* = 0.0006, **E**, left: *P* = 0.0001, **E**, center-right: *P* = 0.0002), ****P < 0.0001, ns: not significant. Source data are available online for this figure.

**Figure EV4:**
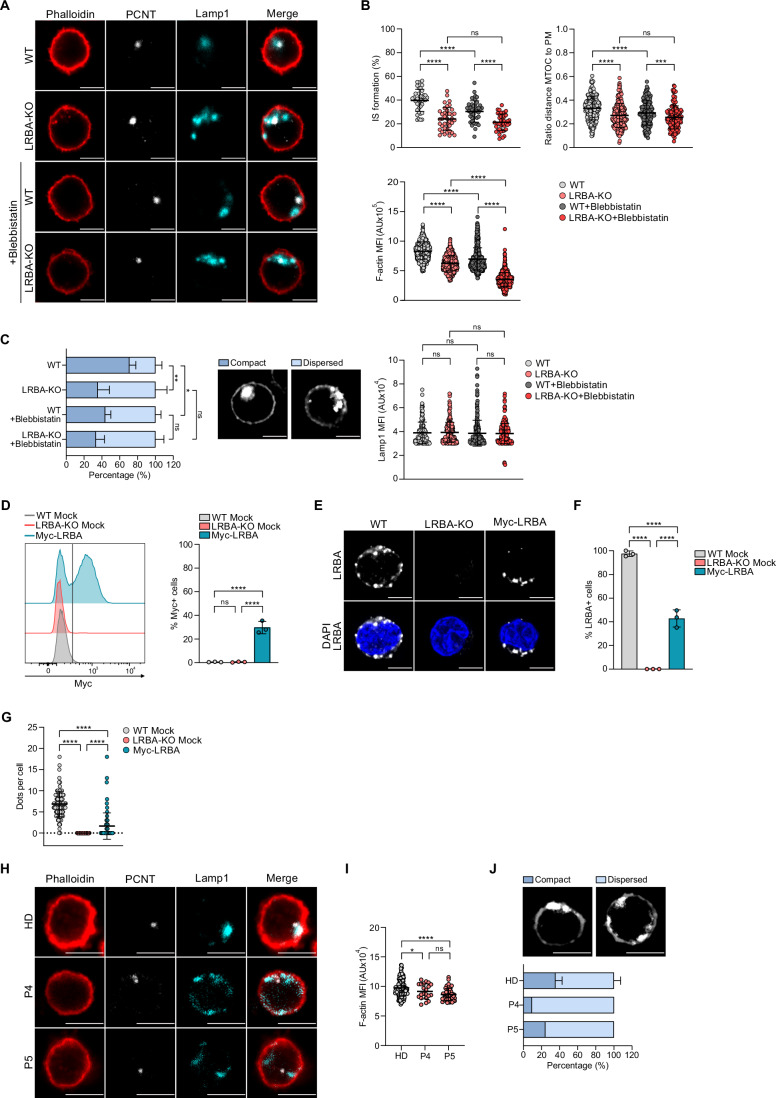
LRBA-deficient cells show abnormal IS formation which is mimicked by treating WT cells with Blebbistatin. (**A**–**C**) IS analysis of WT (grey) and LRBA-KO (red) Ramos B cells untreated or treated with 50 μM Blebbistatin for 30 min and incubated on glass slides coated with 15 μg/ml anti-IgM for 15 min. (**A**) Representative confocal microscopy images of IS formation in Ramos B cells. F-actin was stained with Phalloidin (red), microtubule organizing center (MTOC) with anti-pericentrin (PCNT) (white), and lysosomes with anti-Lamp1 (cyan). Scale bar: 5 μm. (**B**) Analysis of IS formation frequency determined by Phalloidin+Pericentrin+Lamp1+ signal (top-left) (each dot represents one image, WT: *n* = 38, KO: *n* = 40, WT-Blebb: *n* = 40, KO-Blebb: *n* = 40), ratio of the distance of MTOC to the plasma membrane (PM) (top-right) (each dot represents one cell, WT: *n* = 275, KO: *n* = 242, WT-Blebb: *n* = 184, KO-Blebb: *n* = 168), and F-actin MFI (bottom-left) (each dot represents one cell, WT: *n* = 591, KO: *n* = 581, WT-Blebb: *n* = 548, KO-Blebb: *n *= 508). *n* = 3 biological replicates, mean ± SD shown by the black line and error bars. (**C**) Analysis of lysosome distribution during IS formation. Percentage of cells with compact (dark-blue) or dispersed (light-blue) lysosome distribution (left) and MFI of Lamp1+ dots (right). Each dot represents one Lamp1+ dot (MFI), while bars and lines represent the mean ± SD of 48 cells from *n* = 3 biological replicates. (**D**–**G**) LRBA was re-expressed in LRBA-KO Ramos B cells using Myc-tagged LRBA mRNA nucleofection. (**D**) Representative histogram and quantification of the percentage of Myc-LRBA+ cells of WT (grey), LRBA-KO (red) and Myc-LRBA (teal) Ramos B cells using anti-Myc antibody. Each dot represents one biological replicate, while bars represent the mean ± SD of *n* = 3 biological replicates. (**E**) Representative images of LRBA expression in Ramos B cells stained for LRBA (white) and DAPI (blue). Scale bar: 5 μm. (**F**, **G**) Analysis of (**F**) percentage of LRBA expressing cells and (**G**) LRBA dots per cell in WT (grey), LRBA-KO (red) and Myc-LRBA (teal) Ramos B cells. In (**D**, **F**), each dot represents one biological replicate, while bars represent the mean ± SD of *n* = 3 biological replicates. In (**G**), each dot represents one cell (WT: *n* = 125, KO: *n* = 165, Myc: *n* = 38) from *n* = 3 biological replicates. (**H**–**J**) IS analysis of naive B cells from healthy donors (HD) and LRBA-P4 and P5. Cells were incubated on glass slides coated with 15 μg/ml anti-IgM for 15 min. (**H**) Representative confocal microscopy images of IS formation in naive B cells. F-actin was stained with Phalloidin (red), MTOC with anti-pericentrin (PCNT) (white), and lysosomes with anti-Lamp1 (cyan). Scale bar: 5 μm. (**I**) Analysis of F-actin MFI of HD (grey) and LRBA-P4 and P5 (red) of cells that formed an IS. Each dot represents one cell (HD: *n* = 142 from *n* = 3 different HD, P4: *n* = 21 and P5: *n* = 46), mean ± SD shown by the black line and error bars. (**J**) Analysis of lysosome distribution during IS formation. Percentage of cells with compact (dark-blue) or dispersed (light-blue) lysosome distribution. Bars represent mean ± SD for HD (*n* = 3 different HD) and mean for LRBA-P4 and P5. AU Arbitrary Units. Statistical analysis was performed using one-way (**D**, **F**, **G**, **I**) or two-way ANOVA with Tukey’s multiple comparisons test (**B**, **C**), *P < 0.05 (**C**: *P* = 0.0131, **I**: *P* = 0.0461), **P < 0.01 (**C**: *P* = 0.0014), ***P < 0.001 (**B**: *P* = 0.0004), ****P < 0.0001, ns: not significant.

Following rapid F-actin polymerization and BCR downstream signalling during the B cell spreading phase, B cells contract and form the mature immune synapse (IS) (Fleire et al, 2006; Harwood and Batista, 2010). Within the IS, antigen-bound BCRs cluster and form the central supramolecular activation complex (cSMAC), while the microtubule organizing center (MTOC) and lysosomes polarize to the contact site with the APC. This is known as the mature IS, which is further stabilized by integrins in the peripheral SMAC (pSMAC) (Carrasco et al, 2004; Depoil et al, 2008; Stinchcombe et al, 2006). Given the observed defects in actin remodelling and B cell signalling in LRBA-deficient cells, we tested IS formation in WT and LRBA-KO Ramos B cells stimulated on anti-IgM-coated slides mimicking immobilized antigen presentation (Riobó and Yuseff, 2024). Mature IS formation was defined by the presence of F-actin (phalloidin), the centrosome (pericentrin, PCNT) and lysosomes (Lamp1). Only cells exhibiting this triple signal were classified as IS-forming cells. Using this criterion, we observed a reduced frequency of IS formation in LRBA-KO Ramos B cells compared to WT cells (Figs. 4A,B and EV4A,B). Additionally, LRBA-KO Ramos B cells showed impaired MTOC positioning, as determined by the reduced ratio of the distance of the MTOC (PCNT+ signal) to the plasma membrane (visualized by the F-actin ring) (Figs. 4A,B and EV4A,B), and lysosomal distribution (Lamp1+ signal) despite comparable Lamp1 expression levels (Figs. 4C and EV4C). Reduced F-actin intensity was also observed in LRBA-KO Ramos B cells (Figs. 4A,B and EV4A,B), confirming our previous observations (Fig. 3A,B). Treatment of WT Ramos B cells with Blebbistatin recapitulated the abnormal IS phenotype observed in LRBA-KO cells (Fig. EV4A–C). Partial reconstitution of LRBA expression in LRBA-KO Ramos B cells using Myc-tagged LRBA mRNA was sufficient to significantly restore the formation of IS, including an increased F-actin intensity and MTOC and lysosome polarization, compared to LRBA-KO cells, however, partial LRBA reconstitution did not reach WT levels (Figs. 4A–C and EV4D–G). Similar IS abnormalities including reduced F-actin intensity and dispersed lysosomal positioning were observed in naive B cells from LRBA-deficient patients (Fig. EV4H–J). Moreover, LRBA-KO Ramos B cells stimulated with anti-IgM-coated beads, which mimic APCs or pathogens, exhibited a reduced F-actin enrichment at the bead-cell interface, accompanied by a failure of MTOC, lysosome and BCR polarization towards the contact site (Fig. 4D–G). Consistent with earlier findings by western blot (Fig. 3I), pMLC levels were reduced during IS formation in LRBA-KO Ramos B cells (Fig. 4H,I). Co-localization analysis showed that LRBA preferentially co-localized with pMLC than with total MYH9 at the IS, suggesting an association of LRBA with the active pool of NMIIA (Fig. 4J,K). Together, these results indicate that LRBA is required for proper cytoskeletal organization and efficient formation of the mature IS.

### Loss of LRBA results in reduced BCR clustering and internalization

**Figure 5:**
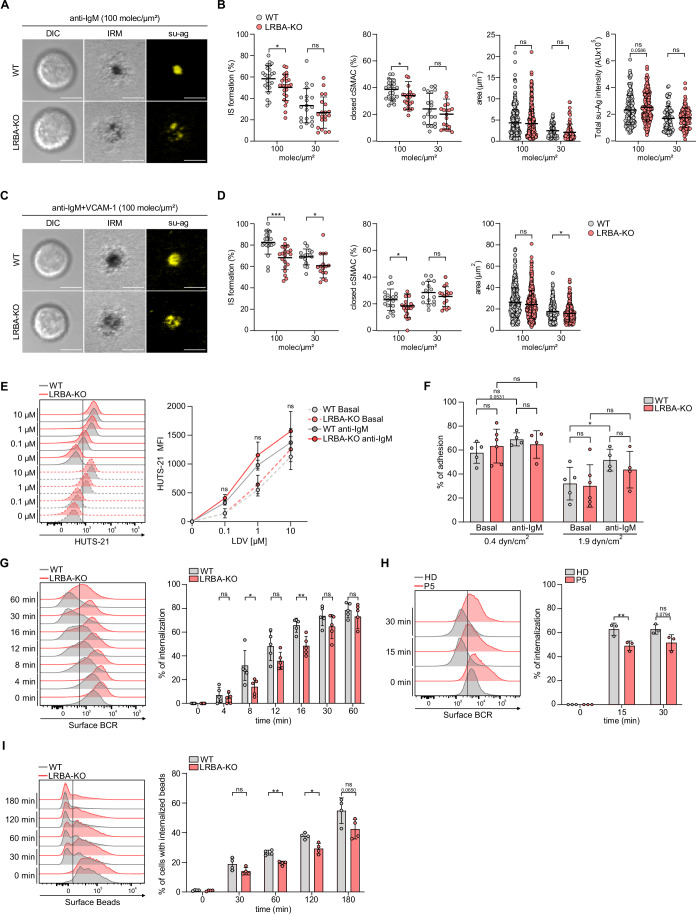
Loss of LRBA leads to reduced cSMAC formation and antigen internalization. (**A**–**D**) WT (grey) and LRBA-KO (red) Ramos B cells were allowed to settle on planar artificial lipid bilayers coated with different densities (30, 100 molecules/μm^2^) of surrogate antigen (su-ag) (**A**, **B**) anti-IgM or (**C**, **D**) anti-IgM in combination with VCAM-1, for 15 min and imaged by interference-reflection microscopy (IRM) and confocal fluorescence microscopy. (**A**, **C**) Representative differential interference contrast (DIC), IRM and su-ag images of WT and LRBA-KO Ramos B cells with 100 molecules/μm^2^ (**A**) anti-IgM or (**C**) anti-IgM+VCAM-1 density. Scale bar: 5 μm. (**B**, **D**) Quantification of frequency of contacts (IRM+ cells, left), percentage of closed cSMAC (su-Ag aggregate area, center-left), contact area (center-right) and total antigen intensity of anti-IgM (right) of WT (grey) and LRBA-KO (red) Ramos B cells. For IS and cSMAC frequency each dot represents one image (anti-IgM: IS: WT/KO-100: *n* = 24, WT/KO-30: *n* = 20; cSMAC: WT-100: *n* = 23, KO-100: *n* = 22, WT/KO-30: *n* = 20) (anti-IgM+VCAM-1: IS: WT/KO-100: *n* = 20, WT/KO-30: *n* = 16; cSMAC: WT/KO-100: *n* = 20, WT/KO-30: *n *= 16) from *n* = 3 biological replicates. For area and antigen intensity each dot represents one cell that formed an IRM and closed its cSMAC (anti-IgM: area: WT-100: *n* = 307, KO-100: *n* = 504, WT-30: *n* = 66, KO-30: *n* = 86; intensity: WT-100: *n* = 177, KO-100: *n* = 230, WT-30: *n* = 92, KO-30: *n* = 103) (anti-IgM+VCAM-1: area: WT-100: *n* = 399, KO-100: *n* = 478, WT-30: *n* = 235, KO-30: *n* = 262) analysed from *n* = 3 biological replicates, mean ± SD shown by the black line and error bars. (**E**) VLA-4 activation was determined by HUTS-21 antibody staining which binds to active VLA-4 of WT (grey) and LRBA-KO (red) Ramos B cells. Cells were incubated with HUTS-21 antibody for 30 min at 37 °C in the presence of the indicated concentrations of a VLA-4 ligand LDV peptide at basal level or in the presence of 5 μg/ml anti-IgM. Each dot represents the mean ± SD of *n* = 3 biological replicates. (**F**) Percentage of adhesion was evaluated in WT (grey) and LRBA-KO (red) Ramos B cells following perfusion at low (0.4 dyn/cm^2^) and then high (1.9 dyn/cm^2^) shear stress using slides coated with 0.25 µg/ml VCAM-1. Adhesion was measured under basal conditions or after stimulation with 5 μg/ml anti-IgM for 5 min. Each dot represents one biological replicate, while bars represent mean ± SD of *n* = 4-6 biological replicates. (**G**) WT (grey) and LRBA-KO (red) Ramos B cells and (**H**) naive B cells from HD (grey) and LRBA-P5 (red) were treated with 10 μg/ml Biotin-coupled anti-IgM and incubated at 37 °C for the indicated time points. The surface BCRs bound to anti-IgM were detected by Neutravidin-DyLight633 staining. Each dot represents one biological replicate, while bars represent (**G**) the mean ± SD percentage of internalized BCR from *n* = 5 biological replicates and (**H**) *n* = 3 biological replicates for LRBA-P5 and *n* = 3 biological replicates from *n* = 2 HD. (**I**) WT (grey) and LRBA-KO (red) Ramos B cells were treated with Biotin-coupled anti-IgM coated beads and incubated at 37 °C for the indicated time points. The surface BCRs bound to anti-IgM-coated beads were detected by Neutravidin-DyLight633 staining. Each dot represents one biological replicate, while bars represent the mean ± SD percentage of internalized beads from *n* = 3 biological replicates. AU Arbitrary Units. Statistical analysis of all experiments was performed using Welch’s *t* test (**B**, **D**, **F**–**I**) or two-way ANOVA with Tukey’s multiple comparisons test (**E**), *P < 0.05 (**B**, left: *P* = 0.0265, **B**, center-left: *P* = 0.0111, **D**, left: *P* = 0.0209, **D**, center: *P* = 0.0368, **D**, right: *P* = 0.0407, **F**: *P* = 0.0370, **G**: *P* = 0.0304, **I**: *P* = 0.0145), **P < 0.01 (**G**: *P* = 0.0051, **I**: *P* = 0.0014), ***P < 0.001 (**D**: *P* = 0.0002), ns: not significant. Source data are available online for this figure.

**Figure EV5:**
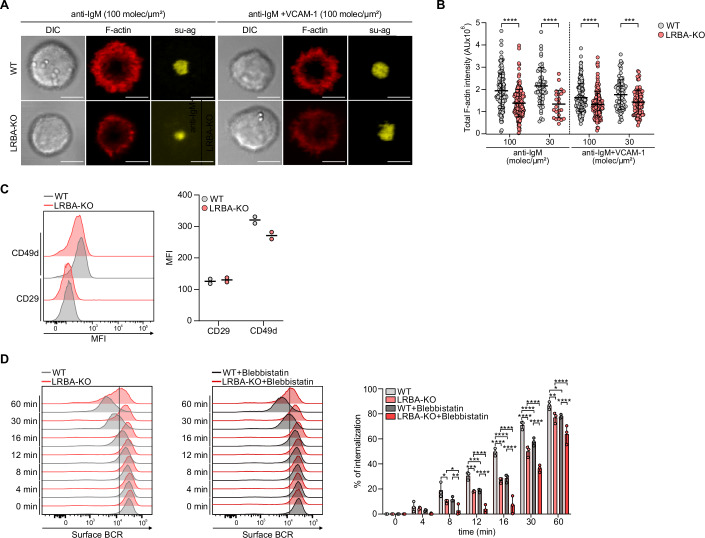
LRBA-deficient B cells show reduced F-actin intensity during immune synapse formation. (**A**, **B**) WT and LRBA-KO Ramos B cells were allowed to settle on planar artificial lipid bilayers coated with different densities (30, 100 molecules/μm^2^) of surrogate antigen (su-ag) anti-IgM or anti-IgM and VCAM-1 for 30 min, fixed and analyzed for total F-actin intensity using Phalloidin staining. (**A**) Representative differential interference contrast (DIC), F-actin and su-ag images of WT and LRBA-KO Ramos B cells with 100 molecules/μm^2^ density. Scale bar: 5 μm. (**B**) Analysis of total F-actin intensity of WT (grey) and LRBA-KO (red) Ramos B cells. Each dot represents one cell (anti-IgM: WT-100: *n* = 138, KO-100: *n* = 183, WT-30: *n* = 61, KO-30: *n* = 23) (anti-IgM+VCAM-1: WT-100: *n* = 185, KO-100: *n* = 175, WT-30: *n* = 84, KO-30: *n* = 96) from *n* = 3 biological replicates, mean ± SD shown by the black line and error bars. (**C**) Expression of CD29 (Integrin β-1) and CD49d (Integrin α-4) (VLA-4) of WT (grey) and LRBA-KO (red) Ramos B cells. Each dot represents the mean of *n* = 2 technical replicates, while the mean of *n* = 2 biological replicates is shown by the black line. (**D**) WT (grey) and LRBA-KO (red) Ramos B cells were left untreated (light color) or treated with 50 μM Blebbistatin (dark color) and incubated with 10 μg/ml Biotin-coupled anti-IgM for 30 min at 4 °C. Cells were then incubated at 37 °C for the indicated time points and surface BCRs bound to anti-IgM were detected by Neutravidin-DyLight633 staining. Each dot represents one biological replicate, while bars represent mean ± SD of *n* = 3 biological replicates. AU Arbitrary Units. Statistical analysis was performed using Welch’s *t* test (**B**) or two-way ANOVA with Tukey’s multiple comparisons test (**D**), *P < 0.05 (**D**: WT vs KO-8 min: *P* = 0.0103, **D**: KO vs. KO Blebbistatin-8 min: *P* = 0.0482, **D**: WT vs. WT Blebbistatin-60 min: *P* = 0.0122), **P < 0.01 (**D**: WT Blebbistatin vs KO Blebbistatin-8 min: *P* = 0.083, **D**: WT vs. KO-60 min: *P* = 0.0046), ***P < 0.001 (**B**: *P* = 0.0003, **D**: WT vs. KO-12 min: *P* = 0.0002, **D**: WT vs. WT Blebbistatin-12 min: *P* = 0.0004), ****P < 0.0001, ns: not significant.

To gain deeper insight into the abnormal IS formation observed in LRBA-KO cells, we evaluated cSMAC formation, reflecting BCR clustering, using artificial planar lipid bilayers tethered with anti-IgM as a surrogate antigen (su-ag) mimicking the surface of an APC (Carrasco et al, 2004). The frequency of B cells that formed an IS was analyzed by interference reflection microscopy (IRM) and the formation of the cSMAC and BCR clustering was determined by the accumulated su-ag during IS formation. Following incubation of WT and LRBA-KO Ramos B cells with the su-ag tethered lipid bilayer, analysis of the cell contact with 100 molecules/µm^2^ su-ag revealed significantly reduced formation of IS and confirmed reduced F-actin accumulation in the absence of LRBA compared to WT cells (Figs. 5A,B and EV5A,B). Additionally, LRBA-KO cells showed a reduced formation of closed cSMACs at high su-ag density (estimated by both fluorescence and dark dot-like IRM spots), indicating a compromised BCR clustering (Fig. 5A,B). In contrast, the contact area and the total amount of su-ag accumulated at the IS were comparable between WT and LRBA-KO Ramos B cells, suggesting that once LRBA-KO cells establish contact with the antigen, the interaction is stable (Fig. 5A,B). Given the differences in the cSMAC formation, we investigated pSMAC formation by tethering both VCAM-1 and anti-IgM (su-ag) to the lipid bilayers. Loss of LRBA resulted in reduced IS formation at 30 and 100 molecules/µm^2^, while only small differences in the contact area size at 30 molecules/µm^2^ were observed (Fig. 5C,D). Moreover, clustering of the su-ag at 100 molecules/µm^2^ was significantly diminished in LRBA-KO Ramos B cells (Fig. 5C,D). The small differences in the contact area size (estimated by IRM) for WT and LRBA-KO Ramos B cells indicated that VLA-4/VCAM-1 adhesion, and thus pSMAC assembly, were only mildly impaired. The levels of CD49d (integrin α-4) and CD29 (integrin β-1) were comparable between WT and LRBA-KO Ramos B cells (Fig. EV5C). Moreover, VLA-4 activation, determined by measuring its conformational state using the HUTS-21 antibody, and its ligand binding capacity to the specific VLA-4 ligand, the LDV peptide, were similar in WT and LRBA-KO Ramos B cells (Fig. 5E). In addition, adhesion to VCAM-1 at low 0.4 dyn/cm^2^ and high 1.9 dyn/cm^2^ shear stress were similar in WT and LRBA-KO cells (Fig. 5F), indicating that loss of LRBA does not affect VLA-4 expression, activity or adhesion under shear stress.

Finally, once the mature IS has formed, the BCR and its antigen are internalized for subsequent antigen processing (Batista et al, 2001). To determine if loss of LRBA has an impact on BCR and antigen internalization, WT and LRBA-KO Ramos B cells as well as naive B cells from a HD and an LRBA-deficient patient were incubated with biotin anti-IgM and BCR internalization was quantified by Neutravidin staining. We observed reduced internalization of the anti-IgM coupled to BCR in LRBA-KO Ramos B cells and in LRBA-deficient patient cells compared to WT cells (Fig. 5G,H). A similar defect in BCR internalization was observed in WT Ramos B cells after inhibiting NMIIA with Blebbistatin (Fig. EV5D). In addition, internalization of anti-IgM coated beads was reduced in LRBA-KO Ramos B cells (Fig. 5I). These data suggest that LRBA deficiency disrupts BCR clustering and cSMAC organization and compromises BCR and antigen internalization.

## Discussion

This study reveals a novel role of LRBA in regulating cytoskeleton dynamics during B cell activation and identifies non-muscle myosin IIA (NMIIA) as a new interaction partner of LRBA in B cells. Loss of LRBA leads to abnormal cell migration, reduced downstream BCR signalling and diminished BCR polarization. Additionally, LRBA deficiency disrupts proper IS architecture, including cSMAC formation, MTOC translocation and lysosome polarization, and reduces internalization of the BCR and its bound antigen. These defects are likely driven by reduced F-actin polymerization and impaired NMIIA activation in the absence of LRBA. Collectively, these abnormalities may contribute to the defective humoral immune response observed in LRBA-deficient patients.

Our data indicates that LRBA interacts with MYH9 *via* its DUF1088 domain. To date, this domain has primarily been associated with nuclear translocation in Neurobeachin (NBEA), the only other BEACH family member that shares the DUF1088 with LRBA (Cullinane et al, 2013; Tuand et al, 2016). DUF domains are implicated in mediating protein-protein interactions and have recently been linked to cytoskeletal regulation (Bateman et al, 2010; Lubaba et al, 2025; Srivastava et al, 2024). Our mapping of the LRBA-MYH9 interaction to the DUF1088 further extends the emerging connection between DUF domains and cytoskeletal functions.

NMIIA plays a key role in regulating cell migration as NMIIA-deficient murine B cells demonstrated impaired chemotactic migration towards CXCL12 in transwell assays, while NMIIA-KO tumorigenic fibroblast-like cells exhibited reduced 3D invasion capacity through matrigel despite displaying enhanced directional migration with increased directionality in 2D migration assays (Cowan et al, 2022; Hoogeboom et al, 2018). Similarly, LRBA-deficient B cells exhibited distinct migration behaviours depending on the analysed environment. In confined microchannels allowing spontaneous migration without physical obstacles, LRBA-deficient cells migrated faster than HD cells, accompanied by front-enriched F-actin and loss of rear-polarized pMLC, indicating a disrupted front-rear organization. Notably, in HD LCL cells, LRBA co-localized with pMLC at the cell rear, suggesting a direct spatial association between LRBA and active NMIIA at this site. In contrast, fewer LRBA-deficient cells were able to pass through constrictions in microchannels or migrate towards CXCL12 in transwell assays despite normal CXCR4/CXCR7 receptor expression. These findings suggest that defective actomyosin contractility in the absence of LRBA limits cell and nuclear deformation, which is required for migration through restrictive environments (Calero-Cuenca et al, 2018; McGregor et al, 2016). Similar migration dichotomy has been observed in NMIIA-KO cells (Cowan et al, 2022; Hoogeboom et al, 2018), and in HD LCL cells treated with Blebbistatin, which supports the idea that NMIIA contractile forces are crucial for nuclear deformation, enabling cellular passage through restrictive environments. Consistent with our observations, previous reports of reduced transwell migration of LRBA-deficient LCL cells towards Sphingosine-1-phosphate (S1P), despite normal S1P internalization, likely reflect impaired cytoskeleton remodeling and reduced capacity to pass restrictive barriers during migration, rather than defective S1P receptor downstream signaling (Sic et al, 2014).

LRBA-KO Ramos B cells also showed diminished F-actin polymerization, a process essential for efficient B cell activation, as it drives the formation of BCR microclusters, supports immune synapse architecture, and enables the mechanical forces required for antigen extraction (Li et al, 2018). Of note, the reduction in F-actin intensity observed already at baseline suggests that LRBA contributes to constitutive actin homeostasis beyond stimulus-induced polymerization. Indeed, we observed reduced BCR downstream signaling and calcium release in the absence of LRBA upon BCR engagement with anti-IgM. These early signalling events are required for NF-κB activation (Petro and Khan, 2001; Wen et al, 2019). Consistently, a previous study in *Lrba*^*−/−*^ mice described poor NF-κB response upon anti-IgM stimulation in vitro (Moreno-Corona et al, 2020), indicating that LRBA-deficient B cells fail to respond to BCR stimulation. This was further supported by the delayed BCR polarization after soluble anti-IgM stimulation, which is required for efficient B cell activation (Arbogast et al, 2019). Reduced BCR polarization and cSMAC formation was also observed when analysing IS formation in LRBA-deficient cells, further accompanied by diminished MTOC translocation and lysosome polarization. In addition, we observed a slight reduction in pSMAC formation in LRBA-KO Ramos B cells while VLA-4 activity and adhesion under shear stress were not significantly affected. This suggests that in the absence of LRBA, residual BCR signaling is sufficient to support VLA-4 inside-out activation, while impaired actomyosin contractility and reduced BCR clustering prevent proper spatial organization of activated integrins at the IS (Carrasco et al, 2004; Wang et al, 2022). Reduced BCR clustering and polarization have also been reported to limit antigen centralization and lysosomal secretion at the synapse, impairing BCR-mediated antigen uptake (Yuseff et al, 2013). While we did not measure antigen extraction from an APC, we did observe reduced internalization of soluble (anti-IgM) and particulate antigen (anti-IgM-coated beads) in the absence of LRBA. Notably, similar defects in IS formation and BCR internalization have been reported in other primary immunodeficiencies, including WAS, DOCK8 and BTK deficiency, where they contribute to impaired B cell activation (Bai et al, 2016; Gu et al, 2024; Roman-Garcia et al, 2018).

Collectively, the observed IS and BCR internalization defects are likely driven by impaired NMIIA activation, as evidenced by the reduced pMLC levels in LRBA-KO Ramos B cells. This is further supported by the preferential co-localization of LRBA with the active form of NMIIA (pMLC) rather than total MYH9 at the IS. It has been previously reported that NMIIA-driven actomyosin arcs drive centripetal movement of BCR microclusters, enabling cSMAC formation, and that inhibition of NMIIA prevents IS formation (Wang et al, 2022). Consistent with this, inhibition of NMIIA by Blebbistatin treatment of WT Ramos B cells recapitulated the IS formation defects observed in LRBA-KO cells. Similarly, NMIIA-mediated contractile forces are required for BCR-mediated antigen internalization (Hoogeboom et al, 2018; Seeley-Fallen et al, 2022), and reduced antigen internalization was equally observed upon Blebbistatin treatment of WT Ramos B cells. Notably, Blebbistatin treatment did not recapitulate the BCR signaling defect observed in LRBA-KO cells, suggesting that impaired NMIIA contractility is not the primary driver of reduced BCR signaling in LRBA deficiency. Instead, the BCR signaling defect is likely attributable to the reduced F-actin polymerization observed in LRBA-KO cells, consistent with the established role of Arp2/3-dependent actin dynamics in BCR signaling initiation (Bolger-Munro et al, 2019; Li et al, 2018), while the IS maturation and BCR internalization defects are likely attributable to reduced NMIIA contractility. The precise relationship between LRBA, NMIIA activation and actin dynamics during BCR engagement, however, remains to be fully elucidated.

Interestingly, despite these functional abnormalities combined with the well-described reduction of memory B cells and plasma cells in combination with hypogammaglobulinemia, histological analysis of one LRBA-deficient patient showed preserved germinal center (GC) structures (Al Sukaiti et al, 2017). Similarly, *Lrba*^*−/−*^ mice display mostly normal splenic GC formation, isotype switching and affinity maturation following immunization (Burnett et al, 2017; Gámez-Díaz et al, 2017), although recent studies showed reduced GC size in Peyer’s Patches of *Lrba*^*−/−*^ compared to wt controls (Flores-Hermenegildo et al, 2024). However, systematic characterizations of GC architecture and function remain limited, and murine models do not recapitulate the human LRBA-deficient disease phenotype (Burnett et al, 2017; Gámez-Díaz et al, 2017). The functional defects we observe would be expected to impact B cell activation critical for GC responses. Nevertheless, the observed residual BCR signaling and cSMAC formation may be sufficient for initial GC formation, but may affect the GC output, including class-switch recombination (CSR), memory cell and plasma cell differentiation. Further studies including analysis of additional patient biopsies, will be required to better define GC architecture and function in LRBA deficiency. Importantly, B cell activation not only depends on BCR signals but also on co-stimulatory signals such as CD40L and IL-4 signals (Chakma and Good-Jacobson, 2023; Elgueta et al, 2009). Stimulation through the BCR and CD40, in combination with IL-4, induces expression of activation-induced cytidine deaminase (AID), which is required for the initiation of CSR (Stavnezer et al, 2008). LRBA has previously been described to act as a cAMP-dependent kinase (PKA) anchoring protein (AKAP) by interacting with PKA-RII subunits, which phosphorylates PKA substrates such as AID, allowing them to enter the nucleus (Moreno-Corona et al, 2020). Moreover, we have previously reported higher apoptosis of LRBA-deficient B cells, likely due to defective autophagy (Lopez-Herrera et al, 2012). Autophagy has been demonstrated essential for plasma cell survival and antibody production (Pengo et al, 2013; Pengo and Cenci, 2013). We therefore suggest that a combination of reduced B cell activation due to cytoskeleton abnormalities, a decreased AID phosphorylation affecting CSR, and a defective autophagy impairing plasma cell maintenance may account for the reduced numbers of memory B cells, reduced CSR, and hypogammaglobulinemia observed in patients with LRBA deficiency. Interestingly, WASp has been reported to play a similar dual role linking autophagy and cytoskeleton remodeling (Rivers et al, 2020), which may help explain the reduced numbers of memory B cells and plasma cells observed in WAS patients (Castiello et al, 2014). Similarly to LRBA deficiency, DOCK8-deficient patients present with reduced humoral immune response, susceptibility to infections, and autoimmunity (Biggs et al, 2017). Although DOCK8 has not been directly linked to autophagy, a recent DOCK8 interactome study in IgA+ B cells identified potential interaction partners involved in autophagy regulation (Zhang et al, 2024), suggesting that the role of DOCK8 extends beyond actin cytoskeleton remodeling.

Collectively, LRBA demonstrates functional flexibility as a scaffolding protein, orchestrating diverse cellular processes likely through its multiple interaction domains (Ezen et al, 2024). Beyond its well-characterized role in CTLA-4 recycling, LRBA functions as an AKAP, regulates autophagy and actin cytoskeleton dynamics, and is essential for vesicle trafficking in different cell types (Lo et al, 2015; Moreno-Corona et al, 2020; Szentgyörgyi et al, 2024; Yanagawa et al, 2023; Sindram et al, 2025). While our study identifies NMIIA as a novel interaction partner of LRBA in B cells and demonstrates that LRBA is essential for cytoskeleton organization, we cannot exclude that additional LRBA-interacting proteins, including both known and yet to be identified, also contribute to the B cell defects observed in LRBA deficiency. Hence, it would be interesting to investigate how and where the distinct LRBA protein complexes underlying such diverse cellular functions are assembled and regulated in a cell-type-specific manner. As we used immortalized LCLs for our MS analysis, which may not express key factors required for CSR and GC-specific functions, it would be particularly beneficial to analyze LRBA complexomes in different B cell subsets to dissect the role of LRBA during distinct stages of B cell differentiation. This could help to better explain the complex picture of defective humoral immunity in LRBA deficiency.

In conclusion, we identified that LRBA is essential for several cytoskeleton-dependent immune functions in B cells. Loss of LRBA resulted in abnormal cell migration, diminished B cell activation, impaired IS formation, and reduced antigen internalization. We suggest that this novel role of LRBA contributes to the defective humoral immune response observed in LRBA-deficient patients.

## Methods

**Table:** Reagents and tools table

Reagent/resource	Reference or source	Identifier or catalog number
**Experimental models**		
LCL (Lymphoblastoid cell line (*H. sapiens*)	Generated in our laboratory using healthy donor cells and Epstein–Barr virus supernatant	N/A
Naïve primary B cells (*H. sapiens*)	From healthy donors and LRBA-deficient patients in Freiburg	N/A
Primary B cells (*H. sapiens*)	From healthy donors in Freiburg	N/A
Ramos B cells	ATCC	CRL-1596
**Recombinant DNA**		
EGFP-Vimentin-7	Addgene	56439
mCherry-ITSN2	Addgene	129615
pCMV6-AC-Myc-DDK-LRBA	Origene	RC17204
pCMV-mCherry-MHC-IIA	Addgene	35687
pcDNA3-FLAG-MYO18A	Signaling Factory & Robotics Facility University of Freiburg	N/A
pcDNA3-HA-TPM3	Signaling Factory & Robotics Facility University of Freiburg	N/A
pcDNA-myc-FLNa WT	Addgene	8982
pClneo FLAG LRBA fragments	Dr. Bernice Lo, National Institute of Health, USA	N/A
**Antibodies**		
AlexaFluor647 anti-CD107a (Lamp1)	BD Biosciences	562622
AlexaFluor555 anti-mouse	Cell Signaling	4409
AlexaFluor647 anti-mouse	Cell Signaling	4410
AlexaFluor555 anti-rabbit	Cell Signaling	4413
AlexaFluor568 anti-mouse	Life Technologies	A10037
AlexaFluor647 anti-rabbit	Cell Signaling	4414
AlexaFluor647 anti-rabbit	Life Technologies	A21442
AlexaFluor647 Fab goat anti-IgM, Fc5µ	Jackson Immunoresearch	109-607-043
AlexaFluor647 F(ab’)_2_ goat anti-IgG+IgM (H + L)	Jackson Immunoresearch	109-606-127
Goat anti-human IgM Biotin	Thermo Fisher	31778
Goat F(ab’)_2_ anti-human IgM	Jackson ImmunoResearch	169-007-043
HRP-linked anti-mouse	Santa Cruz	sc-516102
HRP-linked anti-rabbit	Invitrogen	31470
HRP-linked anti-Tubulin	Prointech	HRP-66031
Mouse anti-FLAG	Sigma-Aldrich	SAB4301135
Mouse anti-GFP	Invitrogen	A-11120
Mouse anti-Ig light chain λ	Biolegend	316619
Mouse anti-ITSN2	Abnova	H00050618-A01
Mouse anti-MYH9	Sigma-Aldrich	WH0004627M3
Mouse anti-MYH9	Proteintech	60233-1-Ig
Mouse anti-Pericentrin	Novus	NB100-61071
Mouse anti-pMLC (Ser19)	Cell Signaling	3675
PE anti-CD20 HUTS-21	BD Biosciences	556049
PE anti-CXCR4	BD Biosciences	555974
PE anti-CXCR7	Biolegend	391403
PE anti-pAkt (Ser473)	Biolegend	606553
PE anti-pPLCγ2	BD Biosciences	558490
PE anti-rabbit	BD Biosciences	558416
PerCP-Cy5.5 anti-IgM	Biolegend	314512
PLA probe anti-mouse minus	Sigma-Aldrich	DU92004
PLA probe anti-goat minus	Sigma-Aldrich	DU92006
PLA probe anti-rabbit plus	Sigma-Aldrich	DUO92101
Rabbit anti-GAPDH	Cell Signaling	2118
Rabbit anti-HA	Cell Signaling	3724
Rabbit anti-IgM	Biolegend	SA5-10316
Rabbit anti-LRBA	Sigma-Aldrich	HPA023597
Rabbit anti-Myc	Cell Signaling	2278S
Rabbit anti-MYO18A	Bethyl Laboratories	A301-597A-T
Rabbit anti-pMLC (Thr18/Ser19)	Cell Signaling	3674
Rabbit anti-pSyk	Cell Signaling	2710S
**Oligonucleotides and other sequence-based reagents**		
gRNA for CrispR	IDT	N.A.
shControl	Thermo Fisher	
shLRBA	Thermo Fisher	
**Chemicals, enzymes and other reagents**		
1,2-dioleoyl-PC (DOPC)	Santa Cruz	sc-208733
1 µm Fluoresbrite YG Microspheres	Polysciences	17154-5
3 µm polystyrene carboxylate microspheres	Polysciences	09850
8-chamber 1.5H coverslips	Ibidi	80806
12–230 kDa Separation Module Wes Simple Western	Bio-Techne	SM-W001
AlexaFluor647-Streptavidin	Molecular probes	10349222
Ammonium Chloride (NH_4_Cl)	Sigma-Aldrich	213330
Anti-Mouse Detection Module Wes Simple Western	Bio-Techne	DM-002
Anti-Rabbit Detection Module Wes Simple Western	Bio-Techne	DM-001
BD Cytofix/Cytoperm solution^TM^	BD Biosciences	554714
BD Perm/Wash Buffer^TM^	BD Biosciences	554723
BSA Albumin Bovine	Serva	11903.03
CD40L, soluble, human recombinant	Enzo	ALX-522-015
DAPI	Sigma-Aldrich	D9542
Dimethyl Sulfoxide (DMSO)	Merck	67-68-5
DPBS	Sigma-Aldrich	D8537
Duolink In Situ Mounting Medium with DAPI	Merck	DEU82040
DyLight633 Neutravidin	Invitrogen	22844
Dynabeads protein G	Thermo Fisher Scientific	10004D
EasySep^TM^ Human B Cell Isolation Kit	StemCell	17954
EasySep^TM^ Human Naïve B Cell Isolation Kit	StemCell	17254
EDTA-free protease inhibitor cocktail	Roche	5,893E + 09
Ethanol	VWR	20.821.310
Ethylenediaminetetraacetic acid solution (EDTA)	Sigma-Aldrich	E8008
FCS2 chamber	Bioptechs	060319-2-03
Fetal calf serum (FCS)	Capricorn	10-FBS-11F
FITC-Phalloidin	Merck	P5282
HBSS with calcium and magnesium	Gibco	14025092
HiScribe® T7 ARCA mRNA Kit	New England Biolabs	E2065S
Human IL-21	Peprotech	200-21
Indo-1	ATT Bioquest	21036
Ionomycin	Cell Signaling	9995S
Lipofectamine™ 2000	Invitrogen	11668-027
Lysotracker green DND-26	Invitrogen	L7526
μ-slide VI 0.4 channels	Ibidi	80606
Microchannels for migration	Custom made by Juan Eduardo Montero-Hernández and Fernando E. Sepulveda (Institut Imagine, Paris, France)	Vargas et al, 2014
Milk powder	Roth	T145.1/.3
Multispot microscope slides	Hendley-Essex	PH055
N-2-[4-[[[(2-methylphenyl) amino]carbonyl]amino]phenyl]acetyl-Leu	R&D Systems	7020
NHS-LC-LC-biotin Pierce	Thermo Scientific	PG82075
NP-40	Calbiochem	492018
Opti-MEM™ - Reduced Serum Medium	Thermo Fisher	31985070
Pancoll human	Pan Biotech	P04-601000
Paraformaldehyde	Sigma-Aldrich	158127
Penicillin-Streptomycin (10 000U/ml)	Thermo Fisher	15140122
Perm/Wash BufferTM	BD Biosciences	554723
Phospho Safe Buffer	Merck	TB402
Pierce^TM^ BCA Protein Assay Kit	Thermo Fisher	23225
Pluronic Acid	ATT Bioquest	20052
PmeI	New England Biolabs	R0560S
Poly-L-Lysine	Sigma-Aldrich	P4832
PolyLink Protein Coupling Kit	Polysciences	09850
Precision Count Beads^TM^	Biolegend	424902
Prolong Gold Antifade mounting medium	Thermo Fisher	P36930
PVDF membrane	Bio-Rad Laboratories GmbH	1620177
QIAquick PCR Purification Kit	Qiagen	28104
Recombinant human CXCL12	Peprotech	300-28A-10UG
Recombinant human VCAM-1-FC	R&D Systems	809-VR-050
RPMI 1640 + L-Glutamine	Sigma-Aldrich	21875091
(S)-4’-nitro-Blebbistatin	Cay24171-1	Biomol
Saccharose	Sigma-Aldrich	S0389
Saponin	Sigma-Aldrich	47036
SG Cell Line 4D-Nucleofector® X Kit S	Lonza	V4XC-3032
Signal Fire/ Plus chemiluminescent substrates	Cell Signaling	12630S
SILAC RPMI Kit	Silantes	284986434
Sodium chloride (NaCl)	Roth	9265.1
Sodium deoxycholate	Sigma-Aldrich	89904
Sodium dodecyl sulfate (SDS)	Sigma-Aldrich	436143
Sodium pyruvate	Sigma-Aldrich	P5280
SPY-555 FastAct	Spirochrome	SC205
SPY-650 tubulin	Spirochrome	SC503
Transwell chambers 5 µm	Corning	3421
Trichloroacetic acid (TCA)	Sigma-Aldrich	8.223.420.250
Tris	Applichem GmbH	A1379
TRITC-Phalloidin	Molecular Probes	R415
Triton X-100	Sigma-Aldrich	93443
Tween 20	Sigma-Aldrich	P9416
Violet Celltracer	Molecular Probes	C34557
**Software**		
Affinity designer 2	Serif	Version 2.6.5
CellProfiler		Version 4.2.8
Duolink Image Tool	Sigma-Aldrich	
FlowJo	Treestar Inc	Version 10.10.0
Gene Ontology		
GraphPad Prism		Version 10.0
Fiji		Version 2.9.0
Icy Software		Version 3
Image-Pro	Media Cybernetics	Version 11.0
Imaris	Oxford Instrument	Version 7.3.1
Imaris	Oxford Instrument	Version 10
MATLAB	MathWorks	
MaxQuant		Version 2.6.5.0
UniProt		
Zen Black	Zeiss	Version 3.10
Zen Blue	Zeiss	Version 3.11
**Other**		
FACSCanto II	BD Biosciences	
Fusion SL device	Peqlab	
4D-Nucleofector™	Lonza	AAF-1003X
LSRFortessa	BD Biosciences	
LTQ Orbitrap XL Mass Spectrometer	Thermo Scientific	IQLAAEGAAVFACZMAIK
Olympus IX83	Olympus	
Olympus Ixplore SPIN	Olympus	
WES ProteinSimple	Bio-Techne	004-600
Zeiss Axio Observer Z1 microscope	Zeiss	
Zeiss Axiovert LSM 510 META	Zeiss	
Zeiss LSM 710	Zeiss	
Zeiss LSM 880	Zeiss	

### Study protocol

Collection of peripheral blood mononuclear cells (PBMCs) from healthy donors (HD) and LRBA-deficient patients for generating LCL cell lines and for measuring IS formation and BCR internalization was approved by the Ethics Committee of the University of Freiburg, Germany, vote n° 290/13. Patients have signed an informed consent, and experiments were performed to the principles set out in the WMA Declaration of Helsinki and the Department of Health and Human Services Belmont report.

### PBMCs and B cell isolation

PBMCs were extracted from EDTA blood of HDs and five LRBA-deficient patients (P1: c.2004+2 T > C, P2: p.S2714HfsTer26, P3: I2657S, P4: p.S2457; p.Q1715X, and P5: Q1010X) by density gradient centrifugation using Lymphoprep^TM^ density medium (Gibco). PBMCs were used for the isolation of naive B cells (CD3-CD19 + CD27-) or total B cells (CD3-CD19 + ) using negative selection with EasySep^TM^ Human Naive B Cell Isolation Kit (StemCell) or EasySep^TM^ Human B Cell Isolation Kit (StemCell). Isolated naive B cells were used for the generation of LCL cells, immune synapse formation and BCR internalization. For co-IP, primary B cells were stimulated with 250 ng/ml CD40L (Enzo) and 10 µg/ml IL-21 (Peprotech) for 24 h before cell lysis.

### Human lymphoblastoid B cell line generation

LCL cells were generated from naive B cells of HDs and LRBA-deficient patients P1, P2 and P3 by incubation with EBV-containing media for four days. Afterwards, cells were cultured in RPMI + 10% FCS + 1% Pen/Strep and tested biweekly for Mycoplasma contamination. LCL cells express LRBA as shown by western blot (Fig. EV1A).

### Generation of a LRBA-KO Ramos cell line by CRISPR-Cas9

LRBA-KO Ramos cells were generated using the Alt-R-CRISPR-Cas9 system according to the manufacturer’s instructions (www.idtdna.com). The gRNA CCACCAACAGGTGATGACGG specific for exon 2 of human LRBA was introduced into the cells by electroporation together with the crRNA:tracrRNA complex and Cas9 protein. After 48 h of incubation, cells were single-cell sorted by flow cytometry. LRBA-KO clones were validated by western blot for the absence of LRBA protein and by Sanger sequencing for mutation identification (Fig. EV2A,B). Cells were cultured in RPMI + 10% FCS + 1% Pen/Strep and tested biweekly for Mycoplasma contamination.

### SILAC culture and mass spectrometry analysis

For SILAC labeling, LCL cells from HD1 and LRBA-P1 were cultured in RPMI 1640 (Silantes) containing 10% dialyzed FCS, 200 mM Glutamine and 1% P/S, and one of the following combinations: 242.28 mg/ml Lys-^12^C_6_^14^N_2_ (Lys-0) and 253.68 mg/ml Arg-^12^C_6_^14^N_4_ (Arg-0), or 242.28 mg/ml Lys-^13^C_6_^15^N_2_ (Lys-8) and 253.68 mg/ml Arg-^13^C_6_^15^N_4_ (Arg-10) (Silantes) for ten days. Cells were collected and lysed with IP buffer (50 mM Tris-HCl pH 6.8, 150 mM NaCl, 0.2% NP-40, 1 mM EDTA), plus 1x complete EDTA-free protease inhibitor cocktail (Roche). Immunoprecipitations were performed using 1.2 mg of cell lysates and 15 μg of anti-LRBA (Sigma-Aldrich). Dynabeads protein G (Thermo Fisher Scientific) were added to the protein/antibody mix and incubated O/N at 4 °C. The following day, HD and LRBA-P1 samples were combined and heated in SDS-Page loading buffer containing 1 mM DTT for 5 min at 95 °C and alkylated with 5.5 mM iodoacetamide for 30 min at RT. Proteins were separated by SDS-PAGE and gel lanes were cut into 10 equal-sized slices. Proteins in the gel slices were digested with trypsin and the resulting peptide mixtures were processed on STAGE tips and analysed by LC-MS/MS as previously described (Xie et al, 2011), using a LTQ Orbitrap XL mass spectrometer coupled to an Agilent 1200 nanoflow-HPLC. MS raw data files were uploaded into MaxQuant for peptide and protein identification (Tyanova et al, 2014). A full-length UniProt human database containing common contaminants of in-gel digestions was used as a reference. Carbamidomethylcysteine was set as a fixed modification and protein amino-terminal acetylation and oxidation of methionine were set as variable modifications. Triple SILAC was chosen as quantitation mode. Gene ontology analysis was performed on the 33 significantly enriched proteins using the PANTHER overrepresentation test within Geneontology.org release 38.560 (Ashburner et al, 2000). Enrichment *p*-value-values were determined by Fisher’s exact test against the Homo sapiens reference list and adjusted by Benjamini-Hochberg false discovery rate (FDR) correction. The FDR-corrected *p*-values were visualized as -log_10_-transformed values.

### Proximity ligation assay (PLA)

In situ PLA was performed using Duolink kit (Sigma-Aldrich) according to the manufacturer’s protocol. LCL cells from a HD and LRBA-P1 were collected, washed with PBS and fixed with 4% PFA + 2% Saccarose for 15 min at 4 °C. After fixation, cells were placed on glass slides and permeabilized with 0.1% Triton X-100 for 10 min at 37 °C. Cells were blocked with Duolink Blocking Solution for 30 min at 37 °C, and incubated with primary antibodies anti-LRBA (Sigma-Aldrich) and anti-MYH9 (Sigma-Aldrich) for 1 h 30 min at 37 °C. Cells were washed twice and incubated with secondary antibodies that are conjugated with oligonucleotides, PLA probe anti-mouse MINUS or PLA probe anti-rabbit PLUS (Sigma-Aldrich), for 1 h at 37 °C. DNA oligonucleotide ligation and amplification with DNA polymerase were performed at 37 °C for 30 min and 1 h 40 min, respectively. Images were acquired using a 20x objective with a confocal microscope (LSM710, Zeiss) and signal quantification was performed using Duolink Image Tool (Sigma-Aldrich).

### Plasmids

Human LRBA full-length tagged to Myc-DDK was purchased from Origene (pCMV6-AC-Myc-DDK-LRBA). pCMV-mCherry-MHC-IIA, pcDNA3-myc-FLNa WT, EGFP-Vimentin-7 and mCherry-ITSN2 were purchased from Addgene. pcDNA3-HA-TPM3 and pcDNA3-Flag-MYO18A were generated by the Signaling Factory & Robotics facility at the University of Freiburg, Germany. Human LRBA was cloned in seven different fragments into pCIneo FLAG vectors (Promega). These plasmids were kindly provided by Dr. Bernice Lo from the National Institute of Health, Bethesda, USA (Lo et al, 2015).

### Transfections

HEK293T cells were co-transfected with 2 μg of Myc-LRBA plasmid and 2 μg of mCherry-MYH9, Myc-FLNA, Flag-MYO18A, mCherry-ITSN2, EGFP-Vimentin or HA-TPM3 or with 2 μg mCherry-MYH9 with seven different FLAG-tagged LRBA fragments using Lipofectamine 2000 (Invitrogen), according to the manufacturer’s protocol. Cells were harvested after 48 h and used for co-immunoprecipitation experiments.

### Co-immunoprecipitation (co-IP)

Cell pellets were lysed with IP buffer (50 mM Tris-HCl pH 6.8, 150 mM NaCl, 0.2% NP-40, 1 mM EDTA), plus 1× complete EDTA-free protease inhibitor cocktail (Roche). Immunoprecipitations were performed using 200 μg of cell lysates from HEK293T cells and 1 μg of anti-LRBA (Sigma-Aldrich) or 2 μg of anti-FLAG (Sigma-Aldrich). For total B cells, 3 μg of anti-LRBA (Sigma-Aldrich) was used. Dynabeads protein G (Thermo Fisher Scientific) were added to the protein/antibody mix and incubated O/N at 4 °C. Beads were washed with IP buffer and proteins were eluted with 2% SDS for 10 min at 50 °C. Eluted proteins were either separated by SDS-PAGE, immunoblotted and detected or analyzed using the WES™ system (Protein Simple) as described in immunoblotting.

### Immunoblotting

For pSyk analysis with Blebbistatin treatment, Ramos WT and LRBA-KO cells were left untreated or treated with 50 µM Blebbistatin for 30 min and left unstimulated or stimulated with 5 μg/ml anti-IgM (Thermo Fisher) for 1 min, 3 min and 5 min. Blebbistatin was present during anti-IgM stimulation. For pMLC analysis, Ramos WT and LRBA-KO cells were left unstimulated or stimulated with 5 μg/ml anti-IgM (Thermo Fisher) for 1 min, 3 min, 5 min, 15 min, 30 min and 60 min. For phosphoproteins cells were lysed with Phosho Safe Buffer (Merck) + 1× complete EDTA-free protease inhibitor cocktail (Roche) while Ramos B cells and LCL cells were lysed with RIPA buffer (50 mM Tris-HCl, 1% NP-40, 0.5% sodium deoxycholate, 100 mM NaCl, 1 mM EDTA, 0.1% SDS) + 1× complete EDTA-free protease inhibitor cocktail (Roche) on ice for 20 min. Lysates were centrifuged at 20,000×*g* for 10 min at 4 °C. Protein concentrations were determined with the Pierce™ BCA Protein Assay Kit (Thermo Fisher Scientific). Protein lysates were size-fractionated by SDS-PAGE and immunoblotted on a PVDF-membrane for 1 h 45 min at 45 V. After blocking with 5% milk in TBST (20 mM Tris, 150 mM NaCl, 0.1% Tween-20), the membranes were incubated O/N at 4 °C with the following antibodies: anti-LRBA (Sigma-Aldrich), anti-MYH9 (Sigma-Aldrich), anti-Myc (Cell Signaling), anti-HA (Cell Signaling), anti-GFP (Invitrogen), anti-ITSN2 (Abnova), anti-MYO18A (Bethyl Laboratories) and anti-pSyk (Cell Signaling). After O/N incubation with primary antibodies, membranes were incubated in HRP-coupled secondary antibodies, anti-mouse (Santa Cruz) or anti-rabbit (Cell Signaling) for 1 h at RT. After washing, membranes were developed using Signal Fire Plus chemiluminescent substrates (Cell Signaling) with the Fusion SL device (Peqlab). HRP-linked anti-Tubulin (Proteintech) was used as a loading control. Analysis of phospho-MLC and co-IP of LRBA fragments with MYH9 were performed using the WES™ system (Protein Simple) and the 12–230 kDa WES kit (Biotechne), according to manufacturer’s instruction. The primary antibodies used were anti-pMLC (Cell Signaling), anti-GAPDH (Cell Signaling), anti-Flag (Sigma) and anti-MYH9 (Proteintech). The secondary antibodies, anti-rabbit (Biotechne) and anti-mouse (Biotechne) from the WES™ detection kit were used according to manufacturer’s instructions. Data analysis for all blots was performed using Fiji software version 2.9.0.

### Nucleofection with Myc-LRBA mRNA

To generate a DNA template suitable for in vitro transcription (IVT) 10 μg pCMV6-AC-Myc-DDK-LRBA (RC17204) were linearized using PmeI (New England Biolabs). The linearized plasmid was purified using QIAquick PCR Purification Kit (Qiagen). IVT was performed using HiScribe® T7 ARCA mRNA Kit with tailing (New England Biolabs) following the manufacturer’s instructions with a prolonged transcription step up to 2 h. Following A-tailing and LiCl precipitation, mRNA was air-dried and dissolved in nuclease-free H_2_O. For nucleofection, 5 μg LRBA mRNA was transferred to 0.5 × 10^6^ LRBA-KO Ramos B cells by electroporation using the SG Cell Line 4D-Nucleofector® X Kit S (Lonza) on a Lonza 4D-Nucleofector™ with pulse code CA-137. Additionally, WT and LRBA-KO Ramos B cells were nucleofected without mRNA as a control (mock). After nucleofection, cells were seeded in 1 ml RPMI + 10% FCS + 1% P/S and incubated for 24 h. The following day, cells were analysed for Myc expression using flow cytometry, and for LRBA expression and immune synapse formation using immunofluorescence microscopy.

### Flow cytometry analysis of receptor expression or phosphoproteins

For IgM expression analysis, Ramos WT and LRBA-KO cells were stained with PerCp-Cy5.5 anti-IgM (clone MHM-88, BioLegend). For CXCR4 and CXCR7 expression, Ramos WT and LRBA-KO cells or LCL cells from a HD and LRBA-P2, respectively, were left unstimulated or stimulated with 100 ng/ml CXCL12 (Peprotech) for 30 min, 1 h, 2 h, 4 h and 6 h and stained with PE anti-CXCR4 (BD Biosciences) or PE anti-CXCR7 (Biolegend). For phosphoprotein expression, Ramos WT and LRBA-KO cells were left unstimulated or stimulated with 5 μg/ml anti-IgM (Thermo Fisher) for 1 min, 3 min and 5 min, fixed with BD Cytofix/Cytoperm buffer (BD Biosciences) for 20 min at 4 °C and permeabilized with BD Perm/Wash buffer (BD Biosciences) for 30 min at 4 °C. Cells were stained with PE anti-phospho-Syk (BD Biosciences), PE anti-phospho PLCγ2 (BD Biosciences) or PE anti-phospho AKT (Ser473) (Biolegend) for 1 h at 4 °C. For analysis of pPLCγ2 with Blebbistatin treatment, Ramos WT and LRBA-KO cells were additionally treated with 50 µM Blebbistatin 30 min prior to stimulation. Blebbistatin was present during anti-IgM stimulation. For intracellular Myc staining after nucleofection, cells were fixed and permeabilized as described before and stained with anti-Myc (Cell Signaling) for 1 h at 4 °C. Secondary antibody staining was performed using PE anti-rabbit (BD Biosciences) for 30 min at 4 °C. Cells were acquired on a LSRFortessa (BD Biosciences), and data analysis and calculation of the mean fluorescence intensity (MFI) were performed using FlowJo 10.10.0 software (Treestar Inc.).

### VLA-4 activity

Ramos WT and LRBA-KO cells were incubated with different concentrations (0 μM, 0.1 μM, 1 μM, 10 μM) of the VLA-4 specific ligand LDV-containing probe (N-2-[4-[[[(2-methylphenyl) amino]carbonyl]amino]phenyl]acetyl-Leu) (R&D Systems) in the presence of PE anti-CD29 HUTS-21 (BD Biosciences), which specifically recognizes the LDV-bound VLA-4 conformation, for 30 min at 37 °C. Subsequently, cells were acquired on a LSRFortessa (BD Bioscience) and data analysis and calculation of the MFI were performed using FlowJo 10.10.0 software (Treestar Inc.).

### Shear stress assay

μ-slide VI 0.4 channels (Ibidi) were coated with 0.25 µg/ml recombinant human VCAM-1 diluted in Hank’s balanced salt solution containing Ca^2+^ and Mg^2+^ (HBSS^++^, Gibco) O/N at 4 °C. The following day, channels were blocked with 5% FCS in HBS^++^ for 1 h at 37 °C and washed with HBSS^++^. Next, channels were connected to a syringe pump (Harvard Apparatus) fitted with a syringe containing 50 ml RPMI + 10% FCS to establish laminar flow. Ramos WT and LRBA-KO cells were left unstimulated or stimulated with 10 μg/ml anti-IgM (Thermo Fisher) for 10 min at 37 °C and then injected into the flow system. Cells were left to bind to VCAM-1 for 5 min and images were acquired. To measure binding to VCAM-1 under shear stress, flow at 0.4 dyn/cm^2^ and at 1.9 dyn/cm^2^ were applied for 5 min. Five images before and after 0.4 dyn/cm^2^ and 1.9 dyn/cm^2^ flow were acquired using a 10x objective with an inverted microscope (Olympus IX83), and cell count before and after flow was determined using Fiji software, version 2.9.0. The percentage of adhesion after flow was calculated: %adherent cells = (post-flow average cell count)/(pre-flow average cell count) × 100.

### Immune synapse analysis using coated glass slides

Multispot microscope slides (Hendley-Essex) were coated with 0.01% Poly-L-lysine for 1 h at 37 °C, washed and coated with 15 μg/ml anti-IgM (Thermo Fisher) for 2 h at 37 °C. Ramos WT, LRBA-KO cells, LRBA-KO cells nucleofected with Myc-LRBA RNA and naive B cells from HD and LRBA-P4 and P5 were seeded on the coated spots and slides were incubated for 15 min at 37 °C. Cells were fixed with 4% PFA for 15 min at RT and after washing with PBS incubated in quenching solution (50 mM NH_4_Cl in PBS) for 15 min at RT. Afterwards, cells were blocked with blocking solution (1% BSA + 0.2% Saponin in PBS) for 30 min at RT and stained with anti-Pericentrin (Novus) and AlexaFluor647 anti-CD107a (Lamp1) (BD Biosciences) or anti-LRBA (Sigma-Aldrich) for 1 h at RT. After washing with blocking solution, cells were stained with AlexaFluor555 anti-rabbit (Cell Signaling) and FITC-Phalloidin (Merck) for 45 min at RT. After washing, cells were stained with DAPI (Thermo Fisher) for 5 min at RT and mounted with DuoLink In Situ Mounting Medium (Merck). For analysis of IS formation with Blebbistatin treatment, Ramos WT and LRBA-KO cells were additionally treated with 50 µM Blebbistatin 30 min prior to cell seeding. Blebbistatin was present during IS formation. Images were acquired using a 63x objective (1.4 NA oil immersion) with a confocal microscope (LSM880, Zeiss), and analyzed with Fiji software, version 2.9.0. Formation of a mature IS after contact of Ramos cells with anti-IgM coated surfaces was determined by a positive signal for FITC-Phalloidin, AlexaFluor555-Pericentrin (PCNT) and AlexaFluor647-CD107a (Lamp1). Only cells with a triple-positive signal were considered as IS forming cells. The frequency of immune synapse formation per imaged field was calculated as [number of B cells showing with triple positive signal/total number of B cells (estimated by DAPI staining)]×100.

### Immune synapse analysis using coated beads

Goat F(ab’)_2_ anti-human IgM (Jackson ImmunoResearch) was covalently linked to 3 µm polystyrene carboxylate microspheres (Polysciences). Covalent coupling was performed using PolyLink Protein Coupling Kit (Polysciences). Briefly, beads were resuspended in PolyLink coupling buffer (50 mM MES pH = 5.2, 0.05% BSA, 0.05% Proclin 300) and carbodiimide solution at 200 mg/mL, and incubated with end-to-end rotation for 15 min at RT. 100 µg goat F(ab’)² anti-human IgM diluted in PBS were added to the beads and incubated under rotation for 60 min at RT. Beads were washed and resuspended in PolyLink wash/storage buffer. For analysis of lysosomes, F-actin and the MTOC, WT and LRBA-KO Ramos cells were seeded on Poly-L-lysine coated 8-chamber 1.5H coverslips (Ibidi) and labeled with 50 nM Lysotracker green DND-26 (Invitrogen), 1 mM SPY-555 FastAct (Spirochrome) and 1 mM SPY-650 tubulin (Spirochrome). Cells were then stimulated with activation beads coupled to F(ab’)_2_ anti-human µIgM at a 2:1 bead to cell ratio, and allowed to sediment on ice for 10 min. Images were acquired at 37 °C, 5% CO_2_ using an Olympus Ixplore SPIN (Olympus) spinning disk confocal microscope equipped with 63x objective (1.2 NA water immersion) and an ORCA-FUSION camera. Image analysis was performed using CellProfiler 4.2. Briefly, MTOC were segmented in the SPY-650 channel by three class Otsu’s thresholding method in single cell crops. Distance between the segmented MTOC and manually drawn beads were then measured. To quantify F-actin enrichment at the bead-cell interface, the bead outline and the cell outline were defined for each cell. The area of overlap between the bead and the cell outline was designated as the contact region (region 1), and a region of equal size at the opposite pole of the cell was defined as the distal reference region (region 2). F-actin enrichment was calculated by normalizing the SPY555-FastAct MFI of region 1 to the SPY555-FastAct MFI of region 2. For analysis of BCR polarization towards the bead and LRBA and pMLC co-localization, WT and LRBA-KO cells were seeded on Poly-L-lysine coated 8-chamber 1.5H coverslips (Ibidi) for 1 h at 37 °C, stimulated with activation beads coupled to F(ab’)_2_ anti-human µIgM at a 2:1 bead to cell ratio for 30 min and fixed with 4% PFA. Cells were blocked with blocking solution (2% BSA, 0.01% Triton X-100) for 2 h at RT and stained with anti-lambda (Biolegend), anti-pMLC (Cell Signaling) or anti-LRBA (Sigma) O/N at 4 °C in staining solution (1% BSA). The following day, cells were washed with PBS and stained with AlexaFluor555 anti-mouse (Cell Signaling), AlexaFluor647 anti-mouse (Cell Signaling) or AlexaFluor647 anti-rabbit (Cell Signaling) for 1 h at RT. Cells were washed three times with PBS for 10 min, stained with DAPI (Thermo Fisher) for 5 min at RT and mounted with DuoLink In Situ Mounting Medium (Merck). Images were acquired using a 63x objective (1.4 NA oil immersion) with a confocal microscope (LSM710, Zeiss), and analyzed with Fiji software, version 2.9.0. To analyze BCR polarization towards the bead, single focal plane images were used. For each cell–bead pair, ROIs were manually drawn in Fiji around the B cell and the bead. A Polarization Index (PI) was calculated by projecting the displacement between the intensity-weighted centroid and the geometric centroid of the cell ROI onto the unit vector pointing from the cell toward the bead, normalized by the effective cell radius. A PI of +1 indicates complete BCR polarization toward the bead, −1 indicates polarization away from the bead, and 0 indicates a non-polarized distribution. Co-localization between LRBA and pMLC was assessed using Pearson’s correlation coefficient with threshold using the JACoP plugin (Bolte and Cordelières, 2006).

### BCR internalization and bead phagocytosis

Ramos WT and LRBA-KO cells and naive B cells from HD and LRBA-P5 were incubated with biotin anti-IgM (Thermo Fisher) for 30 min for binding and then for BCR internalization for 0 min, 4 min, 8 min, 16 min, 30 min and 1 h (Ramos cells) or 0 min, 15 min and 30 min (naive B cells) at 37 °C or for bead phagocytosis of Ramos WT and LRBA-KO cells with 1 μm biotin anti-IgM coated fluorescent beads for 0 min, 30 min, 1 h, 2 h and 3 h at 37 °C in a 1:10 cell:bead ratio. After incubation, cells were washed and stained with DyLight633 Neutravidin (Invitrogen) and then acquired using FACSCanto II (BD Biosciences). For BCR internalization with Blebbistatin treatment, Ramos WT and LRBA-KO cells were treated with 50 µM Blebbistatin during anti-IgM binding and BCR internalization. Data analysis was performed using FlowJo 10.10.0 software (Treestar Inc.). For bead phagocytosis carboxylate YG 1 µm microspheres (Polysciences) were coated with 40 μg/ml anti-IgM (Thermo Fisher) O/N at 4 °C under continuous rotation. The following day, beads were washed and coating was confirmed by flow cytometry.

### Calcium release

Ramos WT and LRBA-KO cells were incubated with 4.5 μM Indo-1 (ATT Bioquest) diluted in pluronic acid (ATT Bioquest), DMSO and FCS at 37 °C for 45 min with gentle shaking every 15 min. Post-incubation, cells were kept on ice and protected from light. Prior to acquisition, cells were incubated at 37 °C for 5 min. Basal calcium levels were recorded for 50 s and subsequently, cells were stimulated with 2 μM Ionomycin (Cell Signaling) or 0.5 μg/ml anti-IgM (Biolegend). Calcium flux was recorded up to 5 min post-stimulation using LSRFortessa (BD Bioscience). Data analysis and area under the curve (AUC) calculation were performed using FlowJo software version 10.4.

### F-actin polymerization

Ramos WT and LRBA-KO cells were seeded for 30 min at 37 °C on multispot microscope slides (Hendley-Essex) previously coated with 0.1% Poly-L-lysine. Cells were left unstimulated (0 s) or stimulated with 5 μg/ml anti-IgM (Thermo Fisher) for 5 s, 15 s, 30 s, 1 min and 5 min at 37 °C and fixed with 4% PFA for 15 min at RT. Afterwards, cells were blocked with blocking solution (1% BSA + 0.2% Saponin in PBS) for 30 min at RT and stained with FITC-Phalloidin (Merck) for 45 min at RT. Cells were washed with blocking solution and PBS, and then stained with DAPI (Thermo Fisher) for 5 min at RT and mounted with DuoLink In Situ Mounting Medium (Merck). Images were acquired using a 63x objective (1.4 NA oil immersion) with a confocal microscope (LSM880, Zeiss) and were analyzed with Fiji software, version 2.9.0.

### BCR polarization

Ramos WT and LRBA-KO cells were seeded for 30 min at 37 °C on multispot microscope slides (Hendley-Essex) previously coated with 0.01% Poly-L-lysine. Cells were stimulated with 5 μg/ml AlexaFluor647 F(ab’)2 goat anti-IgG+IgM (H + L) (Jackson Immunoresearch) for 1 min, 5 min, 15 min, 30 min and 60 min at 37 °C and fixed with 4% PFA for 15 min at RT. Afterwards, cells were blocked with blocking solution (1% BSA + 0.2% Saponin in PBS) for 30 min at RT, washed with blocking solution and PBS, and then stained with DAPI (Thermo Fisher) for 5 min at RT and mounted with DuoLink In Situ Mounting Medium (Merck). Images were acquired using a 63x objective (1.4 NA oil immersion) with a confocal microscope (LSM710, Zeiss) and were analyzed with Fiji software, version 2.9.0. To analyze BCR polarization, Z-stack images were acquired and maximum intensity projections were generated. For each cell, a single ROI was manually drawn in Fiji (ImageJ) based on the T-PMT channel and applied to all channels. For each channel, the geometric centroid (x, y) and the intensity-weighted centroid (xm, ym) were extracted. The Polarization Index (PI) was calculated as the Euclidean distance between the intensity-weighted centroid and the geometric centroid, normalized by the effective cell radius: ${PI}=\frac{\sqrt{{({x}_{m}-x)}^{2}+{({y}_{m}-y)}^{2}}}{{R}_{{cell}}}.$ A PI of 0 indicates a uniform, non-polarized distribution of the signal within the cell, while higher values indicate increasing polarization of the signal away from the cell center.

### Surface IgM distribution

WT and LRBA-KO cells were stained with AlexaFluor647 Fab goat anti-IgM, Fc5µ (Jackson Immunoresearch) for 30 min at 4 °C for surface BCR labelling to prevent BCR internalization. Cells were washed, transferred onto 0.01% Poly-L-lysine coated coverslips and incubated for 20 min at 4 °C, fixed with 4% PFA at RT and mounted with DuoLink In Situ Mounting Medium (Merck). Z-Stacks were acquired using a 63x objective (1.4 NA oil immersion) with a confocal microscope (LSM880, Zeiss). Images were processed using the Zen Airyscan Processing module (ZEN Blue, 3.11, Zeiss), followed by AutoQuant Deconvolution (Image-Pro, 11.0, Media Cybernetics). A two-dimensional image representation of surface IgM was obtained via maximum intensity projection across the acquired z-stacks. To assess the spatial organization of IgM, the positive IgM signal was separated from the background via Otsu thresholding. Next, the Hopkins Index (H) was derived, with each positive pixel considered an individual data point.

### Planar lipid bilayers

Artificial planar lipid bilayers were assembled in FCS2 chambers (Bioptechs) as described previously (Carrasco et al, 2004). Briefly, unlabeled murine 1,2-dioleoyl-PC (DOPC) liposomes and DOPC liposomes containing biotinylated lipids were mixed with DOPC liposomes at distinct ratios to obtain molecular densities of 30 and 100 molecules/μm^2^. Artificial planar lipid bilayers were assembled on sulfochromic acid-treated coverslips in FCS2 closed flow chambers (Bioptechs) and blocked with PBS + 2% FCS for 1 h at RT. su-Ag was tethered to membranes by incubation with AlexaFluor647-conjugated streptavidin (Molecular Probes), followed by monobiotinylated mouse anti-human IgM monoclonal antibody (hAb, clone G20-127, BD Pharming) only or with monobiotinylated mouse anti-human IgM monoclonal antibody and monobiotinylated recombinant human VCAM-1-Fc (R&D Systems). Monobiotinylation was previously done by labeling the antibody and VCAM-1-Fc with NHS-LC-LC-biotin (1 μg/ml; 30 min, RT, in PBS; Pierce), followed by dialysis and analysis by flow cytometry. WT and LRBA-KO Ramos cells were co-injected into the pre-warmed chambers for imaging. To distinguish WT and LRBA-KO cells, one cell type was labeled with 0.1 μM violet Celltracer (Molecular Probes) for 10 min at 37 °C. Cells were left in contact with the planar lipid bilayers for 15 min to form a mature immune synapse before imaging. To measure total F-actin intensity during immune synapse formation, 4% PFA was injected into the chambers after imaging and incubated for 10 min at 37 °C. Cells were permeabilized with PBS + 150 mM NaCl + 0.2% Triton X-100 for 5 min at RT, blocked with PBS + 50 mM NaCl + 1% BSA + 0.05% Tween-20 for 30 min at RT and stained with TRITC-conjugated Phalloidin (Molecular Probes) for 25 min at RT. Images were acquired using a 40x objective (1.4 NA oil immersion) with an Axiovert LSM 510 META inverted microscope and analyzed using Fiji software version 2.9.0, CellProfiler software 4.2.8 and Imaris software version 7.3.1. IS were defined as Ramos B cells forming a contact with the planar lipid bilayer as visualized by IRM. Only contacts displaying a dark, dot-like structure were considered bona fide synapses. Closed cSMACs were identified based on the estimated by the su-Ag–associated fluorescence and classified as closed when forming a round, dot-like fluorescent accumulation at the cell–bilayer interface. The frequency of immune synapse formation and closed cSMACs per imaged field was calculated as [number of B cells showing a closed cSMAC and IRM contact/total number of B cells (estimated by DIC)]×100.

### Transwell migration assay

Transwell migration was determined in Transwell chambers (5 μm pore size, Corning). Briefly, RPMI + 0.5% FCS + 1% P/S with or without 100 ng/ml CXCL12 (Peprotech) was added to 24-well plates (the lower chamber) while Ramos and LCL cells were seeded in the upper chamber. For analysis of migration with Blebbistatin treatment LCL cells were additionally treated with 10 µM Blebbistatin, which was present during the 6 h incubation. After 6 h incubation, Precision Count Beads were added and cells and 50,000 beads were acquired on a LSRFortessa (BD Bioscience) and data analysis was performed using FlowJo 10.10.0 software (Treestar Inc.).

### Cell migration assay in microchannels

Microdevices were prepared as previously described (Vargas et al, 2014). Briefly, devices were fabricated using polydimethylsiloxane and custom-made molds and coated with 10 μg/ml fibronectin (Sigma-Aldrich) for 1 h. HD, LRBA-P2 and P3 LCL cells were left untreated or treated with 25 µM Blebbistatin and let to migrate inside channels and recorded using a Zeiss Axio Observer Z1 microscope (ZEISS), with a time lapse of 1 or 2 min using a 10x objective (0.45 NA). Image analysis was performed using ImageJ. Kymographs for each channel were generated using a semiautomated macro and single-cell trajectories were manually isolated and analysed with a custom-made MATLAB (MathWorks) code to determine the cell speed. For constriction analysis, manual quantification was performed to obtain the percentage of cells that passed. The following outcomes were assessed: (1) passing and (2) not-passing cells.

For immunofluorescence analysis during migration in microchannels, minor adjustments were made to the microchannels as previously described (Gajardo et al, 2023). Cells were allowed to migrate into 8 μm-wide microchannels for 16 h and fixed with 4% PFA + 0.25% glutaraldehyde for 45 min at RT. After washing with PBS, microdevices were carefully detached. Cells were permeabilized with 0.05% Triton X-100, blocked with 5% BSA and stained with rabbit anti-LRBA (Sigma), anti-pMLC (Cell Signaling) and AlexaFluor488-Phalloidin (Thermo Fisher) for 2 h at RT. Cells were washed three times with PBS and incubated with AlexaFluor568 anti-mouse (Life Technologies), AlexaFluor647 anti-rabbit (Life Technologies) and Hoechst for 1 h at RT. Coverslips were rinsed with PBS and mounted with ProLong™ Gold Antifade mounting medium (Thermo Fisher). Images were acquired using a 63x objective (1.4 NA oil immersion) with a Zeiss Axio Observer Z1 microscope (Zeiss). Front-to-rear ratio was calculated by manually determining regions of interests (ROI) using Icy software at the front and rear of individual cells and measuring F-actin, pMLC and LRBA MFI in each ROI. Co-localization was analyzed using Imaris software (Oxford Instruments) and Pearson’s coefficient was calculated based on the voxel intensity within the cell volume.

### Statistical analysis

Data analysis was performed without blinding and statistical significance was calculated with Welch’s *t* test, one-way ANOVA with Tukey’s multiple comparison and two-way ANOVA with Tukey’s or Holm–Sidak multiple comparisons test using GraphPad Prism 10.0 software. A *P* value of *<*0.05 was considered statistically significant, *P < 0.05, **P < 0.01, ***P < 0.001, ****P < 0.0001.

## Supplementary information

Peer Review File

Dataset EV1

Source data Fig. 1

Source data Fig. 2

Source data Fig. 3

Source data Fig. 4

Source data Fig. 5

Expanded View Figures

## Data Availability

This study includes no data deposited in external repositories. The source data of this paper are collected in the following database record: biostudies:S-SCDT-10_1038-S44319-026-00831-3.
