# Supplementary figures and images for: LRBA regulates actin cytoskeleton dynamics through NMIIA during B cell immune responses
Source: EMBO Rep. 2026 Jun 12;27(14):3982–4010. doi: 10.1038/s44319-026-00831-3 (PMC13400755; doi:10.1038/s44319-026-00831-3)

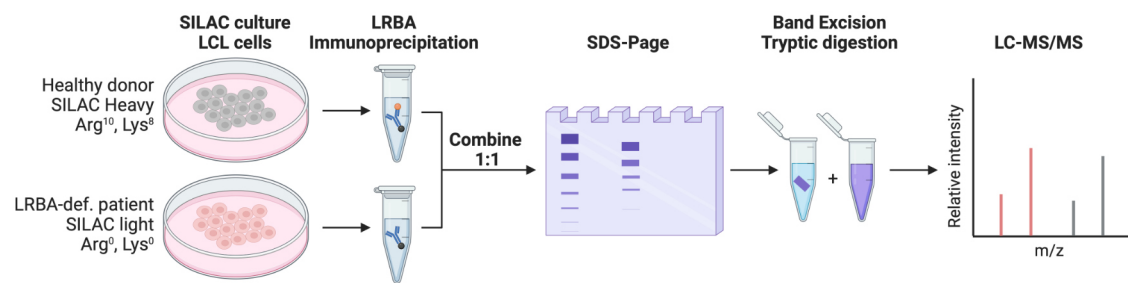

Supplement: Supplementary file 3 — Source data Fig. 1 [file 44319_2026_831_MOESM3_ESM.zip › Figure 1A/SILAC-IP-MS scheme.pdf]

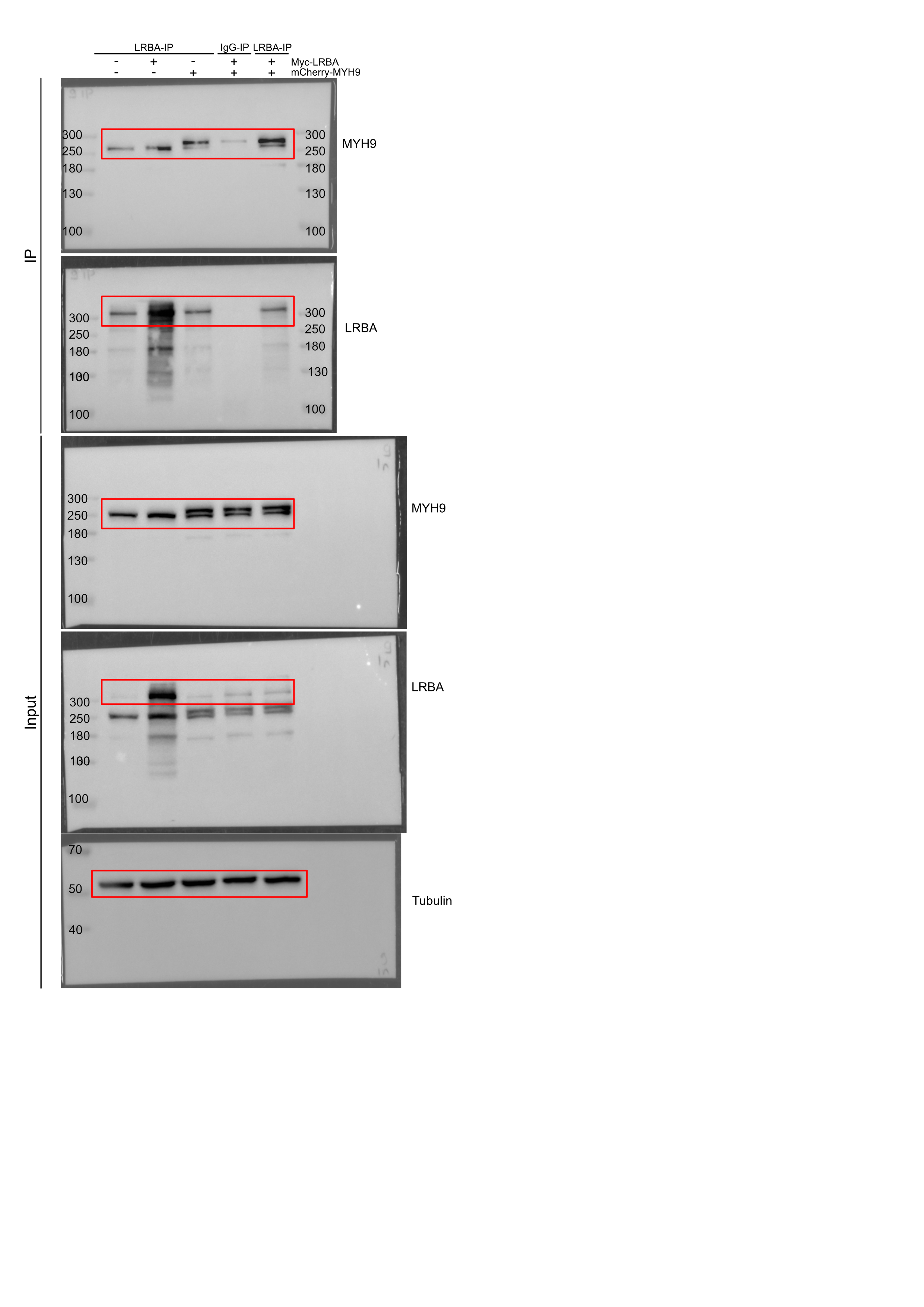

Supplement: Supplementary file 3 — Source data Fig. 1 [file 44319_2026_831_MOESM3_ESM.zip › Figure 1D/Co-IP LRBA and MYH9.png]

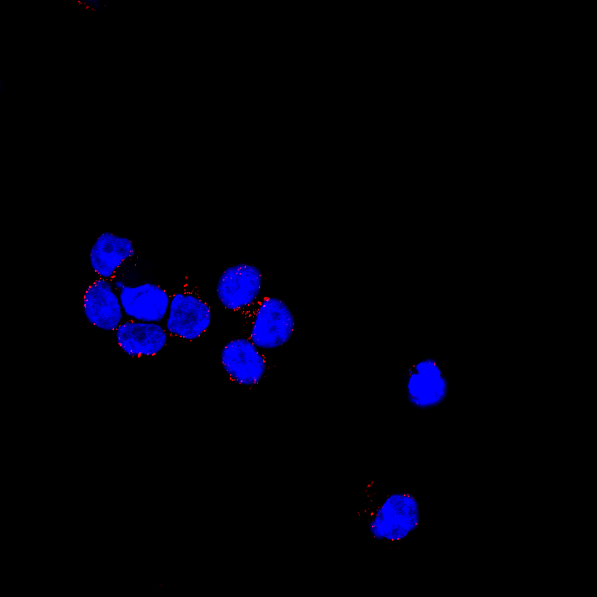

Supplement: Supplementary file 3 — Source data Fig. 1 [file 44319_2026_831_MOESM3_ESM.zip › Figure 1E/HD_PLA_LRBA-MYH9.tif]

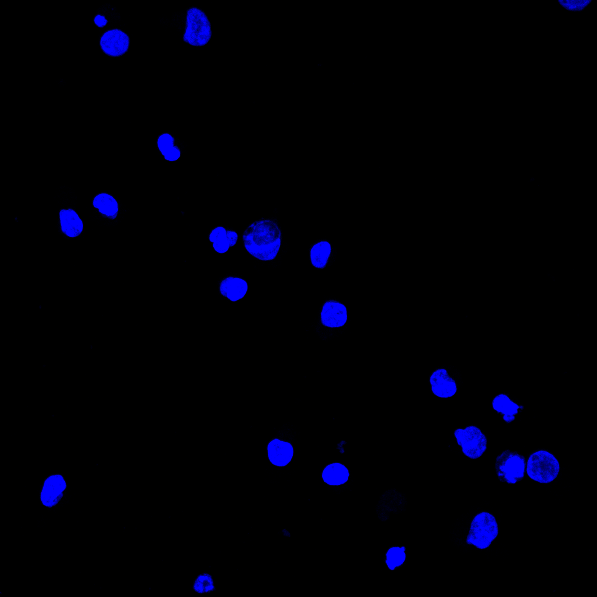

Supplement: Supplementary file 3 — Source data Fig. 1 [file 44319_2026_831_MOESM3_ESM.zip › Figure 1E/LRBA-P1_PLA_LRBA-MYH9.tif]

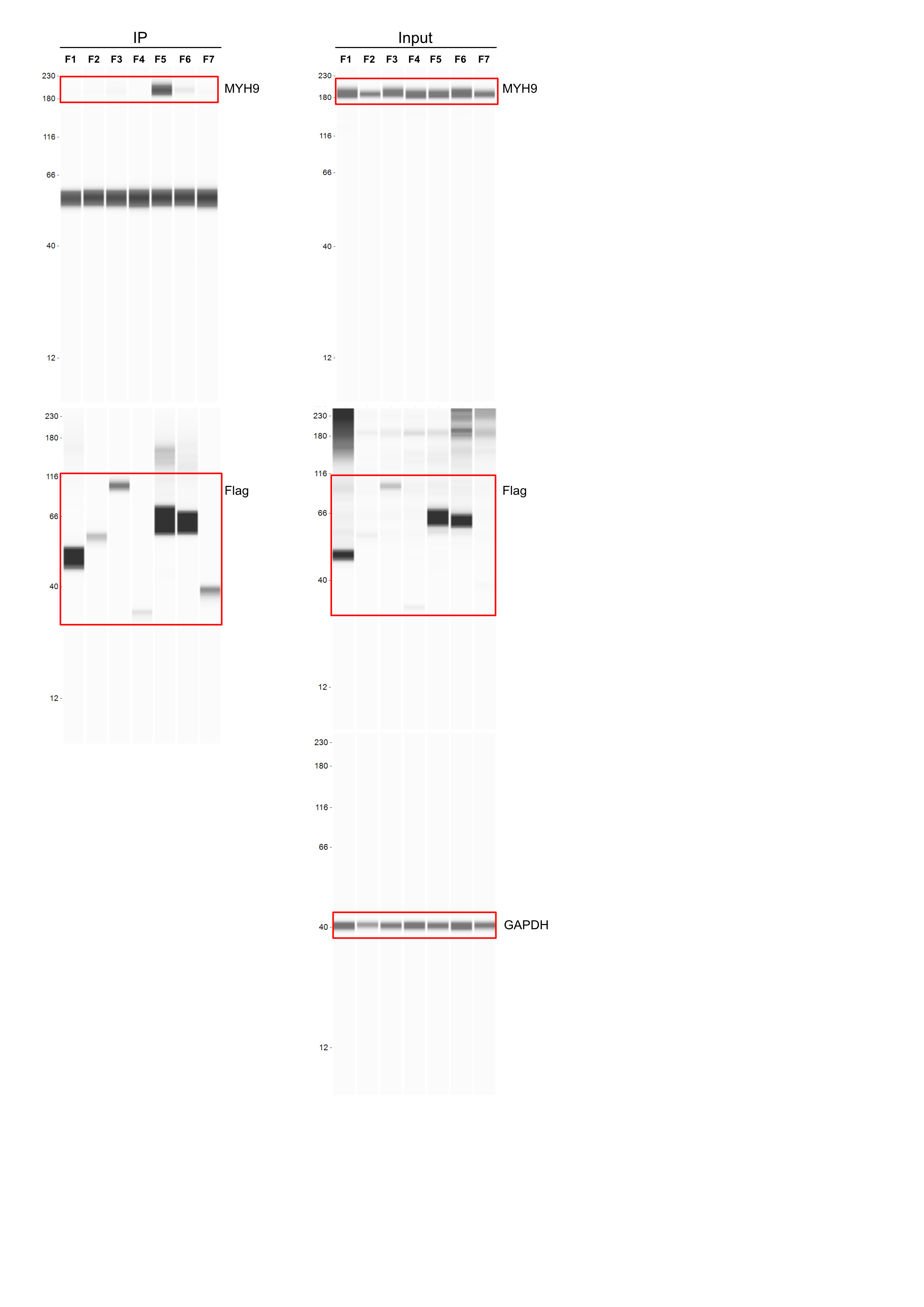

Supplement: Supplementary file 3 — Source data Fig. 1 [file 44319_2026_831_MOESM3_ESM.zip › Figure 1F/Co-IP LRBA Fragments and MYH9.png]

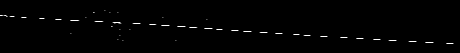

Supplement: Supplementary file 4 — Source data Fig. 2 [file 44319_2026_831_MOESM4_ESM.zip › Figure 2B/Kymograph HD.png]

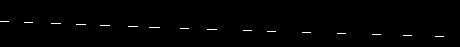

Supplement: Supplementary file 4 — Source data Fig. 2 [file 44319_2026_831_MOESM4_ESM.zip › Figure 2B/Kymograph LRBA-P2.png]

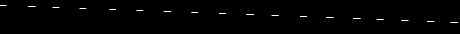

Supplement: Supplementary file 4 — Source data Fig. 2 [file 44319_2026_831_MOESM4_ESM.zip › Figure 2B/Kymograph LRBA-P3.png]

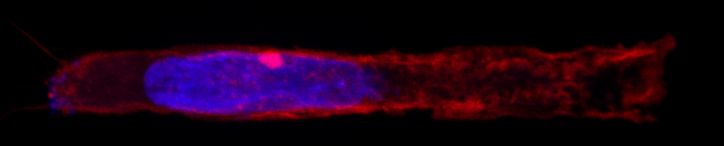

Supplement: Supplementary file 4 — Source data Fig. 2 [file 44319_2026_831_MOESM4_ESM.zip › Figure 2D/HD_F-actin.png]

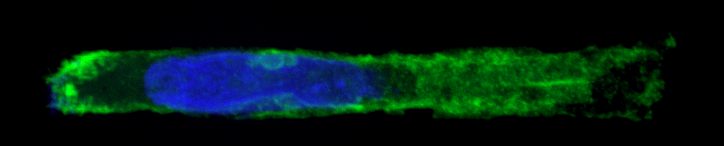

Supplement: Supplementary file 4 — Source data Fig. 2 [file 44319_2026_831_MOESM4_ESM.zip › Figure 2D/HD_pMLC.png]

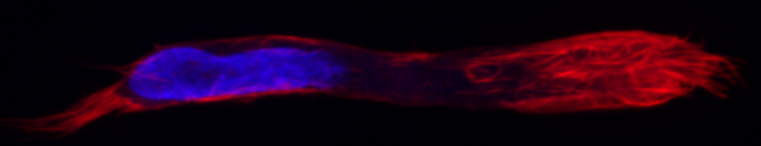

Supplement: Supplementary file 4 — Source data Fig. 2 [file 44319_2026_831_MOESM4_ESM.zip › Figure 2D/LRBA-P2_F-actin.png]

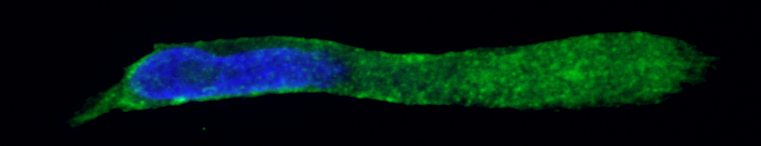

Supplement: Supplementary file 4 — Source data Fig. 2 [file 44319_2026_831_MOESM4_ESM.zip › Figure 2D/LRBA-P2_pMLC.png]

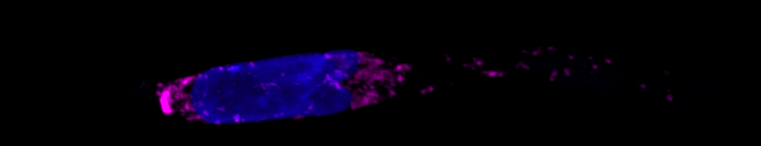

Supplement: Supplementary file 4 — Source data Fig. 2 [file 44319_2026_831_MOESM4_ESM.zip › Figure 2F/HD_LRBA.png]

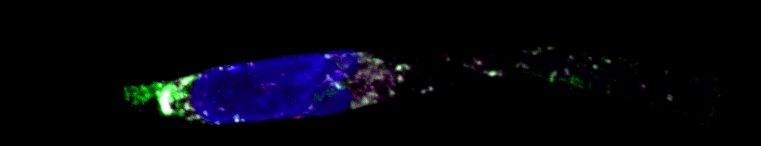

Supplement: Supplementary file 4 — Source data Fig. 2 [file 44319_2026_831_MOESM4_ESM.zip › Figure 2F/HD_Merge.png]

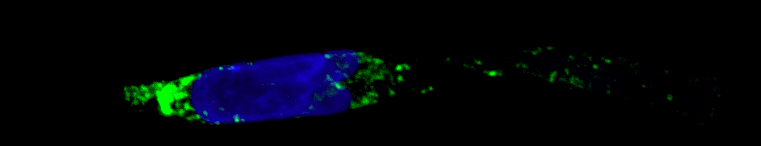

Supplement: Supplementary file 4 — Source data Fig. 2 [file 44319_2026_831_MOESM4_ESM.zip › Figure 2F/HD_pMLC.png]

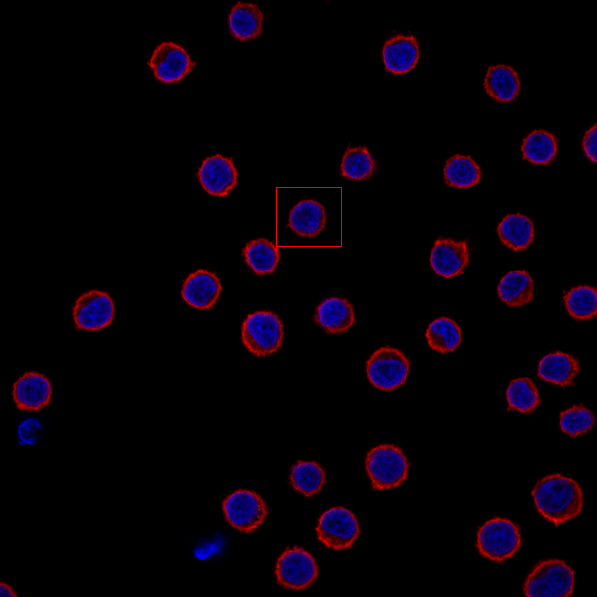

Supplement: Supplementary file 5 — Source data Fig. 3 [file 44319_2026_831_MOESM5_ESM.zip › Figure 3A/Ramos LRBA-KO_0 sec.tif]

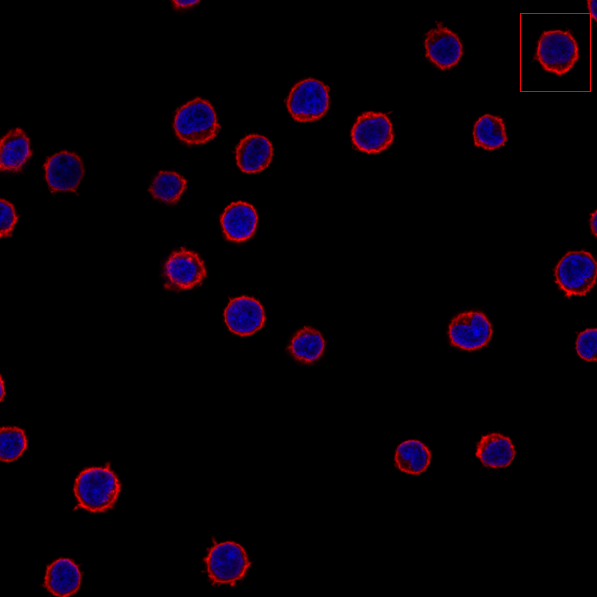

Supplement: Supplementary file 5 — Source data Fig. 3 [file 44319_2026_831_MOESM5_ESM.zip › Figure 3A/Ramos LRBA-KO_15 sec.tif]

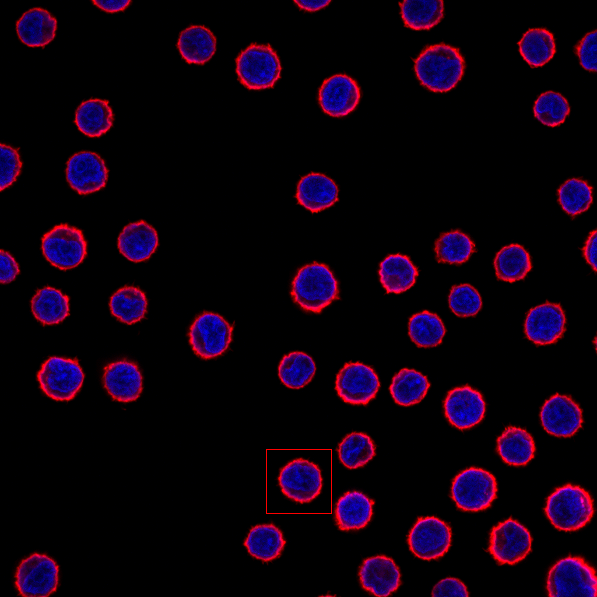

Supplement: Supplementary file 5 — Source data Fig. 3 [file 44319_2026_831_MOESM5_ESM.zip › Figure 3A/Ramos LRBA-KO_30 sec.tif]

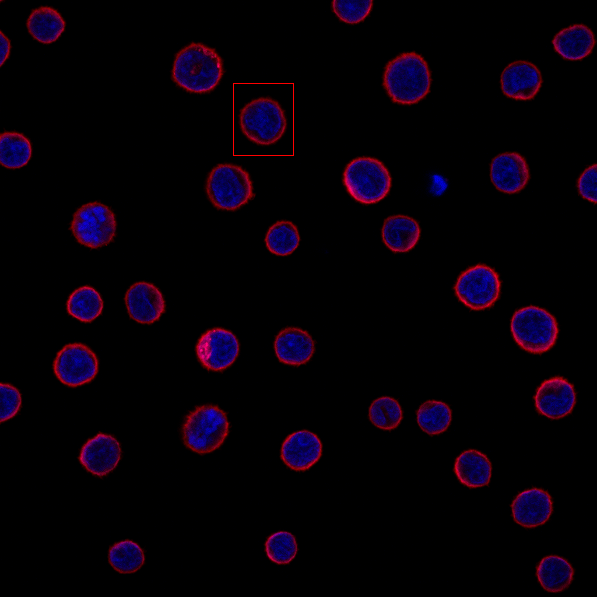

Supplement: Supplementary file 5 — Source data Fig. 3 [file 44319_2026_831_MOESM5_ESM.zip › Figure 3A/Ramos LRBA-KO_300 sec.tif]

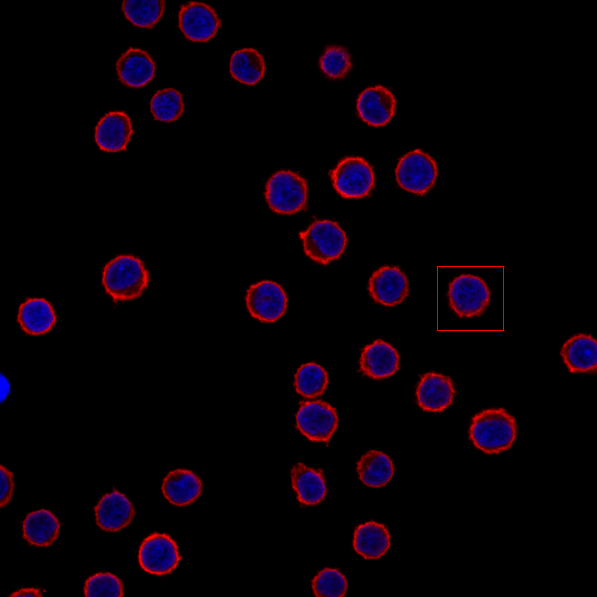

Supplement: Supplementary file 5 — Source data Fig. 3 [file 44319_2026_831_MOESM5_ESM.zip › Figure 3A/Ramos LRBA-KO_5 sec.tif]

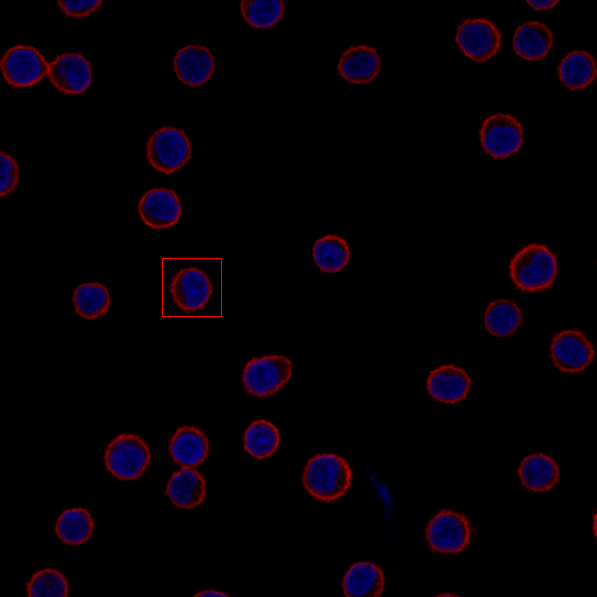

Supplement: Supplementary file 5 — Source data Fig. 3 [file 44319_2026_831_MOESM5_ESM.zip › Figure 3A/Ramos LRBA-KO_60 sec.tif]

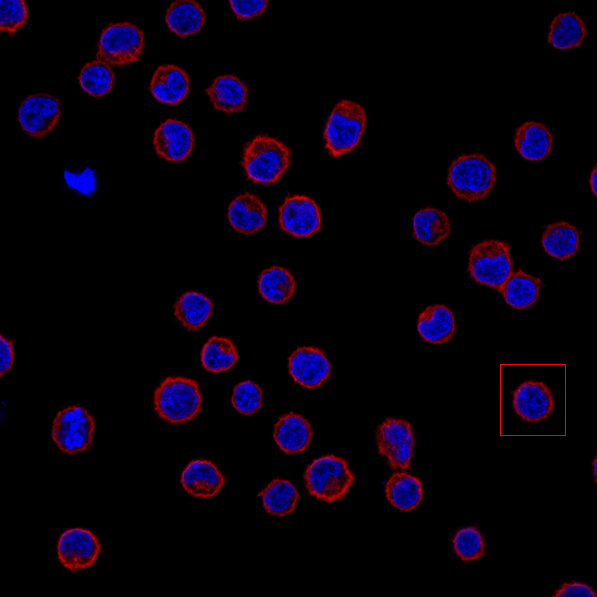

Supplement: Supplementary file 5 — Source data Fig. 3 [file 44319_2026_831_MOESM5_ESM.zip › Figure 3A/Ramos WT_0 sec.tif]

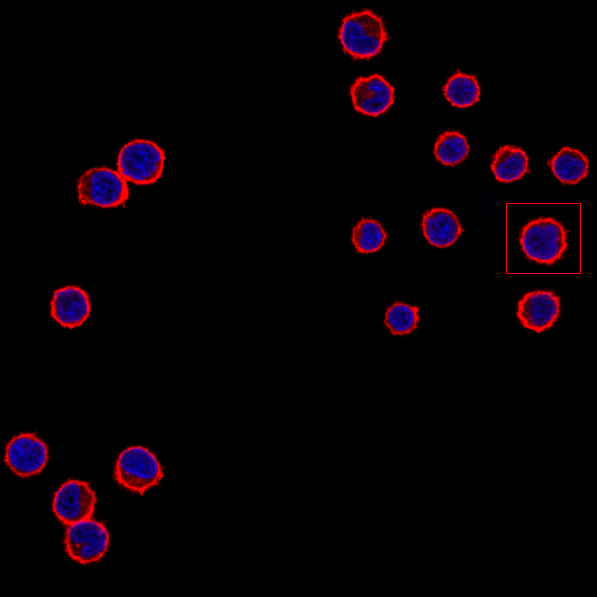

Supplement: Supplementary file 5 — Source data Fig. 3 [file 44319_2026_831_MOESM5_ESM.zip › Figure 3A/Ramos WT_15 sec.tif]

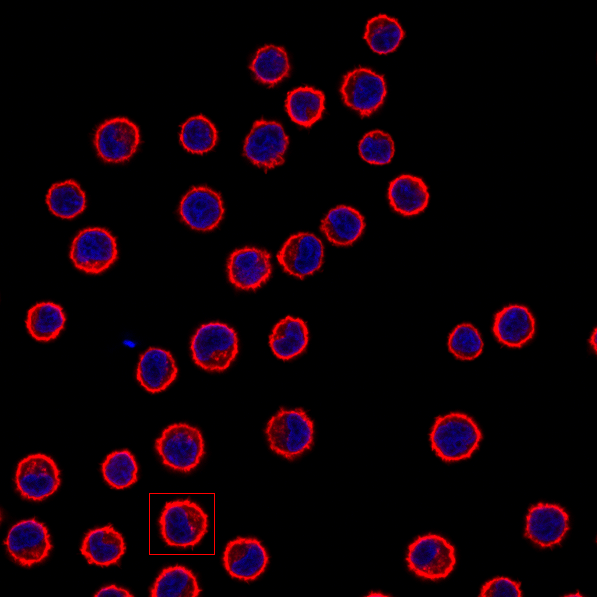

Supplement: Supplementary file 5 — Source data Fig. 3 [file 44319_2026_831_MOESM5_ESM.zip › Figure 3A/Ramos WT_30 sec.tif]

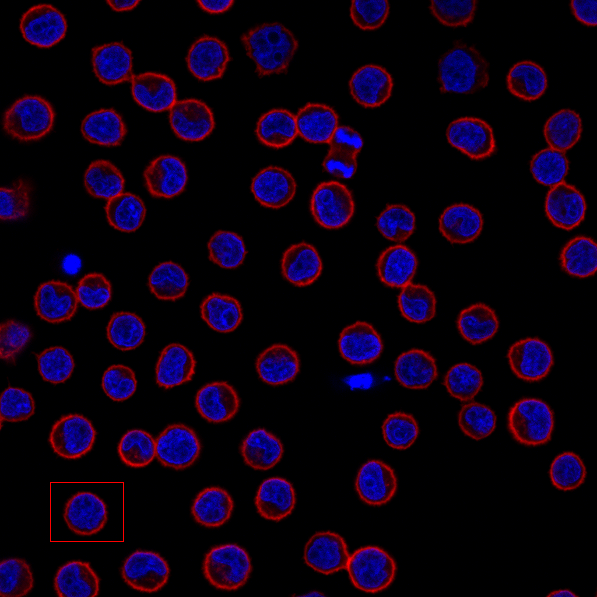

Supplement: Supplementary file 5 — Source data Fig. 3 [file 44319_2026_831_MOESM5_ESM.zip › Figure 3A/Ramos WT_300 sec.tif]

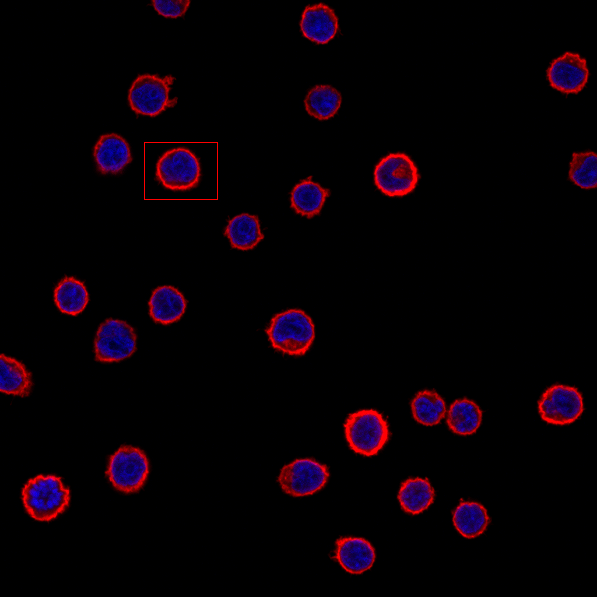

Supplement: Supplementary file 5 — Source data Fig. 3 [file 44319_2026_831_MOESM5_ESM.zip › Figure 3A/Ramos WT_5 sec.tif]

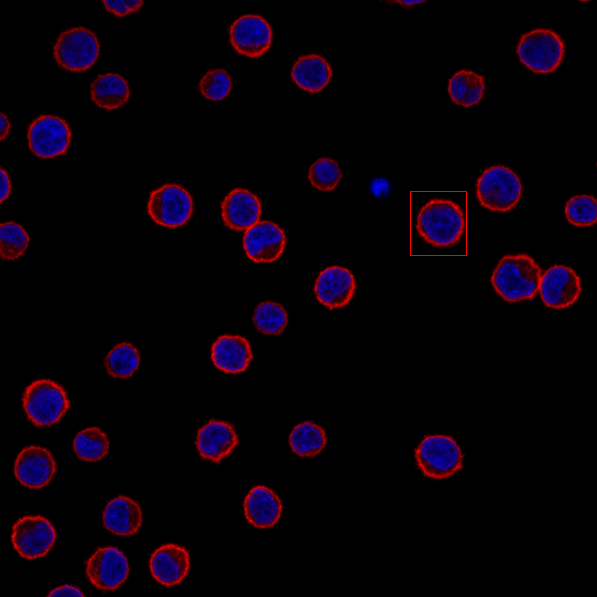

Supplement: Supplementary file 5 — Source data Fig. 3 [file 44319_2026_831_MOESM5_ESM.zip › Figure 3A/Ramos WT_60 sec.tif]

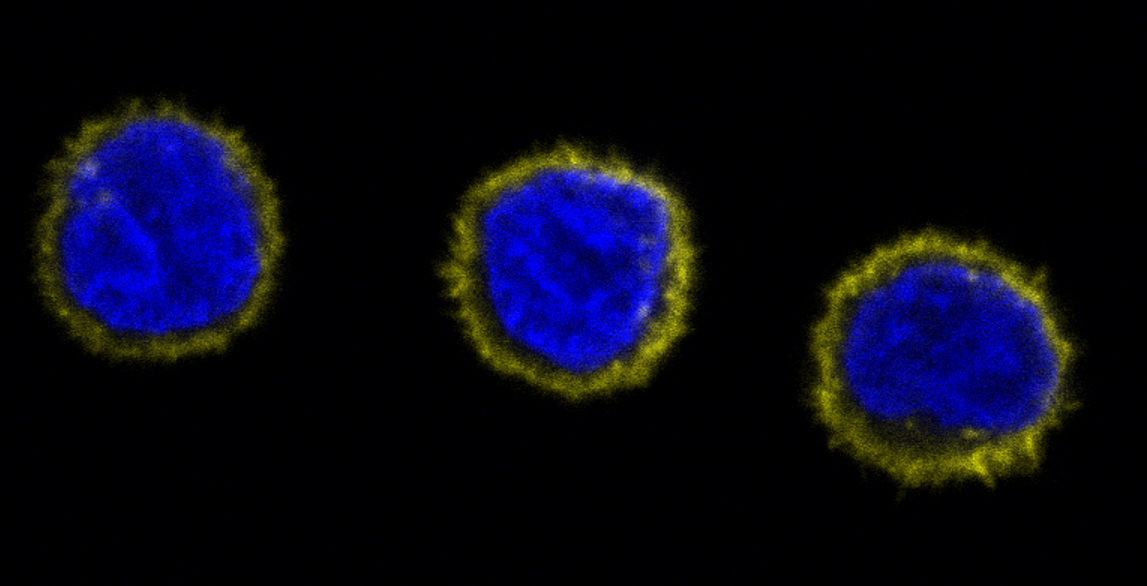

Supplement: Supplementary file 5 — Source data Fig. 3 [file 44319_2026_831_MOESM5_ESM.zip › Figure 3G/Ramos LRBA-KO_15min_BCR+DAPI.tif]

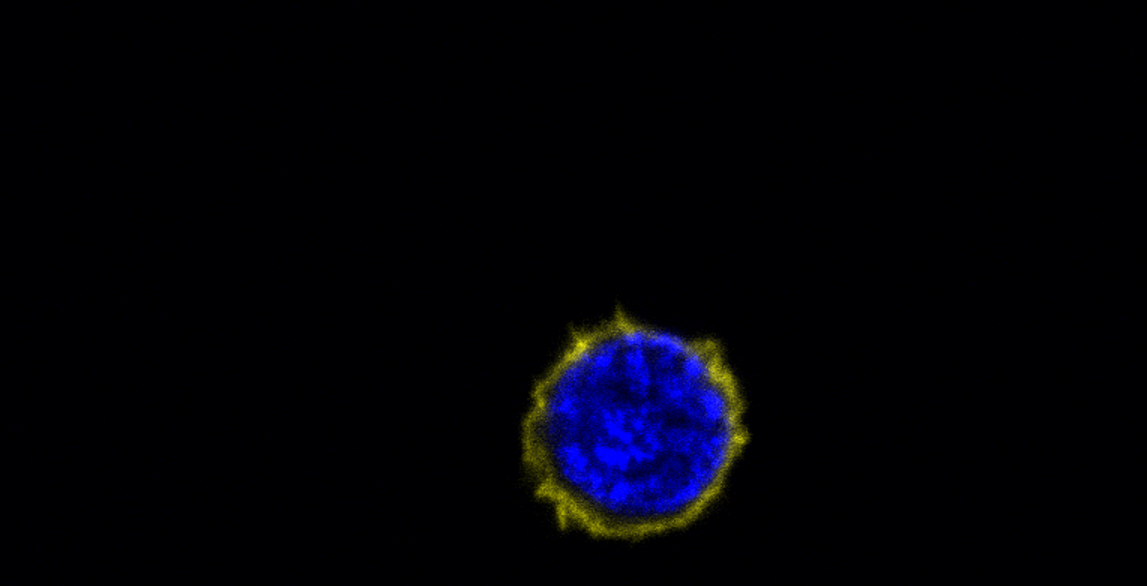

Supplement: Supplementary file 5 — Source data Fig. 3 [file 44319_2026_831_MOESM5_ESM.zip › Figure 3G/Ramos LRBA-KO_1min_BCR+DAPI.tif]

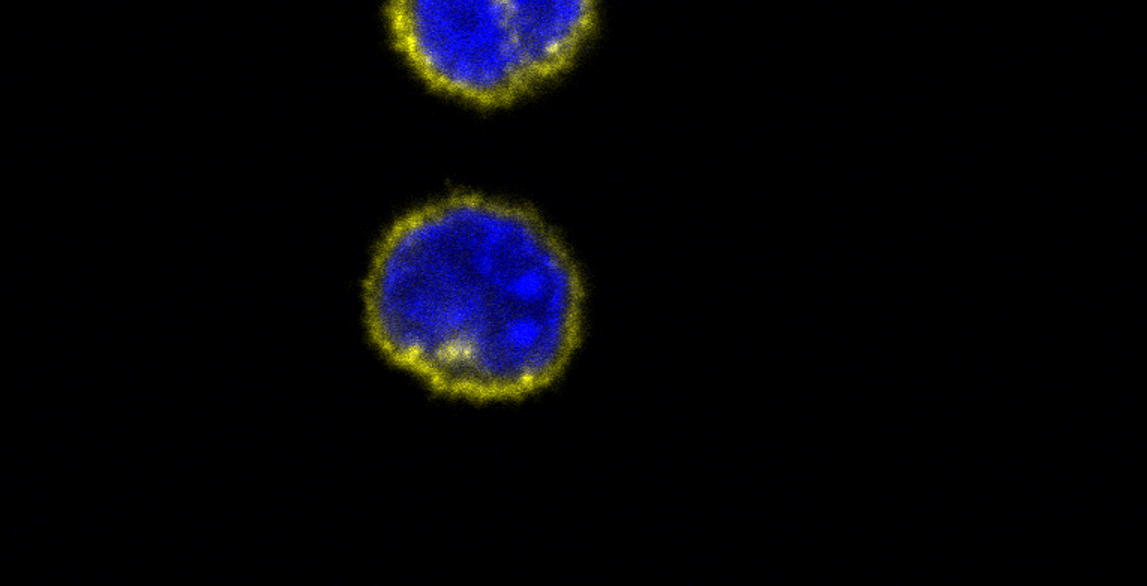

Supplement: Supplementary file 5 — Source data Fig. 3 [file 44319_2026_831_MOESM5_ESM.zip › Figure 3G/Ramos LRBA-KO_30min_BCR+DAPI.tif]

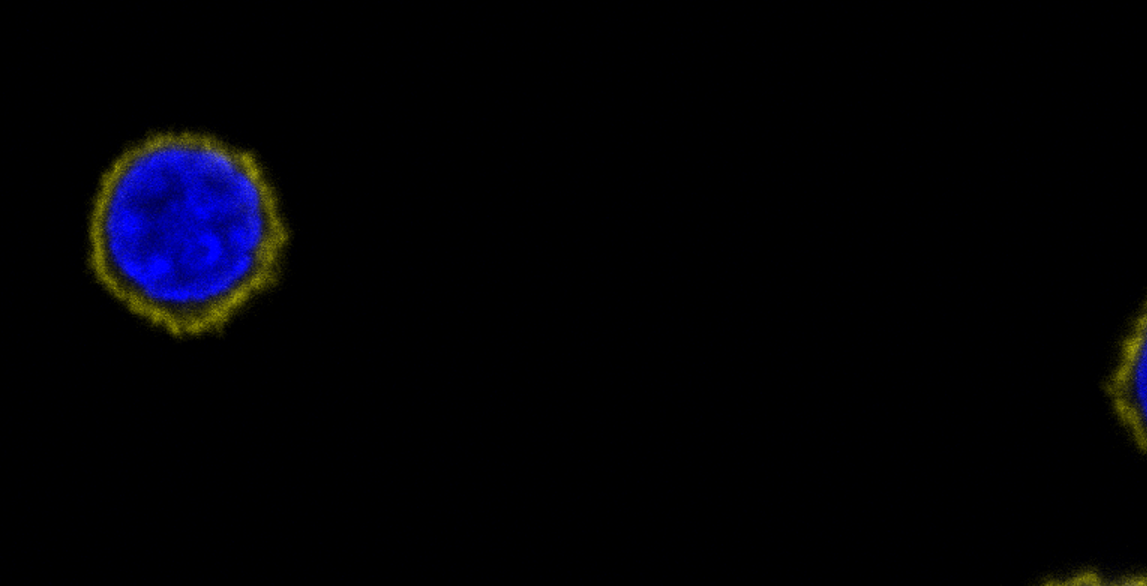

Supplement: Supplementary file 5 — Source data Fig. 3 [file 44319_2026_831_MOESM5_ESM.zip › Figure 3G/Ramos LRBA-KO_5min_BCR+DAPI.tif]

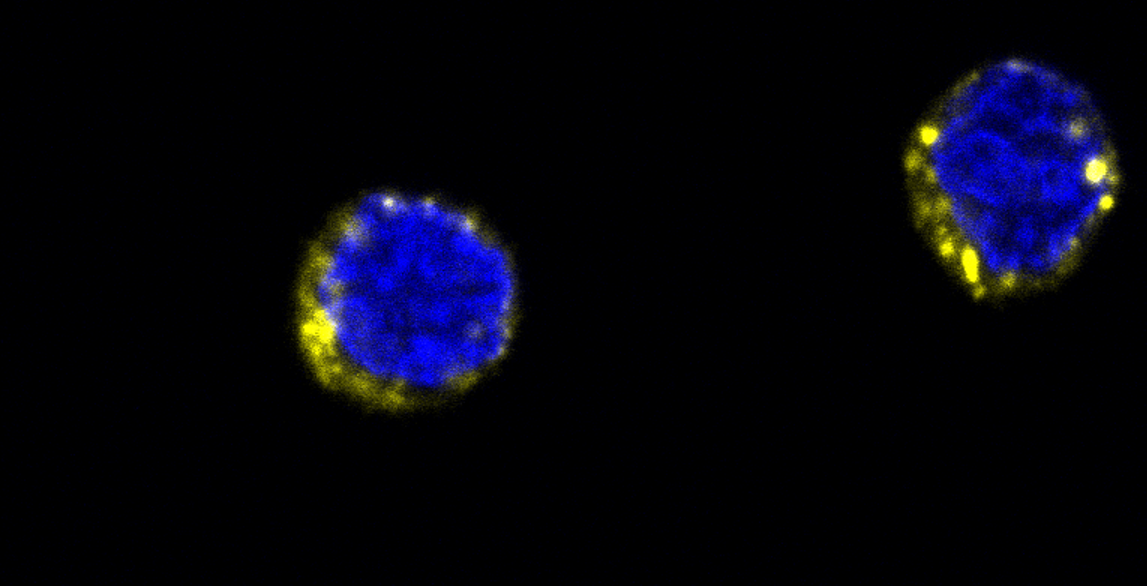

Supplement: Supplementary file 5 — Source data Fig. 3 [file 44319_2026_831_MOESM5_ESM.zip › Figure 3G/Ramos LRBA-KO_60min_BCR+DAPI.tif]

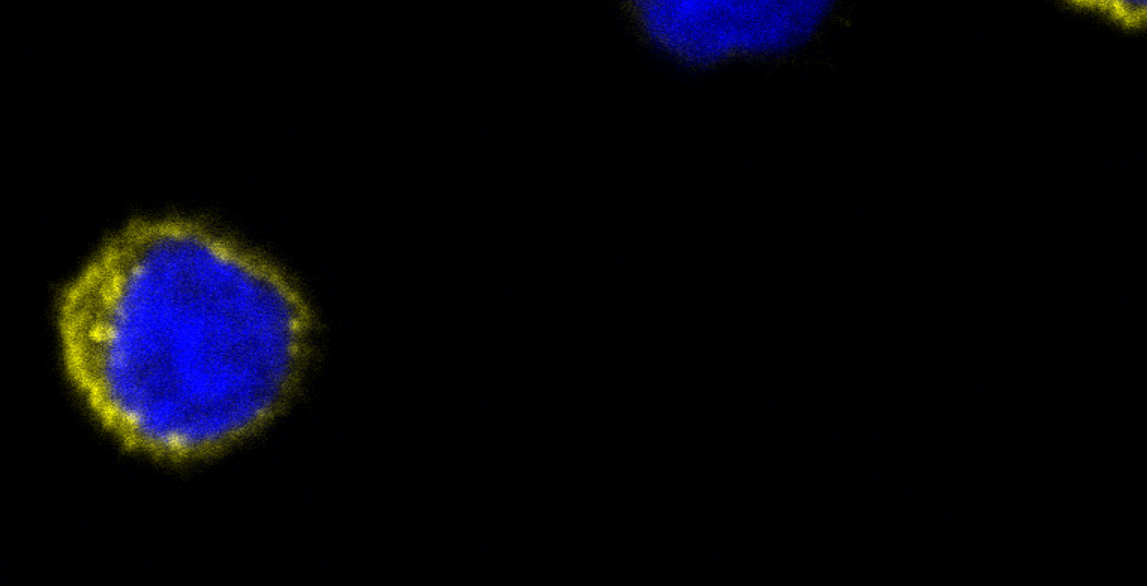

Supplement: Supplementary file 5 — Source data Fig. 3 [file 44319_2026_831_MOESM5_ESM.zip › Figure 3G/Ramos WT_15min_BCR+DAPI.tif]

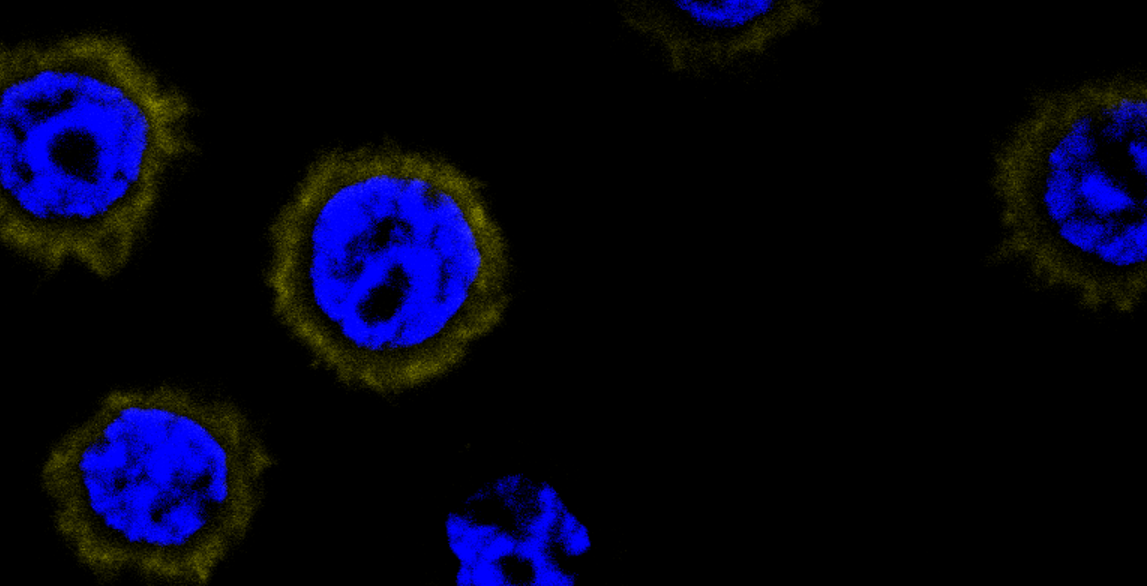

Supplement: Supplementary file 5 — Source data Fig. 3 [file 44319_2026_831_MOESM5_ESM.zip › Figure 3G/Ramos WT_1min_BCR+DAPI.tif]

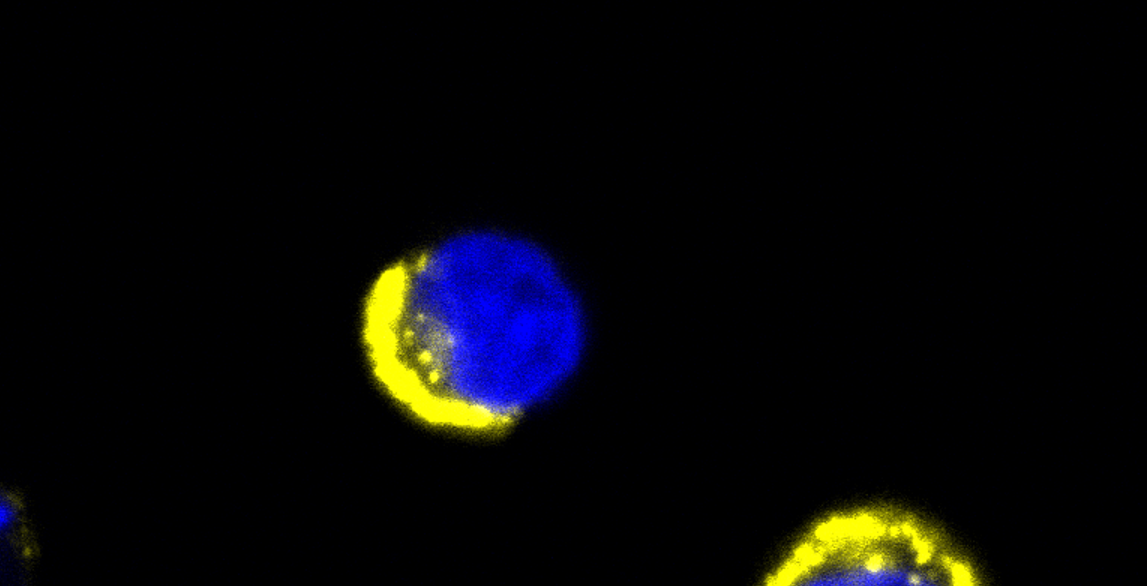

Supplement: Supplementary file 5 — Source data Fig. 3 [file 44319_2026_831_MOESM5_ESM.zip › Figure 3G/Ramos WT_30min_BCR+DAPI.tif]

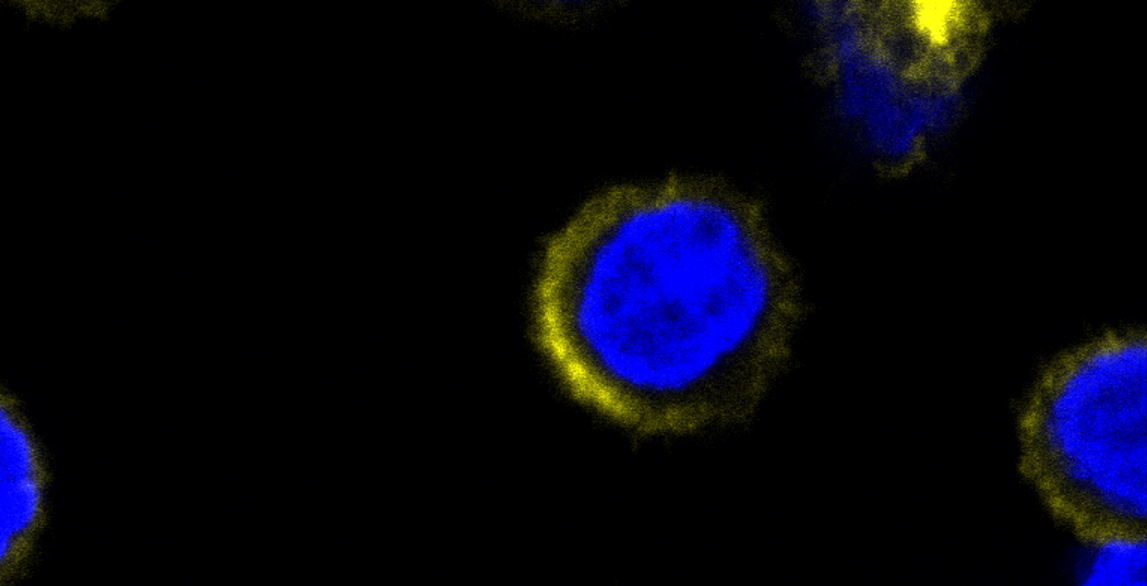

Supplement: Supplementary file 5 — Source data Fig. 3 [file 44319_2026_831_MOESM5_ESM.zip › Figure 3G/Ramos WT_5min_BCR+DAPI.tif]

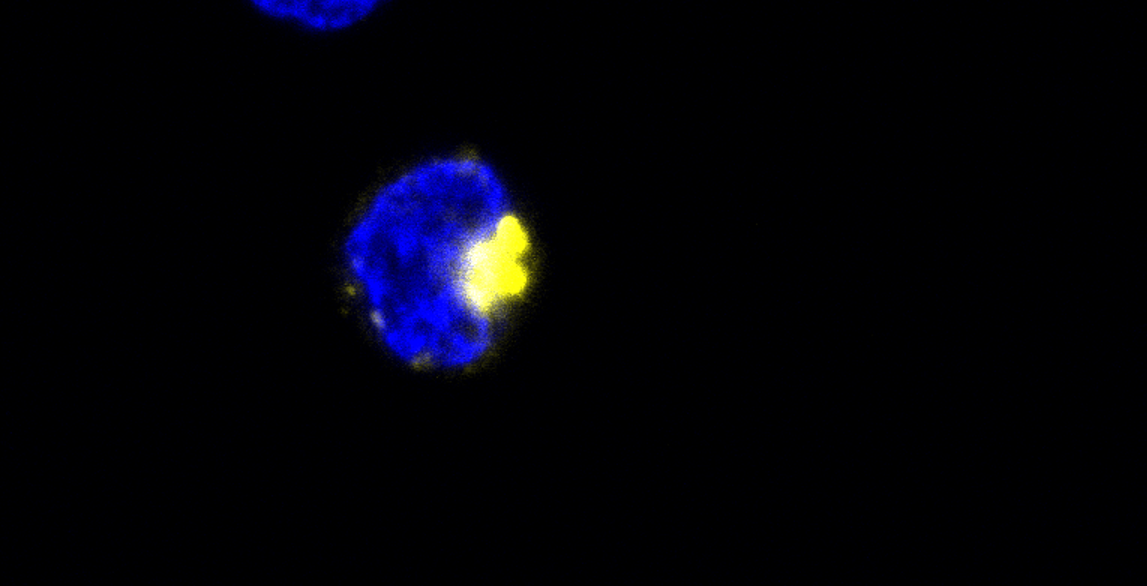

Supplement: Supplementary file 5 — Source data Fig. 3 [file 44319_2026_831_MOESM5_ESM.zip › Figure 3G/Ramos WT_60min_BCR+DAPI.tif]

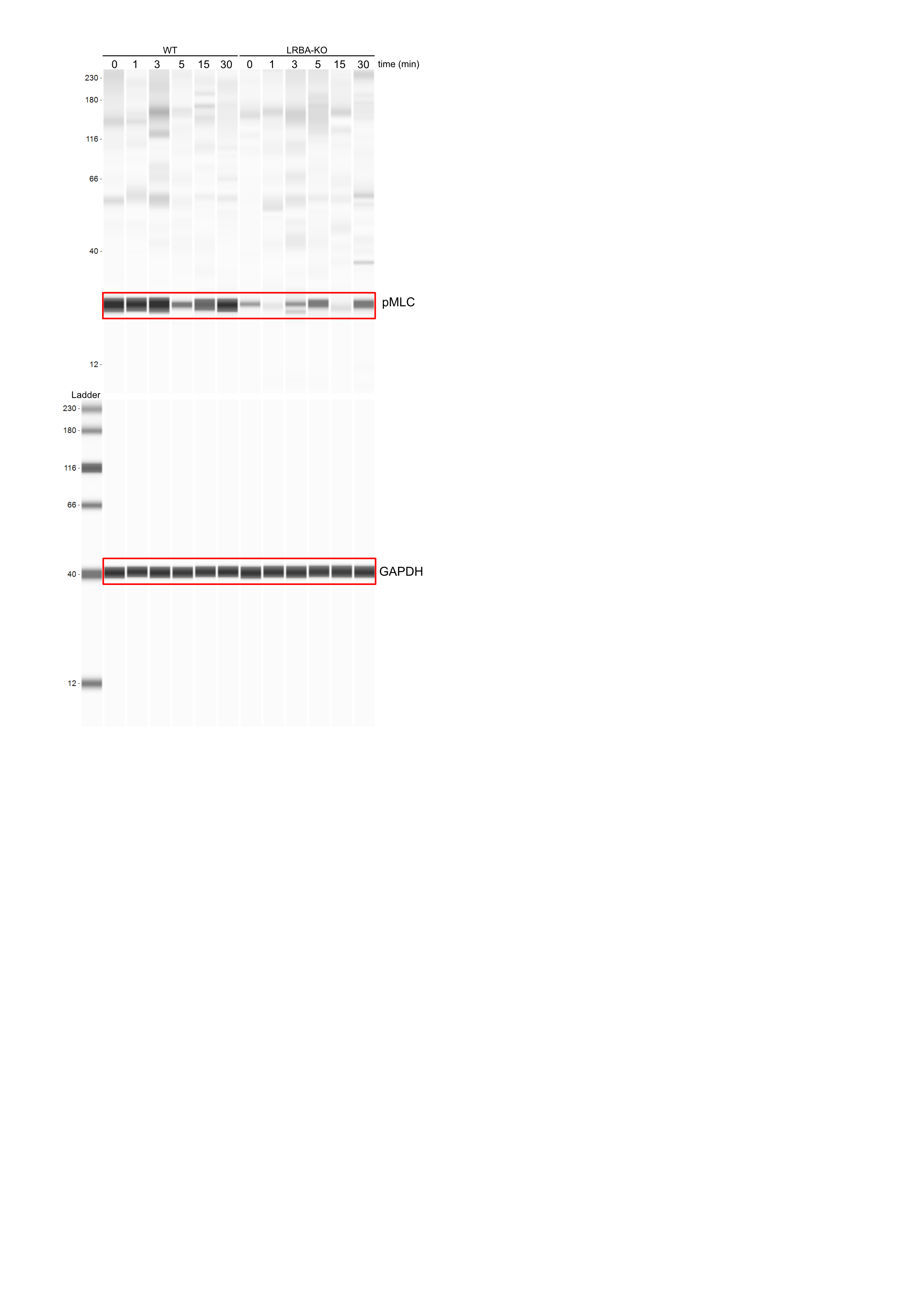

Supplement: Supplementary file 5 — Source data Fig. 3 [file 44319_2026_831_MOESM5_ESM.zip › Figure 3I/pMLC Western Blot.png]

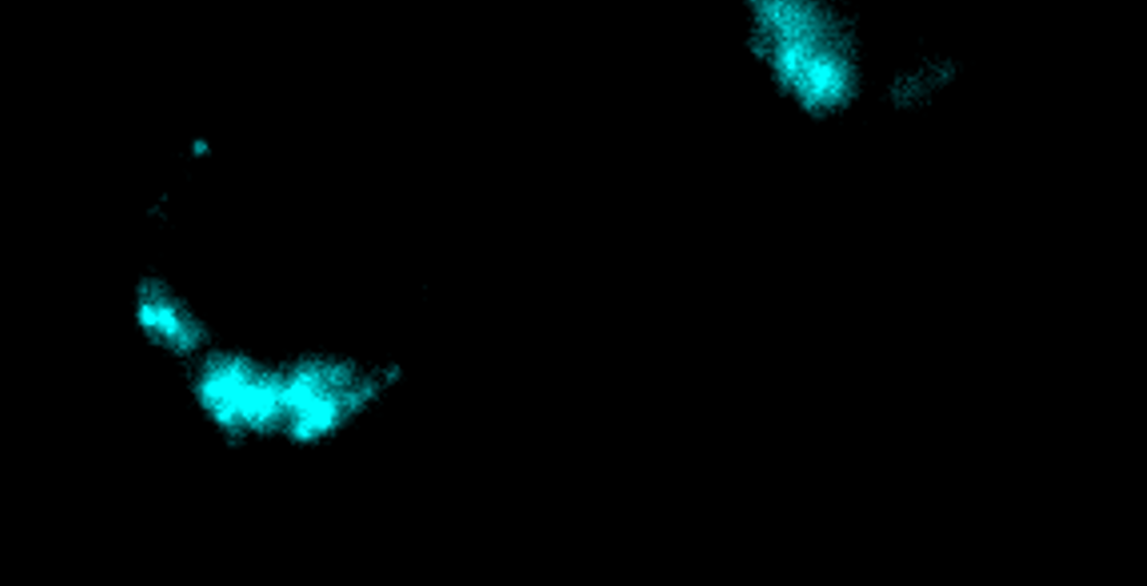

Supplement: Supplementary file 6 — Source data Fig. 4 [file 44319_2026_831_MOESM6_ESM.zip › Figure 4A/Ramos LRBA-KO_Lamp1.tif]

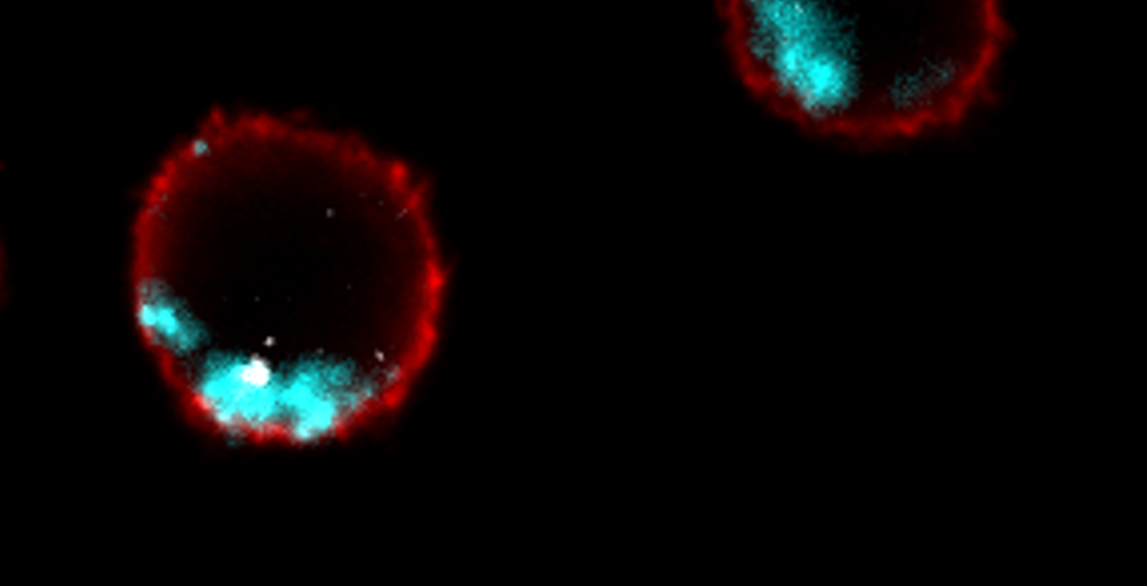

Supplement: Supplementary file 6 — Source data Fig. 4 [file 44319_2026_831_MOESM6_ESM.zip › Figure 4A/Ramos LRBA-KO_Merge.tif]

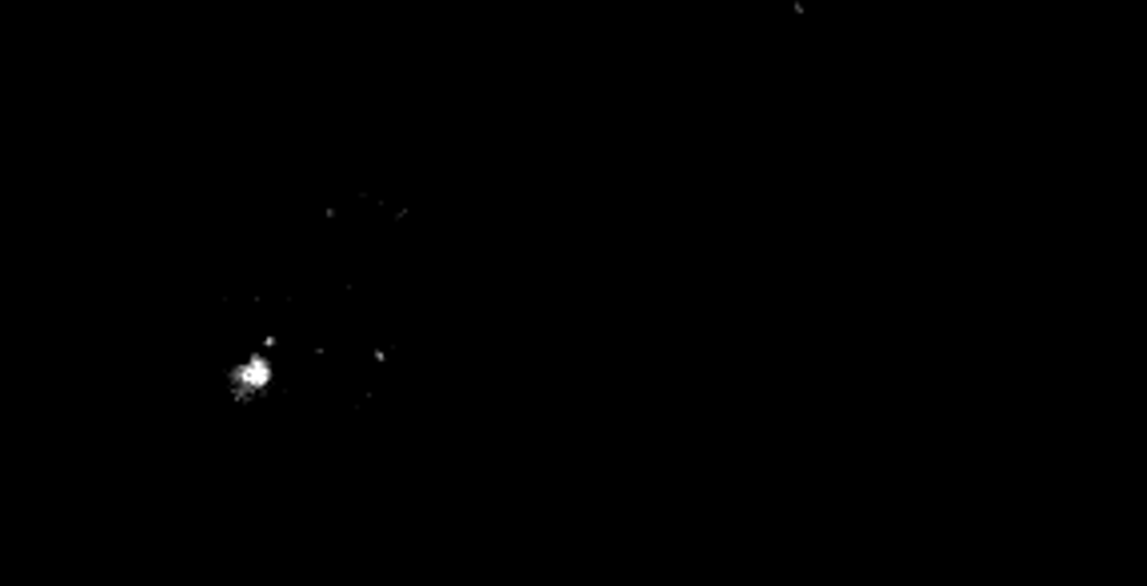

Supplement: Supplementary file 6 — Source data Fig. 4 [file 44319_2026_831_MOESM6_ESM.zip › Figure 4A/Ramos LRBA-KO_PCNT.tif]

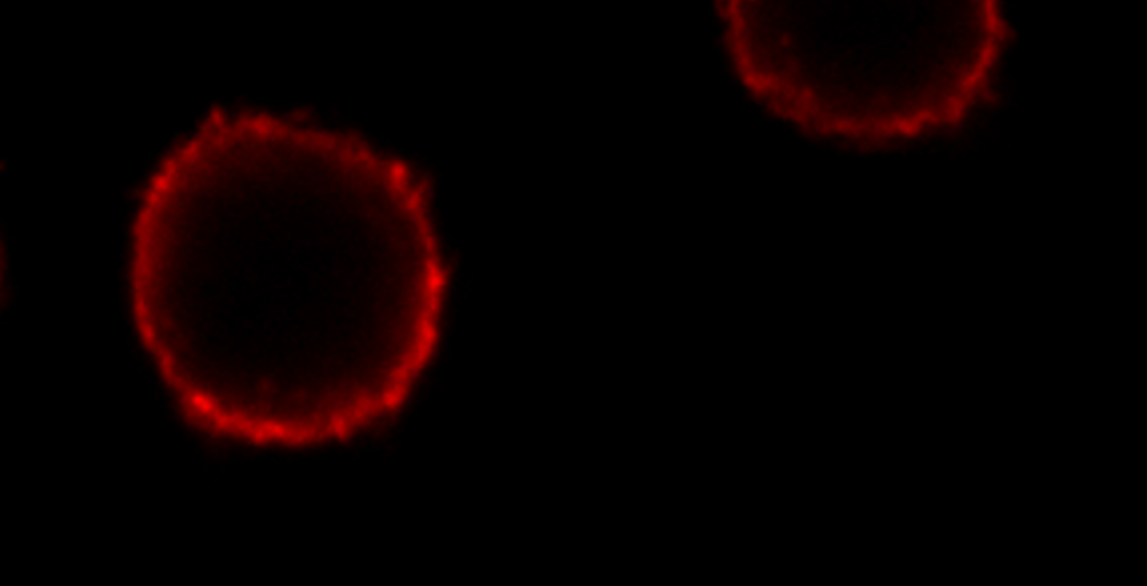

Supplement: Supplementary file 6 — Source data Fig. 4 [file 44319_2026_831_MOESM6_ESM.zip › Figure 4A/Ramos LRBA-KO_Phalloidin.tif]

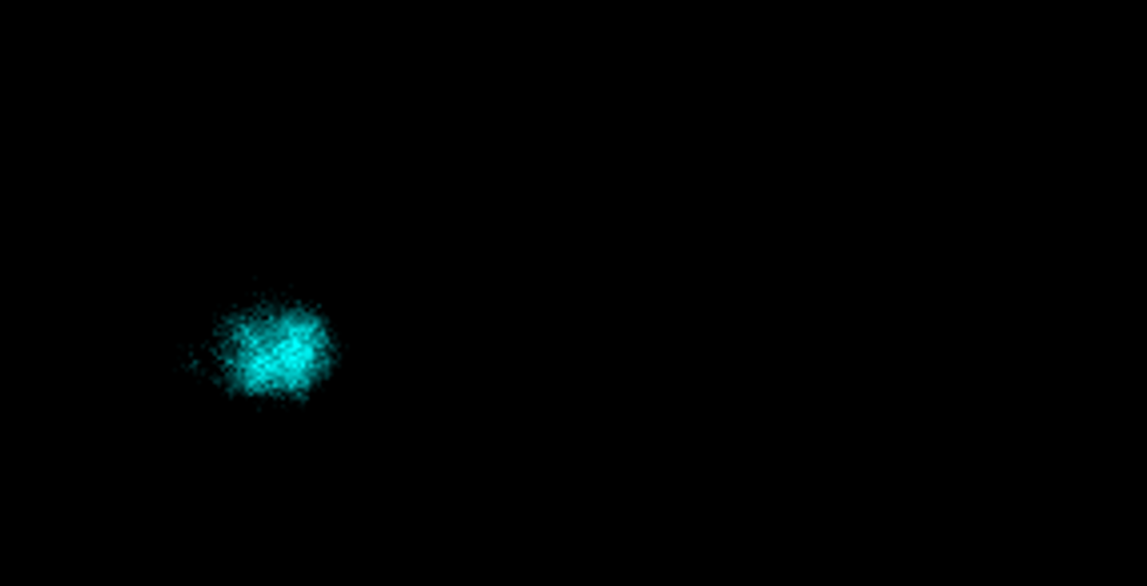

Supplement: Supplementary file 6 — Source data Fig. 4 [file 44319_2026_831_MOESM6_ESM.zip › Figure 4A/Ramos Myc-LRBA_Lamp1.tif]

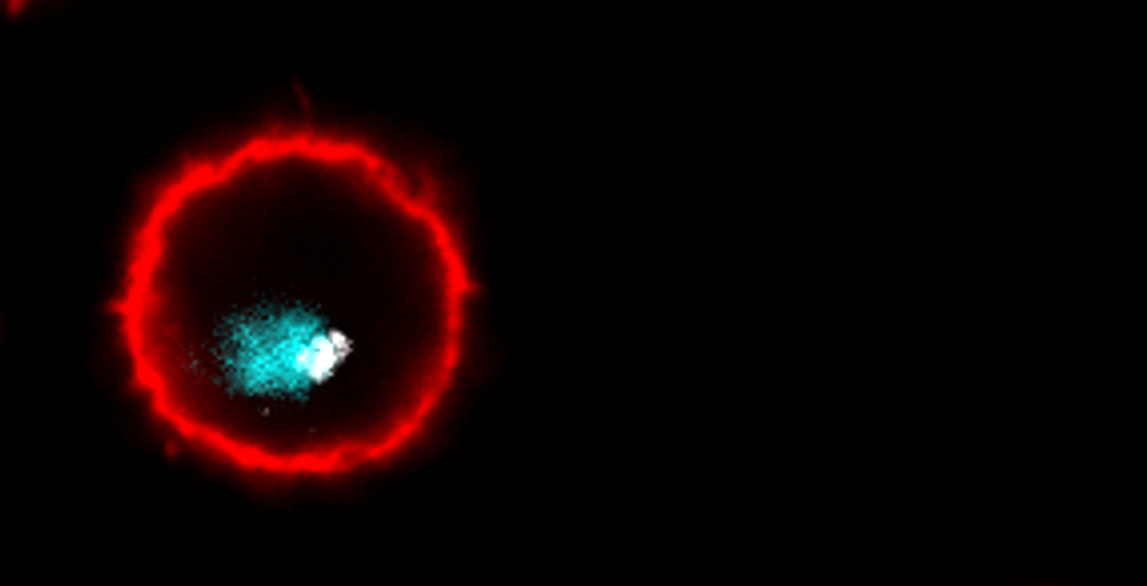

Supplement: Supplementary file 6 — Source data Fig. 4 [file 44319_2026_831_MOESM6_ESM.zip › Figure 4A/Ramos Myc-LRBA_Merge.tif]

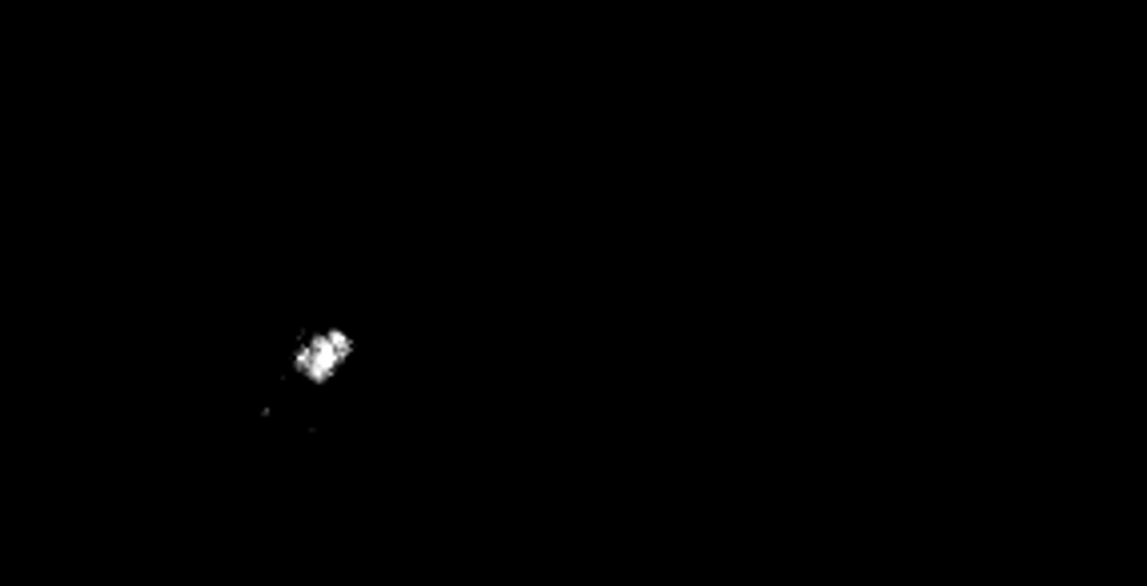

Supplement: Supplementary file 6 — Source data Fig. 4 [file 44319_2026_831_MOESM6_ESM.zip › Figure 4A/Ramos Myc-LRBA_PCNT.tif]

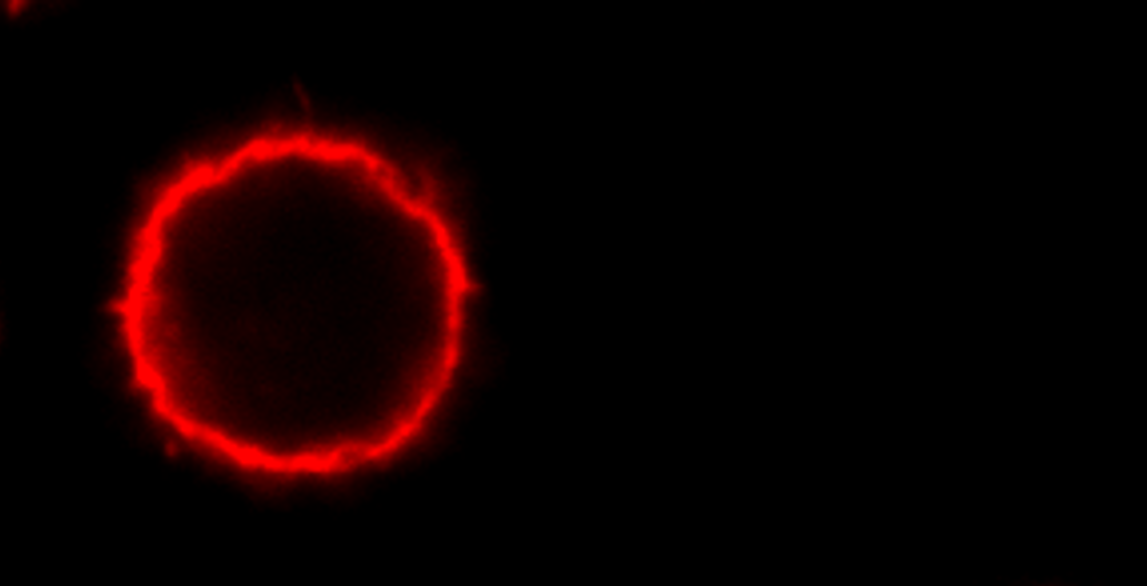

Supplement: Supplementary file 6 — Source data Fig. 4 [file 44319_2026_831_MOESM6_ESM.zip › Figure 4A/Ramos Myc-LRBA_Phalloidin.tif]

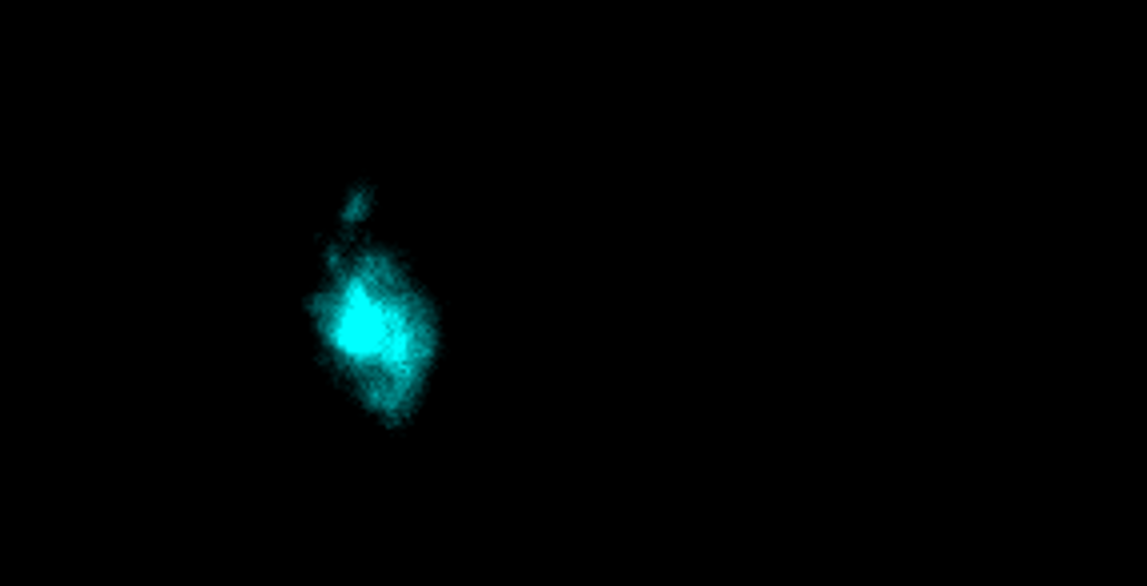

Supplement: Supplementary file 6 — Source data Fig. 4 [file 44319_2026_831_MOESM6_ESM.zip › Figure 4A/Ramos WT_Lamp1.tif]

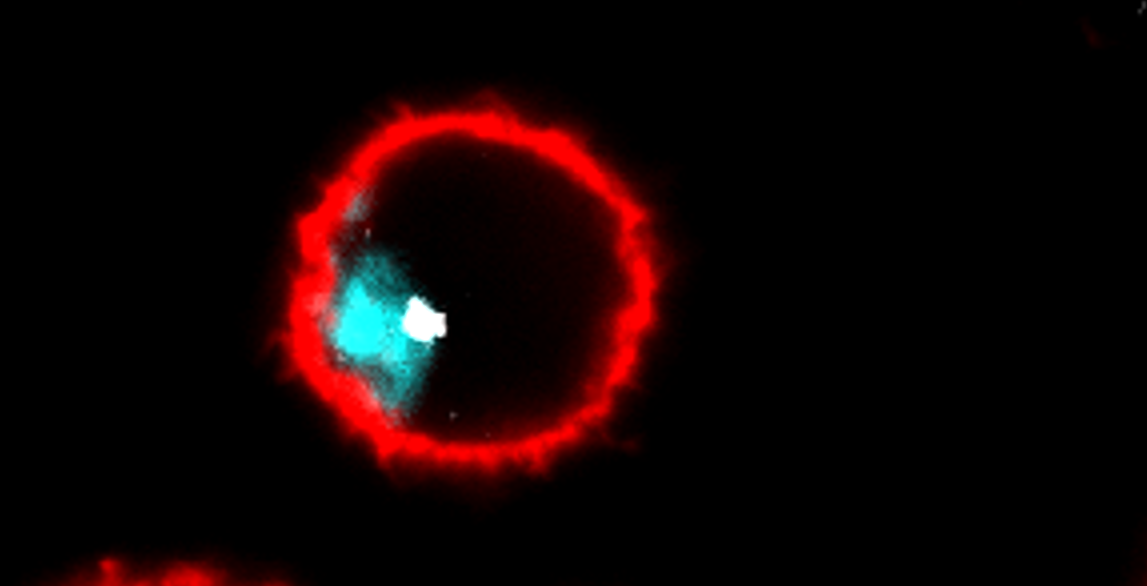

Supplement: Supplementary file 6 — Source data Fig. 4 [file 44319_2026_831_MOESM6_ESM.zip › Figure 4A/Ramos WT_Merge.tif]

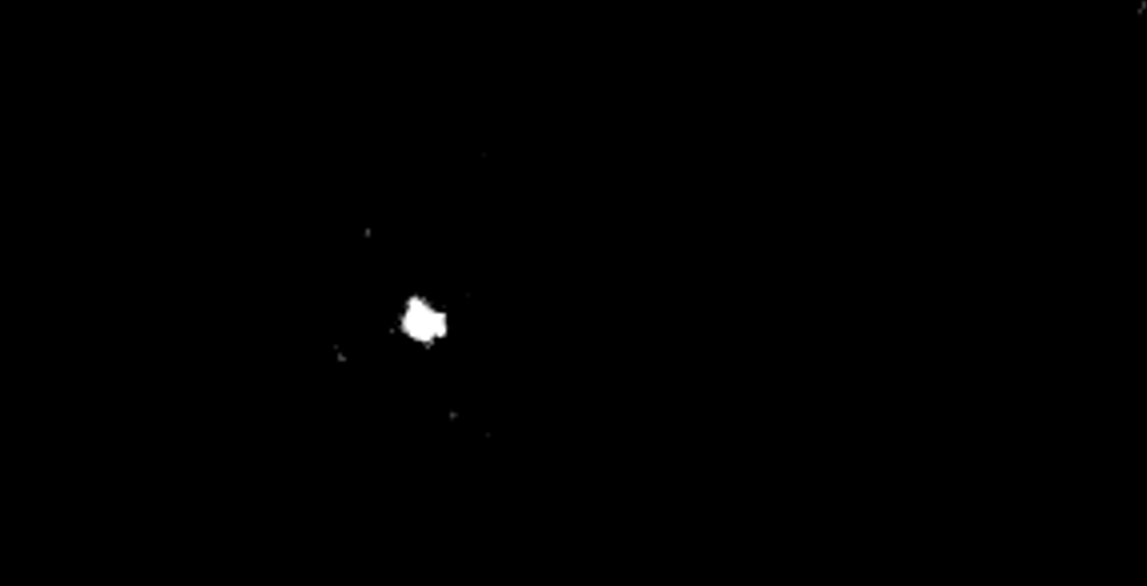

Supplement: Supplementary file 6 — Source data Fig. 4 [file 44319_2026_831_MOESM6_ESM.zip › Figure 4A/Ramos WT_PCNT.tif]

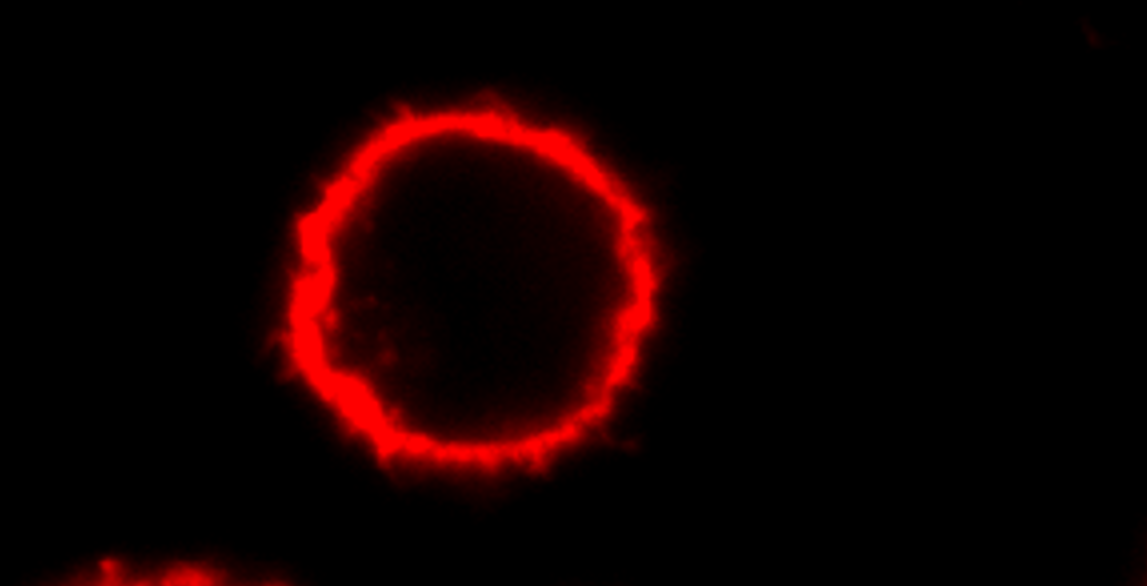

Supplement: Supplementary file 6 — Source data Fig. 4 [file 44319_2026_831_MOESM6_ESM.zip › Figure 4A/Ramos WT_Phalloidin.tif]

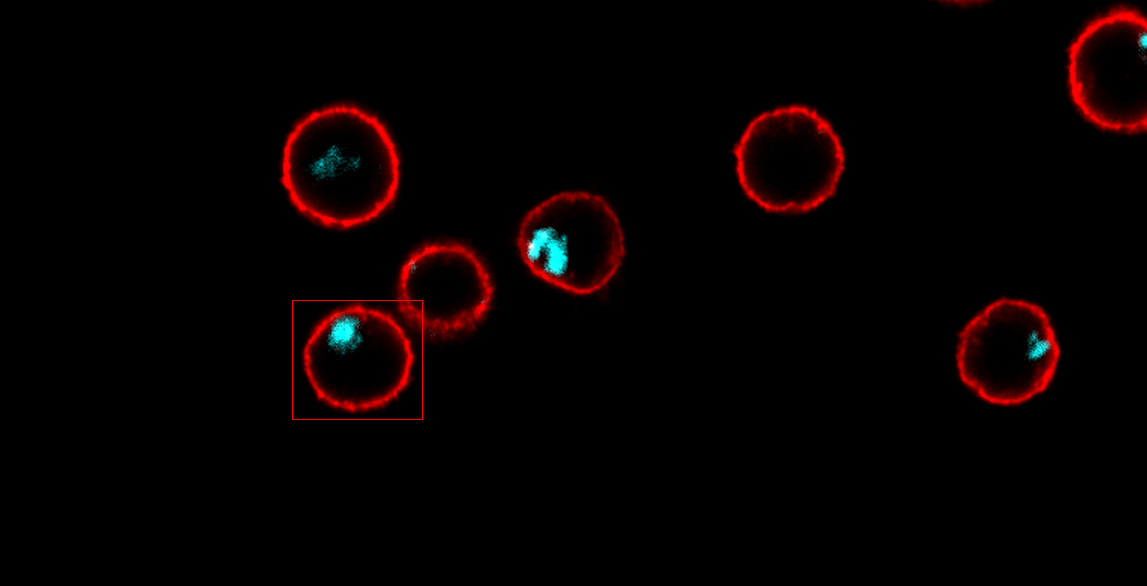

Supplement: Supplementary file 6 — Source data Fig. 4 [file 44319_2026_831_MOESM6_ESM.zip › Figure 4C/Compact_Lamp1.tif]

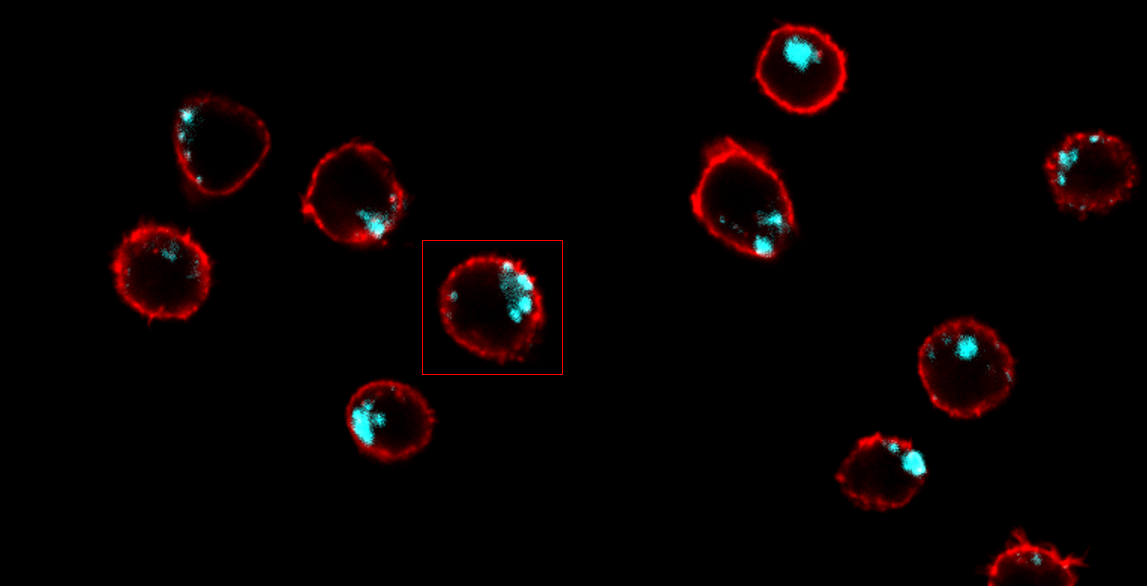

Supplement: Supplementary file 6 — Source data Fig. 4 [file 44319_2026_831_MOESM6_ESM.zip › Figure 4C/Dispersed_Lamp1.tif]

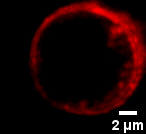

Supplement: Supplementary file 6 — Source data Fig. 4 [file 44319_2026_831_MOESM6_ESM.zip › Figure 4D/Ramos LRBA-KO_FastAct.png]

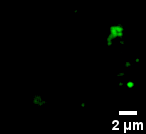

Supplement: Supplementary file 6 — Source data Fig. 4 [file 44319_2026_831_MOESM6_ESM.zip › Figure 4D/Ramos LRBA-KO_Lysotracker.png]

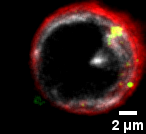

Supplement: Supplementary file 6 — Source data Fig. 4 [file 44319_2026_831_MOESM6_ESM.zip › Figure 4D/Ramos LRBA-KO_Merge.png]

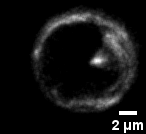

Supplement: Supplementary file 6 — Source data Fig. 4 [file 44319_2026_831_MOESM6_ESM.zip › Figure 4D/Ramos LRBA-KO_Tubulin.png]

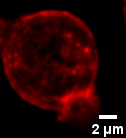

Supplement: Supplementary file 6 — Source data Fig. 4 [file 44319_2026_831_MOESM6_ESM.zip › Figure 4D/Ramos WT_FastAct.png]

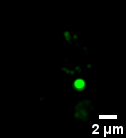

Supplement: Supplementary file 6 — Source data Fig. 4 [file 44319_2026_831_MOESM6_ESM.zip › Figure 4D/Ramos WT_Lysotrackert.png]

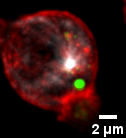

Supplement: Supplementary file 6 — Source data Fig. 4 [file 44319_2026_831_MOESM6_ESM.zip › Figure 4D/Ramos WT_Merge.png]

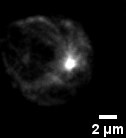

Supplement: Supplementary file 6 — Source data Fig. 4 [file 44319_2026_831_MOESM6_ESM.zip › Figure 4D/Ramos WT_Tubulin.png]

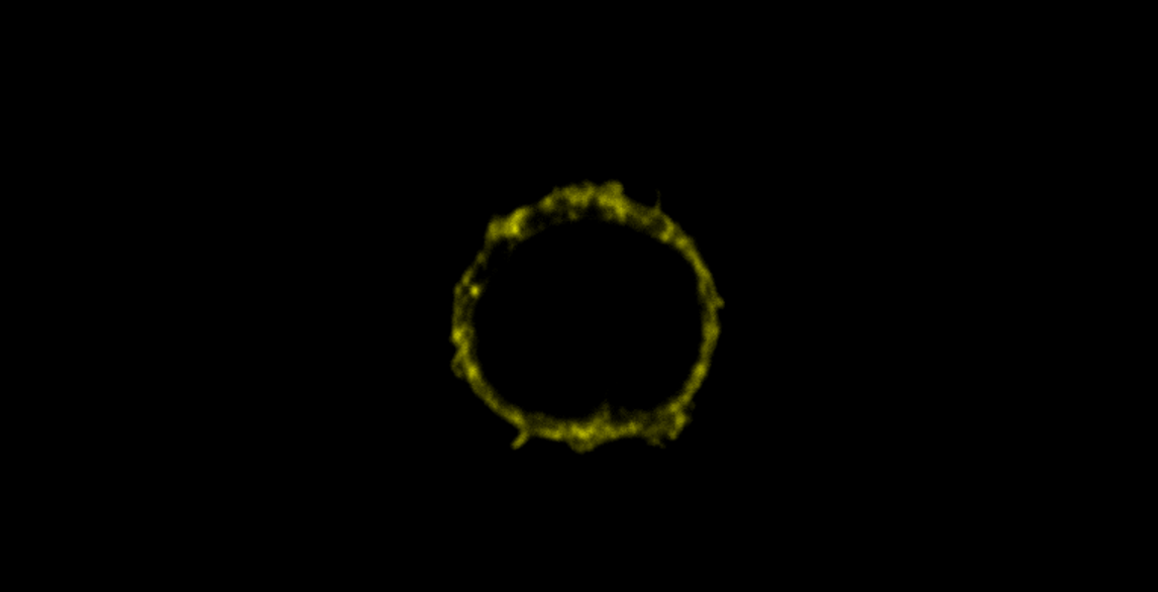

Supplement: Supplementary file 6 — Source data Fig. 4 [file 44319_2026_831_MOESM6_ESM.zip › Figure 4F/Ramos LRBA-KO_BCR Bead_BCR.tif]

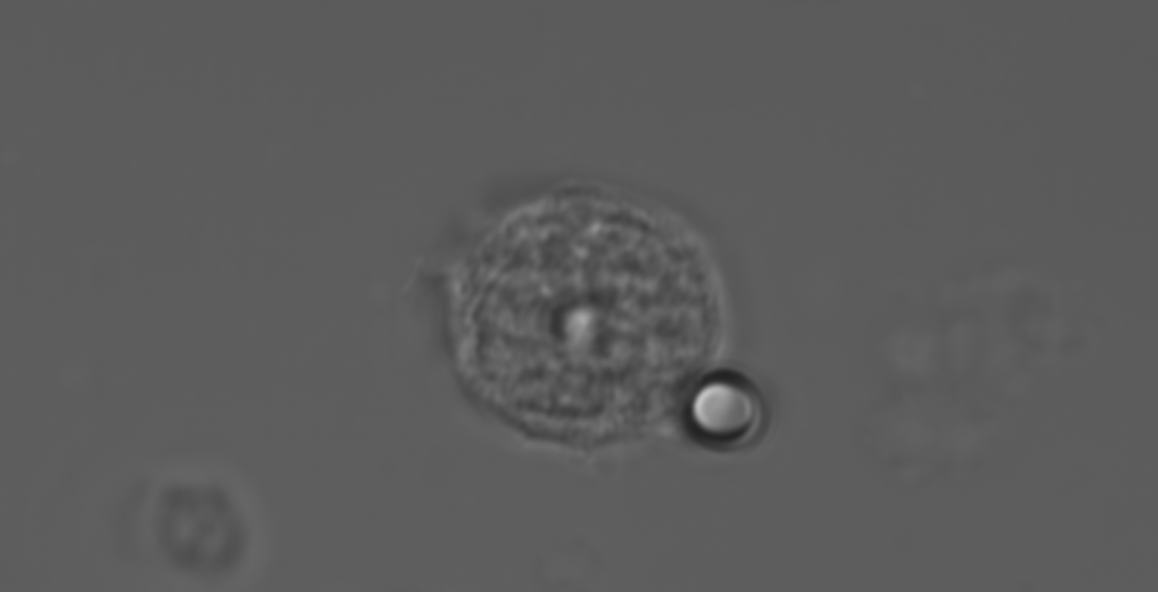

Supplement: Supplementary file 6 — Source data Fig. 4 [file 44319_2026_831_MOESM6_ESM.zip › Figure 4F/Ramos LRBA-KO_BCR Bead_TPMT.tif]

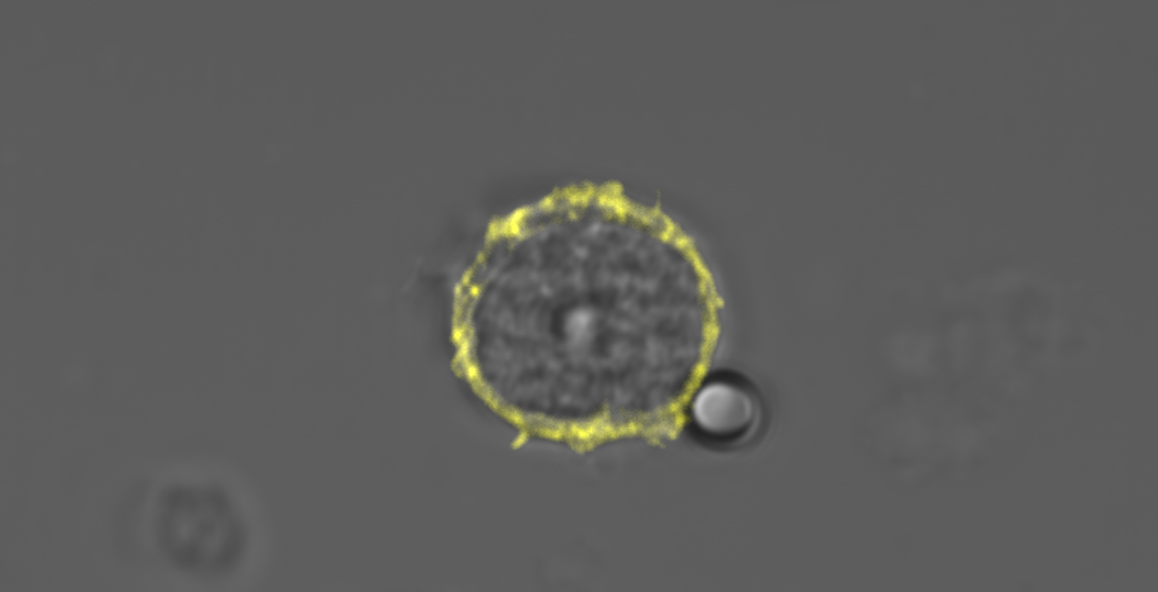

Supplement: Supplementary file 6 — Source data Fig. 4 [file 44319_2026_831_MOESM6_ESM.zip › Figure 4F/Ramos LRBA-KO_BCR Bead_TPMTBCR.tif]

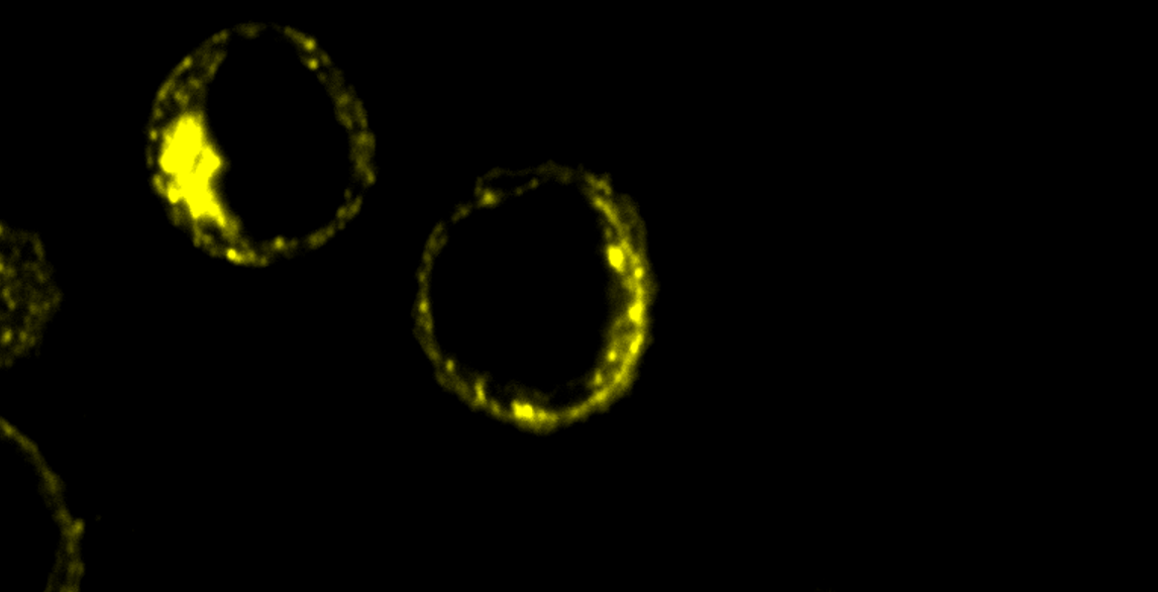

Supplement: Supplementary file 6 — Source data Fig. 4 [file 44319_2026_831_MOESM6_ESM.zip › Figure 4F/Ramos WT_BCR Bead_BCR.tif]

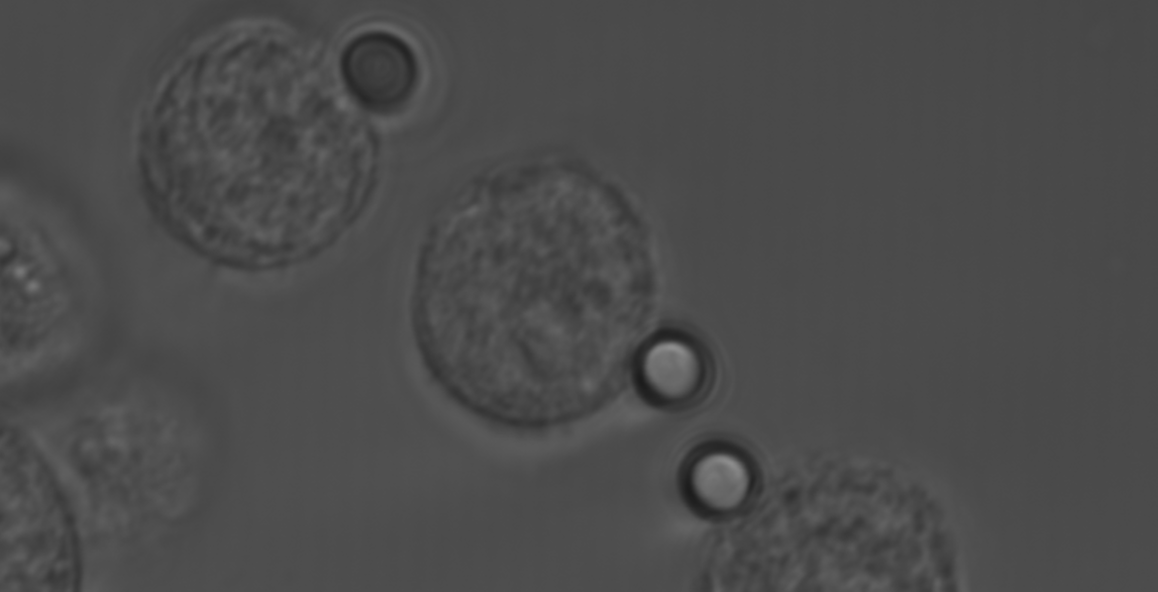

Supplement: Supplementary file 6 — Source data Fig. 4 [file 44319_2026_831_MOESM6_ESM.zip › Figure 4F/Ramos WT_BCR Bead_TPMT.tif]

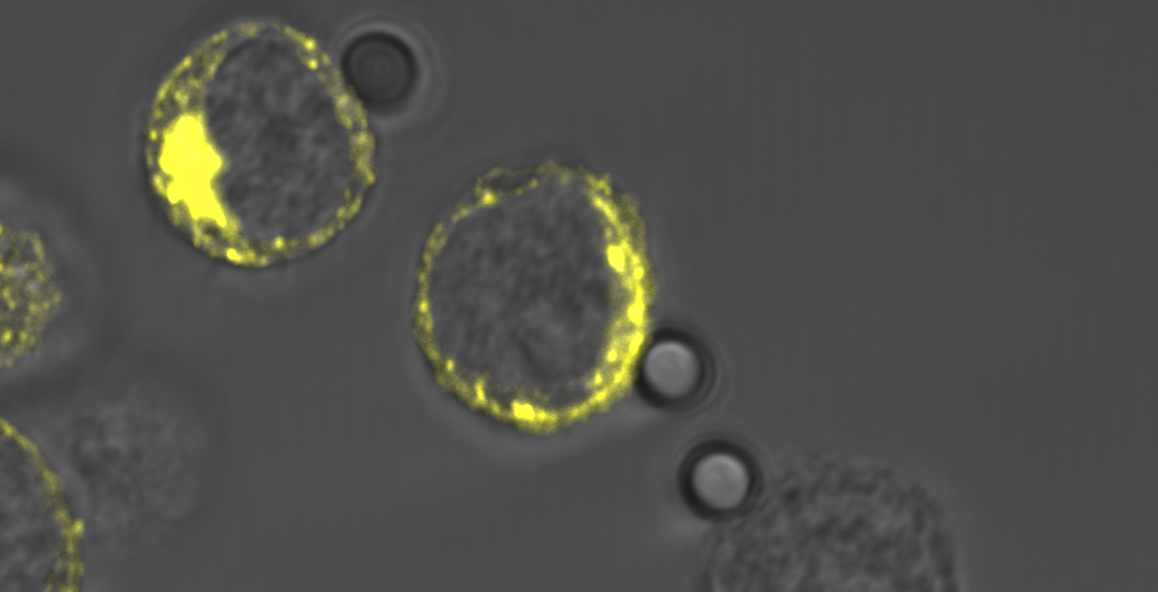

Supplement: Supplementary file 6 — Source data Fig. 4 [file 44319_2026_831_MOESM6_ESM.zip › Figure 4F/Ramos WT_BCR Bead_TPMT+BCR.tif]

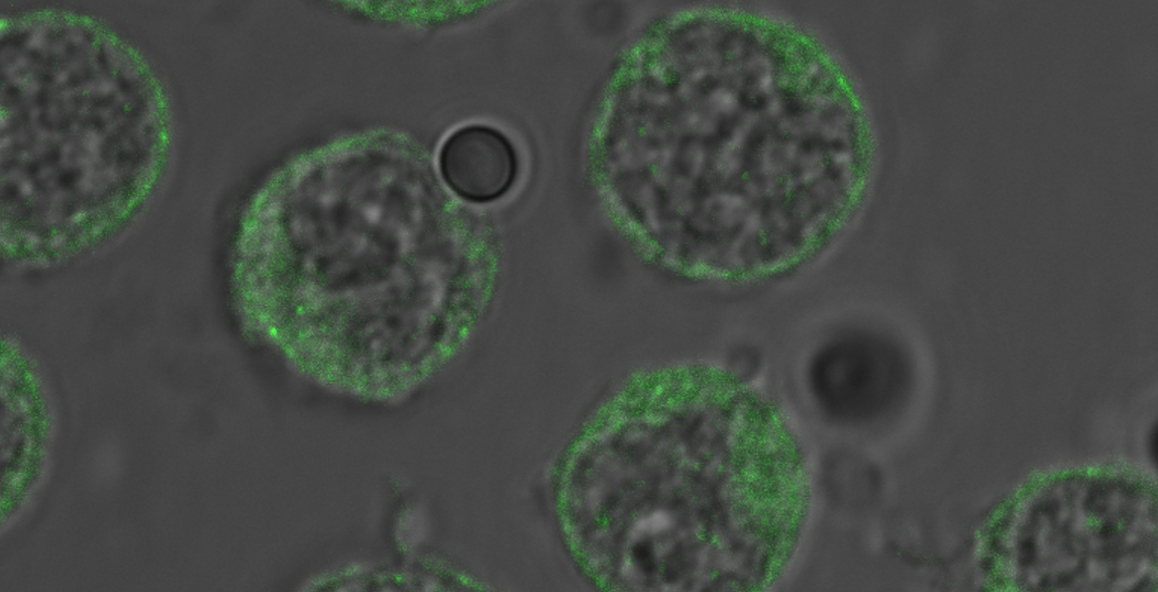

Supplement: Supplementary file 6 — Source data Fig. 4 [file 44319_2026_831_MOESM6_ESM.zip › Figure 4H/Ramos LRBA-KO_Merge.tif]

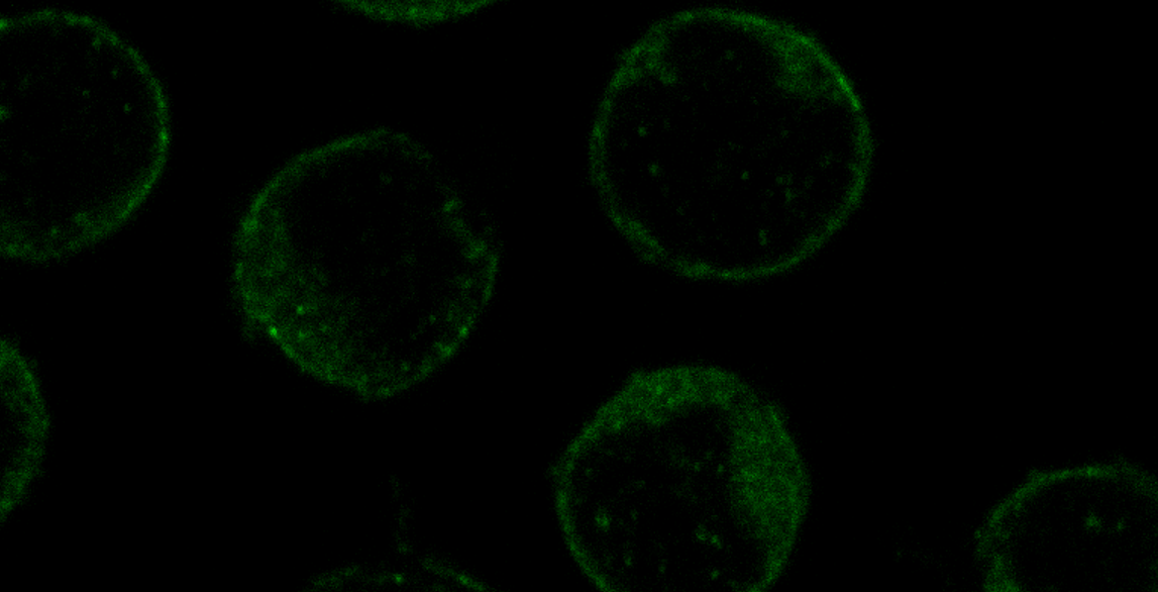

Supplement: Supplementary file 6 — Source data Fig. 4 [file 44319_2026_831_MOESM6_ESM.zip › Figure 4H/Ramos LRBA-KO_pMLC.tif]

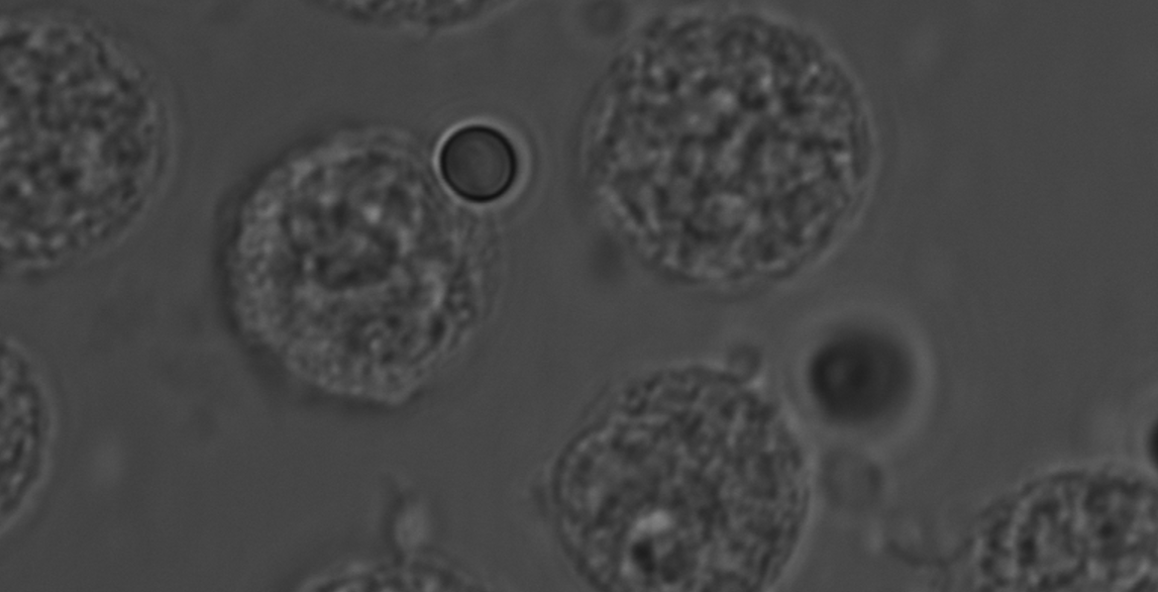

Supplement: Supplementary file 6 — Source data Fig. 4 [file 44319_2026_831_MOESM6_ESM.zip › Figure 4H/Ramos LRBA-KO_T-PMT.tif]

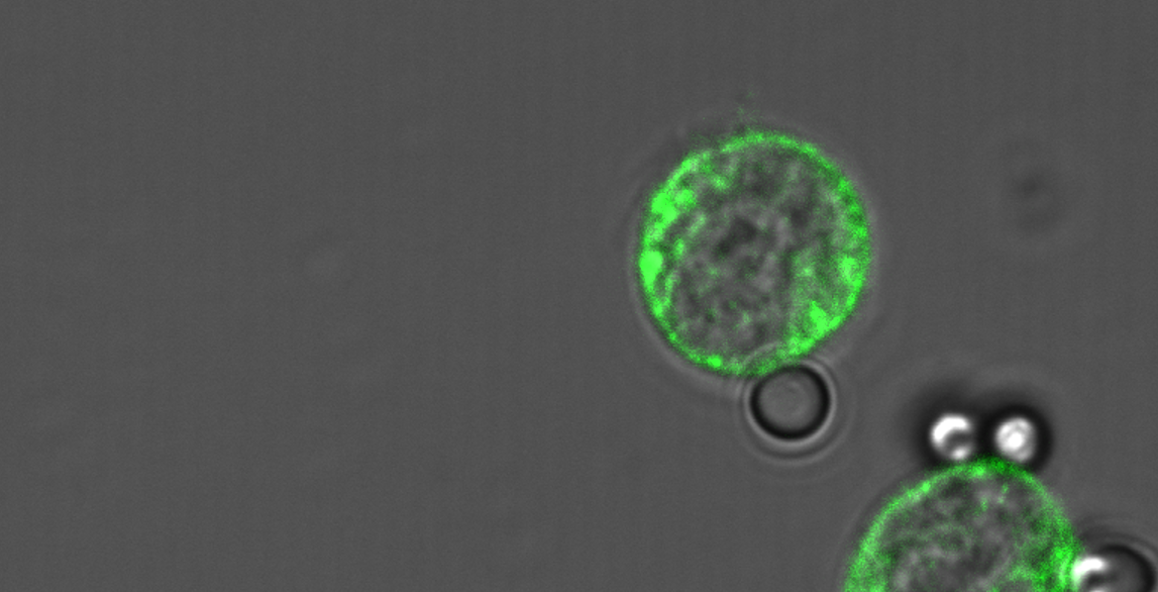

Supplement: Supplementary file 6 — Source data Fig. 4 [file 44319_2026_831_MOESM6_ESM.zip › Figure 4H/Ramos WT_Merge.tif]

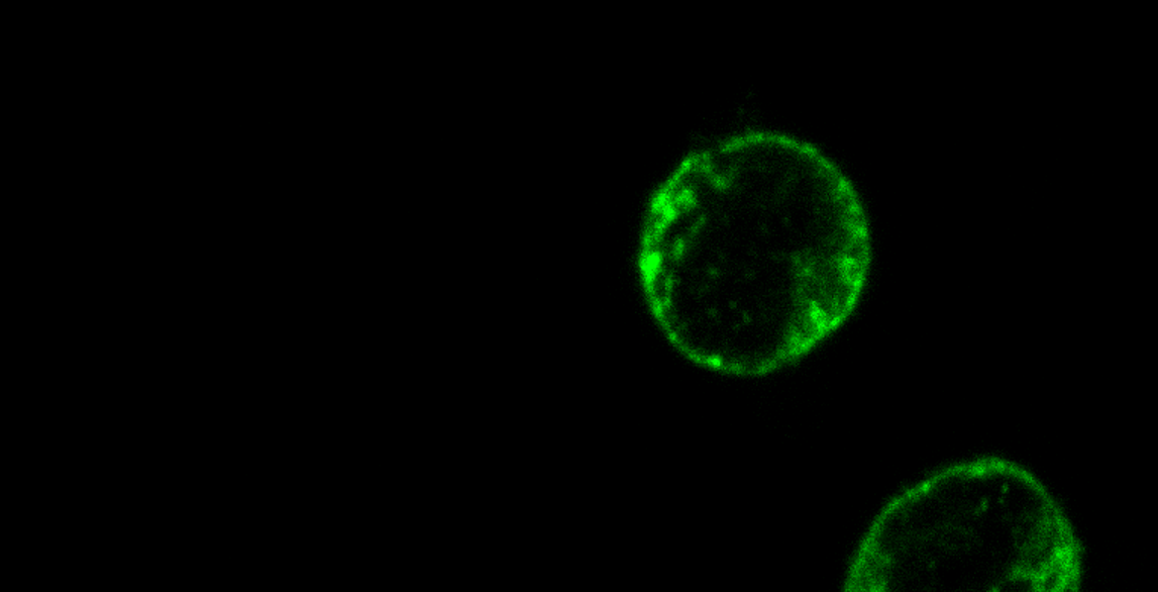

Supplement: Supplementary file 6 — Source data Fig. 4 [file 44319_2026_831_MOESM6_ESM.zip › Figure 4H/Ramos WT_pMLC.tif]

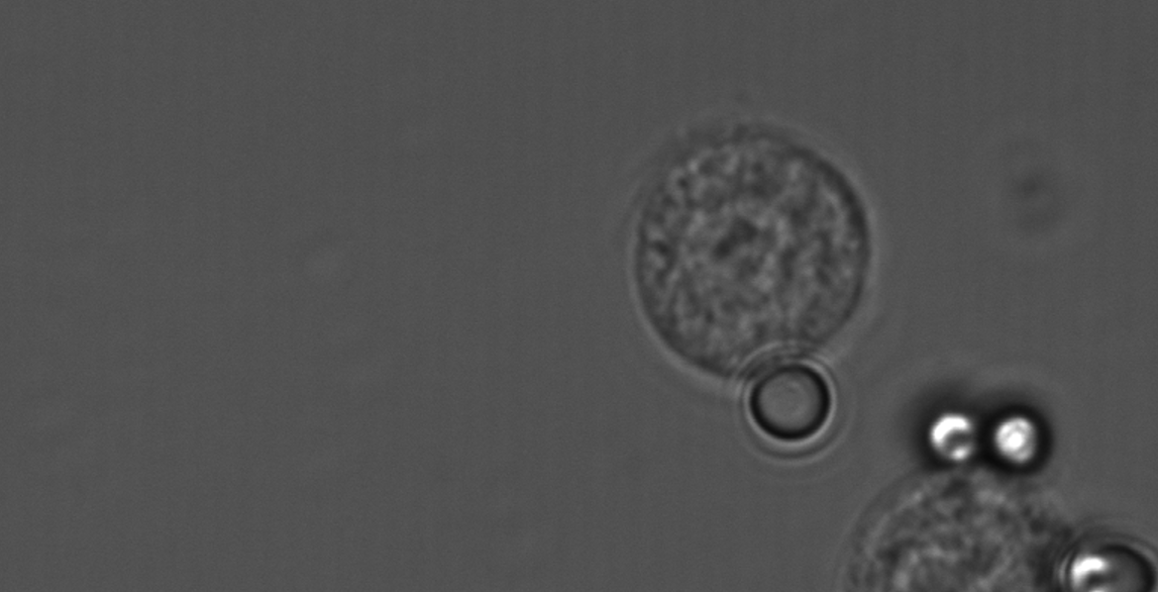

Supplement: Supplementary file 6 — Source data Fig. 4 [file 44319_2026_831_MOESM6_ESM.zip › Figure 4H/Ramos WT_T-PMT.tif]

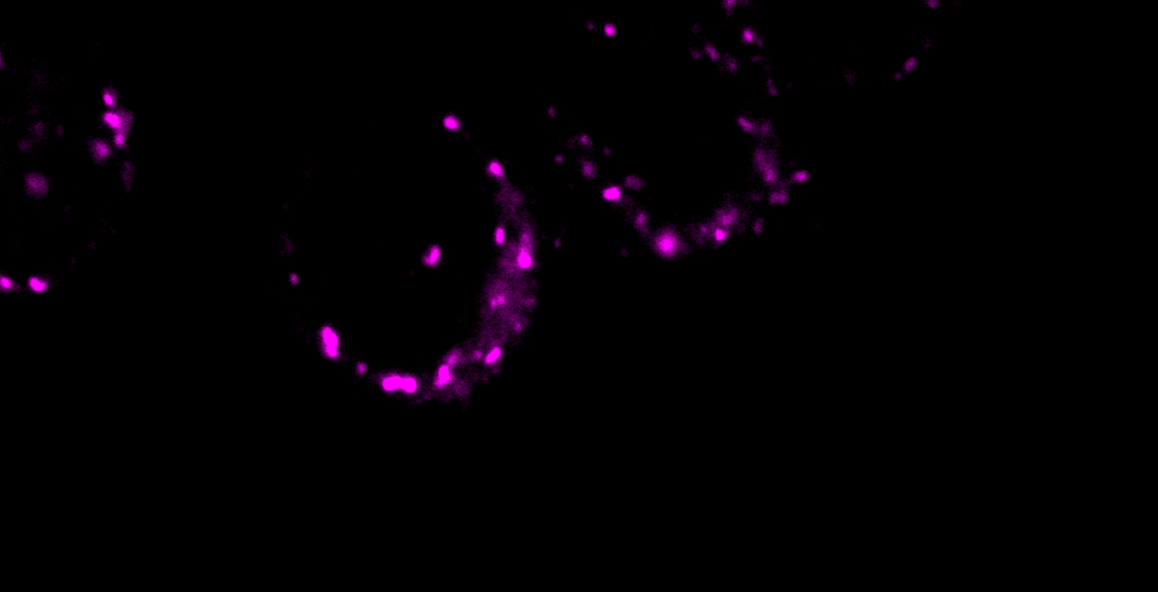

Supplement: Supplementary file 6 — Source data Fig. 4 [file 44319_2026_831_MOESM6_ESM.zip › Figure 4J/Ramos WT_LRBA_left panel.tif]

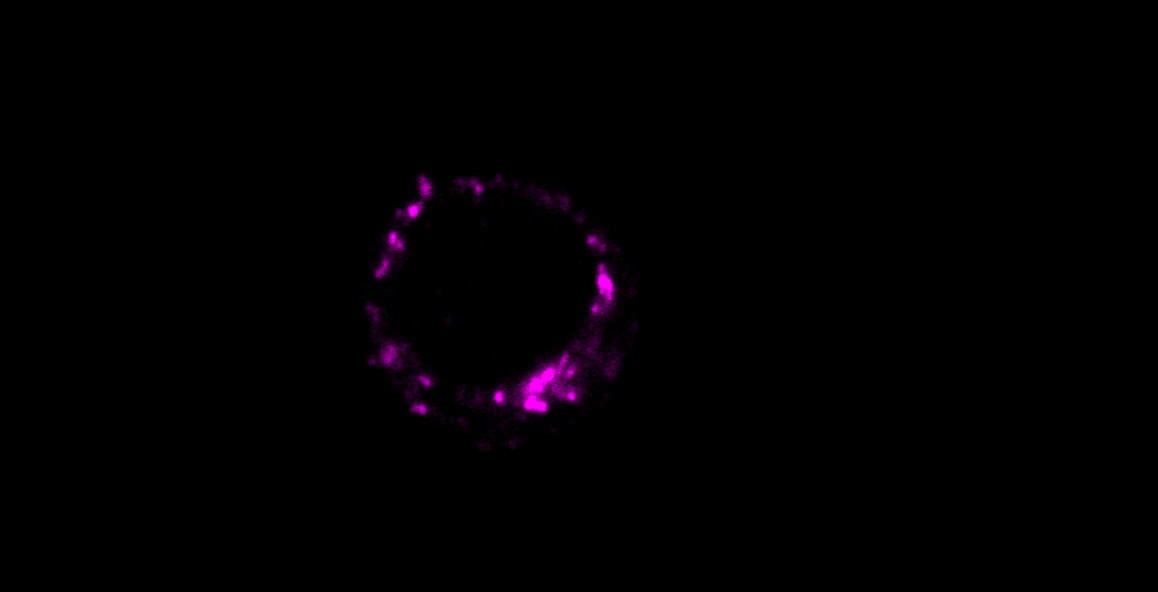

Supplement: Supplementary file 6 — Source data Fig. 4 [file 44319_2026_831_MOESM6_ESM.zip › Figure 4J/Ramos WT_LRBA_right panel.tif]

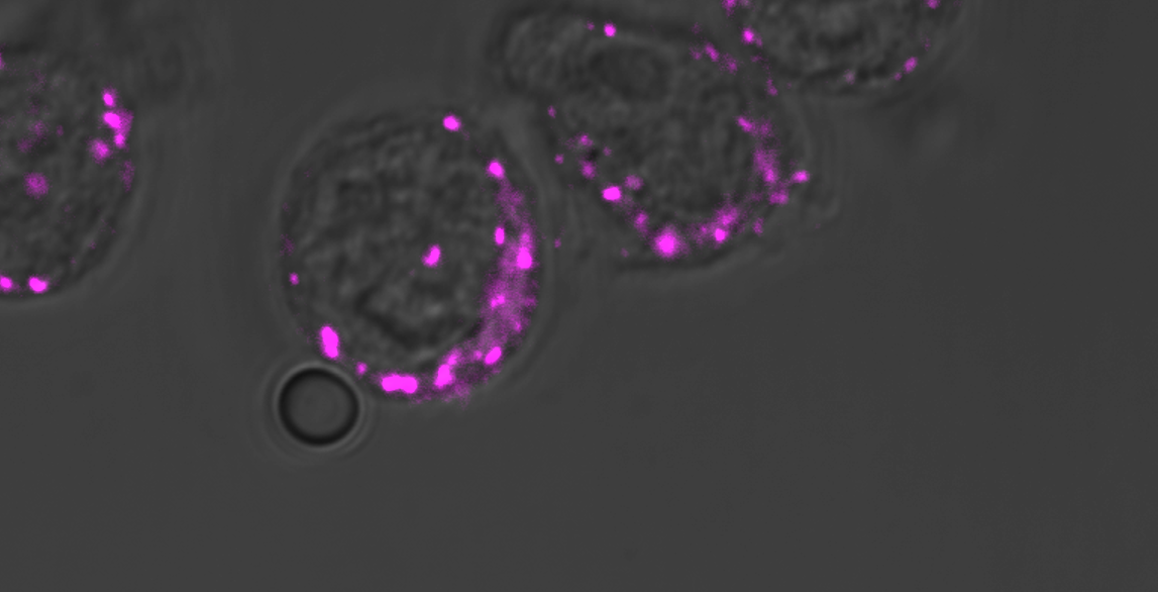

Supplement: Supplementary file 6 — Source data Fig. 4 [file 44319_2026_831_MOESM6_ESM.zip › Figure 4J/Ramos WT_LRBA+T-PMT_left panel.tif]

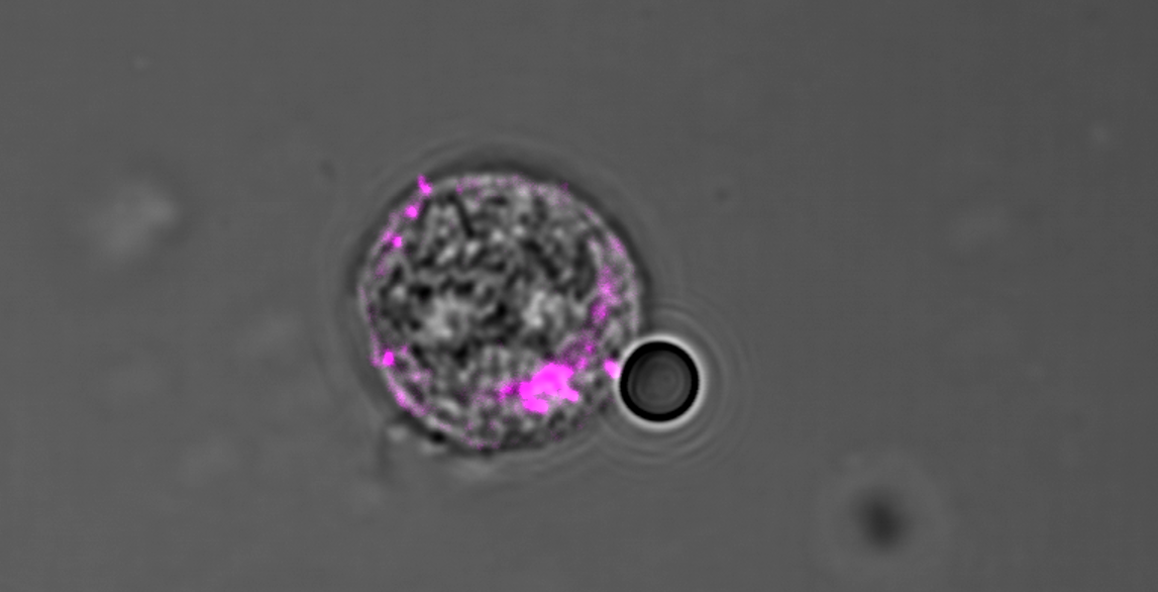

Supplement: Supplementary file 6 — Source data Fig. 4 [file 44319_2026_831_MOESM6_ESM.zip › Figure 4J/Ramos WT_LRBA+T-PMT_right panel.tif]

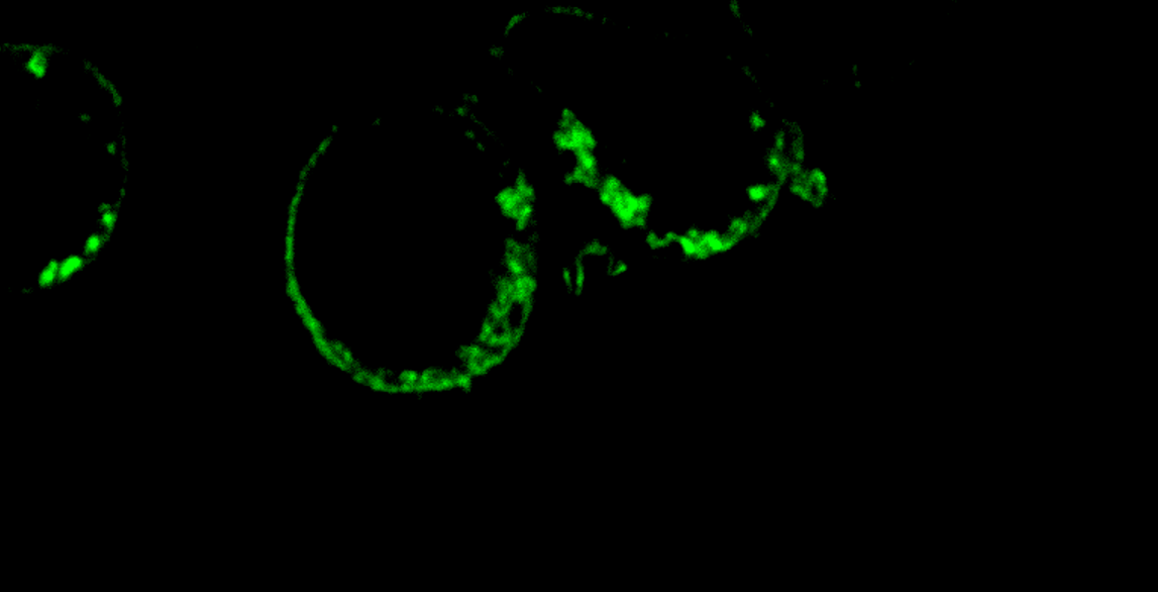

Supplement: Supplementary file 6 — Source data Fig. 4 [file 44319_2026_831_MOESM6_ESM.zip › Figure 4J/Ramos WT_MYH9_left panel.tif]

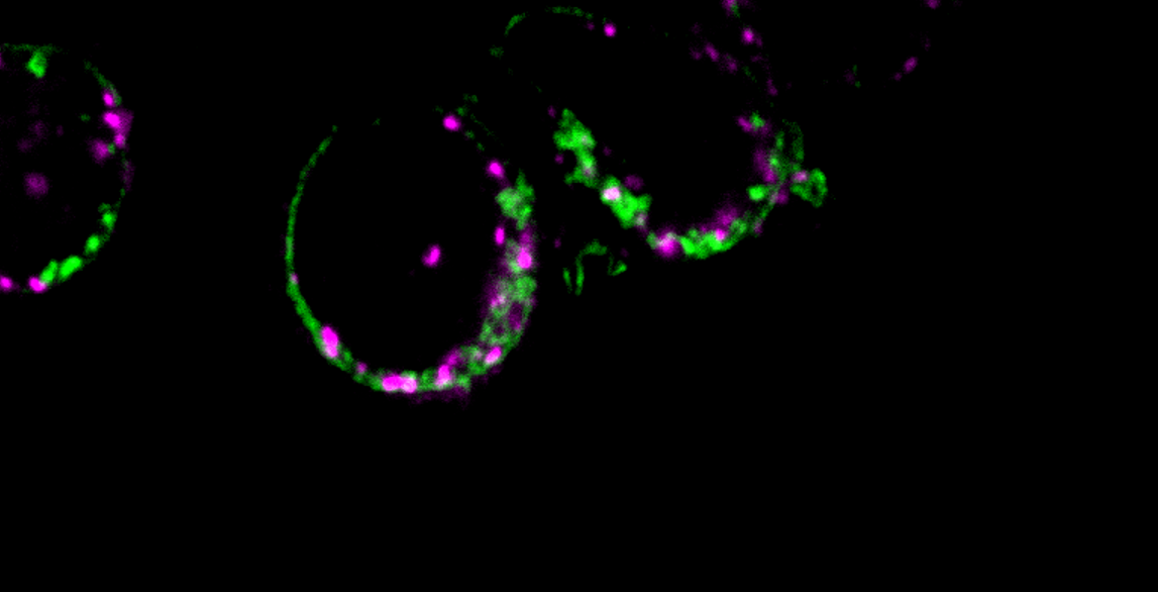

Supplement: Supplementary file 6 — Source data Fig. 4 [file 44319_2026_831_MOESM6_ESM.zip › Figure 4J/Ramos WT_MYH9+LRBA_left panel.tif]

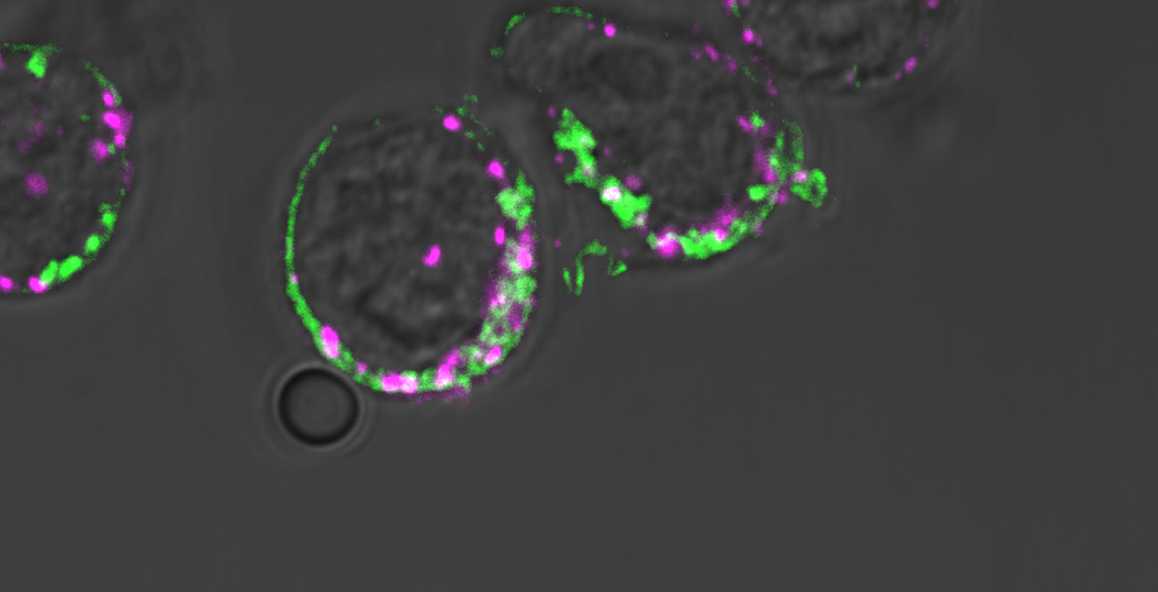

Supplement: Supplementary file 6 — Source data Fig. 4 [file 44319_2026_831_MOESM6_ESM.zip › Figure 4J/Ramos WT_MYH9+LRBA+T-PMT_left panel.tif]

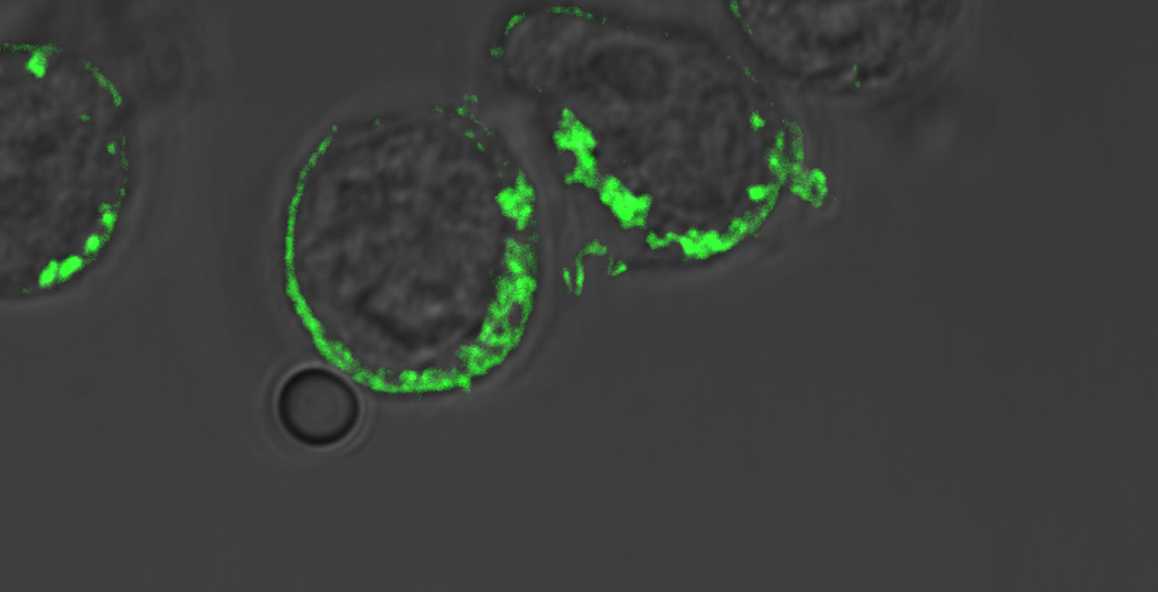

Supplement: Supplementary file 6 — Source data Fig. 4 [file 44319_2026_831_MOESM6_ESM.zip › Figure 4J/Ramos WT_MYH9+T-PMT_left panel.tif]

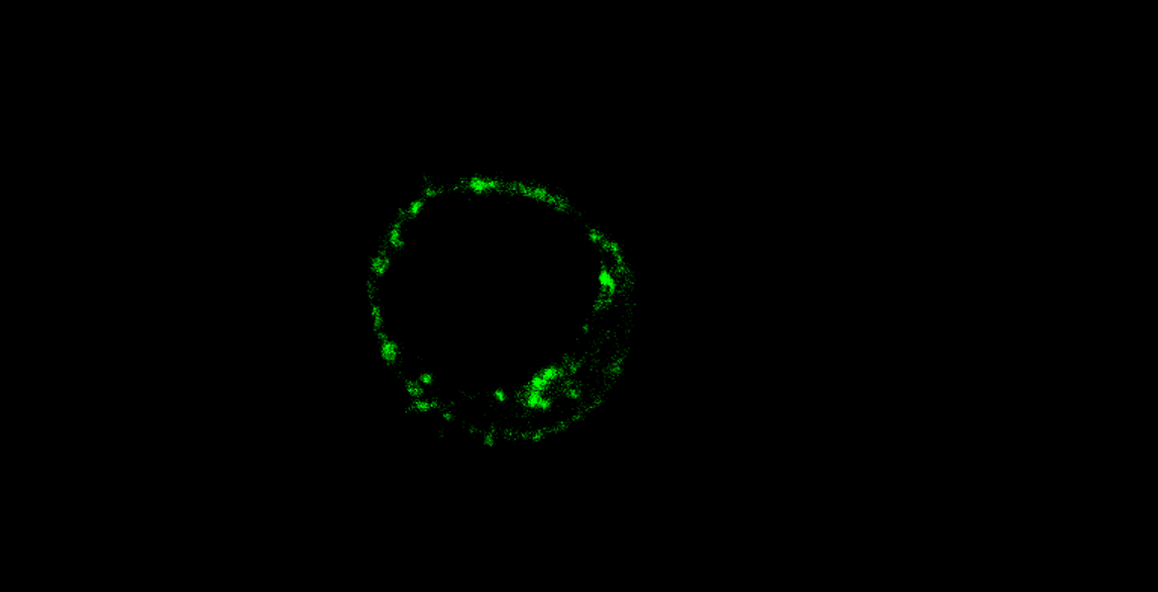

Supplement: Supplementary file 6 — Source data Fig. 4 [file 44319_2026_831_MOESM6_ESM.zip › Figure 4J/Ramos WT_pMLC_right panel.tif]

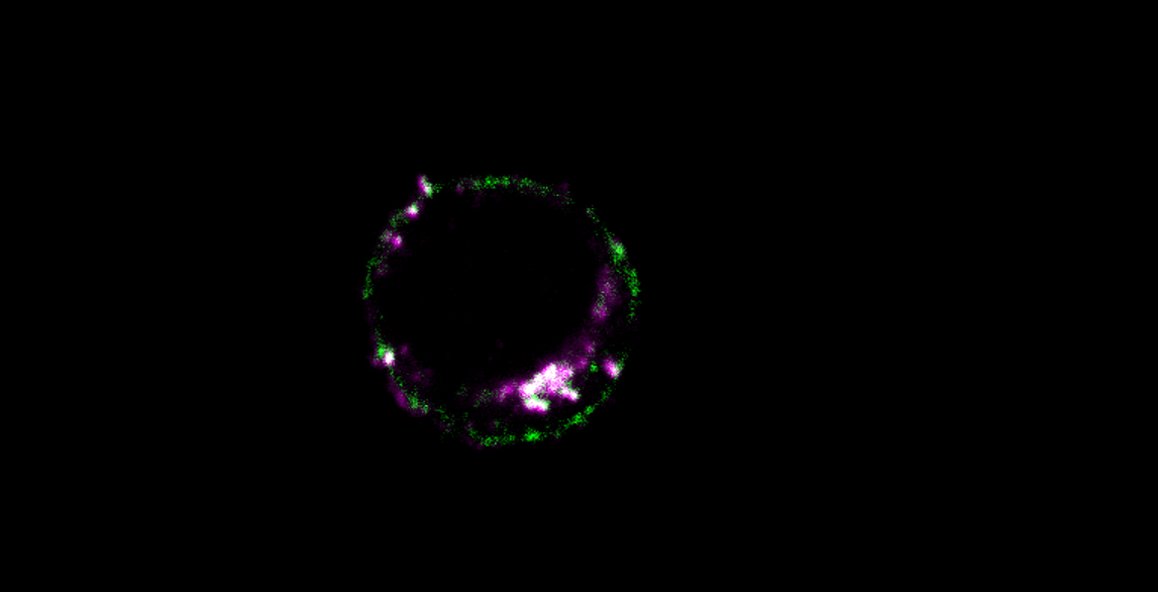

Supplement: Supplementary file 6 — Source data Fig. 4 [file 44319_2026_831_MOESM6_ESM.zip › Figure 4J/Ramos WT_pMLC+LRBA_right panel.tif]

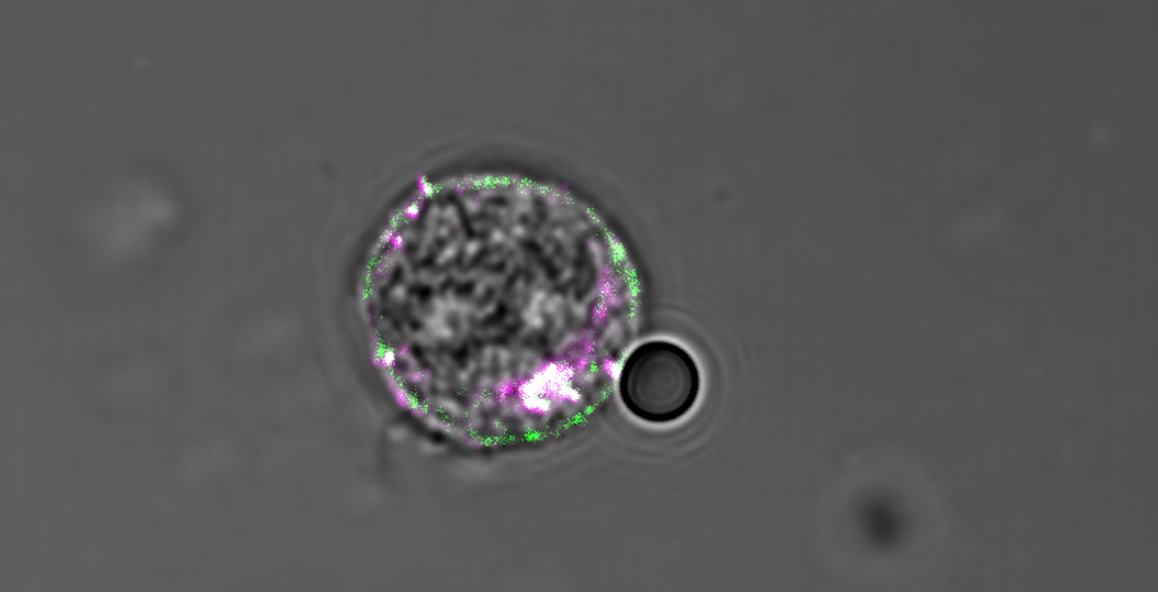

Supplement: Supplementary file 6 — Source data Fig. 4 [file 44319_2026_831_MOESM6_ESM.zip › Figure 4J/Ramos WT_pMLC+LRBA+T-PMT_right panel.tif]

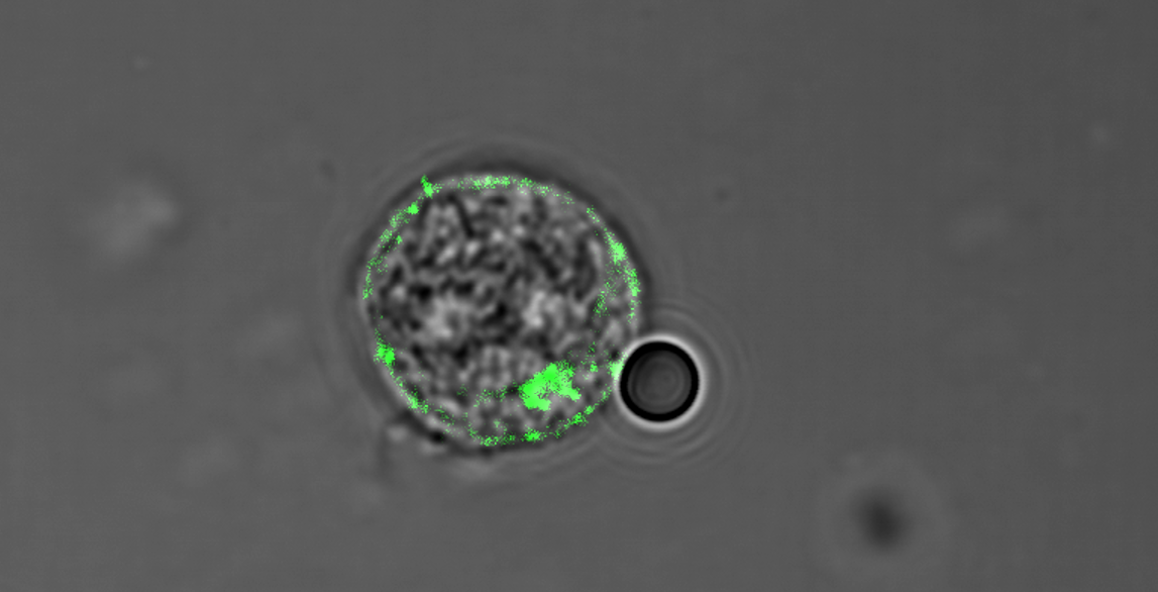

Supplement: Supplementary file 6 — Source data Fig. 4 [file 44319_2026_831_MOESM6_ESM.zip › Figure 4J/Ramos WT_pMLC+T-PMT_right panel.tif]

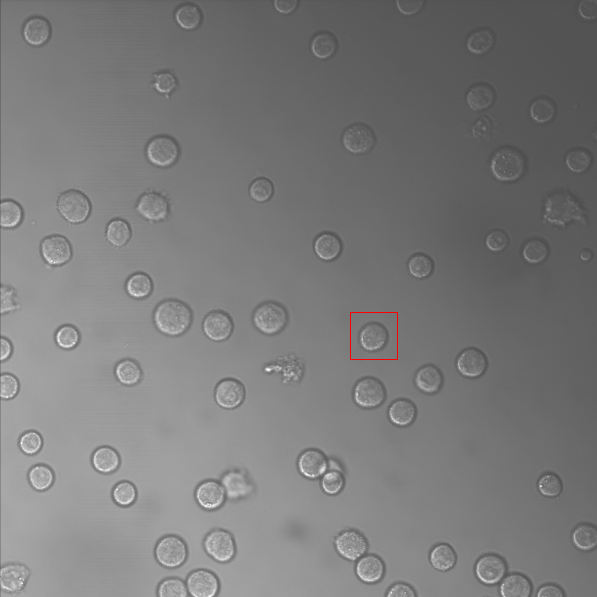

Supplement: Supplementary file 7 — Source data Fig. 5 [file 44319_2026_831_MOESM7_ESM.zip › Figure 5A/Ramos LRBA-KO_anti-IgM_DIC.tif]

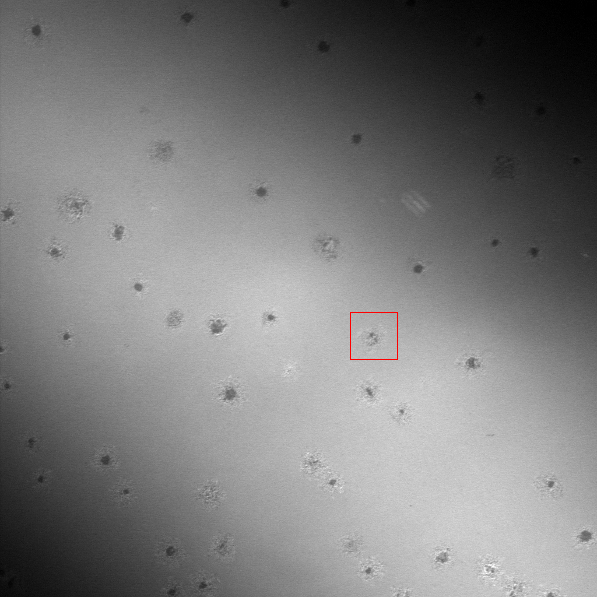

Supplement: Supplementary file 7 — Source data Fig. 5 [file 44319_2026_831_MOESM7_ESM.zip › Figure 5A/Ramos LRBA-KO_anti-IgM_IRM.tif]

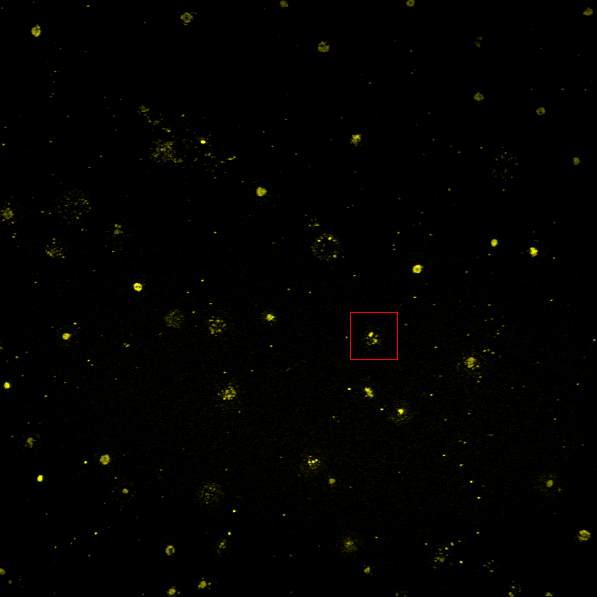

Supplement: Supplementary file 7 — Source data Fig. 5 [file 44319_2026_831_MOESM7_ESM.zip › Figure 5A/Ramos LRBA-KO_anti-IgM_su-Ag.tif]

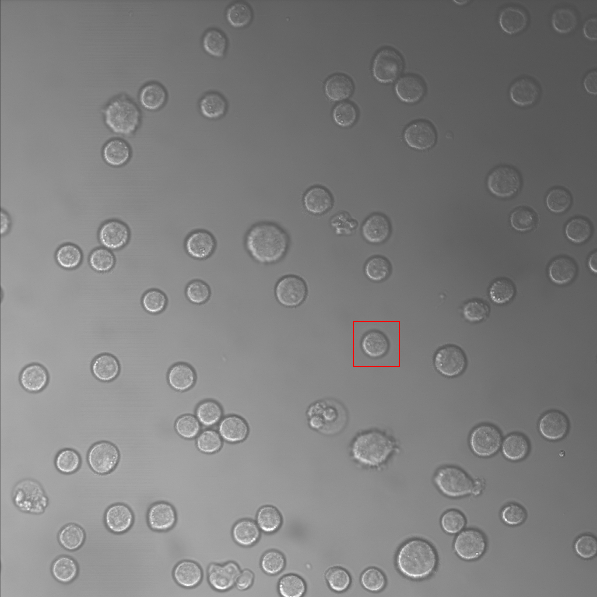

Supplement: Supplementary file 7 — Source data Fig. 5 [file 44319_2026_831_MOESM7_ESM.zip › Figure 5A/Ramos WT_anti-IgM_DIC.tif]

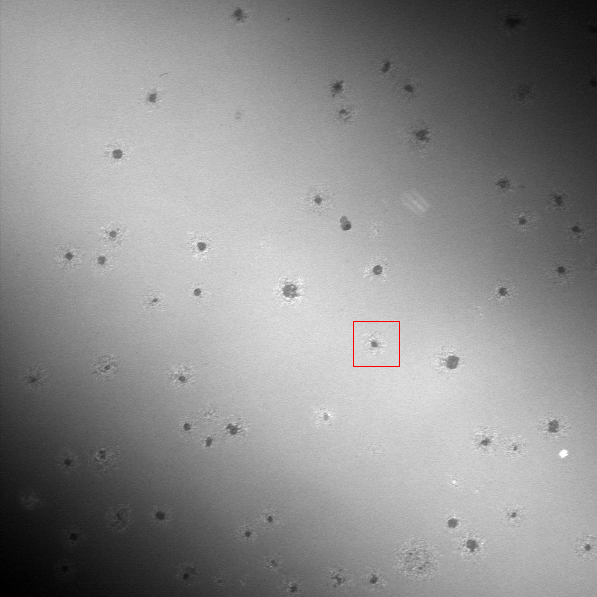

Supplement: Supplementary file 7 — Source data Fig. 5 [file 44319_2026_831_MOESM7_ESM.zip › Figure 5A/Ramos WT_anti-IgM_IRM.tif]

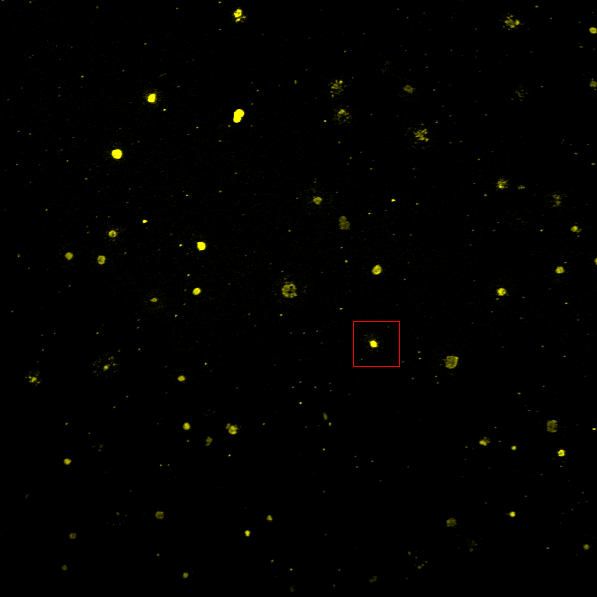

Supplement: Supplementary file 7 — Source data Fig. 5 [file 44319_2026_831_MOESM7_ESM.zip › Figure 5A/Ramos WT_anti-IgM_su-Ag.tif]

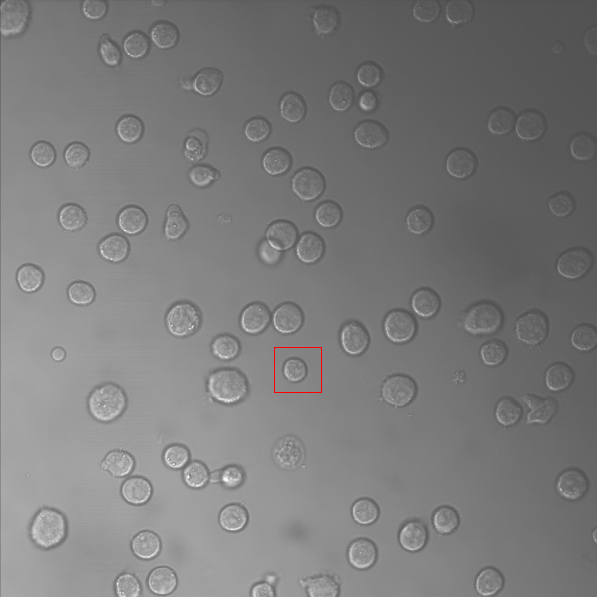

Supplement: Supplementary file 7 — Source data Fig. 5 [file 44319_2026_831_MOESM7_ESM.zip › Figure 5C/Ramos LRBA-KO_anti-IgM+VCAM-1_DIC.tif]

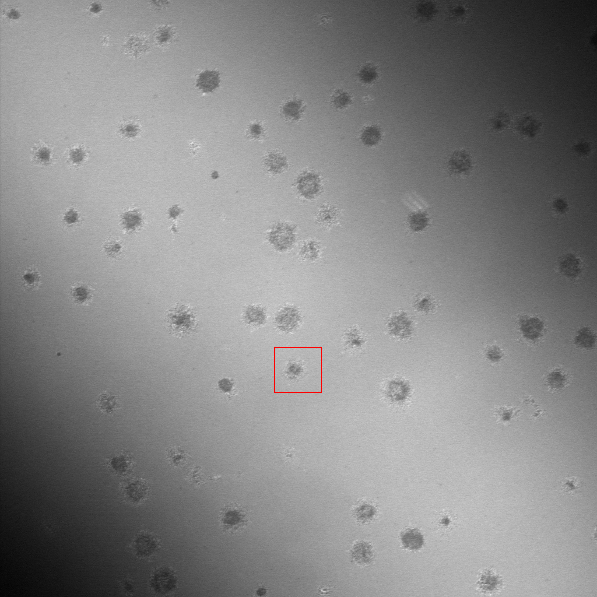

Supplement: Supplementary file 7 — Source data Fig. 5 [file 44319_2026_831_MOESM7_ESM.zip › Figure 5C/Ramos LRBA-KO_anti-IgM+VCAM-1_IRM.tif]

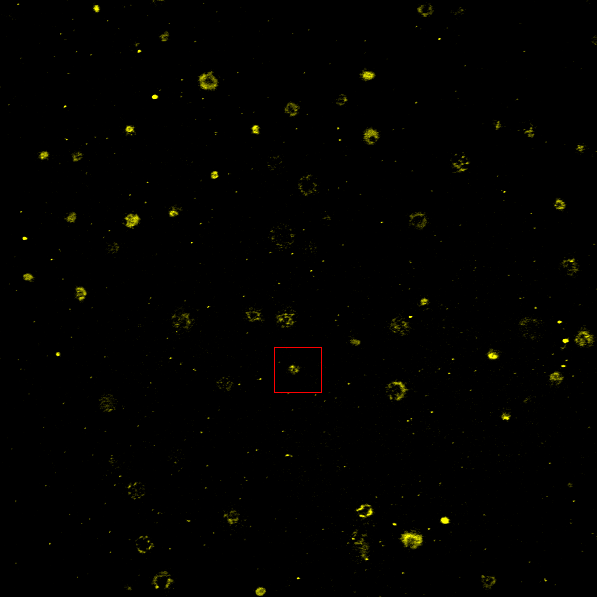

Supplement: Supplementary file 7 — Source data Fig. 5 [file 44319_2026_831_MOESM7_ESM.zip › Figure 5C/Ramos LRBA-KO_anti-IgM+VCAM-1_su-Ag.tif]

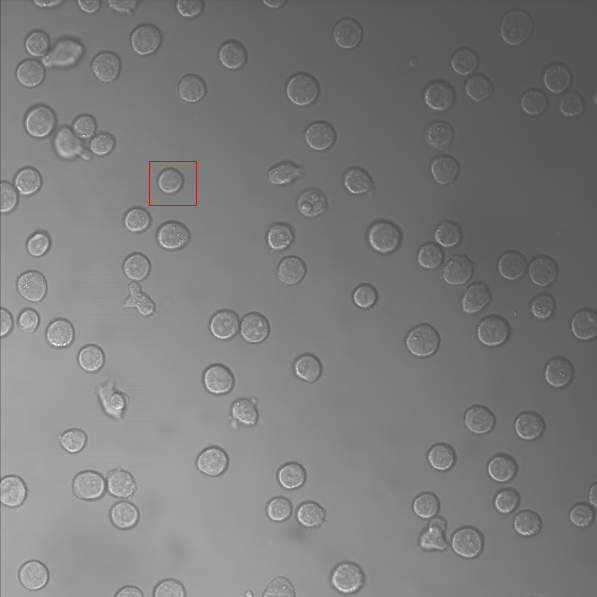

Supplement: Supplementary file 7 — Source data Fig. 5 [file 44319_2026_831_MOESM7_ESM.zip › Figure 5C/Ramos WT_anti-IgM+VCAM-1_DIC.tif]

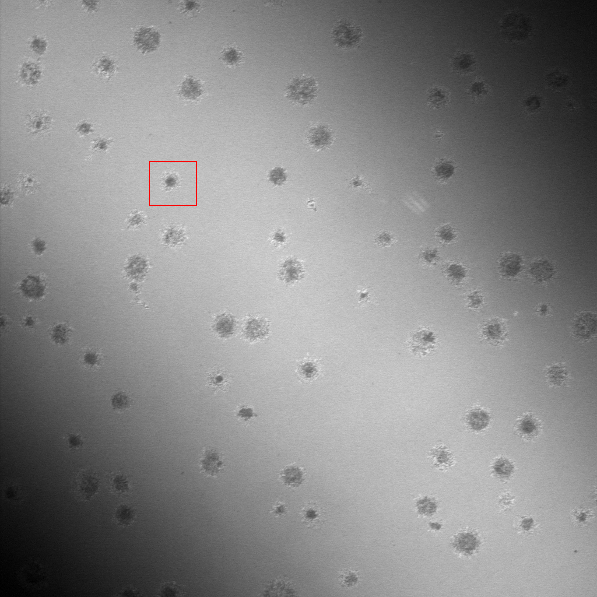

Supplement: Supplementary file 7 — Source data Fig. 5 [file 44319_2026_831_MOESM7_ESM.zip › Figure 5C/Ramos WT_anti-IgM+VCAM-1_IRM.tif]

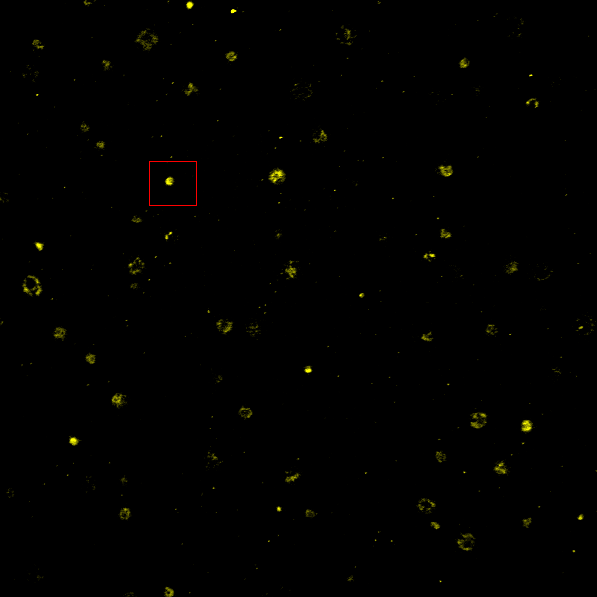

Supplement: Supplementary file 7 — Source data Fig. 5 [file 44319_2026_831_MOESM7_ESM.zip › Figure 5C/Ramos WT_anti-IgM+VCAM-1_su-Ag.tif]
