# Supplementary material for: LRBA regulates actin cytoskeleton dynamics through NMIIA during B cell immune responses
Source: EMBO Rep. 2026 Jun 12;27(14):3982–4010. doi: 10.1038/s44319-026-00831-3 (PMC13400755; doi:10.1038/s44319-026-00831-3)
Supplement: Supplementary file 8 — Expanded View Figures [file 44319_2026_831_MOESM8_ESM.pdf]

Expanded View Figures

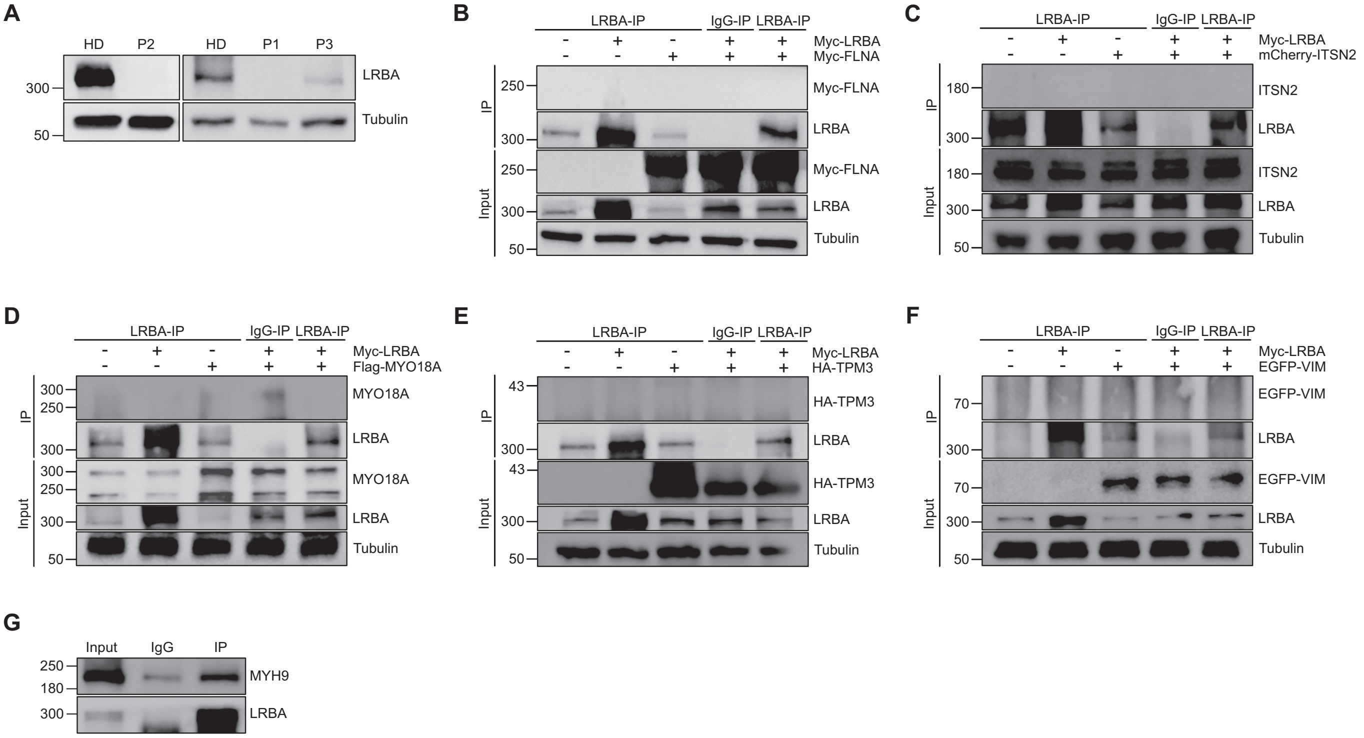

Figure EV1. LRBA does not interact with FLNA, ITSN2, MYO18A, TPM3 and VIM.

(**A**) Immunoblot analysis of LRBA protein expression in LCL cells from HD and LRBA-P1, P2 and P3. (**B–F**) WT HEK293T cells were co-transfected with Myc-LRBA plasmid and either (**B**) Myc-FLNA plasmid, (**C**) mCherry-ITSN2, (**D**) Flag-MYO18A, (**E**) HA-TPM3 or (**F**) EGFP-VIM plasmid. Immunoprecipitation was performed with anti-LRBA and immunoblotted for (**B**) FLNA using anti-Myc, (**C**) mCherry-ITSN2 using anti-ITSN2, (**D**) Flag-MYO18A using anti-MYO18A, (**E**) HA-TPM3 using anti-HA or (**F**) EGFP-VIM using anti-GFP. Tubulin was used as a loading control for the input. $n = 1$ biological replicate. (**G**) HD primary B cells were stimulated with 250 ng/ml CD40L and 10 μg/ml IL-21 for 24 h. Immunoprecipitation was performed using anti-LRBA and immunoblotted for MYH9. $n = 1$ biological replicate.

 © The Author(s)

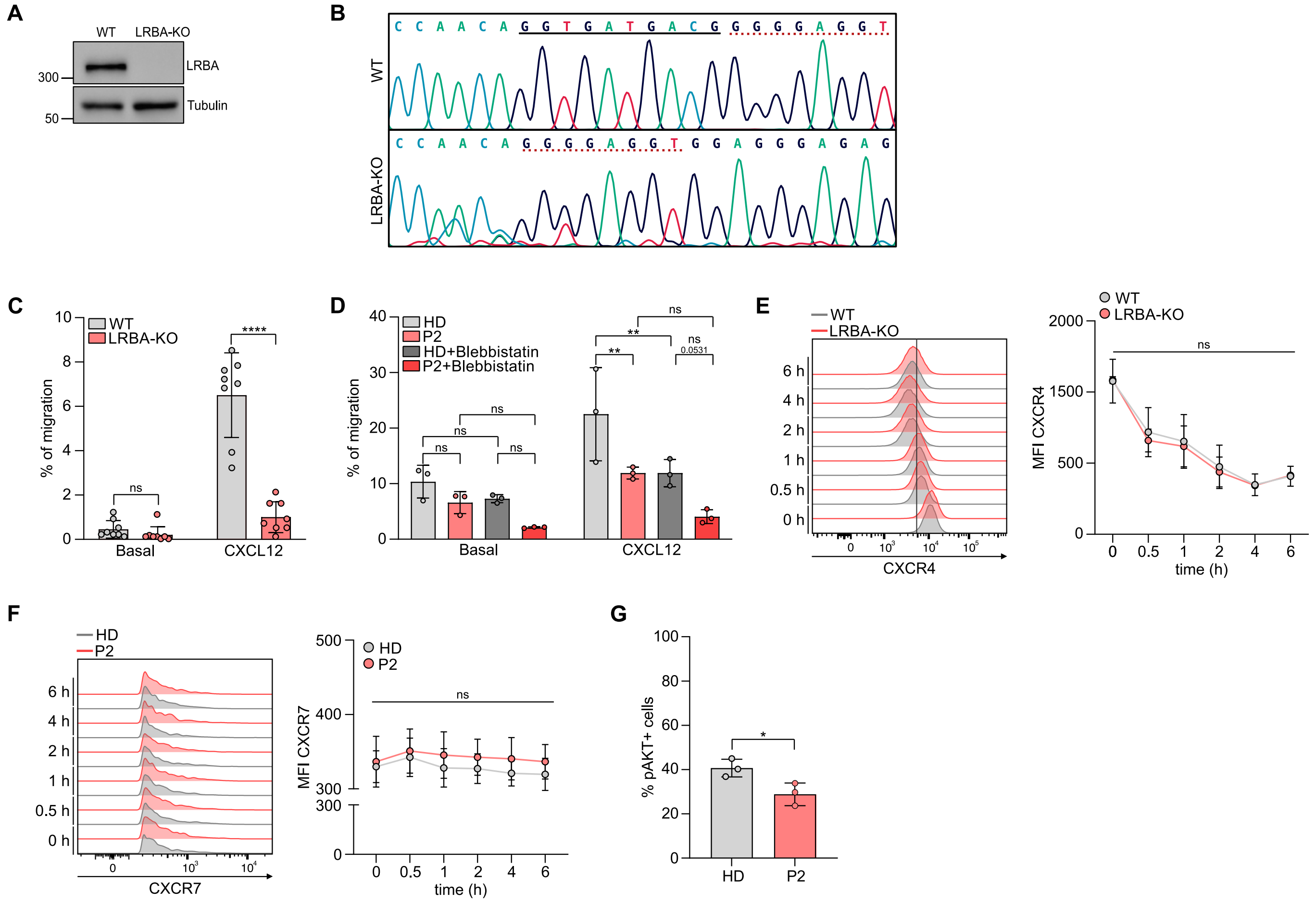

Figure EV2. Loss of LRBA does not affect CXCR4 and CXCR7 expression.

(**A**) Immunoblot analysis of LRBA protein expression in WT and LRBA-KO Ramos B cells generated using the CRISPR-Cas9 system showing absence of LRBA. (**B**) Sequencing analysis of *LRBA* exon 2 showing a deletion of 10 nucleotides in LRBA-KO cells compared to the WT sequence. (**C**) Percentage of chemokine-directed cell migration was evaluated in WT (grey) and LRBA-KO (red) Ramos B cells. Cells migrated through a 5 µm membrane towards either RPMI only (Basal) or 100 ng/ml CXCL12 for 6 h. Each dot represents one biological replicate, while bars represent mean ± SD of $n = 8$ biological replicates. (**D**) Percentage of chemokine-directed cell migration was evaluated in HD (grey) and LRBA-P2 (red) LCL cells. Cells were left untreated (light colors) or treated with 10 µM Blebbistatin (dark colors) and migrated through a 5 µm membrane towards either RPMI only (Basal) or 100 ng/ml CXCL12 for 6 h. Each dot represents one biological replicate, while bars represent mean ± SD of $n = 3$ biological replicates. (**E**) WT (grey) and LRBA-KO (red) Ramos B cells and (**F**) LCL cells from a HD (grey) and LRBA-P2 (red) were stimulated with 100 ng/ml CXCL12 for the indicated time points and analyzed for (**E**) CXCR4 and (**F**) CXCR7 expression. MFI of the total cell population is shown in (**E**) while MFI of CXCR7+ cells is shown in (**F**). Each dot represents the mean ± SEM from $n = 3$ biological replicates. (**G**) Percentage of pAKT+ HD (grey) and LRBA-P2 (red) LCL cells. Each dot represents the mean of $n = 2$ technical replicates, while bars represent mean ± SD of $n = 3$ biological replicates. Statistical analysis was performed using Welch's *t* test (**G**) or two-way ANOVA with Tukey's multiple comparison test (**C**–**F**), $^*P < 0.05$ (**G**: $P = 0.0371$) $^{**}P < 0.01$ (**D**: WT vs. KO: $P = 0.0074$, **D**: WT vs. WT-Blebbistatin: $P = 0.0074$), $^{****}P < 0.0001$, ns: not significant.

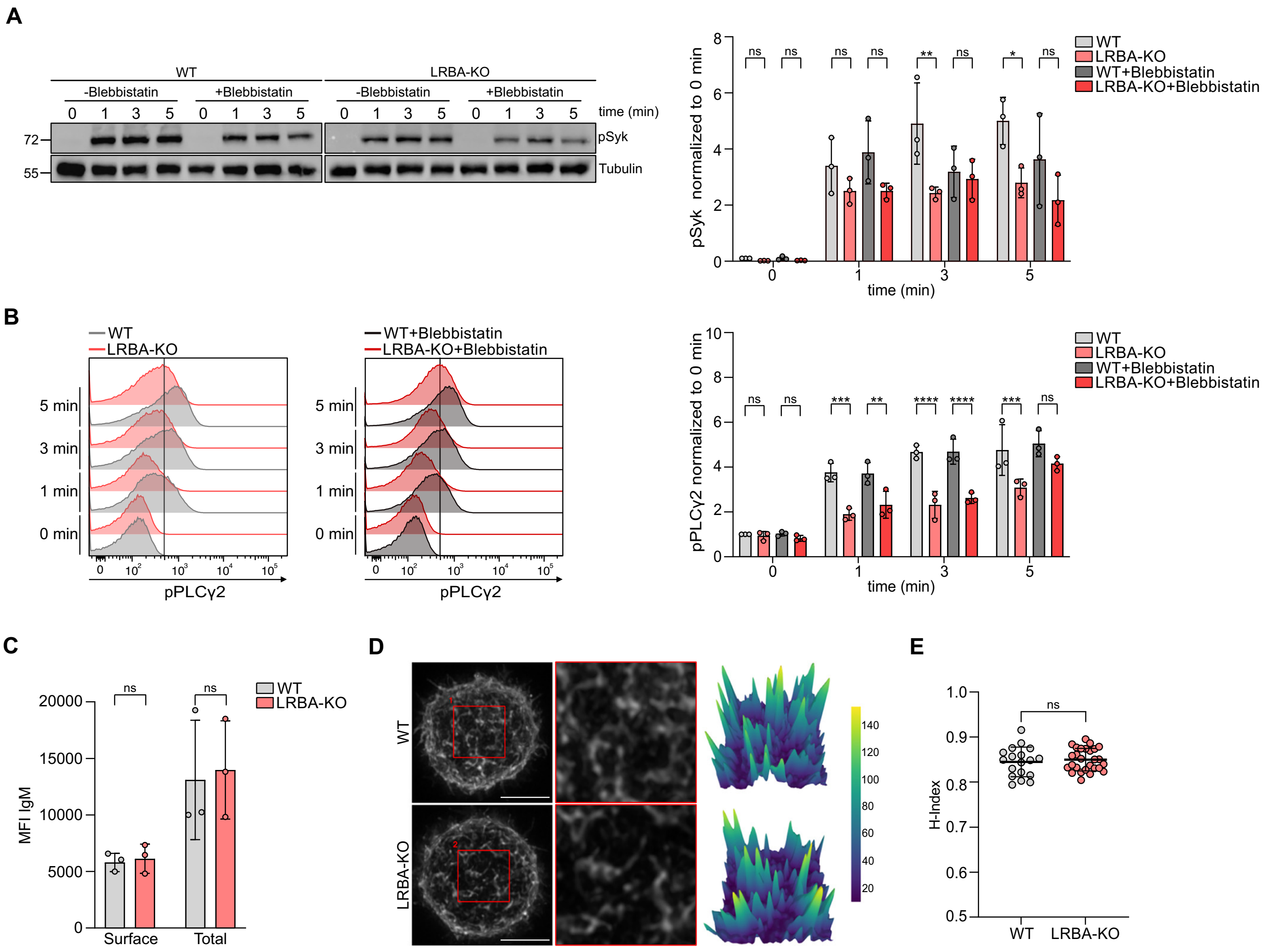

Figure EV3. Blebbistatin treatment does not alter BCR downstream signaling.

(**A**, **B**) WT (grey) and LRBA-KO (red) Ramos B cells were left untreated (light color) or treated with 50 µM Blebbistatin (dark color) for 30 min and stimulated with 5 µg/ml anti-IgM for the indicated time points. (**A**) Phosphorylation of Syk (pSyk) analyzed by western blot. Left: Representative immunoblot analyses of pSyk expression of one experiment. Tubulin was used as loading control. Right: Densitometry analyses of pSyk immunoblots normalized first to Tubulin and to then pSyk WT 0 min. (**B**) Phosphorylation of PLCy2 (pPLCy2) analyzed by flow cytometry. Left: Representative histograms of pPLCy2 MFI of one experiment. Right: Analysis of MFI normalized to pPLCy2 WT 0 min. Each dot represents one biological replicate, while bars represent mean ± SD of $n = 3$ biological replicates. (**C**) Surface and intracellular expression of IgM in WT (grey) and LRBA-KO (red) Ramos B cells. Each dot represents the mean of $n = 2$ technical replicates, while bars represent mean ± SD of $n = 3$ biological replicates. (**D**, **E**) WT and LRBA-KO Ramos B cells were stained with AF647-conjugated anti-IgM Fab fragments, fixed and imaged using confocal microscopy. (**D**) Representative images of BCR distribution after Airyscan processing and maximum intensity projection across the acquired z-stacks (left). Region of interest (ROI) in red square shown as a 2D image (center) and 3D surface plot generated from the ROI with the color-coded *z* axis representing the intensity values. Scale bar: 5 µm. (**E**) Quantification of the distribution of the BCR by the Hopkins (H) index in WT (grey) and LRBA-KO (red) Ramos B cells. Each dot represents one cell (WT: $n = 18$, KO: $n = 27$) from $n = 3$ biological replicates, mean ± SD shown by the black line and error bars. Statistical analysis of all experiments was performed using Welch's *t* test (**C**, **E**) or two-way ANOVA with Tukey's multiple comparisons test (**A**, **B**), $^{*}P < 0.05$ (**A**: $P = 0.011$), $^{**}P < 0.01$ (**A**: $P = 0.0038$, **B**: $P = 0.0057$), $^{***}P < 0.001$ (**B**, 1 min: $P = 0.0002$, **B**, 5 min: $P = 0.0007$), $^{****}P < 0.0001$. All other comparisons are ns: not significant.

 © The Author(s)

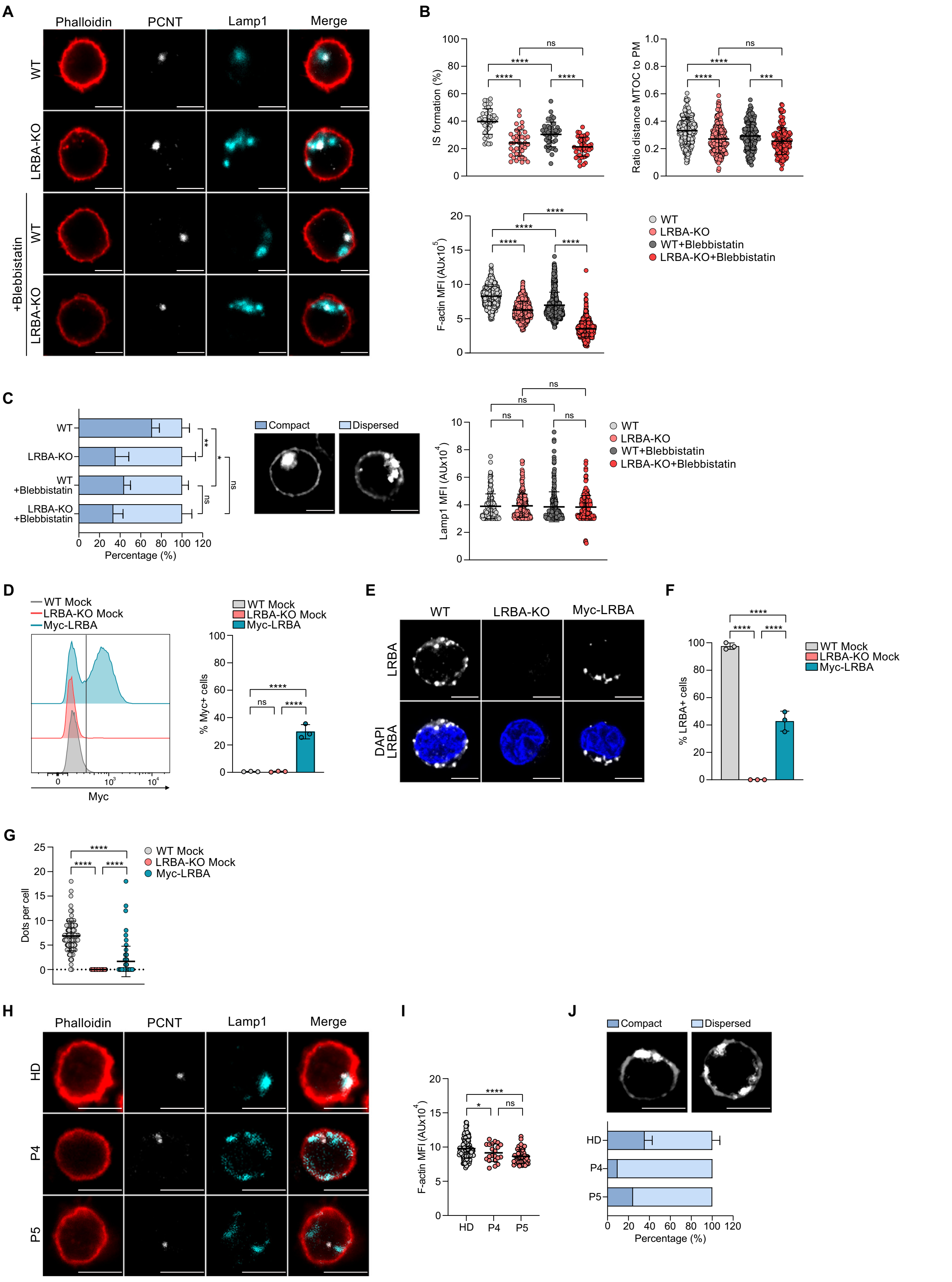
A
Phalloidin
PCNT
Lamp1
Merge
WT
LRBA-KO
+Blebbistatin
WT
LRBA-KO
B
IS formation (%)
Ratio distance MTOC to PM
F-actin MFI (AUx10^5)
ns
****
***
WT
LRBA-KO
WT+Blebbistatin
LRBA-KO+Blebbistatin
C
WT
LRBA-KO
WT +Blebbistatin
LRBA-KO +Blebbistatin
Percentage (%)
Compact
Dispersed
Lamp1 MFI (AUx10^4)
D
WT Mock
LRBA-KO Mock
Myc-LRBA
Myc
% Myc+ cells
E
WT
LRBA-KO
Myc-LRBA
LRBA
DAPI LRBA
F
% LRBA+ cells
G
Dots per cell
H
Phalloidin
PCNT
Lamp1
Merge
HD
P4
P5
I
F-actin MFI (AUx10^4)
HD
P4
P5
J
Compact
Dispersed
Percentage (%)

© The Author(s)

Figure EV4. LRBA-deficient cells show abnormal IS formation which is mimicked by treating WT cells with Blebbistatin.

(**A–C**) IS analysis of WT (grey) and LRBA-KO (red) Ramos B cells untreated or treated with 50 μM Blebbistatin for 30 min and incubated on glass slides coated with 15 μg/ml anti-IgM for 15 min. (**A**) Representative confocal microscopy images of IS formation in Ramos B cells. F-actin was stained with Phalloidin (red), microtubule organizing center (MTOC) with anti-pericentrin (PCNT) (white), and lysosomes with anti-Lamp1 (cyan). Scale bar: 5 μm. (**B**) Analysis of IS formation frequency determined by Phalloidin+Pericentrin+Lamp1+ signal (top-left) (each dot represents one image, WT: $n = 38$, KO: $n = 40$, WT-Blebb: $n = 40$, KO-Blebb: $n = 40$), ratio of the distance of MTOC to the plasma membrane (PM) (top-right) (each dot represents one cell, WT: $n = 275$, KO: $n = 242$, WT-Blebb: $n = 184$, KO-Blebb: $n = 168$), and F-actin MFI (bottom-left) (each dot represents one cell, WT: $n = 591$, KO: $n = 581$, WT-Blebb: $n = 548$, KO-Blebb: $n = 508$). $n = 3$ biological replicates, mean ± SD shown by the black line and error bars. (**C**) Analysis of lysosome distribution during IS formation. Percentage of cells with compact (dark-blue) or dispersed (light-blue) lysosome distribution (left) and MFI of Lamp1+ dots (right). Each dot represents one Lamp1+ dot (MFI), while bars and lines represent the mean ± SD of 48 cells from $n = 3$ biological replicates. (**D–G**) LRBA was re-expressed in LRBA-KO Ramos B cells using Myc-tagged LRBA mRNA nucleofection. (**D**) Representative histogram and quantification of the percentage of Myc-LRBA+ cells of WT (grey), LRBA-KO (red) and Myc-LRBA (teal) Ramos B cells using anti-Myc antibody. Each dot represents one biological replicate, while bars represent the mean ± SD of $n = 3$ biological replicates. (**E**) Representative images of LRBA expression in Ramos B cells stained for LRBA (white) and DAPI (blue). Scale bar: 5 μm. (**F**, **G**) Analysis of (**F**) percentage of LRBA expressing cells and (**G**) LRBA dots per cell in WT (grey), LRBA-KO (red) and Myc-LRBA (teal) Ramos B cells. In (**D**, **F**), each dot represents one biological replicate, while bars represent the mean ± SD of $n = 3$ biological replicates. In (**G**), each dot represents one cell (WT: $n = 125$, KO: $n = 165$, Myc: $n = 38$) from $n = 3$ biological replicates. (**H–J**) IS analysis of naive B cells from healthy donors (HD) and LRBA-P4 and P5. Cells were incubated on glass slides coated with 15 μg/ml anti-IgM for 15 min. (**H**) Representative confocal microscopy images of IS formation in naive B cells. F-actin was stained with Phalloidin (red), MTOC with anti-pericentrin (PCNT) (white), and lysosomes with anti-Lamp1 (cyan). Scale bar: 5 μm. (**I**) Analysis of F-actin MFI of HD (grey) and LRBA-P4 and P5 (red) of cells that formed an IS. Each dot represents one cell (HD: $n = 142$ from $n = 3$ different HD, P4: $n = 21$ and P5: $n = 46$), mean ± SD shown by the black line and error bars. (**J**) Analysis of lysosome distribution during IS formation. Percentage of cells with compact (dark-blue) or dispersed (light-blue) lysosome distribution. Bars represent mean ± SD for HD ($n = 3$ different HD) and mean for LRBA-P4 and P5. AU Arbitrary Units. Statistical analysis was performed using one-way (**D**, **F**, **G**, **I**) or two-way ANOVA with Tukey's multiple comparisons test (**B**, **C**), $^*P < 0.05$ (**C**: $P = 0.0131$, **I**: $P = 0.0461$), $^{**}P < 0.01$ (**C**: $P = 0.0014$), $^{***}P < 0.001$ (**B**: $P = 0.0004$), $^{****}P < 0.0001$, ns: not significant.

 © The Author(s)

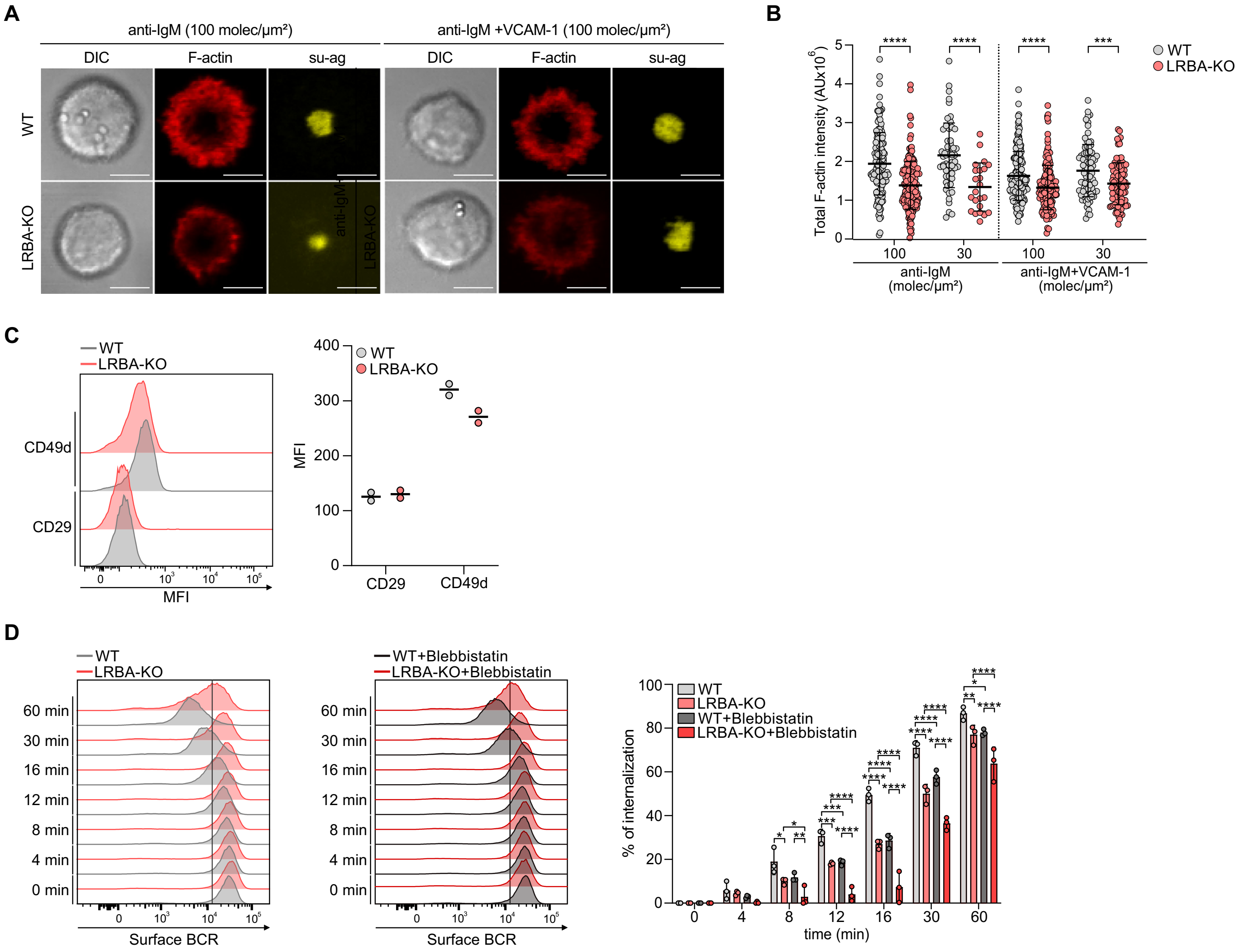

Figure EV5. LRBA-deficient B cells show reduced F-actin intensity during immune synapse formation.

(**A**, **B**) WT and LRBA-KO Ramos B cells were allowed to settle on planar artificial lipid bilayers coated with different densities (30, 100 molecules/µm²) of surrogate antigen (su-ag) anti-IgM or anti-IgM and VCAM-1 for 30 min, fixed and analyzed for total F-actin intensity using Phalloidin staining. (**A**) Representative differential interference contrast (DIC), F-actin and su-ag images of WT and LRBA-KO Ramos B cells with 100 molecules/µm² density. Scale bar: 5 µm. (**B**) Analysis of total F-actin intensity of WT (grey) and LRBA-KO (red) Ramos B cells. Each dot represents one cell (anti-IgM: WT-100: $n = 138$, KO-100: $n = 183$, WT-30: $n = 61$, KO-30: $n = 23$) (anti-IgM+VCAM-1: WT-100: $n = 185$, KO-100: $n = 175$, WT-30: $n = 84$, KO-30: $n = 96$) from $n = 3$ biological replicates, mean ± SD shown by the black line and error bars. (**C**) Expression of CD29 (Integrin β-1) and CD49d (Integrin α-4) (VLA-4) of WT (grey) and LRBA-KO (red) Ramos B cells. Each dot represents the mean of $n = 2$ technical replicates, while the mean of $n = 2$ biological replicates is shown by the black line. (**D**) WT (grey) and LRBA-KO (red) Ramos B cells were left untreated (light color) or treated with 50 µM Blebbistatin (dark color) and incubated with 10 µg/ml Biotin-coupled anti-IgM for 30 min at 4 °C. Cells were then incubated at 37 °C for the indicated time points and surface BCRs bound to anti-IgM were detected by Neutravidin-DyLight633 staining. Each dot represents one biological replicate, while bars represent mean ± SD of $n = 3$ biological replicates. AU Arbitrary Units. Statistical analysis was performed using Welch's *t* test (**B**) or two-way ANOVA with Tukey's multiple comparisons test (**D**), *$P < 0.05$ (**D**: WT vs KO-8 min: $P = 0.0103$, **D**: KO vs. KO Blebbistatin-8 min: $P = 0.0482$, **D**: WT vs. WT Blebbistatin-60 min: $P = 0.0122$), **$P < 0.01$ (**D**: WT Blebbistatin vs KO Blebbistatin-8 min: $P = 0.083$, **D**: WT vs. KO-60 min: $P = 0.0046$), ***$P < 0.001$ (**B**: $P = 0.0003$, **D**: WT vs. KO-12 min: $P = 0.0002$, **D**: WT vs. WT Blebbistatin-12 min: $P = 0.0004$), ****$P < 0.0001$, ns: not significant.
