# Supplementary material for: LRBA regulates actin cytoskeleton dynamics through NMIIA during B cell immune responses
Source: EMBO Rep. 2026 Jun 12;27(14):3982–4010. doi: 10.1038/s44319-026-00831-3 (PMC13400755; doi:10.1038/s44319-026-00831-3)
Supplement: Supplementary file 1 — Peer Review File [file 44319_2026_831_MOESM1_ESM.pdf]

EMBO reports

LRBA regulates actin cytoskeleton dynamics through NMIIA during B cell immune responses

Elena Sindram, Juan Eduardo Montero-Hernández, Quentin Frenger, Hannah van Dijk, Anna Lang, Vanessa Zeidler, Rebecca Marsiske, Manuel Rhiel, Kerstin Geiger, Tatjana Cornu, Emily Mace, Volker Spindler, Jörn Dengjel, Frederic Gros, Bodo Grimbacher, Fernando Sepulveda, Yolanda Carrasco, Virginia Andreani, and Laura Gámez-Díaz

Corresponding author(s): Elena Sindram (elena.sindram@uniklinik-freiburg.de) , Laura Gámez-Díaz (laura.gamez@uniklinik-freiburg.de), Virginia Andreani (virginia.andreani@uniklinik-freiburg.de)

Review Timeline:

Submission Date:	22nd Oct 25
Editorial Decision:	18th Nov 25
Revision Received:	15th Apr 26
Editorial Decision:	18th May 26
Revision Received:	26th May 26
Accepted:	30th May 26

Editor: Achim Breiling

Transaction Report:

(Note: With the exception of the correction of typographical or spelling errors that could be a source of ambiguity, letters and reports are not edited. Depending on transfer agreements, referee reports obtained elsewhere may or may not be included in this compilation. Referee reports are anonymous unless the Referee chooses to sign their reports.)

Dear Dr. Sindram,

Thank you for the submission of your manuscript to EMBO reports. I have now received reports from the three referees that were asked to evaluate your study, which can be found at the end of this email.

As you will see, the referees think that these findings are of interest. However, they have several comments, concerns, and suggestions, indicating that a major revision of the manuscript is necessary to allow publication of the study in EMBO reports. As the reports are below, and all the referee concerns need to be addressed, I will not detail them here.

Given the constructive referee comments, I would thus like to invite you to revise your manuscript with the understanding that the concerns of the referees must be addressed in the revised manuscript and/or in a detailed point-by-point response. Acceptance of your manuscript will depend on a positive outcome of a second round of review. It is EMBO reports policy to allow a single round of revision only and acceptance of the manuscript will therefore depend on the completeness of your responses included in the next, final version of the manuscript.

Revised manuscripts should be submitted within three months of a request for revision. Please contact me to discuss the revision (also by video chat) if you have questions or comments regarding the revision, or should you need additional time.

When submitting your revised manuscript, please also carefully review the instructions that follow below.

PLEASE NOTE THAT upon resubmission revised manuscripts are subjected to an initial quality control prior to exposition to re-review. Upon failure in the initial quality control, the manuscripts are sent back to the authors, which may lead to delays. Frequent reasons for such a failure are the lack of the data availability section (please see below) and the presence of statistics based on n=2 (the authors are then asked to present scatter plots or provide more data points).

When submitting your revised manuscript, we will require:

1) a .docx formatted version of the final manuscript text (including legends for main figures, EV figures and tables), but without the figures included. Figure legends should be compiled at the end of the manuscript text.

2) individual production quality figure files as .eps, .tif, .jpg (one file per figure), of main figures and EV figures. Please upload these as separate, individual files upon re-submission.

The Expanded View format, which will be displayed in the main HTML of the paper in a collapsible format, has replaced the Supplementary information. You can submit up to 6 images as Expanded View. Please follow the nomenclature Figure EV1, Figure EV2 etc. The figure legend for these should be included in the main manuscript document file in a section called Expanded View Figure Legends after the main Figure Legends section. Additional Supplementary material should be supplied as a single pdf file labeled Appendix. The Appendix should have page numbers and needs to include a table of content on the first page (with page numbers) and legends for all content. Please follow the nomenclature Appendix Figure Sx, Appendix Table Sx etc. throughout the text, and also label the figures and tables according to this nomenclature.

For more details, please refer to our guide to authors:
http://www.embopress.org/page/journal/14693178/authorguide#manuscriptpreparation

Please consult our guide for figure preparation:
http://wol-prod-cdn.literatumonline.com/pb-assets/embo-site/EMBOPress_Figure_Guidelines_061115-1561436025777.pdf

See also the guidelines for figure legend preparation:
https://www.embopress.org/page/journal/14693178/authorguide#figureformat

3) a .docx formatted letter INCLUDING the reviewers' reports and your detailed point-by-point responses to their comments. As part of the EMBO Press transparent editorial process, the point-by-point response is part of the Review Process File (RPF), which will be published alongside your paper.

4) a complete author checklist, which you can download from our author guidelines (https://www.embopress.org/page/journal/14693178/authorguide). Please insert page numbers in the checklist to indicate where the requested information can be found in the manuscript. The completed author checklist will also be part of the RPF.

Please also follow our guidelines for the use of living organisms, and the respective reporting guidelines:
http://www.embopress.org/page/journal/14693178/authorguide#livingorganisms

5) that primary datasets produced in this study (e.g. RNA-seq, ChIP-seq, structural and array data) are deposited in an

appropriate public database. If no primary datasets have been deposited, please also state this in a dedicated section (e.g. 'No primary datasets have been generated and deposited'), see below.

See also: http://embor.embopress.org/authorguide#datadeposition

Please remember to provide a reviewer password if the datasets are not yet public.

The accession numbers and database should be listed in a formal "Data Availability" section that follows the model below. This is now mandatory (like the COI statement). Please note that the Data Availability Section is restricted to new primary data that are part of this study. This section is mandatory. As indicated above, if no primary datasets have been deposited, please state this in this section

Data availability

The datasets produced in this study are available in the following databases:

- RNA-Seq data: Gene Expression Omnibus GSE46843 (https://www.ncbi.nlm.nih.gov/geo/query/acc.cgi?acc=GSE46843)
- [data type]: [name of the resource] [accession number/identifier/doi] ([URL or identifiers.org/DATABASE:ACCESSION])

*** Note - All links should resolve to a page where the data can be accessed. ***

Moreover, I have these editorial requests:

6) We now request the publication of original source data with the aim of making primary data more accessible and transparent to the reader. You will receive a separate email with instructions for providing source data with your revised manuscript, including information how to upload and organize the files.

7) Our journal encourages inclusion of *data citations in the reference list* to directly cite datasets that were re-used and obtained from public databases. Data citations in the article text are distinct from normal bibliographical citations and should directly link to the database records from which the data can be accessed. In the main text, data citations are formatted as follows: "Data ref: Smith et al, 2001" or "Data ref: NCBI Sequence Read Archive PRJNA342805, 2017". In the Reference list, data citations must be labeled with "[DATASET]". A data reference must provide the database name, accession number/identifiers and a resolvable link to the landing page from which the data can be accessed at the end of the reference. Further instructions are available at: http://www.embopress.org/page/journal/14693178/authorguide#referencesformat

8) Regarding data quantification and statistics, please make sure that the number "n" for how many independent experiments were performed, their nature (biological versus technical replicates), the bars and error bars (e.g. SEM, SD) and the test used to calculate p-values is indicated in the respective figure legends (also for EV and Appendix figures). Please also check that all the p-values are explained in the legend, and that these fit to those shown in the figure. Please provide statistical testing where applicable. Please avoid the phrase 'independent experiment' but clearly state if these were biological or technical replicates. Please also indicate (e.g. with n.s.) if testing was performed, but the differences are not significant. In case n=2, please show the data as separate datapoints without error bars and statistics. See also: http://www.embopress.org/page/journal/14693178/authorguide#statisticalanalysis

If n<5, please show single datapoints for diagrams.

9) Please add scale bars of similar style and thickness to all microscopic images, using clearly visible black or white bars (depending on the background). Please place these in the lower right corner of the images themselves. Please do not write on or near the bars in the image but define the size in the respective figure legend.

10) Please also note our reference format:
http://www.embopress.org/page/journal/14693178/authorguide#referencesformat

11) We updated our journal's competing interests policy in January 2022 and request authors to consider both actual and perceived competing interests. Please review the policy https://www.embopress.org/competing-interests and update your competing interests if necessary. Please name this section 'Disclosure and Competing Interests Statement' and put it after the Acknowledgements section.

12) We now use CRediT to specify the contributions of each author in the journal submission system. CRediT replaces the author contribution section. Please use the free text box to provide more detailed descriptions and do NOT provide your final manuscript text file with an author contributions section. See also our guide to authors:
https://www.embopress.org/page/journal/14693178/authorguide#authorshipguidelines

13) All Materials and Methods need to be described in the main text using our 'Structured Methods' format, which is required for

all research articles. According to this format, the Methods section should include a Reagents and Tools Table (listing key reagents, experimental models, software, and relevant equipment and including their sources and relevant identifiers), uploaded as separate file, and a Methods section in which we encourage the authors to describe their methods using a step-by-step protocol format with bullet points, to facilitate the adoption of the methodologies across labs. More information on how to adhere to this format as well as downloadable templates (.doc) for the Reagents and Tools Table can be found in our author guidelines (section 'Structured Methods'):

https://www.embopress.org/page/journal/14693178/authorguide#manuscriptpreparation

14) Please add up to 5 keywords to the manuscript and order the manuscript sections like this, using only these names:
Title page - Abstract - Keywords - Introduction - Results - Discussion - Methods - Data availability section - Acknowledgements (please include here all the funding information) - Disclosure and Competing Interests Statement - References - Figure legends - Expanded View Figure legends

15) Please make sure that all the funding information is also entered into the online submission system and that it is complete and similar to the one in the acknowledgement section of the manuscript text file.

We would also welcome the submission of cover suggestions, or motifs to be used by our Graphics Illustrator in designing a cover.

As part of the EMBO publication's Transparent Editorial Process, EMBO reports publishes online a Review Process File (RPF) to accompany accepted manuscripts. This File will be published in conjunction with your paper and will include the referee reports, your point-by-point response and all pertinent correspondence relating to the manuscript.

You are able to opt out of this by letting the editorial office know (emboreports@embo.org). If you do opt out, the Review Process File link will point to the following statement: "No Review Process File is available with this article, as the authors have chosen not to make the review process public in this case."

I look forward to seeing a revised form of your manuscript when it is ready.

Yours sincerely,

Achim Breiling
Senior Editor
EMBO Reports

Referee #1:

Sindram et al. describe the interaction of LRBA with 33 specific partners, particularly with MYH9, indicating a potential role in actin cytoskeleton remodeling.

The authors measured the motility of B cell lines derived from patients or RAMOS KO-LRBA lines. They observed an increased migration velocity that was corrected by incubating the cells with a MYH9 inhibitor (Blebbistatin). They also observed decreased nuclear deformation capacity in LRBA-deficient cells.
The authors then demonstrated a BCR signaling defect in LRBA-deficient lines. This defect is associated with decreased F-actin polymerization in response to BCR stimulation. They also observed a defect in the formation of a mature immunological synapse in LRBA KO B cells, which is associated with impaired BCR clustering and internalization.

This is an interesting study that highlights the interaction of LRBA with MYH9 and therefore the potential role of LRBA in actin cytoskeleton remodeling and BCR-dependent signaling. The authors propose that this signaling deficit could explain the humoral deficit observed in LRBA patient.

Comments:

-LRBA partners were identified from B-LCLs of controls and LRBA-deficient patients. Supplementary Table 1 lists the identified partners. Unless I've misread it, I don't see MYH9 in this list.
-The interaction with MYH9 was successfully validated in HEK293T cells by immunoprecipitation and in B-LCLs by proximity ligation assay (PLA). Unexpectedly, the other partners identified in LCLs do not appear to interact with LRBA in HEK293T cells. But what about the interaction in B-LCLs by PLA? Is it possible that the initially identified interactions require proteins that are not expressed in HEK293T cells?
- Through which respective domains of LRBA and MYH9 is the interaction likely to occur? Is there any structural data available?
- LRBA-deficient cells migrate more rapidly through microchannels that allow for spontaneous migration. The authors conclude

that this is likely a consequence of F-actin disorganization (between the front and rear of the cell). Given the system used, it is possible to visualize the distribution of actin within these microchannels. It would be interesting to show the images to support the authors' conclusions.
-One limitation of this work is that the authors use patient B-LCLs to study migration and then the Ramos LRBA KO model to study the BCR signaling. Why are both cell models not used throughout the entire study?
-If the MYH9-LRBA interaction is central to the BCR-dependent signaling defect, the signaling should be studied in a MYH9 deficient cell model, either B-LCLs from MYH9 deficient patients or Ramos KO-MYH9.
-Another limitation of the study is that MYH9 appears to be very poorly expressed at the transcriptomic level in the Ramos cell line. Did the authors verify protein expression in their cell line? If expression is low, is it possible that the observed signaling deficiency is related to an interaction of LRBA with another protein?
-MYH9 deficiency in humans is associated with macrothrombocytopenia and inclusion bodies in granulocytes (and also deafness). The granulocyte inclusions consist of cytoplasmic RNA-containing inclusions (Dohle bodies). Are these characteristics found in LRBA patients? It seems that thrombocytopenia is more likely of autoimmune origin ?
-Considering the central role that the authors attribute to the LRBA-MYH9 interaction, it would be really interesting to compare LRBA deficiency with MYH9 deficiency, whether in cell models or in patient cells.

Referee #2:

This study examined the impact of LRBA deficiency on human B-cell biology. The first part of the manuscript identified non-muscle myosin IIA (NMIIA) as an interacting partner of LRBA in B cells using coimmunoprecipitation and mass spectrometry, and verified this interaction using the proximity ligation. The rest of the manuscript characterized the phenotypes of LRBA deficiency using the human B cell line Ramos and primary human B cells, as well as primary B cell-derived lymphoblasts from LRBA-deficient patients. The phenotypes include reduced chemokine-induced migration, increased random migration, and reductions in cellular F-actin levels, immunological synapse formation, the microtubule organization center and lysosome polarization, and B-cell receptor (BCR) internalization. These data, together, suggest that LRBA regulates B cell functions through modulating the actin cytoskeleton. While the identification of NMIIA as an LRBA-interacting partner and the phenotypic data presented here are interesting, this manuscript fails to reveal the mechanisms by which LRBA regulates actin reorganization in response to B cell receptor (BCR) activation through its interaction with NMIIA. Furthermore, the authors over-interpreted or misinterpreted some of their data.

Specific comments:
1. This manuscript identified NMIIA as an interacting partner of LRBA. This part of the data is strong and convincing. However, in the rest of the manuscript, NMIIA was not examined. The spatial and functional relationships between LRBA and NMIIA during B cell migration, immunological synapse formation, and BCR activation and internalization were not examined, thereby missing a great opportunity to elucidate their mechanistic interactions. Instead, the authors conclude by correlating their data with previously published research data.

2. The roles of NMIIA and the actin cytoskeleton in cell migration have been well studied. If the authors can examine the spatial relationships of LRBA, NMIIA, and F-actin during B cell migration using immunofluorescence, the resulting data will provide clues as to whether LRBA regulates B cell migration through NMIIA or F-actin.

3. Fig. 2C is confusing. It is unclear what white dashes, their lengths, and their spacing represent.

4. Fig. 2F is unclear. If ~60% of LRBA-deficient B cells can pass the restricted area shown in the bar graph on the right, why does the diagram on the left show LRBA-deficient B cells stuck in the narrow area? Did LRBA-deficient cells move more slowly than WT cells or get stuck in the narrow area? Please add a time scale to the diagram.

5. Based solely on the reduced phalloidin staining levels in LRBA knockout cells, the authors concluded that LRBA-deficiency reduces actin polymerization. However, increased actin depolymerization can also reduce phalloidin staining. This possibility is not excluded in this manuscript.

6. When stimulating with soluble anti-IgM antibodies, surface BCRs cluster and move to one pole of a cell, driven by polarizing actin polymerization, in primary mouse B cells (Liu et al. J. Immunol. 2012). However, the authors observed only an increase in phalloidin staining, not actin polarization, in WT Ramos cells after stimulation. The authors did not examine surface BCR clustering in response to soluble anti-IgM stimulation either. Therefore, it is unclear whether B cells were sufficiently activated to reorganize the actin cytoskeleton. If Ramos cells do not reorganize the actin cytoskeleton due to their lymphoma nature, it should be discussed.

7. In Fig. 4A, the authors used microtubule organization center (pericentrin) and lysosome (LAMP1) staining to define immunological synapses. First, the authors did not clearly describe how they defined immunological synapses using these two stainings in the results and methods sections. Second, without BCR staining, immunological synapses cannot be defined, as microtubule organization and lysosomes are not components of immunological synapses, but surface BCRs are, even though

they polarize towards immunological synapses.

8. Fig. 4G showed the most convincing data in this manuscript to support that LRBA-deficiency affects actin reorganization towards BCR activation sites, as phalloidin staining under an anti-IgM-coated bead was missing in LRBA-deficient B cells. However, the authors ignored this evidence and only measured the mean fluorescence intensity of phalloidin. Instead of the mean phalloidin fluorescence intensity, the authors should quantify the phalloidin fluorescence intensity ratio under the bead versus the opposite site of the plasma membrane. Again, there was no BCR staining to show the SMAC formation.

9. For Fig. 5, please describe how immunological synapses and closed cSMACs were defined and counted. Phalloidin staining should also be included to support the conclusion. To claim actin dynamic changes in LRBA-deficient cells, multiple early time points should be included.

Referee #3:

This study presents a new explanatory framework for humoral immune deficiencies in LRBA deficiency, by identifying NMIIA as a new interaction partner of LRBA in B cells and showing a role of LRBA in regulating actin cytoskeleton dynamics during B cell activation. This is conceptually appealing because although LRBA deficiency is classically defined as a T-cell/Treg/CTLA-4 trafficking disorder, B cells are also intrinsically affected, and several of their defects map onto processes that normally depend on non-muscle myosin II (NMII), particularly NMIIA.

The study relies somewhat heavily on EBV-transformed LCL cell lines. Although these retain B-cell identity, EBV transformation profoundly remodels the cytoskeletal and trafficking programs that underlie non-muscle myosin II (NMIIA)-dependent antigen extraction. This is largely mitigated by central experiments in Ramos cells, and a few in primary B cells.

It is somewhat hard to fully disentangle primary and secondary effects, i.e., if LRBA deficiency impairs BCR signalling, that could explain some of the defects in SMAC formation and antigen internalization, and if LRBA impairs SMAC formation, this could explain some more of the downstream defects.

However, overall, the study is well presented and the approach and methodology appear sound. The manuscript is well-written, and the figures are for the most part very clear.

Results section:

It is curious that the SILAC experiment identifies as significant interactors ($p<0.05$) filamin A (FLNA), intersectin-2 (ITSN2), tropomyosin 3 (TPM3) and vimentin (VIM), whereas myosin heavy chain 9 (MYH9) and myosin 18A (MYO18A) although strongly enriched (log2 fold change > 0.82) do not achieve significance ($p<0.13$). Can the authors comment on this?

Their data indicate that LRBA interacts with NMIIA in both healthy donor LCL cells and transfected HEK293T cells, suggesting a role for LRBA in actin cytoskeleton regulation. However, could this not also be shown in primary healthy B cells, potentially after LPS activation?

The CXCL12-driven chemotactic response would have similarly been interesting to see in primary naïve B cells either LRBA-sufficient or deficient upon LPS stimulation.

"Interestingly, evaluation of B cell migration in microchannels with 3 µm constrictions, mimicking an in vivo situation, showed that loss of LRBA progressively reduced the cell passage through micrometric spaces, which is likely attributed to a poor capacity to deform the nucleus (Figure 2F)." The authors should provide a reference, and specify what 'in vivo situation' this is suggested to reflect, e.g., extravasation.

"Of note, surface and intracellular IgM expression was similar between WT and LRBA-KO cells (Supplementary Figure 2E), demonstrating that reduced BCR response is not due to abnormal IgM expression. Taken together, our data suggest that LRBA is important for regulating F-actin polymerization after BCR stimulation..." Although overall expression does not differ, it is very possible that IgM localization is dysregulated before BCR activation, since the actin and tetraspanin networks have been shown to organize receptor nanoclusters (Mattila et al., Immunity 2013). Did the authors investigate this possibility?

"we tested the formation of the mature IS in WT and LRBA-KO Ramos B cells stimulated on anti-IgM-coated slides mimicking immobilized antigen presentation..." Because of the lack of lateral mobility of antigen on antigen-coated slides, it is a poor model for IS formation, which relies on lateral mobility. It would have been more proper to employ a planar lipid bilayer system. Can the authors comment on this in relation to their observed IS formation? (I see they used PLB later).

VLA-4 (very late antigen-4, integrin α4β1) undergoes classic inside-out signaling. Can the authors comment on their observation of intact VLA-4 mediated adhesion and contact area by LRBA-KO Ramos cells, given that LRBA interferes with BCR-mediated

signals required for inside-out signaling?

Discussion section:

"Previous reports on reduced migration of LRBA-deficient LCL cells towards Sphingosine-1-phosphate (S1P) with normal S1P internalization may also be caused by the observed actin cytoskeleton abnormalities during migration instead of S1P receptor downstream signalling (Sic et al., 2014)." Can the authors qualify this comment in the light of their finding that amoeboid-type cell motility is actually increased. Were the conditions of the here reinterpreted experiments by Sic et al. such that one would expect a non-amoeboid mode of migration?

"LRBA-deficient patients show normal generation of germinal centers", does this not go against the authors own assertion that LRBA is important for BCR signaling, SMAC formation and antigen presentation, mechanisms that are all required for the primary foci that precede GC formation?

"Stimulation through the BCR and CD40, in combination with IL-4, triggers the activation of activation-induced cytidine deaminase (AID) necessary for the initiation of class-switch recombination (Stavnezer et al., 2008)." What is meant by 'activation of AID'? BCR signals combined with CD40L and IL-4 cause increased expression of AID.

Tables and Figures:

Table 1: There are many histones and nuclear proteins identified in the SILAC MS analysis. Could the authors comment on the cellular localization/distribution of LRBA?

Figure 1: The blue-line-identified cut-off is quite non-standard. Can the authors comment on this?

The authors reference a study showing interaction of LRBA with AID by co-IP, an interaction not identified here. This is likely because the LCL subjected to this analysis do not express AID. The authors should discuss the limitations of the applied methodology in terms of what is expressed in their LCL at 'resting' conditions, compared to potential important interactors in e.g., activated or GC B cells.

Minor points:

177-181: It should be made clear in the text what cell type(s) are referred to here, i.e., LCL and/or Ramos.

194: "after IgM stimulation", should be: 'after IgM crosslinking', or 'after anti-IgM stimulation'.

301-302: "Similarly, loss of LRBA in B cells exhibited distinct migration behaviours depending on the analysed environment." Should be rephrased, e.g., 'Similarly, upon loss of LRBA, B cells exhibited distinct migration behaviours depending on the analysed environment.'

In Figure 4B, E, H, please place the mean/median bars + errors on top of the individual data points. As it is now, it is not possible to see.

This is actually the case for several of the subsequent figures as well.

Point by point reply

Referee-#1:
Sindram et al. describe the interaction of LRBA with 33 specific partners, particularly with MYH9, indicating a potential role in actin cytoskeleton remodeling.

The authors measured the motility of B cell lines derived from patients or RAMOS KO-LRBA lines. They observed an increased migration velocity that was corrected by incubating the cells with a MYH9 inhibitor (Blebbistatin). They also observed decreased nuclear deformation capacity in LRBA-deficient cells.
The authors then demonstrated a BCR signaling defect in LRBA-deficient lines. This defect is associated with decreased F-actin polymerization in response to BCR stimulation. They also observed a defect in the formation of a mature immunological synapse in LRBA KO B cells, which is associated with impaired BCR clustering and internalization.

This is an interesting study that highlights the interaction of LRBA with MYH9 and therefore the potential role of LRBA in actin cytoskeleton remodeling and BCR-dependent signaling. The authors propose that this signaling deficit could explain the humoral deficit observed in LRBA patient.

Comments:
1.1 LRBA partners were identified from B-LCLs of controls and LRBA-deficient patients. Supplementary Table 1 lists the identified partners. Unless I've misread it, I don't see MYH9 in this list.
We thank the reviewer for pointing out the absence of MYH9 from Table 1. This omission was because Table 1 only included proteins that reached statistical significance at $p < 0.05$. MYH9 had a p-value of 0.12846 with a log2 fold change of 0.83. In this revised manuscript, we have updated Table 1 to include proteins that also reached the secondary threshold of $p < 0.13$ **(page 62-64).** These include MYH9 and 21 other proteins. Of note, among these proteins, only MYH9 and MYO18A were selected for further analysis, as they were also enriched in the mass spectrometry dataset and are functionally associated with cytoskeleton organization in B cells (Seeley-Fallen et al., 2022, PMID: 35493485; Hoogeboom et al., 2018, PMID: 29791846; Matsui et al., 2012, PMID: 21751808; Cheung et al., 2022, PMID: 34001658).

1.2 The interaction with MYH9 was successfully validated in HEK293T cells by immunoprecipitation and in B-LCLs by proximity ligation assay (PLA). Unexpectedly, the other partners identified in LCLs do not appear to interact with LRBA in HEK293T cells. But what about the interaction in B-LCLs by PLA? Is it possible that the initially identified interactions require proteins that are not expressed in HEK293T cells?
We thank the reviewer for raising this point. We focused our validation analyses on candidates that showed evidence of interaction in the overexpression-based co-IP system. As the other candidates did not demonstrate interaction with LRBA in HEK293T cells, we did not pursue their validation by PLA in LCL cells. Our rationale was to first establish robust biochemical evidence of interaction under controlled conditions before proceeding to endogenous validation.

Importantly, we agree with the reviewer that cell-type-specific factors such as other proteins not expressed in HEK293T cells may influence these interactions. While overexpression-based approaches are widely used to assess direct protein–protein interactions sensitively and independently of cell type

or endogenous factors, they may fail to capture interactions that depend on additional cofactors. Therefore, our approach cannot exclude the possibility that LRBA requires additional endogenous proteins, which exist in B cells but not in HEK293T cells, to enable the interactions of LRBA with the identified potential candidates.

Consistent with this, we validated the LRBA-MYH9 interaction both in the overexpression system (co-IP) and at endogenous levels by PLA in LCL cells. In the revised version, we have also validated the LRBA-MYH9 interaction in isolated primary B cells from a healthy donor stimulated with CD40L and IL-21 (**Reviewer Figure R1, Expanded View Figure 1G**).

Finally, we note that validation rates of potential interactors originally identified by proteomics are inherently low (Nesvizhskii et al., 2007, PMID: 17901868; Nesvizhskii, 2012, PMID: 22611043), and such datasets should be considered interpreted as sources of candidate interactions rather than definitive binding partners.

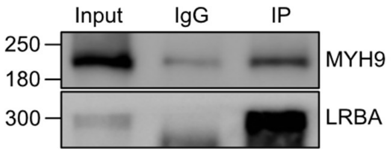

Reviewer Figure R1 and Expanded View Figure 1G. *HD primary B cells were stimulated with 250 ng/ml CD40L and 10 µg/ml IL-21 for 24 h. Immunoprecipitation was performed using anti-LRBA and immunoblotted for MYH9. n=1 biological experiment.*

1.3 Through which respective domains of LRBA and MYH9 is the interaction likely to occur? Is there any structural data available?

We thank the reviewer for this comment. As there is no complete structural data available on LRBA and MYH9 for us to run *in silico* predictions, we addressed this question in our revised manuscript by co-expressing full-length MYH9 (tagged to mCherry) alongside seven different Flag-tagged LRBA fragments (F1 to F7), each containing a different LRBA protein domain. Following co-IP experiments, we determined that MYH9 interacts with fragment 5, which encodes the domain of unknown function (DUF) 1088.

DUF domains are generally conserved domains known to mediate protein-protein interactions (Bateman et al., 2010, PMID: 20944204). Only LRBA and Neurobeachin (NBEA) harbour a DUF1088 domain within the BEACH protein family (Cullinane et al., 2014, PMID: 23521701). Interestingly, NBEA localizes in the nucleus of mouse neuronal cells via a conserved penta-arginine nuclear localization sequence (NLS) (Tuand et al., 2016, PMID: 26999814). Although LRBA has been reported to be predominantly localized in the cytoplasm, Reiser et al. found LRBA in the nucleus of mouse bone marrow cells (Reiser et al., 2014). It will be of interest to determine if this nuclear location is via its DUF1088 domain. Notably, DUF domains have been directly linked to cytoskeletal regulation. In The DUF domain of PDZ And LIM Domain 5 (PDLIM5), the DUF domain mediates cytoskeletal organization and dendritic morphology (Srivastava et al., 2024, PMID: 39125895), while in the DUF domain of Microtubule-associated serine/threonine-protein kinase 3 (MAST3) it supports protein–protein interactions in cytoskeletal signaling (Lubaba et al, 2025, PMID: 41203242). Our identification of DUF1088 as the interaction site between LRBA and MYH9 further implicates the DUF domain in cytoskeletal functions.

These results are shown below in **Reviewer Figure R2** and are now included in the manuscript as **Figure 1, F and G** and in the **Result section (page 6, line 151-155)** and **Discussion (page 13, line 323-330)** in the revised manuscript.

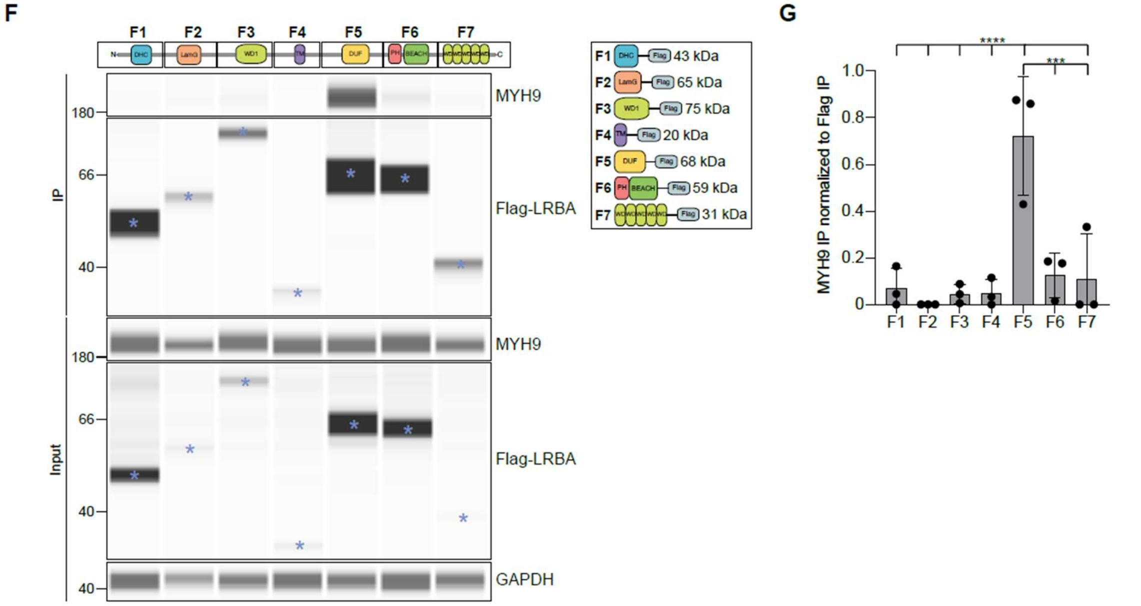

Reviewer Figure R2 and Figure 1, F and G. (F) *WT HEK293T cells were co-transfected with a plasmid encoding full length, mCherry-tagged MYH9 and Flag-tagged LRBA protein domains (fragments 1 to 7). Flag-LRBA was immunoprecipitated with anti-Flag, and western blots were probed with anti-MYH9. Schematic representation of plasmids containing the different LRBA protein domains is shown on the top and right. Asterisks in the western blots indicate the LRBA protein domains.* ***(G)*** *Ratio of MYH9/LRBA fragment detected by co-IP. Each dot represents one biological replicate, while bars represent the mean ± SD of n=3 biological replicates. Statistical analysis was performed using Two-Way ANOVA with Tukey's multiple comparison test, ***p<0.001 (F5 vs. F6: p=0.0002, F5 vs. F7: p=0.0001), ****p<0.0001.*

1.4 LRBA-deficient cells migrate more rapidly through microchannels that allow for spontaneous migration. The authors conclude that this is likely a consequence of F-actin disorganization (between the front and rear of the cell). Given the system used, it is possible to visualize the distribution of actin within these microchannels. It would be interesting to show the images to support the authors' conclusions.

We thank the reviewer for this suggestion. We have therefore analyzed the distribution of F-actin in LCL cells in microchannels. For this, LCL cells were let to migrate in microchannels, fixed and stained for F-actin using Phalloidin. Our results showed that HD LCL cells uniformly distributed F-actin between the front and the rear. In contrast, LRBA-deficient cells showed an accumulation of F-actin at the cell front. This altered distribution is consistent with our conclusion that LRBA deficiency leads to disorganized front-rear cytoskeletal polarity during confined migration.

We also stained for phosphorylated myosin light chain (pMLC) to indicate actomyosin contractility (Pecci et al., 2018, PMID: 29679756). In HD LCL cells, pMLC is enriched at the rear of the cell, which is consistent with rear-driven actomyosin contractility facilitating tail retraction and directional

migration. In contrast, LCL cells from the LRBA-deficient patient exhibited a uniform distribution of pMLC between the front and rear of the cell, indicating a loss of spatially restricted contractibility. Together, these results highlight the critical role of LRBA in maintaining B cell polarity during amoeboid migration.

These results are shown below in **Reviewer Figure R3** and are now included in the manuscript as **Figure 2, D-E** and in the **Result section (page 7 and 8, line 182-187)** and **Discussion (page 13, line 338-339)** in the revised manuscript.

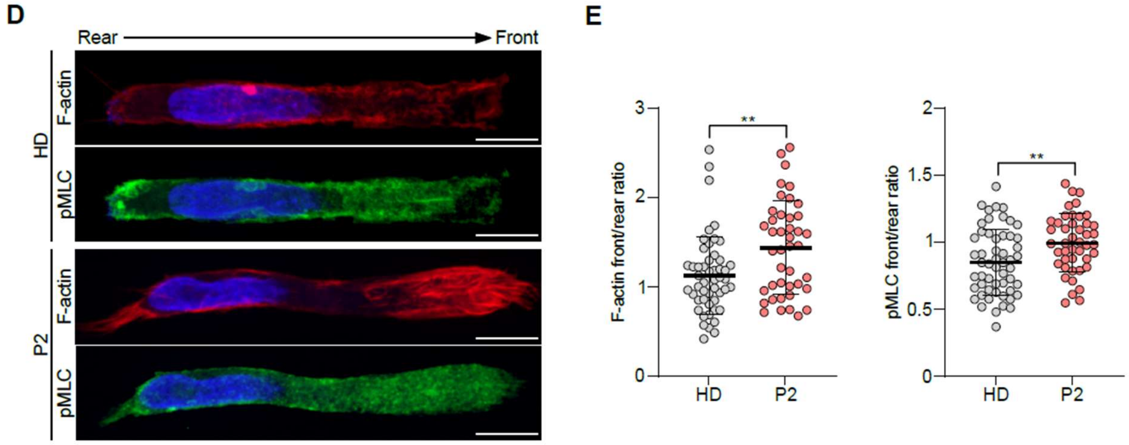

Reviewer Figure R3 and Figure 2, D and E. (D) *Representative confocal microscopy images of HD and LRBA-P2 LCL cells which were let to migrate in microchannels, fixed and stained for F-actin using Phalloidin (red), pMLC (green) and Hoechst (blue). Scale bar: 10 µm.* ***(E)*** *Analysis of F-actin (left) and pMLC (right) distribution between the front and the rear in HD (grey) and LRBA-P2 (red) LCL cells. Each dot represents one cell (F-actin: HD: n=51, P2: n=42; pMLC: HD: n=54, P2: n=44) from n=3 biological replicates, mean ± SD shown by the black line and error bars. Statistical analysis was performed using Welch's t-test, **p<0.01 (E-F-actin: p=0.0034, E-pMLC: p=0.0028, H: p=0.0072).*

1.5 One limitation of this work is that the authors use patient B-LCLs to study migration and then the Ramos LRBA KO model to study the BCR signaling. Why are both cell models not used throughout the entire study?

We thank the reviewer for raising this question. Although we aimed to use both cell models throughout the study to ensure consistency, technical limitations required the use of distinct systems for specific assays. For example, Ramos B cells were not suitable for migration experiments in microchannels because they exhibited minimal spontaneous motility and could not be reliably analyzed in microchannel assays. In contrast, LCL cells showed robust migration under confined conditions and are therefore an established model for these experiments (Gajardo et al., 2023, PMID: 37390900).

Conversely, patient-derived LCL cells were not suitable for BCR signaling analysis, because B-cell transformation using Epstein-Barr Virus (EBV) induces substantial transcriptional and phenotypic alterations, including changes in critical B cell markers such as CD19 and CD21, limiting their physiological relevance for signalling studies (MacKinnon et al., 2024, PMID: 38910966; SoRelle et al., 2021, PMID: 33501914). In addition, LCL cells lack surface IgM expression, precluding analysis of naïve BCR signaling responses, which depends on IgM engagement. Ramos B cells, which retain high surface IgM expression, are well-established model for BCR signalling, immune synapse formation, and

endocytosis, and were therefore used for these assays (Deretic et al., 2021, PMID: 34336818; He et al., 2018, PMID: 29669863; Kim et al., 2004, PMID: 15509800; Li et al., 2024, PMID: 38353705; Roper et al., 2019, PMID: 31815664; Schwarz et al., 2021, PMID: 34526379). The results on IgM expression are shown below for the reviewer only in **Reviewer Figure R4**.

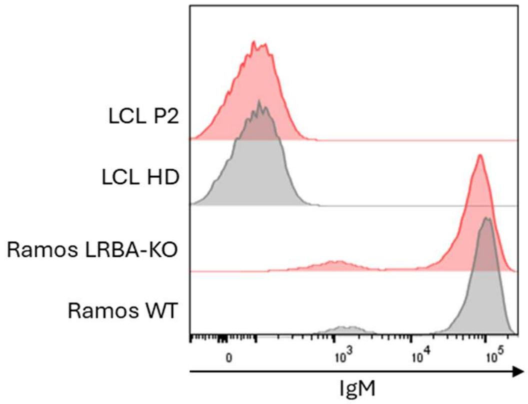

Reviewer Figure R4. *Flow cytometry analysis of surface expression of IgM in LCL cells from HD (grey) and LRBA-P2 (red) (top), and WT (grey) and LRBA-KO (red) Ramos B cells (bottom).*

1.6 If the MYH9-LRBA interaction is central to the BCR-dependent signaling defect, the signaling should be studied in a MYH9 deficient cell model, either B-LCLs from MYH9 deficient patients or Ramos KO-MYH9.

We thank the reviewer for this valuable suggestion and agree that studying BCR signaling in MYH9-deficient B cells would be highly informative. We therefore attempted to generate MYH9-deficient Ramos B cells using two different CRISPR-Cas9 approaches targeting exon 2. Despite efficient protocols of these two systems routinely used in our laboratory, we were unable to obtain MYH9 knock-out (KO) clones. The first method involved electroporating Ramos cells with a pMAX-CRISPR-Cas9-2A-GFP plasmid (14-55% success rate, Sindram et al., 2023, PMID: 37382373) and in the second, we used the Alt-R CRISPR-Cas9 system (4-14% success rate, Sindram et al., 2025, PMID: 40550954). Following single-cell sorting, only a small number of clones survived but all retained MYH9 expression.

Similarly, shRNA-mediated knock-down (KD) reduced MYH9 expression in Jurkat T and HEK293T but not in Ramos B cells. This data is presented for the reviewer in **Reviewer Figure 5.**

This suggests that MYH9 loss is poorly tolerated in Ramos B cells or any initial KD clones are likely overgrown by those cells retaining normal MYH9 expression. This is consistent with prior evidence that complete MYH9 deficiency is incompatible with viability, as homozygous loss is embryonically lethal in mice and has not been reported in humans (Conti et al., 2004, PMID: 15292239).

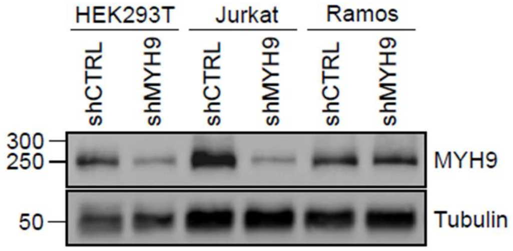

Reviewer Figure R5. *Western Blot analysis of MYH9 expression in HEK293T, Jurkat T and Ramos B cells transduced with either CTRL or MYH9 shRNA. Tubulin was used as a loading control.*

In the absence of a MYH9-deficient model we used Blebbistatin, a well-established non-muscle myosin II inhibitor (Straight et al., 2003), to assess the contribution of NMII activity to BCR signaling, migration, and immune synapse (IS) formation and BCR internalization. Our data shows that Blebbistatin treatment phenocopied LRBA deficiency in transwell migration **(Reviewer Figure R8),** IS formation **(Expanded View Figure 4A-C)** and BCR internalization **(Reviewer Figure R9)**, all processes that depend on NMIIA-driven actomyosin contractility (Seeley-Fallen et al., 2022, PMID: 35493485; Hoogeboom et al., 2018, PMID: 29791846). On the contrary, Blebbistatin did not affect BCR-induced phosphorylation of Syk (pSyk) or PLCy2 (pPLCy2) in either WT or LRBA-KO cells **(Reviewer Figure R6)**. These results indicate that the BCR signaling defect in LRBA-deficient cells is unlikely to result directly from impaired NMII contractility. Instead, they support a model in which LRBA regulates early BCR signaling through broader effects on actin dynamics, consistent with the defective Arp2/3-dependent F-actin polymerization observed in our study.

Although NMII is recruited to the BCR contact zone during the early response to antigen engagement, its principal function is in the contractile remodelling that promotes BCR clustering and signal attenuation, rather than in the initiation of downstream signalling itself (Seeley-Fallen et al., 2022, PMID: 35493485; Bhanja et al., 2022, PMID: 34124846). Blebbistatin selectively inhibits NMII ATPase activity and contractility (Straight et al., 2003, PMID: 12637748; Kovács et al., 2004, PMID: 15205456), and it has been shown that the initial Arp2/3-driven F-actin polymerization burst that drives early BCR signalling is largely independent of NMII contractility (Bolger-Munro et al., 2019, PMID: 31157616; Li et al., 2019, PMID: 30687315, Seeley-Fallen et al., 2022, PMID: 35493485). Thus, the Blebbistatin experiment therefore indicates that the BCR signalling defect in LRBA-KO cells is not a direct consequence of impaired NMII contractility, but rather reflects a broader dysregulation of Arp2/3-dependent actin remodelling during BCR engagement, as supported by the impaired early F-actin polymerization demonstrated in Figure 3A-B.

These results are shown below in **Reviewer Figure R6** and are now included in the manuscript as **Expanded View Figure 3, A and B**, and in the **Result section (page 9, line 213-216)** and the **Discussion (page 15 and 16, line 391-397)** in the revised manuscript. Of note, we used Western Blot for pSyk analysis in our revision experiments due to the low overall MFI in our flow cytometry analysis.

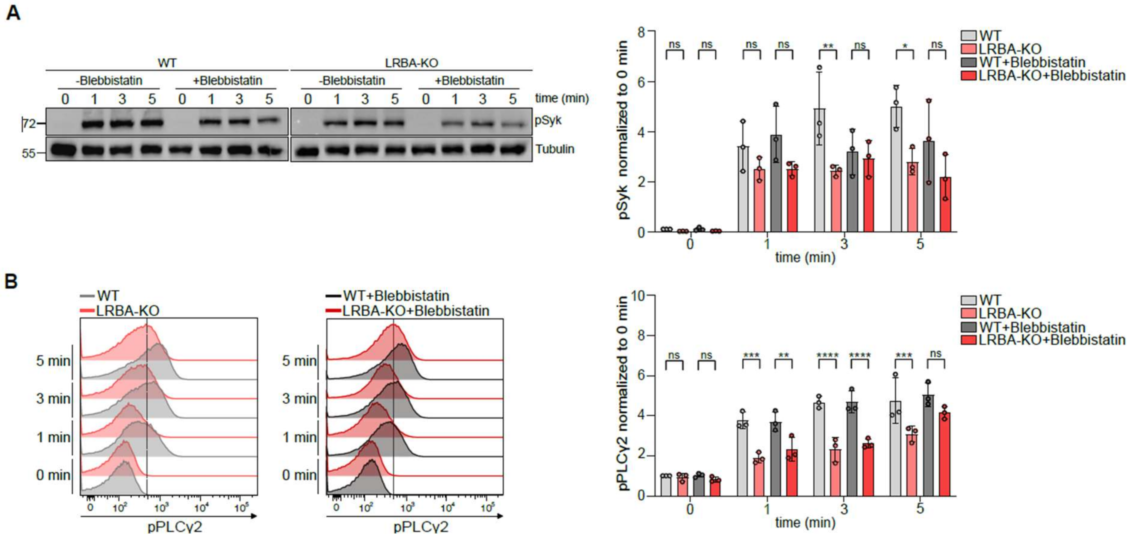

Reviewer Figure R6 and Expanded View Figure 3, A and B. (A-B) *WT (grey) and LRBA-KO (red) Ramos B cells were left untreated (light color) or treated with 50 µM Blebbistatin (dark color) for 30 min and stimulated with 5 µg/ml anti-IgM for the indicated time points.* ***(A)*** *Phosphorylation of Syk (pSyk) analyzed by western blot. Left: Representative immunoblot analyses of pSyk expression of one experiment. Tubulin was used as loading control. Right: Densitometry analyses of pSyk immunoblots normalized first to Tubulin and to then pSyk WT 0 min.* ***(B)*** *Phosphorylation of PLCy2 (pPLCy2) analyzed by flow cytometry. Left: Representative histograms of pPLCy2 MFI of one experiment. Right: Analysis of MFI normalized to pPLCy2 WT 0 min. Each dot represents one biological replicate, while bars represent mean ± SD of n=3 biological replicates. Statistical analysis of all experiments was performed using Two-way ANOVA with Tukey's multiple comparisons test, *p<0.05 (A: p=0.011), **p<0.01 (A: p=0.0038, B: p=0.0057), ***p<0.001 (B-1min: p=0.0002, B-5min: p=0.0007), ****p<0.0001. All other comparisons are ns: not significant.*

1.7 Another limitation of the study is that MYH9 appears to be very poorly expressed at the transcriptomic level in the Ramos cell line. Did the authors verify protein expression in their cell line? If expression is low, is it possible that the observed signaling deficiency is related to an interaction of LRBA with another protein?

We appreciate the reviewer's concern. While we did not analyze MYH9 expression in Ramos B cells at transcriptomic level, we directly assessed MYH9 protein expression by Western blot and found robust MYH9 protein expression in both Ramos B cells and LCL cells **(Reviewer Figure R7)**, confirming that MYH9 is abundantly expressed at protein level in our experimental model. These results corroborate the data provided by the Human Protein Atlas at the transcriptomic level, which indicates that MYH9 is highly expressed in the RNA of Ramos B cells (https://www.proteinatlas.org/ENSG00000100345-MYH9/cell+line).

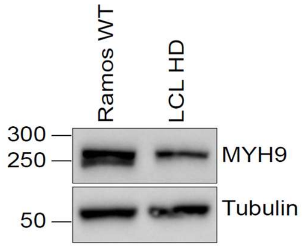

***Reviewer Figure R7**. Western Blot analysis of MYH9 protein expression in WT Ramos B cells and LCL cells from a healthy donor (HD). Tubulin was used as a loading control.*

1.8 MYH9 deficiency in humans is associated with macrothrombocytopenia and inclusion bodies in granulocytes (and also deafness). The granulocyte inclusions consist of cytoplasmic RNA-containing inclusions (Dohle bodies). Are these characteristics found in LRBA patients? It seems that thrombocytopenia is more likely of autoimmune origin?

We thank the reviewer for this observation. LRBA-deficient patients do not exhibit the typical features of MYH9-related disease (MYH9-RD), such as macrothrombocytopenia with giant platelets and Döhle-like inclusion bodies in granulocytes, or associated syndromic features, including hearing loss and nephropathy (Kelly et al., 2000, PMID: 10973260; Kunishima et al., 2001, PMID: 11159552; Seri et al., 2000, PMID: 10973259; Shen et al., 2024, PMID: 39309229).

As suggested by the reviewer, thrombocytopenia in LRBA deficiency is generally considered to be autoimmune origin (immune thrombocytopenia, ITP). This is consistent with the well-established role of LRBA in immune regulation: its deficiency leads to regulatory T cell dysfunction, loss of immune tolerance, and autoantibody production including those against platelets and other self-antigens (Alkhairy et al., 2015, PMID: 26707784; Bal et al., 2017, PMID: 28956255; Gámez-Díaz et al., 2016, PMID: 26768763; Habibi et al., 2019, PMID: 30995531; Jamee et al., 2021, PMID: 33788257).

The distinct clinical manifestations of MYH-9 RD and LRBA deficiency likely reflect their differential expression and functional roles. MYH9 is highly expressed in granulocytes and platelets, where it directly contributes to cytoskeletal organization (Human Protein Atlas: https://www.proteinatlas.org/ENSG00000100345-MYH9; Pecci et al., 2005, PMID: 16162639), whereas LRBA is predominantly expressed in activated lymphocytes and is critical for immune homeostasis (Human Protein Atlas: https://www.proteinatlas.org/ENSG00000198589-LRBA; Gámez-Díaz et al., 2018, PMID: 29740429). Accordingly, LRBA deficiency results in immune dysregulation and defective humoral immunity, features not reported in MYH9-RD.

1.9 Considering the central role that the authors attribute to the LRBA-MYH9 interaction, it would be really interesting to compare LRBA deficiency with MYH9 deficiency, whether in cell models or in patient cells.

We thank the reviewer for this suggestion and agree that a direct comparison between LRBA and MYH9 deficiency would be highly informative. However, as mentioned above in 1.6, our attempts to generate MYH9 KO or KD Ramos B cells were unsuccessful. In addition, we do not have access to patient samples with MYH9-related disorders.

To address this limitation, we used Blebbistatin as a functional surrogate for MYH9 loss. Using this approach, we evaluated BCR signaling, BCR internalization, cell migration and immune synapse formation. As described in 1.6, Blebbistatin treatment did not alter BCR-induced phosphorylation of

Syk or PLCγ2 in WT cells, nor did it further reduce signaling in LRBA-KO cells **(Reviewer Figure R6)**. In contrast, Blebbistatin treatment of HD LCL cells significantly reduced transwell migration **(Reviewer Figure R8)** and immune synapse formation **(Expanded View Figure 4 A-C)** and BCR internalization **(Reviewer Figure R9)** in WT Ramos B cells, closely phenocopying untreated LRBA-KO cells. Together, these data demonstrate that NMIIA contractility is required for transwell migration, immune synapse formation and BCR internalization but it is dispensable for early BCR signaling. Thus, while LRBA interacts with MYH9, its role in BCR signaling likely involves additional mechanisms beyond NMII-dependent contractility.

The results of transwell migration after Blebbistatin treatment are shown below in **Reviewer Figure R8** and are now included in the manuscript as **Expanded View Figure 2D** and in the **Result section (page 7, line 170-172)** and the **Discussion (page 14, line 347-349)** in the revised manuscript. The results of BCR internalization after Blebbistatin treatment are shown below in **Reviewer Figure R9** and are now included in the manuscript as **Expanded View Figure 5D** and in the **Result section (page 12, line 308-309)** and the **Discussion (page 15, line 388-391)** in the revised manuscript.

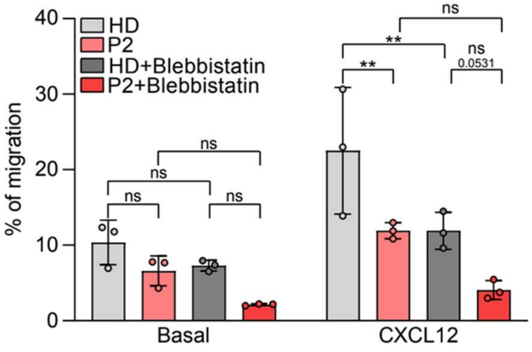

Reviewer Figure R8 and Expanded View Figure 2D. *Percentage of chemokine-directed cell migration was evaluated in HD (grey) and LRBA-P2 (red) LCL cells. Cells were left untreated (light colors) or treated with 10 µM Blebbistatin (dark colors) and migrated through a 5 µm membrane towards either RPMI only (Basal) or 100 ng/ml CXCL12 for 6 hours. Each dot represents one biological replicate, while bars represent mean ± SD of n=3 biological replicates. Statistical analysis was performed using Two-Way ANOVA with Tukey's multiple comparison test, **p<0.01 (WT vs. KO: p=0.0074, WT vs. WT-Blebbistatin: p=0.0074), ns: not significant.*

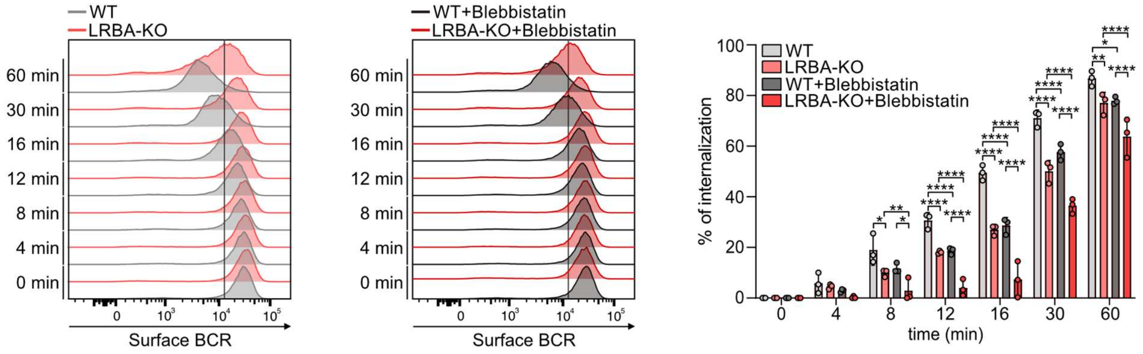

Reviewer Figure R9 and Expanded View Figure 5D. *WT (grey) and LRBA-KO (red) Ramos B cells were left untreated (light color) or treated with 50 µM Blebbistatin (dark color) and incubated with 10 µg/ml Biotin-coupled anti-IgM for 30 min at 4°C. Cells were then incubated at 37°C for the indicated time points and surface BCRs bound to anti-IgM were detected by Neutravidin-DyLight633 staining. Each dot represents one biological replicate, while bars represent mean ± SD of n=3 biological replicates. Statistical analysis was performed using Welch's t-test or using Two-way ANOVA with Tukey's multiple comparisons test, *p<0.05 (WT vs KO-8min: p=0.0103, KO vs. KO Blebbistatin-8min: p=0.0482, WT vs. WT Blebbistatin-60min: p=0.0122), **p<0.01 (WT Blebbistatin vs KO Blebbistatin-8min: p=0.083, WT vs. KO-60min: p= 0.0046), ***p<0.001 (WT vs. KO-12min: p=0.0002, WT vs. WT Blebbistatin: p=0.0004), ****p<0.0001. All other comparisons are ns: not significant.*

Referee-#2:

This study examined the impact of LRBA deficiency on human B-cell biology. The first part of the manuscript identified non-muscle myosin IIA (NMIIA) as an interacting partner of LRBA in B cells using coimmunoprecipitation and mass spectrometry, and verified this interaction using the proximity ligation. The rest of the manuscript characterized the phenotypes of LRBA deficiency using the human B cell line Ramos and primary human B cells, as well as primary B cell-derived lymphoblasts from LRBA-deficient patients. The phenotypes include reduced chemokine-induced migration, increased random migration, and reductions in cellular F-actin levels, immunological synapse formation, the microtubule organization center and lysosome polarization, and B-cell receptor (BCR) internalization. These data, together, suggest that LRBA regulates B cell functions through modulating the actin cytoskeleton. While the identification of NMIIA as an LRBA-interacting partner and the phenotypic data presented here are interesting, this manuscript fails to reveal the mechanisms by which LRBA regulates actin reorganization in response to B cell receptor (BCR) activation through its interaction with NMIIA. Furthermore, the authors over-interpreted or misinterpreted some of their data.

Specific comments:

2.1. This manuscript identified NMIIA as an interacting partner of LRBA. This part of the data is strong and convincing. However, in the rest of the manuscript, NMIIA was not examined. The spatial and functional relationships between LRBA and NMIIA during B cell migration, immunological synapse formation, and BCR activation and internalization were not examined, thereby missing a great opportunity to elucidate their mechanistic interactions. Instead, the authors conclude by correlating their data with previously published research data.

We thank the reviewer for this comment and agree that examining the spatial and functional relationship between LRBA and NMIIA would strengthen the mechanistic understanding of their interaction. Therefore, during the revision, we addressed this by investigating i) whether LRBA regulates NMIIA activation; and ii) whether LRBA and NMIIA co-localize spatially during cell migration and immune synapse formation.

To assess NMIIA activation, we measured phosphorylation of the myosin light chain (pMLC) in WT and LRBA-KO Ramos B cells following stimulation with soluble anti-IgM at time points corresponding to early signaling, immune synapse (IS) formation, BCR polarization and BCR internalization (0 min, 1 min, 3 min, 5 min, 15 min and 30 min). Our results show that LRBA-KO cells exhibited significantly reduced basal pMLC levels compared to WT cells, indicating impaired NMIIA activation. Upon stimulation, LRBA-KO cells displayed dysregulated pMLC dynamics and failed to sustain NMIIA activation over time. These defects likely contribute to the impaired IS formation and antigen internalization observed in LRBA-deficient cells.

These results are shown below in **Reviewer Figure R10** and are now included in the manuscript as **Figure 3I,** and in the **Result section (page 9, line 222-226)** and the **Discussion (page 15, line 381-383)** in the revised manuscript.

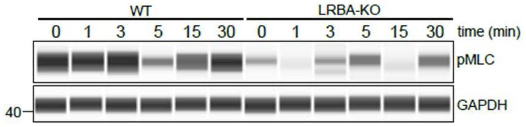

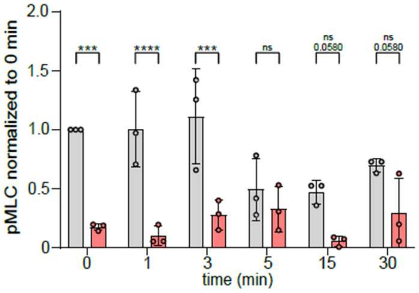

Reviewer Figure R10 and Figure 3I. *Phosphorylation of MLC was measured in WT (grey) and LRBA-KO (red) Ramos B cells after stimulation with 5 µg/ml anti-IgM for the indicated time points. Left: Representative immunoblot analyses of pMLC expression of one experiment. GAPDH was used as a housekeeping protein. Right: Densitometry analyses of pMLC immunoblots normalized first to GAPDH and then to pMLC WT 0 min. Each dot represents one biological replicate, while bars represent mean ± SD of n=3 biological replicates. Statistical analysis of all experiments was performed using Two-way ANOVA with Holm-Sidaks multiple comparison test, ***p<0.001 (0min: p=0.0003, 3min: p=0.0002), ns: not significant.*

We also evaluated the spatial co-localization of LRBA and pMLC during cell migration in microchannels and immune synapse formation with anti-IgM coated beads. In microchannels, LRBA predominantly localized to the cell rear and the perinuclear region in HD LCL cells. Similarly, pMLC was mainly enriched at the rear of the cell in HD LCL cells; however, this polarized distribution was lost in LRBA-deficient cells, where pMLC exhibited a more uniform distribution between front and rear. Co-localization analysis revealed a high Pearson correlation between LRBA and pMLC, which indicates that LRBA associates with the active form of NMIIA during cell migration. Similarly, during immune synapse formation, LRBA showed limited co-localization with total MYH9 but preferentially co-localized with active NMIIA (pMLC) at the IS. In addition, pMLC intensity was reduced in LRBA-deficient Ramos B cells. Overall, these findings indicate that LRBA preferentially co-localizes with the active form of NMIIA and may contribute to its activation.

The results for LRBA and pMLC during migration are shown below in **Reviewer Figure R11** and are now included in the manuscript as **Figure 2, F and G,** and in the **Result section (page 8, line 187-189)** and the **Discussion (page 13 and 14, line 339-341)** in the revised manuscript. The results for LRBA and pMLC during immune synapse formation are shown below in **Reviewer Figure R12** and are now included in the manuscript as **Figure 4 H-K,** and in the **Result section (page 10 and 11, line 264-267)** and the **Discussion (page 15, line 383-384)** in the revised manuscript.

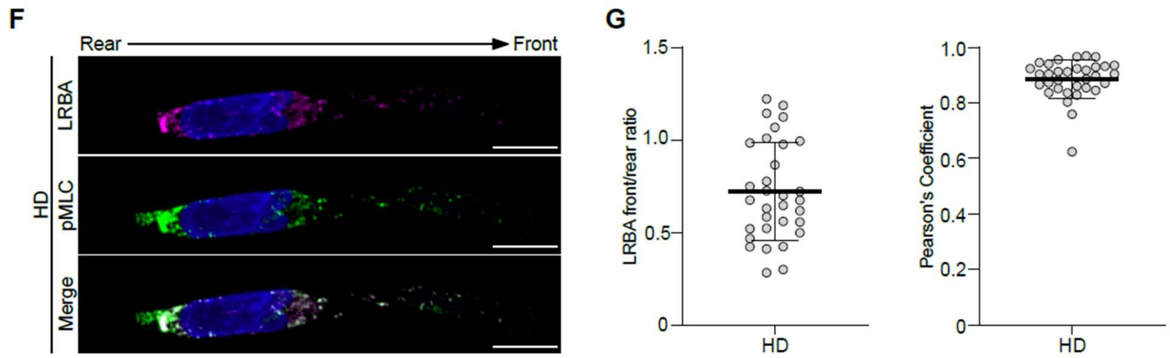

Reviewer Figure R11 and Figure 2, F and G. (F) *Representative confocal microscopy images of HD LCL cells which were let to migrate in microchannels, fixed and stained for LRBA (magenta), pMLC (green) and Hoechst (blue). Scale bar: 10 µm.* ***(G)*** *Analysis of LRBA distribution between the front and rear in HD (grey) LCL cells (left) and co-localization of pMLC and LRBA shown as Pearson's coefficient (right). Each dot represents one cell (Front/rear: n=32, Pearson: n=33) from n=3 biological replicates, mean ± SD shown by the black line and error bars.*

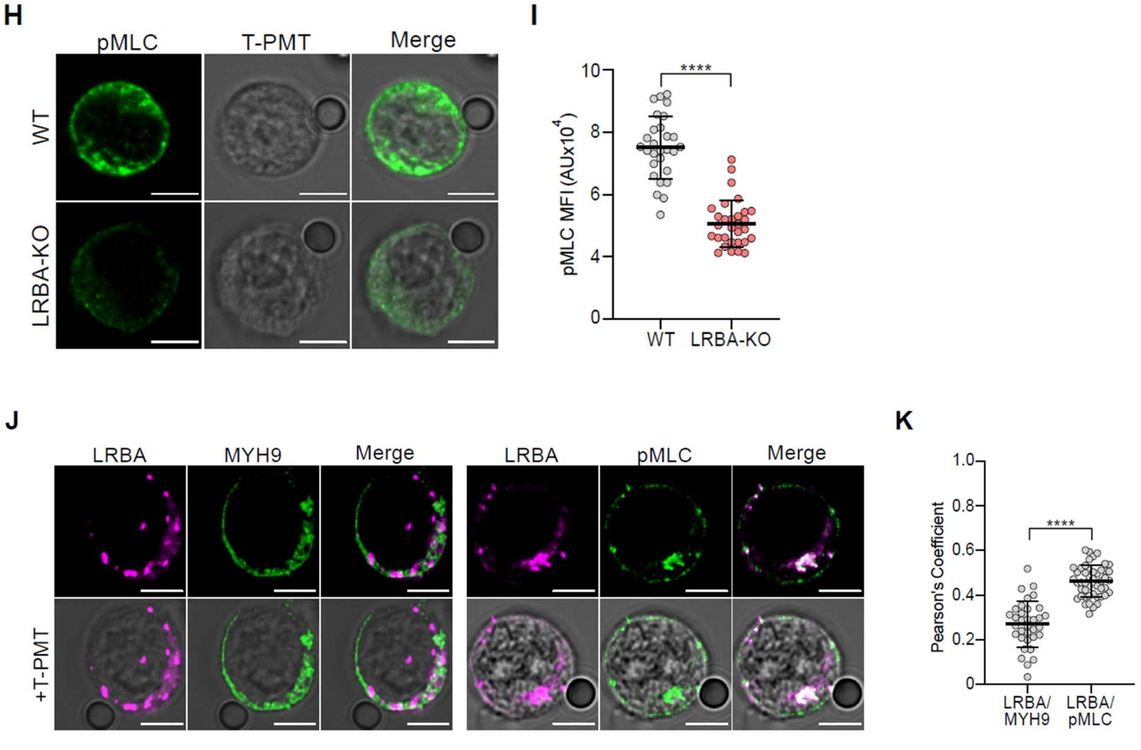

Reviewer Figure R12 and Figure 4, H-K. *IS analysis of WT (grey) and LRBA-KO (red) Ramos B cells after incubation with anti-IgM-coated 3 µm beads for 30 min.* ***(H)*** *Representative confocal microscopy images of Ramos cells in transmitted-light (T-PMT) and stained for pMLC (green). Scale bar: 5 µm.* ***(I)*** *Analysis of pMLC MFI of WT (grey) and LRBA-KO (red) Ramos B cells. Each dot represents one cell (WT: n=28, KO: n=30) from n=3 biological replicates, mean ± SD shown by the black line and error bars.* ***(J)*** *Representative confocal microscopy images of Ramos WT cells stained for MYH9 or pMLC (green) and for LRBA (magenta). Top panel is shown without and bottom panel with transmitted-light (T-PMT). Left:*

LRBA and MYH9. Right: LRBA and pMLC. Scale bar: 5 µm. ***(K)*** *Co-localization of MYH9 and LRBA and pMLC and LRBA shown as Pearson's coefficient. Each dot represents one cell (MYH9: n=34, pMLC:n=47) from n=3 biological replicates, mean ± SD shown by the black line and error bars. Statistical analysis was performed using Welch's t-test, ****p<0.0001.*

2.2 The roles of NMIIA and the actin cytoskeleton in cell migration have been well studied. If the authors can examine the spatial relationships of LRBA, NMIIA, and F-actin during B cell migration using immunofluorescence, the resulting data will provide clues as to whether LRBA regulates B cell migration through NMIIA or F-actin.

We thank the reviewer for this comment and agree that analyzing the spatial relationships of LRBA, NMIIA and F-actin is important to better understand the role of LRBA in B cell migration.

To address this, we analyzed the distribution of F-actin and pMLC in LCL cells migrating in microchannels. For this, LCL cells were let to migrate, fixed and stained with Phalloidin (F-actin) and anti-pMLC. Our results showed that HD LCL cells had uniformly distributed F-actin between the front and the rear and pMLC was enriched at the cell rear. In contrast, LRBA-deficient cells showed an accumulation of F-actin at the cell front and a uniform distribution of pMLC between front and rear of the cell, indicating loss of spatially restricted contractility and a disrupted cytoskeletal polarity. Together, the front-enriched F-actin and the loss of rear-polarized pMLC in LRBA-deficient cells provide evidence of impaired front-rear cytoskeletal symmetry, likely contributing to the more rapid migration in microchannels. When we stained for LRBA we identified that it predominantly localizes to the cell rear and the perinuclear region and showed high co-localization with pMLC **(Reviewer Figure R11).**

We therefore suggest that LRBA regulates B cell migration by coordinating both F-actin organization and NMIIA-dependent contractility.

These results are shown below in **Reviewer Figure R13** and are now included in the manuscript as **Figure 2, D-E** and in the **Result section (page 7 and 8, line 182-187)** and **Discussion (page 13, line 338-339)** in the revised manuscript.

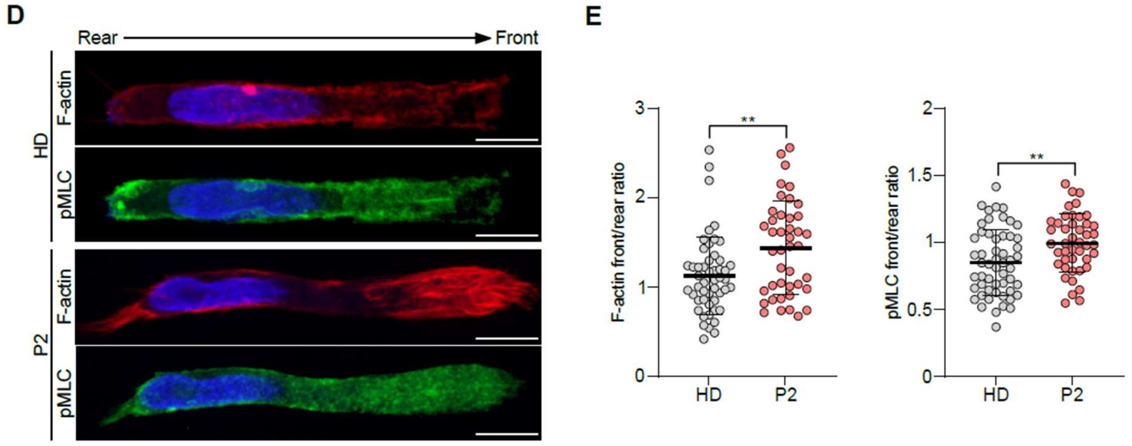

Reviewer Figure R13 and Figure 2, D and E. (D) *Representative confocal microscopy images of HD and LRBA-P2 LCL cells which were let to migrate in microchannels, fixed and stained for F-actin using Phalloidin (red), pMLC (green) and Hoechst (blue). Scale bar: 10 µm.* ***(E)*** *Analysis of F-actin (left) and pMLC (right) distribution between the front and the rear in HD (grey) and LRBA-P2 (red) LCL cells. Each*

*dot represents one cell (F-actin: HD: n=51, P2: n=42; pMLC: HD: n=54, P2: n=44) from n=3 biological replicates, mean ± SD shown by the black line and error bars. Statistical analysis was performed using Welch's t-test, **p<0.01 (E-F-actin: p=0.0034, E-pMLC: p=0.0028, H: p=0.0072).*

2.3. Fig. 2C is confusing. It is unclear what white dashes, their lengths, and their spacing represent.
We apologize for the lack of clarity in this figure. LCL migration in microchannels was recorded by time-lapse microscopy, and space-time kymographs were generated for each microchannel to visualize cell movement in a single image. In these kymographs, nuclear position (in white) of a representative cell migrating inside the microchannel across the field of view (10x objective) is represented in the x-axis (distance in µm). Sequential images were placed one below the other (vertical axis). The interval between two images is 2 minutes. Hence, the slope of nuclear position over time allows us to determine the cell speed (steeper slopes indicate faster movement). For HD, P2 and P3, the movement of a single cell is depicted **(now Figure 2B)**. The spacing between consecutive dashes reflects the distance travelled by the cell between imaging frames.

To improve clarity on the kymographs, we have updated the **figure legend of Figure 2B (page 49, line 1261-1265).**

2.4. Fig. 2F is unclear. If ~60% of LRBA-deficient B cells can pass the restricted area shown in the bar graph on the right, why does the diagram on the left show LRBA-deficient B cells stuck in the narrow area? Did LRBA-deficient cells move more slowly than WT cells or get stuck in the narrow area? Please add a time scale to the diagram.
We thank the reviewer for this comment and apologize for the lack of clarity in Figure 2F **(now Figure 2H)**. The schematic illustration on the left is intended to depict the experimental setup and exemplify the behaviour of a "passing" cell and a "non-passing" cell, rather than reflecting the average behaviour of healthy donor (HD) and LRBA-deficient LCL cells. In this assay, cells migrate through microchannels containing 3 µm constrictions, and the percentage of cells that successfully pass the constriction is quantified (bar graph, right). While most HD cells pass efficiently, approximately 40% of LRBA-deficient cells fail to enter the constriction and instead retreat. This means that the remaining 60% of evaluated LRBA-deficient cells are able to pass through the constriction.
Importantly, this assay measures passage frequency rather than transit time. Although LRBA-deficient cells migrate faster under non-constricted conditions than HD cells (Figure 2, B and C), they exhibit impaired ability to deform and pass through narrow constrictions, indicating a defect in cellular and nuclear deformability rather than intrinsic motility.

To improve clarity, we have modified the schematic illustration on the left to depict "passing" and "non-passing" cells instead of labelling them as HD and LRBA-Pt. This is now shown below as **Reviewer Figure R14** and updated in the manuscript as **Figure 2H** and we updated **the Figure Legend of 2H (page 50, line 1281-1284)**.

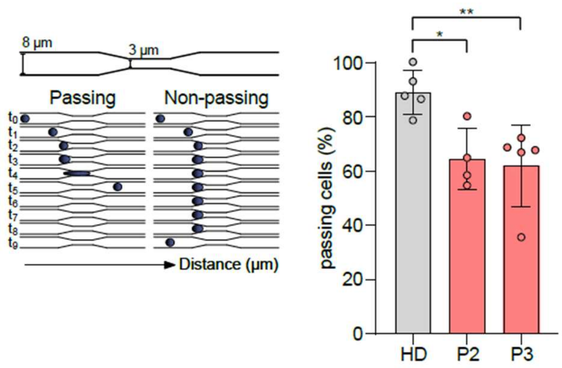

Reviewer Figure R14 and Figure 2H. *Left: Schematic representation of cell migration in microchannels containing 3 µm constrictions, illustrating the two observed behaviours: a passing cell that deforms and crosses the constriction, and a non-passing cell that repeatedly attempts to access the constriction but fails to enter and eventually retreats. Right: Percentage of HD (grey) and LRBA-P2 and P3 LCL cells (red) that migrated through constrictions. Each dot represents the mean of one biological replicate, while bars represent mean ± SD of n=4–5 biological replicates. Statistical analysis was performed using a Two-way ANOVA with Tukey's multiple comparisons test, *p<0.05 (p=0.0120), **p<0.01 (p=0.0072).*

2.5. Based solely on the reduced phalloidin staining levels in LRBA knockout cells, the authors concluded that LRBA-deficiency reduces actin polymerization. However, increased actin depolymerization can also reduce phalloidin staining. This possibility is not excluded in this manuscript. We thank the reviewer for raising this concern, and we agree that reduced phalloidin staining alone cannot distinguish between decreased F-actin polymerization and increased F-actin depolymerization. However, the time-course analysis of F-actin dynamics upon BCR stimulation shown in Figure 3, A and B, provides kinetic evidence that supports impaired F-actin polymerization rather than increased F-actin depolymerization in LRBA-KO cells over time. In detail, WT Ramos B cells showed rapid F-actin accumulation upon BCR stimulation, peaking at 15 and 30 seconds. LRBA-KO Ramos B cells also showed stimulation-induced F-actin accumulation, indicating that polymerization is initiated; however, the magnitude of accumulation was consistently reduced at all time points. Importantly, we did not observe a transient increase followed by accelerated decline, which would be expected if increased depolymerization were the primary defect. Instead, this kinetics support impaired F-actin accumulation, most consistent with reduced polymerization efficiency.

This interpretation is further supported by partial rescue of F-actin levels upon LRBA reconstitution (Figure 4, A-C), and by associated defects in BCR polarization, IS formation, and BCR internalization (Figure 3, G and H; Figure 4 A-C; Figure 5G), which depend on proper F-actin polymerization and actomyosin contractility (Dustin et al., 2019, 31815664; Hoogeboom et al., 2018, PMID: 35493485; Li et al., 2018, PMID: 30687315; Wang et al., 2022, PMID: 35404237).

We acknowledge, however, that our data does not fully exclude the possibility of increased actin depolymerization. Therefore, we have revised the manuscripts text to use more precise terminology, such as reduced F-actin accumulation or F-actin intensity where F-actin intensity was only measured at one time point, which are included in different sections of the revised manuscript **(page 10, line 252), (page 10, line 259), (page 11, line 280), (page 60, line 1511).**

2.6. When stimulating with soluble anti-IgM antibodies, surface BCRs cluster and move to one pole of a cell, driven by polarizing actin polymerization, in primary mouse B cells (Liu et al. J. Immunol. 2012). However, the authors observed only an increase in phalloidin staining, not actin polarization, in WT Ramos cells after stimulation. The authors did not examine surface BCR clustering in response to soluble anti-IgM stimulation either. Therefore, it is unclear whether B cells were sufficiently activated to reorganize the actin cytoskeleton. If Ramos cells do not reorganize the actin cytoskeleton due to their lymphoma nature, it should be discussed.

We thank the reviewer for this comment and agree that studying BCR clustering after soluble anti-IgM stimulation is critical to evaluate cytoskeletal reorganization. In our revision experiments, we stimulated WT and LRBA-KO Ramos B cells with soluble AlexaFluor647-conjugated anti-IgM for 1 min, 5 min, 15 min, 30 min and 60 min, and then evaluated BCR clustering and F-actin polarization by confocal microscopy. We quantified polarization using a fluorescence polarization index, which measures the displacement of the fluorescence center of mass relative to the cell body. This index ranges from 0 to 1, where higher values indicate polarization of the fluorescence signal towards one side of the cell, and values close to 0 indicate symmetric distribution. We found that WT Ramos B cells exhibited progressive BCR polarization after 15 min post-stimulation reaching the highest polarization at 30 min. In contrast, LRBA-KO Ramos B cells showed delayed polarization, with redistribution only observed at 60 min.

In parallel, analysis of F-actin revealed only modest polarization in both WT and LRBA-KO Ramos B cells, indicating that, unlike primary B cells, Ramos B cells do not exhibit strong actin polarization in response to soluble anti-IgM stimulation but instead maintain a relatively uniform F-actin distribution.

Together, these results confirm that Ramos B cells are responsive to BCR stimulation and capable of receptor clustering, but display limited actin polarization, likely reflecting intrinsic properties of this lymphoma-derived cell line.

These results are shown below in **Reviewer Figure R15** and the BCR polarization is now included in the manuscript as **Figure 3, G and H,** and in the **Result section (page 9, line 216-222)** and **Discussion (page 14, line 364-366)** in the revised manuscript.

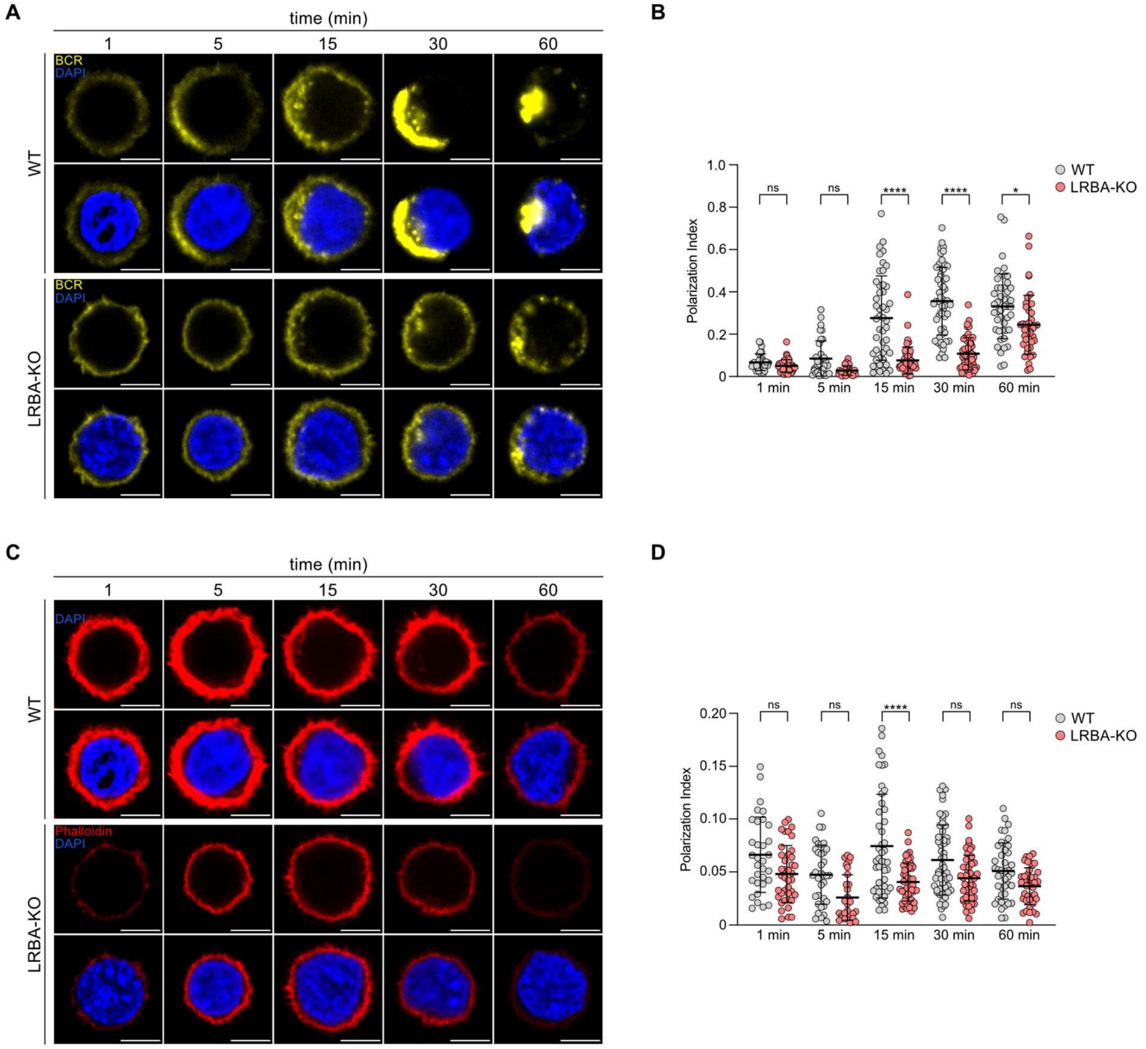

Reviewer Figure R15 and Figure 3, G and H (only BCR). Reduced BCR polarization in the absence of LRBA. (A)-(C) *Representative confocal microscopy images of* ***(A)*** *BCR and* ***(C)*** *F-actin polarization in WT and LRBA-KO cells stimulated with 5 µg/ml AlexaFluor647 anti-IgM for the indicated time points. BCR staining is shown in yellow, F-actin in red and DAPI in blue. Scale bar: 5 µm.* ***(B)-(D)*** *Quantification of the* ***(B)*** *BCR and* ***(D)*** *F-actin polarization index in WT (grey) and LRBA-KO (red) Ramos B cells, which measures the displacement of the fluorescence center of mass relative to the cell body. Positive values indicate polarization of the fluorescence signal towards one side of the cell, whereas values close to 0 indicate symmetric distribution. Each dot represents one cell (WT-1min: n=32, KO-1min: n=40, WT-5min: n=36, KO-5min: n=29, WT-15min: n=48, KO-15min: n=50, WT-30min: n=55, KO-30min: n=49, WT-60min: n=45, KO-60min: n=41), from n=3 biological replicates, mean ± SD shown by the black line and error bars. Statistical analysis was performed using Two-way ANOVA with Holm-Sidaks multiple comparison test, *p<0.05 (B: p=0.0402), ****p<0.0001, ns: not significant.*

2.7. In Fig. 4A, the authors used microtubule organization center (pericentrin) and lysosome (LAMP1) staining to define immunological synapses. First, the authors did not clearly describe how they defined immunological synapses using these two stainings in the results and methods sections. Second, without BCR staining, immunological synapses cannot be defined, as microtubule organization and lysosomes are not components of immunological synapses, but surface BCRs are, even though they polarize towards immunological synapses.

We thank the reviewer for this important clarification and agree that pericentrin (MTOC) and LAMP1 (lysosomes) are not structural components of the immunological synapse but rather markers of cellular polarization associated with synapse maturation. These markers have, however, been widely used as functional readouts of mature B-cell immunological synapse formation (Stinchcombe et al., 2006, PMID: 17006514; Yuseff et al., 2011, PMID: 21820334).

In our experimental setup, B cells were stimulated on anti-IgM-coated surfaces, where engagement of surface BCR with immobilized ligand (anti-IgM) induces synapse formation. Using these conditions, direct visualization of BCR clustering as a discrete structure is technically challenging. Therefore, we defined immunological synapse formation based on coordinated polarization of key cellular components toward the contact site, specifically F-actin (Phalloidin), the centrosome (Pericentrin), and lysosomes (Lamp-1). Cells positive for this coordinated polarization (triple fluorescent signal) were classified as forming a mature immune synapse **(Figure 4 A-C and Expanded View Figure 3 A-C).**

Importantly, we assessed BCR clustering using an independent assay with planar bilayers containing anti-IgM (Figure 5 A-D), where BCR organization can be directly visualized. In addition, during the revision process we incubated Ramos B cells with anti-IgM coated beads and analyzed BCR polarization towards the bead. The data is presented in **Reviewer Figure R17** and **Figure 4, F and G** of the revised manuscript.

To improve clarity, we have revised the **Result section (page 10, line 244-246)** and **Method section (page 26, line 663-668)** to explicitly describe how immunological synapse formation was defined in these experiments (Figure 4, A-C and Expanded View Figure 3, A-C).

2.8. Fig. 4G showed the most convincing data in this manuscript to support that LRBA-deficiency affects actin reorganization towards BCR activation sites, as phalloidin staining under an anti-IgM-coated bead was missing in LRBA-deficient B cells. However, the authors ignored this evidence and only measured the mean fluorescence intensity of phalloidin. Instead of the mean phalloidin fluorescence intensity, the authors should quantify the phalloidin fluorescence intensity ratio under the bead versus the opposite site of the plasma membrane. Again, there was no BCR staining to show the SMAC formation.

We thank the reviewer for this important suggestion and agree that spatial quantification of F-actin is more informative that global intensity measurements. We have therefore evaluated F-actin polarization by calculating the phalloidin fluorescence intensity ratio at the bead contact site relative to the distal side of the cell. This analysis confirmed that WT Ramos B cells exhibit strong F-actin enrichment at the bead interface, whereas LRBA-KO cells show significantly reduced polarization.

These results are shown below in **Reviewer Figure R16** and are now included in the manuscript in **Figure 4E** and in the **Result section (page 10, line 261-264)** of the revised manuscript.

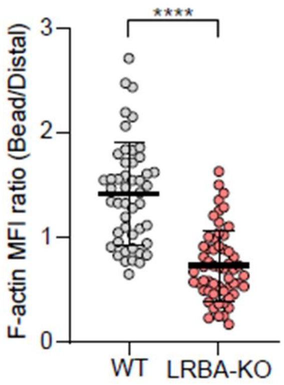

*__Reviewer Figure R16 and Figure 4E (right).__ IS analysis of WT (grey) and LRBA-KO (red) Ramos B cells after incubation with anti-IgM-coated 3 µm beads for 30 min. __(E)__ Quantificiation of the F-actin MFI ratio at the bead vs the distal side of the cell (right). Each dot represents one cell (WT: n=48, KO: n=57) from n=3 biological replicates, mean ± SD shown by the black line and error bars. Statistical analysis was performed using Welch's t-test, ****p<0.0001.*

In addition, we analyzed BCR organization during immune synapse formation by incubating Ramos B cells with anti-IgM-coated beads, followed by fixation and staining with anti-lambda to detect the BCR. BCR redistribution was quantified using a fluorescence polarization index (ranging from -1 to 1), where positive values indicate polarization towards the bead, while negative values indicate BCR distribution at the opposite site of the bead. WT Ramos B cells showed clear BCR polarization toward the bead, consistent with cSMAC formation whereas LRBA-KO cells failed to polarized BCRs to the bead displaying a more uniform distribution.

These results are shown below in **Reviewer Figure R17** and are now included in the manuscript as **Figure 4, F and G,** and in the **Result section (page 10, line 261-264)** and **Discussion (page 14 and 15, line 366-368)** in the revised manuscript.

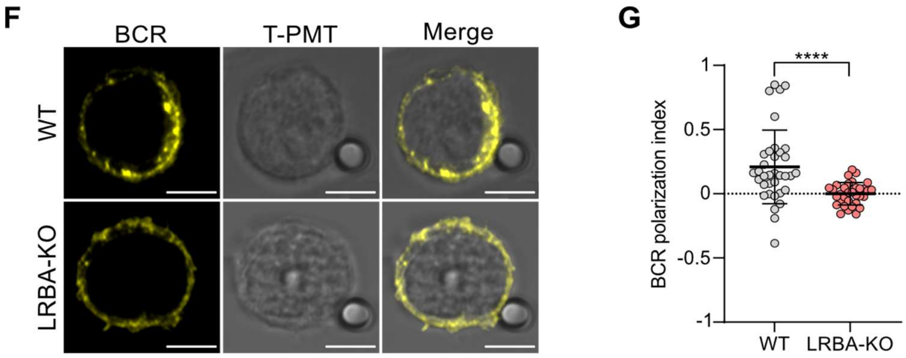

__Reviewer Figure R17 and Figure 4, F and G.__ IS analysis of WT (grey) and LRBA-KO (red) Ramos B cells after incubation with anti-IgM-coated 3 µm beads for 30 min. __(F)__ Representative confocal microscopy images of Ramos cells in transmitted-light (T-PMT) and stained for the BCR using anti-lambda (yellow). Scale bar: 5 µm. __(G)__ Quantification of the BCR polarization index, calculated from the displacement of the fluorescence center of mass relative to the cell center toward the bead-contact site. Positive values

*indicate polarization toward the bead, values near 0 indicate a symmetric distribution, and negative values indicate polarization away from the bead. Each dot represents one cell (WT: n=35, KO: n=32) from n=3 biological replicates, mean ± SD shown by the black line and error bars. Statistical analysis was performed using Mann-Whitney t-test, ****p<0.0001.*

2.9. For Fig. 5, please describe how immunological synapses and closed cSMACs were defined and counted. Phalloidin staining should also be included to support the conclusion. To claim actin dynamic changes in LRBA-deficient cells, multiple early time points should be included.

We thank the reviewer for this clarification request. In Figure 5, immune synapse formation was defined based on cell contact with planar lipid bilayers, as visualized by interference reflection microscopy (IRM). Cells exhibiting a clear, dark-dot like structure at the contact area at the cell-bilayer interface were classified as forming an immune synapse. Closed central supramolecular activation clusters (cSMACs) were identified as a round, punctate accumulations of fluorescence signal at the contact site. The frequency of immune synapse formation and closed cSMACs was calculated as the percentage of cells exhibiting both IRM-defined contact and a closed cSMAC relative to the total number of cells in the field (estimated by DIC imaging). Quantification was initially performed manually using Fiji software and subsequently validated using automated analysis in CellProfiler to minimize bias. To improve clarity on how IS and closed cSMACs were defined, we have now included this information in the **Methods section (page 31 and 32, line 807-814).**

Regarding the phalloidin staining, as shown in **Expanded View Figure 5A**, we evaluated F-actin organization by staining cells with Phalloidin after fixation following 30 min of contact with lipid bilayers. As this approach does not capture the temporal progression of IS formation, we agree with the reviewer that these data do not reflect actin dynamics. We have therefore revised the **Result section (page 11, line 280)** and **Figure legend** of **Expanded View Figure 5A (page 60, line 1511)** to avoid referring to these observations as “F-actin dynamics” and instead describe them as F-actin intensity.

Referee-#3:

This study presents a new explanatory framework for humoral immune deficiencies in LRBA deficiency, by identifying NMIIA as a new interaction partner of LRBA in B cells and showing a role of LRBA in regulating actin cytoskeleton dynamics during B cell activation. This is conceptually appealing because although LRBA deficiency is classically defined as a T-cell/Treg/CTLA-4 trafficking disorder, B cells are also intrinsically affected, and several of their defects map onto processes that normally depend on non-muscle myosin II (NMII), particularly NMIIA.

The study relies somewhat heavily on EBV-transformed LCL cell lines. Although these retain B-cell identity, EBV transformation profoundly remodels the cytoskeletal and trafficking programs that underlie non-muscle myosin II (NMIIA)-dependent antigen extraction. This is largely mitigated by central experiments in Ramos cells, and a few in primary B cells.

It is somewhat hard to fully disentangle primary and secondary effects, i.e., if LRBA deficiency impairs BCR signalling, that could explain some of the defects in SMAC formation and antigen internalization, and if LRBA impairs SMAC formation, this could explain some more of the downstream defects.

However, overall, the study is well presented and the approach and methodology appear sound. The manuscript is well-written, and the figures are for the most part very clear.

Results section:
3.1. It is curious that the SILAC experiment identifies as significant interactors ($p<0.05$) filamin A (FLNA), intersectin-2 (ITSN2), tropomyosin 3 (TPM3) and vimentin (VIM), whereas myosin heavy chain 9 (MYH9) and myosin 18A (MYO18A) although strongly enriched (log2 fold change > 0.82) do not achieve significance ($p<0.13$). Can the authors comment on this?

We thank the reviewer for this observation. While MYH9 and MYO18A showed strong enrichment in our SILAC analysis (log2 fold change > 0.82), they did not reach statistical significance ($p<0.05$) due to variability between the two independent mass spectrometry replicates. This resulted in increased data dispersion and consequently higher p-values despite consistent enrichment trends.

Such discrepancies between fold enrichment and statistical significance are not uncommon in proteomics datasets, particularly when the number of replicates is limited or variability is present (Nesvizhskii et al., 2007, PMID: 17901868; Nesvizhskii, 2012, PMID: 22611043). Therefore, we considered both statistical significance and biological relevance when prioritizing candidates for validation.

Importantly, MYH9 and MYO18A are strongly enriched in our dataset and have well-established roles in cytoskeleton organization, B cell migration, immune synapse formation, and BCR internalization (Seeley-Fallen et al., 2022, PMID: 35493485; Hoogeboom et al., 2018, PMID: 29791846; Matsui et al., 2012, PMID: 21751808; Cheung et al., 2022, PMID: 34001658). Based on this combination of enrichment and biological plausibility, we selected these candidates for further validation by co-IP. More broadly, we consider proteins identified by mass spectrometry as candidate interactors that require independent validation, and we have interpreted the dataset accordingly.

3.2. Their data indicate that LRBA interacts with NMIIA in both healthy donor LCL cells and transfected HEK293T cells, suggesting a role for LRBA in actin cytoskeleton regulation. However, could this not also be shown in primary healthy B cells, potentially after LPS activation?

We thank the reviewer for this suggestion. To validate the LRBA-MYH9 interaction in primary B cells, we isolated B cells from PBMCs from a healthy donor and stimulated them with 250 ng/ml CD40L and 10 µg/ml IL-21 for 24 h. Under these conditions, we confirmed the interaction between LRBA and MYH9 in primary human B cells from a healthy donor. We did not use LPS to stimulate primary B cells because LPS stimulation does not significantly induce LRBA expression in human B cells (Gámez-Díaz et al., 2018, PMID: 29740429).

These results are shown below in **Reviewer Figure R18** and are now included in the manuscript as **Expanded View Figure 1G** and in the **Result section (page 6, line 150-151)** in the revised manuscript.

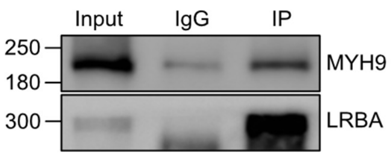

Reviewer Figure R18 and Expanded View Figure 1G. *HD primary B cells were stimulated with 250 ng/ml CD40L and 10 µg/ml IL-21 for 24 h. Immunoprecipitation was performed using anti-LRBA and immunoblotted for MYH9. n=1 biological experiment.*

3.3. The CXCL12-driven chemotactic response would have similarly been interesting to see in primary naïve B cells either LRBA-sufficient or deficient upon LPS stimulation.

We thank the reviewer for this suggestion and agree that it will be very informative to evaluate the CXCL12-driven chemotactic response in primary naïve B cells.

However, our access to patient material is very limited. LRBA-deficient patients in Europe are few, typically have markedly reduced naive B cell numbers, and migration assays require large cell numbers (approximately $3x10^6$ cells per condition), making these experiments not feasible. In contrast, assays such as immune synapse formation and BCR internalization require substantially fewer cells and could therefore be performed in primary cells from patients.

As explained in 3.2, we did not pursue LPS stimulation because LPS stimulation does not significantly induce LRBA expression in human B cells (Gámez-Díaz et al., 2018, PMID: 29740429). In addition, LPS effects differ between species: while it is strongly mitogenic in mouse B cells, in human B cells it primarily promotes differentiation rather than proliferation (Van Bell et al., 2016, PMID: 28116319; Dumont et al., 2009, PMID: 18778289). Therefore, LPS stimulation is unlikely to provide a meaningful comparison between LRBA-sufficient and LRBA-deficient human B cells.

3.4. "Interestingly, evaluation of B cell migration in microchannels with 3 µm constrictions, mimicking an in vivo situation, showed that loss of LRBA progressively reduced the cell passage through micrometric spaces, which is likely attributed to a poor capacity to deform the nucleus (Figure 2F)." The authors should provide a reference, and specify what 'in vivo situation' this is suggested to reflect, e.g., extravasation.

We thank the reviewer for this comment and agree that the “in vivo situation” should be specified more clearly.

The microchannel assay models confined cell migration through dense tissue environments, where cells must deform their nucleus to pass through narrow spaces, such as during interstitial migration

and extravasation (Heuzé et al., 2011, PMID: 21748692; Vargas et al., 2014, PMID: 24637569). Accordingly, we have changed the sentence in the **Results section (page 8, line 189-191)** to clarify this point.

3.5. "Of note, surface and intracellular IgM expression was similar between WT and LRBA-KO cells (Supplementary Figure 2E), demonstrating that reduced BCR response is not due to abnormal IgM expression. Taken together, our data suggest that LRBA is important for regulating F-actin polymerization after BCR stimulation..." Although overall expression does not differ, it is very possible that IgM localization is dysregulated before BCR activation, since the actin and tetraspanin networks have been shown to organize receptor nanoclusters (Mattila et al., Immunity 2013). Did the authors investigate this possibility?

We appreciate the reviewer's comment and agree that BCR organization prior to stimulation should be considered. To address this, we analyzed the surface distribution of IgM in WT and LRBA-KO Ramos B cells under unstimulated conditions. Cells were labeled with an AlexaFluor647-Fab Goat anti-human IgM (Fc5μ) antibody for 30 min at 4°C to prevent receptor internalization, followed by Airyscan confocal microscopy. Images were processed using the Airyscan Processing module (ZEN Blue) followed by AutoQuant Deconvolution (Image-Pro), and a Maximum Intensity Projection was applied across all acquired z-frames.

Quantitative analysis of IgM spatial organization, using the Hopkins index as a measure of clustering, revealed no significant difference between WT and LRBA-KO Ramos B cells. These data indicate that basal surface IgM clustering and thus pre-activated BCR organization is comparable between genotypes.

We acknowledge, however, that Airyscan confocal microscopy operates at the diffraction limit and does not achieve the nanoscale resolution of other techniques such as dSTORM which was used by Mattila et al. (Mattila et al., 2013, PMID: 23499492). We therefore cannot exclude subtle alterations in BCR nanocluster size, density or composition that fall below the resolution threshold. Addressing this would require dedicated super-resolution microscopy approaches.

These results are shown below in **Reviewer Figure R19** and are now included in the manuscript as **Expanded View Figure 3, D and E,** and in the **Result section (page 9, line 227-230)** in the revised manuscript.

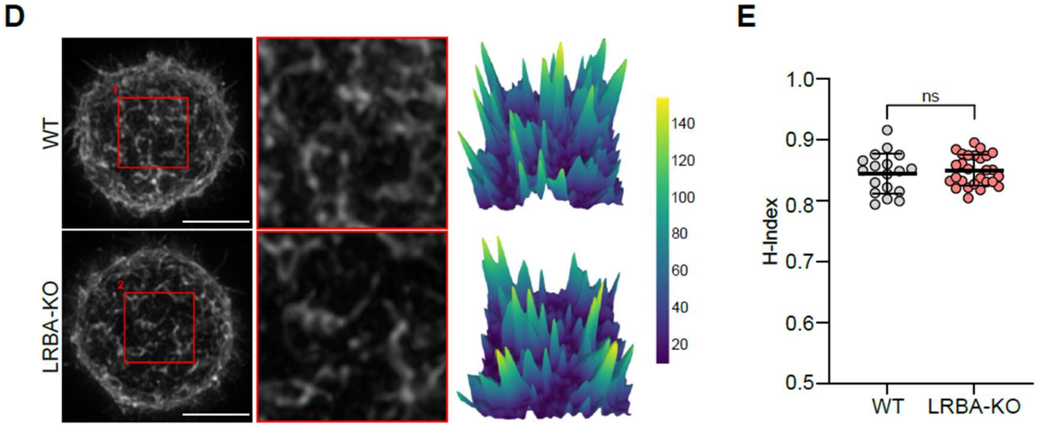

Reviewer Figure R19 and Expanded View Figure 3, D and E. (D-E) *WT and LRBA-KO Ramos B cells were stained with AF647-conjugated anti-IgM Fab fragments, fixed and imaged using confocal microscopy.* ***(D)*** *Representative images of BCR distribution after airyscan processing and maximum intensity projection across the acquired z-stacks (left). Region of interest (ROI) in red square shown as a 2D image (center) and 3D surface plot generated from the ROI with the color-coded z-axis representing the intensity values. Scale bar: 5 µm* ***(E)*** *Quantification of the distribution of the BCR by the Hopkins (H) index in WT (grey) and LRBA-KO (red) Ramos B cells. Each dot represents one cell (WT: n=18, KO: n=27) from n=3 biological replicates, mean ± SD shown by the black line and error bars. Statistical analysis was performed using Welch's t-test, ns: not significant.*

3.6. "we tested the formation of the mature IS in WT and LRBA-KO Ramos B cells stimulated on anti-IgM-coated slides mimicking immobilized antigen presentation..." Because of the lack of lateral mobility of antigen on antigen-coated slides, it is a poor model for IS formation, which relies on lateral mobility. It would have been more proper to employ a planar lipid bilayer system. Can the authors comment on this in relation to their observed IS formation? (I see they used PLB later).

Similarly to the reviewer's opinion, we acknowledge the importance of using planar lipid bilayers as a more physiological system to study immune synapse formation due to the lateral mobility of antigen.

We would like to clarify that we included planar lipid bilayer experiments in the original version of the manuscript **(Figure 5A-D)**. In this system, which allows dynamic BCR clustering and cSMAC formation, we observed reduced contact formation and impaired cSMAC assembly in LRBA-KO cells, indicating that BCR organization is affected in the absence of LRBA.

The experiments using anti-IgM-coated slides **(Figure 4 A–C; Expanded View Figure 4)** were designed to assess polarization of intracellular components (e.g. MTOC, lysosomes, and F-actin) following BCR engagement. While this system lacks antigen mobility, it remains suitable for evaluating downstream polarization events. In addition, anti-IgM-coated beads **(Figure 4D-K)** were used to introduce partial spatial flexibility and better mimic antigen presentation.

Importantly, all three systems, coated slides, beads, and planar lipid bilayers, consistently revealed defects in immune synapse formation in LRBA-deficient cells, including impaired cSMAC formation, defective MTOC and lysosome polarization, and altered F-actin organization. This consistency across different experimental approaches strengthens our conclusion that LRBA is essential for proper immune synapse formation.

3.7. VLA-4 (very late antigen-4, integrin α4β1) undergoes classic inside-out signaling. Can the authors comment on their observation of intact VLA-4 mediated adhesion and contact area by LRBA-KO Ramos cells, given that LRBA interferes with BCR-mediated signals required for inside-out signaling?
We thank the reviewer for highlighting this point. Although LRBA-KO Ramos B cells exhibit reduced BCR signaling **(Figure 3C-F)**, downstream signaling is not completely abolished. We therefore propose that the residual BCR signaling is sufficient to support VLA-4 inside-out activation under the conditions tested. This is consistent with previous findings showing that partial inhibition of BCR signaling is insufficient to fully impair integrin activation (De Gorter et al., 2018, PMID: 29326158).

Under low shear stress, LRBA-KO cells display VLA-4-mediated adhesion comparable to WT cells **(Figure 5, E and F)**, supporting the idea that integrin activation remains largely intact. However, at higher shear stress (1.9 dyn/cm²), WT cells show increased adhesion upon BCR stimulation, whereas LRBA-KO cells exhibit a weaker, non-significant response, suggesting that reduced BCR signaling limits optimal integrin activation under more demanding conditions.

In addition, using planar lipid bilayers containing both anti-IgM and VCAM-1, we observed reduced cSMAC formation in LRBA-KO cells. Notably, among cells that formed an immune synapse, the contact area was comparable between WT and LRBA-KO cells, indicating that VLA-4-mediated adhesion per se is preserved. Instead, LRBA-KO cells showed slightly reduced pSMAC formation which suggests that they fail to properly exclude VLA-4 from the central synapse region, suggesting a defect in integrin spatial organization rather than activation.

Together, these data indicate that while residual BCR signaling is sufficient to drive VLA-4 activation, LRBA deficiency primarily affects cytoskeleton-dependent integrin organization and immune synapse architecture.

This is also included in the **Discussion (page 15, line 368-373).**

Discussion section:
3.8. "Previous reports on reduced migration of LRBA-deficient LCL cells towards Sphingosine-1-phosphate (S1P) with normal S1P internalization may also be caused by the observed actin cytoskeleton abnormalities during migration instead of S1P receptor downstream signalling (Sic et al., 2014)." Can the authors qualify this comment in the light of their finding that amoeboid-type cell motility is actually increased. Were the conditions of the here reinterpreted experiments by Sic et al. such that one would expect a non-amoeboid mode of migration?
We appreciate the reviewer's comment and apologize for the lack of clarity in our original statement. In Sic et al. migration was assessed using transwell assays, similar to our experiments in Figure 2A and Expanded View Figure 2, C and D. While we used CXCL12 as a chemoattractant, Sic et al. used Sphingosine-1-phosphate (S1P) and reported reduced migration of LRBA-deficient cells despite normal S1P internalization. In transwell assays, cells must deform their cell body and nucleus to pass through pores, making migration highly dependent on cytoskeletal remodeling and cellular deformability. In contrast, our microchannel experiments assess spontaneous migration in confined but continuous channels, which better reflect amoeboid-type motility and do not require repeated passage through restrictive pores. Under these conditions, we observed increased migration speed in LRBA-deficient cells.

Taken together, these findings suggest that LRBA deficiency impairs the ability of cells to deform and traverse physical barriers, consistent with reduced migration observed in transwell assays. At the same time, LRBA deficiency alters F-actin and pMLC distribution, leading to disorganized front-rear cytoskeletal polarity during confined migration, which likely contributes to the increased migration speed observed in microchannels **(see Point 1.4, Reviewer Figure R3 and Figure 2, D-E, Result section (page 7 and 8, line 182-187)** and **Discussion (page 13, line 338-339)).**

In addition, we have revised the corresponding paragraph in the **Discussion (page 14, line 341-353)** to improve clarity.

3.9. "LRBA-deficient patients show normal generation of germinal centers", does this not go against the authors own assertion that LRBA is important for BCR signaling, SMAC formation and antigen presentation, mechanisms that are all required for the primary foci that precede GC formation?
We thank the reviewer for this important observation. We acknowledge that our discussion overinterpreted very limited data on germinal center (GC) formation in LRBA deficiency, and we apologize for this overstatement.

To date, preserved GC formation has only been clearly documented in one single LRBA-deficient patient (Al Sukaiti et al., 2017, PMID: 28690850), based on histological analysis of showing lymphoid aggregates with GC-like structures despite agammaglobulinemia. In addition, *Lrba*$^{-/-}$ mice show normal splenic GC formation, isotype switching and affinity maturation after immunization or viral infection (Burnett et al., 2017, PMID: 28611475; Gámez-Díaz et al., 2017, PMID: 28652580), although a more recent study suggests subtle abnormalities, such as reduced GC size in Peyer's Patches compared to wt mice (Flores-Hermebegildo et al., 2024, PMID: 38975349). However, these findings should be interpreted with caution, as mouse models do not fully recapitulate the human phenotype of LRBA deficiency (Gámez-Díaz et al., 2017, PMID: 28652580; Burnett et al., 2017, PMID:28611475). Overall, comprehensive data on GC architecture, dynamics and function in human LRBA deficiency remain limited.

Moreover, our findings do not necessarily contradict the presence of GC structures. The defects we describe in BCR signalling and immune synapse formation are partial rather than complete, and residual signalling may be sufficient to initiate GC formation. However, GC formation *in vivo* is a complex multi-cellular process that unfolds over time and depends on interaction with T follicular helper cells and follicular dendritic cells (Victoria & Nussenzweig, 2022, PMID: 35113731). Therefore, while LRBA-deficient B cells may still participate in GC formation, impaired signaling and synapse formation could affect GC quality and output. This is consistent with the reduced memory B cells, plasma cells, and hypogammaglobulinemia observed in LRBA-deficient patients (Alkhairy et al., 2016, PMID: 26707784; Azizi et al., 2017, PMID: 28512785; Gámez-Díaz et al., 2016, PMID: 26768763; Habibi et al., 2019, PMID: 30995531; Kostel Bal et al., 2017, PMID: 28956255; Taghizade et al., 2023, PMID: 37595759)

Accordingly, included these possibilities about the role of LRBA in GC formation, in the **Discussion (page 16, line 402-414)** of the revised manuscript.

3.10. "Stimulation through the BCR and CD40, in combination with IL-4, triggers the activation of activation-induced cytidine deaminase (AID) necessary for the initiation of class-switch recombination

(Stavnezer et al., 2008)." What is meant by 'activation of AID'? BCR signals combined with CD40L and IL-4 cause increased expression of AID.

We thank the reviewer for highlighting this point and apologize for the imprecise wording. We agree that 'activation' is not the correct term, as stimulation through the BCR, CD40 and IL-4 induces the expression of activation-induced cytidine deaminase (AID). We have therefore changed the sentence in the **Discussion (page 16, line 416-418)** to: *"Stimulation through the BCR and CD40, in combination with IL-4, induces the expression of activation-induced cytidine deaminase (AID), which is required for the initiation of class-switch recombination (Stavnezer et al., 2008)."*

Tables and Figures:

3.11. Table 1: There are many histones and nuclear proteins identified in the SILAC MS analysis. Could the authors comment on the cellular localization/distribution of LRBA?

We thank the reviewer for raising this point. The presence of histones and other nuclear proteins in SILAC-MS datasets is a well-known phenomenon and often reflects contaminants or non-specific background. The CRAPome database, which collects negative control data from affinity purification-MS studies, identifies histones among the most frequently detected contaminants due to their high abundance and propensity for non-specific binding (Mellacheruvu et al., 2013, PMID: 23921808).

LRBA is predominantly localized in the cytoplasm, where it associates with multiple membrane compartments, including the ER, the trans golgi network (TGN), early and late endosomes, recycling endosomes, lysosomes, autophagosomes, and secretory lysosomes of cytotoxic cells (Reiser et al., 2014; Jaramillo et al., 2018, PMID: 30410199; Szentjyörgyi et al., 2024, PMID: 39325073). Consistent with this, our PLA (Figure 1E) and immunofluorescence staining (Expanded View Figure 4E) confirm cytoplasmic localization of LRBA in our system.

Although LRBA has been found in the nucleus of mouse bone marrow cells upon LPS stimulation (Reiser et al., 2014), we did not observe nuclear localization in Ramos B cells. Therefore, we consider the detection of nuclear proteins in our dataset to most likely reflect background rather than specific LRBA interactions.

3.12. Figure 1: The blue-line-identified cut-off is quite non-standard. Can the authors comment on this?

We thank the reviewer for this comment and agree that the blue-line cutoff in Figure 1B does not represent a conventional statistical significance threshold. Our SILAC-IP-MS experiment was performed with two biological replicates and was intended as a hypothesis-generating screen. Therefore, rather than applying a strict significance cutoff alone, we considered both effect size (fold enrichment) and p-value. The blue line indicates an empirically defined "candidate zone" that includes strongly enriched proteins near the significance threshold ($p < 0.13$), which were prioritized for follow-up validation based on their biological relevance, particularly their association with cytoskeleton processes.

We have therefore updated our **Result section (page 6, line 142-145)** and **Figure legend of Figure 1B (page 48, line 1234-1239)** to clarify this cut-off.

3.13. The authors reference a study showing interaction of LRBA with AID by co-IP, an interaction not identified here. This is likely because the LCL subjected to this analysis do not express AID. The authors should discuss the limitations of the applied methodology in terms of what is expressed in their LCL at 'resting' conditions, compared to potential important interactors in e.g., activated or GC B cells.

We thank the reviewer for raising this point regarding the limitations of our proteomics approach. We agree that the use of LCL cells may restrict the identification of certain LRBA interactors that are expressed in naive B cells but not in LCL cells, and that may also be expressed under different cell stimulation conditions. In particular, AID is not expressed in LCLs under resting conditions, which likely explains why this interaction was not detected in our dataset.

In addition, we also did not identify known LRBA interactors, such as FYCO1, UVRAG, or PIK3R4 in our proteomics data, illustrating that MS-based approaches do not capture the full interactome. Hence, we agree with the reviewer that comprehensive analysis of LRBA interaction partners across different B cell subsets, different stimulation conditions, and different time points would provide a more complete understanding of LRBA function and LRBA complexome in B cells. Complexome profiling using cryoslicing blue-native MS analysis (Schulte et al., 2023, PMID: 36697829) of different B cell differentiation stages (naïve, activated, GC, memory, plasma cells) could also help dissect stage-specific LRBA functions and better explain the humoral immune phenotype of LRBA deficiency. However, such an extensive proteomics study is beyond the scope of the current manuscript.

We have included this limitation in our **Discussion (page 18, line 448-452).**

Minor points:

3.14. 177-181: It should be made clear in the text what cell type(s) are referred to here, i.e., LCL and/or Ramos.

We thank the reviewer for this comment and have indicated the cells used. In this case, we used LCL cells to study migration in constricted microchannels. We have updated the **Result section (page 8, line 189-191)** and included that LCL cells were used here to study migration.

3.15. 194: "after IgM stimulation", should be: 'after IgM crosslinking', or 'after anti-IgM stimulation'.

We appreciate the identification of this typo and have changed the wording **page 8 in line 205** from “after IgM stimulation” to “after anti-IgM stimulation”.

3.16. 301-302: "Similarly, loss of LRBA in B cells exhibited distinct migration behaviours depending on the analysed environment." Should be rephrased, e.g., 'Similarly, upon loss of LRBA, B cells exhibited distinct migration behaviours depending on the analysed environment.'

We thank the reviewer for the finding and changed the sentence in **the Discussion (page 13, line 335-336)** to:

“Similarly, LRBA-deficient B cells exhibited distinct migration behaviours depending on the analysed environment.”

3.16. In Figure 4B, E, H, please place the mean/median bars + errors on top of the individual data points. As it is now, it is not possible to see.
This is actually the case for several of the subsequent figures as well.

We thank the reviewer for this important point and apologize for this issue. We have updated all figures and placed the mean bars + errors on top of the individual data points in each scatter plot.

1st Revision - Editorial Decision 18th May 2026

Dear Dr. Sindram,

Thank you for the submission of your revised manuscript to our editorial offices. I have now received the reports from the three referees that were asked to re-evaluate the study, you will find below. As you will see, the referees now fully support the publication of the study in EMBO reports. Referee #3 has two remaining minor concerns, I ask you to address in a final revised version. Please also provide a final p-b-p-response to these points and the editorial requests below.

Editorial requests:

- Please provide a final title with not more than 100 characters (including spaces) without comma.

- There is a name discrepancy. It is Montero-Hernández on the manuscript title page and Montero-Hermández in the submission system. Please check and make sure that in both places the same name is used.

- Please provide the abstract written in present tense throughout.

- Please order the manuscript sections like this, using only these names:
Title page - Abstract - Keywords - Introduction - Results - Discussion - Methods - Data availability section - Acknowledgements (please include here all the funding information) - Disclosure and Competing Interests Statement - References - Figure legends - Expanded View Figure legends

Thus, please combine the section 'Funding' with the acknowledgements.

- We now use CRediT to specify the contributions of each author in the journal submission system. CRediT replaces the author contribution section. Please use the free text box to provide more detailed descriptions and do NOT provide your final manuscript text file with an author contributions section. See also our guide to authors (section 'Author contributions'):
https://link.springer.com/journal/44319/submission-guidelines#cms-Revised-submissions

- Please check again that the number "n" for how many independent experiments were performed, their nature (biological versus technical replicates), the bars and error bars (e.g. SEM, SD) and the test used to calculate p-values is indicated in the respective figure legends (main and EV figures). Please also check that all the p-values are explained in the legend, and that these fit to those shown in the figure. Please provide statistical testing where applicable. Please avoid the phrase 'independent experiment' but clearly state if these were biological or technical replicates. Please also indicate (e.g. with n.s.) if testing was performed, but the differences are not significant. In case n=2, please show the data as separate datapoints without error bars and statistics. See also:

https://link.springer.com/journal/44319/submission-guidelines#cms-Figure-and-data-presentation

If n<5, please show single datapoints for diagrams. Moreover:
- Please note that the exact p values are not provided in the legend of figure 3B
- Please indicate the statistical test used for data analysis in the legend of figure 1C

- Please use our reference format:
https://link.springer.com/journal/44319/submission-guidelines#cms-Reference-guidelines

Et al needs to be used after 10 author names; DOIs should only be used for preprints and datasets that have not been published yet.

- Please confirm that for all Western blot panels shown the loading control was run on the same gel as the other proteins detected. Please note that we discourage comparisons between samples on different gels/blots, even if the samples derive from one experiment, as confounding factors reduce comparability. If unavoidable, the figure legend must state that the samples derive from the same experiment and that gels/blots were processed in parallel. If a 'representative' loading control is shown for multiple gels/blots, the intra-gel controls should be shown in the source data files, and the figure legends should describe the data displayed accurately. See our author guidelines:

https://link.springer.com/journal/44319/submission-guidelines#cms-Figure-and-data-presentation (section 'Electrophoretic gels and blots').

- The nomenclature for the EV figures is not correct. It should be 'Figure EVx' in the legends and the callouts. Please make sure the correct and complete name is used throughout the manuscript .

- Table 1 is a dataset. Please name this 'Dataset EV1' and upload it as a dataset file in excel format with a legend on the first

TAB. Then please update all callouts and remove the table from the manuscript text file.

In addition, I would need from you uploaded separately:
- a short, two-sentence summary of the manuscript (not more than 35 words).
- two to four short (!) bullet points highlighting the key findings of your study (two lines each).
- a schematic summary figure as separate file that provides a sketch of the major findings (not a data image) in jpeg or tiff format (with the exact width of 550 pixels and the exact height of 300 pixels) that can be used as a visual synopsis on our website.

I look forward to seeing a new revised version of your manuscript as soon as possible.

Best,

Achim Breiling
Senior Editor
EMBO Reports

Referee #1:

The authors responded to all my comments very satisfactorily. I would like to congratulate them on the considerable work they have done, which strengthens the conclusions of this excellent work.

Referee #2:

The authors have made extensive revisions and provided additional data and clarifications. The revisions have addressed most of the previous concerns.

Referee #3:

This is a very comprehensive response, and all my major concerns have been addressed. It was not fully possible to elucidate the IgM distribution with the Airy-scan approach, but I consider this a minor shortcoming, which should not preclude publication.

I have only two minor points remaining:

1. For the revision experiment in Figure 4F and G, it is strange that there is essentially no polarization of BCRs to the anti-IgM coated beads. Some degree of polarization would be expected even based on the passive binding of BCRs by the anti-IgM on the bead surface, but no such effect is seen.

2. The response to point 3.7: "... This is consistent with previous findings showing that partial inhibition of BCR signaling is insufficient to fully impair integrin activation (De Gorter et al., 2018, PMID: 29326158)." It is unclear what study they are referring to here, as this does not match the given PMID. Please clarify.

Point-by-point reply

Referee #3:

This is a very comprehensive response, and all my major concerns have been addressed. It was not fully possible to elucidate the IgM distribution with the Airy-scan approach, but I consider this a minor shortcoming, which should not preclude publication.

I have only two minor points remaining:

1. For the revision experiment in Figure 4F and G, it is strange that there is essentially no polarization of BCRs to the anti-IgM coated beads. Some degree of polarization would be expected even based on the passive binding of BCRs by the anti-IgM on the bead surface, but no such effect is seen.
We thank the reviewer for this comment. In our assay, BCR polarization was quantified using a Polarization Index (PI) that measures the net directional redistribution of the BCR signal across the whole cell, normalized to cell radius (Reversat et al., 2015, PMID: 25631815). Generating a measurable PI shift requires not only enrichment of the BCR at the bead-contact site but also a corresponding depletion at the distal pole (as observed in WT cells). Passive BCR engagement by anti-IgM on the bead surface is insufficient to drive this net redistribution, as i) it locally immobilizes receptors at the contact site without depleting the distal membrane, and ii) even local engagement requires active cytoskeletal remodelling to generate a polarization response, as it must overcome the cortical actin barrier, which in its basal state restricts lateral BCR diffusion across the plasma membrane. This remodelling is precisely what is impaired in LRBA-KO cells, consistent with the reduced F-actin polymerization and NMIIA activation shown in our study. Consequently, most LRBA-KO cells exhibit a relatively uniform BCR distribution and fail to show consistent directional enrichment toward the bead or depletion at the distal pole, resulting in PI values close to zero. Although a subset of LRBA-KO cells displays positive PI values, indicating that limited polarization can occur in individual cells, the overall response remains weak and heterogeneous.

A similar defect in BCR polarization toward anti-IgM-coated beads has previously been reported in Atg5-deficient B cells (Arbogast et al., 2019, PMID: 30196744), although the polarization index in that study was calculated differently, based on the relative angle between the cell center of mass and the extremes of the BCR staining distribution.

2. The response to point 3.7: "... This is consistent with previous findings showing that partial inhibition of BCR signaling is insufficient to fully impair integrin activation (De Gorter et al., 2018, PMID: 29326158)." It is unclear what study they are referring to here, as this does not match the given PMID. Please clarify.
We apologize for the incorrect reference cited in our response to point 3.7. The correct reference supporting our statement is Tissino et al. (2018), which demonstrates that partial inhibition of BCR signaling by treating cells with Ibrutinib (BTK inhibitor) is insufficient to fully impair integrin activation (PMID: 29301866).

Editorial requests:

- Please provide a final title with not more than 100 characters (including spaces) without comma.
We have updated the final title to:
LRBA regulates actin cytoskeleton dynamics through NMIIA during B cell immune responses

- There is a name discrepancy. It is Montero-Hernández on the manuscript title page and Montero-Hermández in the submission system. Please check and make sure that in both places the same name is used.

We apologize for the typo. The correct spelling is Montero-Hernández, which has been updated in the submission system to match the manuscript title page.

- Please provide the abstract written in present tense throughout.
Thank you for the indication. We have updated the abstract to present tense.

- Please order the manuscript sections like this, using only these names: Title page - Abstract - Keywords - Introduction - Results - Discussion - Methods - Data availability section - Acknowledgements (please include here all the funding information) - Disclosure and Competing Interests Statement - References - Figure legends - Expanded View Figure legends

Thus, please combine the section 'Funding' with the acknowledgements.
We have updated the order of the manuscript and combined the funding section with the acknowledgements.

- We now use CRediT to specify the contributions of each author in the journal submission system. CRediT replaces the author contribution section. Please use the free text box to provide more detailed descriptions and do NOT provide your final manuscript text file with an author contributions section. See also our guide to authors (section 'Author contributions'): https://link.springer.com/journal/44319/submission-guidelines#cms-Revised-submissions

We have removed the author contributions section from the manuscript and entered the CRediT contributions in the submission system.

- Please check again that the number "n" for how many independent experiments were performed, their nature (biological versus technical replicates), the bars and error bars (e.g. SEM, SD) and the test used to calculate p-values is indicated in the respective figure legends (main and EV figures). Please also check that all the p-values are explained in the legend, and that these fit to those shown in the figure. Please provide statistical testing where applicable. Please avoid the phrase 'independent experiment' but clearly state if these were biological or technical replicates. Please also indicate (e.g. with n.s.) if testing was performed, but the differences are not significant. In case n=2, please show the data as separate datapoints without error bars and statistics. See also:

https://link.springer.com/journal/44319/submission-guidelines#cms-Figure-and-data-presentation

Thank you for the comment. We have revised all figure legends throughout the manuscript to clearly state the number of biological and technical replicates, the type of error bars, and the statistical tests used. Non-significant comparisons are indicated with ns. For Figure 3B, where n=2 biological replicates were analyzed, we removed statistical testing and now display the datapoints separately.

If n<5, please show single datapoints for diagrams. Moreover:
- Please note that the exact p values are not provided in the legend of figure 3B
Thank you for the comment. For Figure 3B, we analyzed at least 171 cells per condition; however, these data derive from n=2 biological replicates. In line with the journal's guidelines, we removed statistical testing and error bars from this figure and now display all datapoints separately. Accordingly, no p-values are reported in this figure. The **figure legend of Figure 3B (page 45, line 1212-1216)** has been updated accordingly.

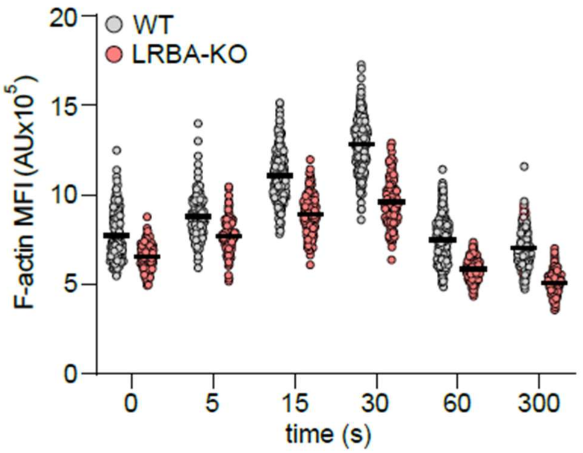

(B) Quantification of F-actin mean fluorescence intensity (MFI) over time in WT (grey) and LRBA-KO (red) Ramos B cells. Each dot represents one cell (WT-0s: n=241, KO-0s: n=205, WT-5s: n=171, KO-5s: n=214, WT-15s: n=222, KO-15s: n=199, WT-30s: n=287, KO-30s: n=189, WT-60s: n=341, KO-60s: n=334, WT-300s: n=336, KO-300s: n=264), while the black horizontal line represents the mean from n=2 biological replicates.

- Please indicate the statistical test used for data analysis in the legend of figure 1C

Thank you for the comment and we apologize for the lack of clarity. For Figure 1C, the GO enrichment analysis was performed on the 33 significantly enriched proteins identified in the SILAC-IP-LC-MS/MS screen shown in Figure 1B using the PANTHER overrepresentation test within Genontology.org (release 38.560). Enrichment p-values were determined by Fisher's exact test against the Homo sapiens reference list and adjusted to Benjamini-Hochberg FDR correction. The bars represent the FDR-corrected p-values visualized as -$\log_{10}$ transformed values. No additional statistical test was performed between GO terms.

For clarification, we have updated the Figure legend of **Figure 1C (page 43, line 1152-1155)** and added an explanation to the **Methods (page 21, line 513-518)**.

- Please use our reference format: https://link.springer.com/journal/44319/submission-guidelines#cms-Reference-guidelines

Et al needs to be used after 10 author names; DOIs should only be used for preprints and datasets that have not been published yet.

Thank you for the indication. We have updated the reference format according to the EMBO reports guidelines.

- Please confirm that for all Western blot panels shown the loading control was run on the same gel as the other proteins detected. Please note that we discourage comparisons between samples on different gels/blots, even if the samples derive from one experiment, as confounding factors reduce comparability. If unavoidable, the figure legend must state that the samples derive from the same experiment and that gels/blots were processed in parallel. If a 'representative' loading control is shown for multiple gels/blots, the intra-gel controls should be shown in the source data files, and the figure legends should describe the data displayed accurately. See our author guidelines: https://link.springer.com/journal/44319/submission-guidelines#cms-Figure-and-data-presentation (section 'Electrophoretic gels and blots').

Thank you for this comment. We confirm that for all conventional Western blot panels the loading control was run on the same gel as the protein of interest (Figure 1D, Figure EV1A–G, Figure EV2A, Figure EV3A). For the Simple Western Automated (WES)-based analyses in Figure 1F and Figure 3I, the

samples derived from the same experiment but the loading control signals were obtained in separate capillaries within the WES system.

For the WES-based analysis, we have added a statement clarifying this to the figure legends of **Figure 1F (page 43, line 1163-1165)** and **Figure 3I (page 46, line 1239-1241)**.

- The nomenclature for the EV figures is not correct. It should be 'Figure EVx' in the legends and the callouts. Please make sure the correct and complete name is used throughout the manuscript. We apologize for the incorrect nomenclature. We have updated the manuscript throughout using the term Figure EVx.

- Table 1 is a dataset. Please name this 'Dataset EV1' and upload it as a dataset file in excel format with a legend on the first TAB. Then please update all callouts and remove the table from the manuscript text file.
We have removed Table 1 from the manuscript, renamed it as Dataset EV1, and uploaded it as an Excel file with the legend on the first tab. All callouts in the manuscript have been updated accordingly.

In addition, I would need from you uploaded separately:
- a short, two-sentence summary of the manuscript (not more than 35 words).
- two to four short (!) bullet points highlighting the key findings of your study (two lines each).
- a schematic summary figure as separate file that provides a sketch of the major findings (not a data image) in jpeg or tiff format (with the exact width of 550 pixels and the exact height of 300 pixels) that can be used as a visual synopsis on our website.

We have uploaded the schematic figure and the summary with bullet points as separate files.

Summary:
LRBA interacts with NMIIA to regulate actin cytoskeleton dynamics in B cells, providing a mechanistic basis for the defective humoral immune response in LRBA deficiency

- Loss of LRBA disrupts F-actin and myosin front to rear polarity, leading to abnormal B cell migration

- LRBA deficiency impairs BCR signaling and polarization, immune synapse formation and antigen internalization, likely driven by reduced F-actin polymerization and NMIIA activation

- The disrupted LRBA-NMIIA interaction may contribute to the humoral immune defects observed in LRBA-deficient patients

Dr. Elena Sindram
Institute for Immunodeficiency, Center for Chronic Immunodeficiency (CCI), Medical Center - University of Freiburg
Medical Center - University of Freiburg
Breisacher Strasse 115
Baden-Württemberg 79106
Germany

Dear Dr. Sindram,

Thank you for the submission of your final revised manuscript to our editorial offices. I now went through this and your final p-b-p-response and consider the remaining points by referee #3 and the editorial requests as adequately addressed.

I thus am very pleased to accept your manuscript for publication in the next available issue of EMBO reports. Thank you for your contribution to our journal.

Your manuscript will be processed for publication by EMBO Press. It will be copy edited and you will receive page proofs prior to publication. Please note that you will be contacted by Springer Nature Author Services to complete licensing and payment information.

You may qualify for financial assistance for your publication charges - either via a Springer Nature fully open access agreement or an EMBO initiative. Check your eligibility: https://link.springer.com/journal/44319/how-to-publish-with-us

Should you be planning a Press Release on your article, please notify the production team via this form in order to coordinate publication and release dates: https://forms.cloud.microsoft/e/eH4A8JVP6g

If you have any questions, please do not hesitate to contact the Editorial Office. Thank you for your contribution to EMBO Reports.

Yours sincerely,

Achim Breiling
Senior Editor
EMBO Reports

>>> Please note that it is EMBO Reports policy for the transcript of the editorial process (containing referee reports and your response letter) to be published as an online supplement to each paper. If you do NOT want this, you will need to inform the Editorial Office via email immediately. More information is available here: https://link.springer.com/partners/embo-press/editorial-policies#Peer%20review

EMBO Press Author Checklist

Corresponding Author Name: Elena Sindram	
Journal Submitted to: EMBO Reports	
Manuscript Number: EMBOR-2025-63029V2	

USEFUL LINKS FOR COMPLETING THIS FORM
The EMBO Journal - Author Guidelines
EMBO Reports - Author Guidelines
Molecular Systems Biology - Author Guidelines
EMBO Molecular Medicine - Author Guidelines

Reporting Checklist for Life Science Articles (updated January
This checklist is adapted from Materials Design Analysis Reporting (MDAR) Checklist for Authors. MDAR establishes a minimum set of requirements in transparent reporting in the life sciences (see Statement of Task: 10.31222/osf.io/9sm4x). Please follow the journal's guidelines in preparing your
Please note that a copy of this checklist will be published alongside your article.

Abridged guidelines for figures
1. Data
The data shown in figures should satisfy the following conditions:
- the data were obtained and processed according to the field's best practice and are presented to reflect the results of the experiments in an accurate and unbiased manner.
- ideally, figure panels should include only measurements that are directly comparable to each other and obtained with the same assay.
- plots include clearly labeled error bars for independent experiments and sample sizes. Unless justified, error bars should not be shown for technical
- if n<5, the individual data points from each experiment should be plotted. Any statistical test employed should be justified.
- Source Data should be included to report the data underlying figures according to the guidelines set out in the authorship guidelines on Data

2. Captions
Each figure caption should contain the following information, for each panel where they are relevant:
- a specification of the experimental system investigated (eg cell line, species name).
- the assay(s) and method(s) used to carry out the reported observations and measurements.
- an explicit mention of the biological and chemical entity(ies) that are being measured.
- an explicit mention of the biological and chemical entity(ies) that are altered/varied/perturbed in a controlled manner.
- the exact sample size (n) for each experimental group/condition, given as a number, not a range;
- a description of the sample collection allowing the reader to understand whether the samples represent technical or biological replicates (including how many animals, litters, cultures, etc.).
- a statement of how many times the experiment shown was independently replicated in the laboratory.
- definitions of statistical methods and measures:
  - common tests, such as t-test (please specify whether paired vs. unpaired), simple χ2 tests, Wilcoxon and Mann-Whitney tests, can be unambiguously identified by name only, but more complex techniques should be described in the methods section;
  - are tests one-sided or two-sided?
  - are there adjustments for multiple comparisons?
  - exact statistical test results, e.g., P values = x but not P values < x;
  - definition of 'center values' as median or average;
  - definition of error bars as s.d. or s.e.m.

Please complete ALL of the questions below.
Select "Not Applicable" only when the requested information is not relevant for your study.

Materials

Newly Created Materials	Information included in the manuscript?	In which section is the information available? (Reagents and Tools Table, Materials and Methods, Figures, Data Availability Section)
New materials and reagents need to be available; do any restrictions apply?	Yes	Reagents and Tools Table and Material and Methods

Antibodies	Information included in the manuscript?	In which section is the information available? (Reagents and Tools Table, Materials and Methods, Figures, Data Availability Section)
For **antibodies** provide the following information:<br>- Commercial antibodies: RRID (if possible) or supplier name, catalogue number and or/clone number<br>- Non-commercial: RRID or citation	Yes	Reagents and Tools Table and Material and Methods

DNA and RNA sequences	Information included in the manuscript?	In which section is the information available? (Reagents and Tools Table, Materials and Methods, Figures, Data Availability Section)
**Short novel DNA or RNA including primers, probes**: provide the sequences.	Yes	Reagents and Tools Table and Material and Methods

Cell materials	Information included in the manuscript?	In which section is the information available? (Reagents and Tools Table, Materials and Methods, Figures, Data Availability Section)
**Cell lines:** Provide species information, strain. Provide accession number in repository **OR** supplier name, catalog number, clone number, and/**OR** RRID.	Yes	Reagents and Tools Table and Material and Methods
**Primary cultures:** Provide species, strain, sex of origin, genetic modification status.	Yes	Reagents and Tools Table and Material and Methods
Report if the cell lines were recently **authenticated** (e.g., by STR profiling) and tested for mycoplasma contamination.	Yes	Material and Methods

Experimental animals	Information included in the manuscript?	In which section is the information available? (Reagents and Tools Table, Materials and Methods, Figures, Data Availability Section)
**Laboratory animals or Model organisms:** Provide species, strain, sex, age, genetic modification status. Provide accession number in repository **OR** supplier name, catalog number, clone number, **OR** RRID.	Not Applicable	
**Animal observed in or captured from the field:** Provide species, sex, and age where possible.	Not Applicable	
Please detail **housing and husbandry conditions**.	Not Applicable	

Plants and microbes	Information included in the manuscript?	In which section is the information available? (Reagents and Tools Table, Materials and Methods, Figures, Data Availability Section)
**Plants:** provide species and strain, ecotype and cultivar where relevant, unique accession number if available, and source (including location for collected wild specimens).	Not Applicable	
**Microbes:** provide species and strain, unique accession number if available, and source.	Not Applicable	

Human research participants	Information included in the manuscript?	In which section is the information available? (Reagents and Tools Table, Materials and Methods, Figures, Data Availability Section)
If collected and within the bounds of privacy constraints report on age, sex and gender or ethnicity for all study participants.	Not Applicable	

Core facilities	Information included in the manuscript?	In which section is the information available? (Reagents and Tools Table, Materials and Methods, Figures, Data Availability Section)
If your work benefited from core facilities, was their service mentioned in the acknowledgments section?	Yes	Acknowledgement section

Design

Study protocol	Information included in the manuscript?	In which section is the information available? (Reagents and Tools Table, Materials and Methods, Figures, Data Availability Section)
If study protocol has been **pre-registered, provide DOI in the manuscript**. For clinical trials, provide the trial registration number **OR** cite DOI.	Not Applicable	
Report the **clinical trial registration number** (at ClinicalTrials.gov or equivalent), where applicable.	Not Applicable	

Laboratory protocol	Information included in the manuscript?	In which section is the information available? (Reagents and Tools Table, Materials and Methods, Figures, Data Availability Section)
Provide DOI OR other citation details if **external detailed step-by-step protocols** are available.	Yes	Material and Methods, References

Experimental study design and statistics	Information included in the manuscript?	In which section is the information available? (Reagents and Tools Table, Materials and Methods, Figures, Data Availability Section)
Include a statement about **sample size** estimate even if no statistical methods were used.	Yes	Figure legends
Were any steps taken to minimize the effects of subjective bias when allocating animals/samples to treatment (e.g. **randomization procedure**)? If yes, have they been described?	Not Applicable	
Include a statement about **blinding** even if no blinding was done.	Yes	Material and Methods
Describe **inclusion/exclusion criteria** if samples or animals were excluded from the analysis. Were the criteria pre-established?<br><br>If sample or data points were omitted from analysis, report if this was due to attrition or intentional exclusion and provide justification.	Not Applicable	
For every figure, are **statistical tests** justified as appropriate? Do the data meet the assumptions of the tests (e.g., normal distribution)? Describe any methods used to assess it. Is there an estimate of variation within each group of data? Is the variance similar between the groups that are being statistically compared?	Yes	Figure legends

Sample definition and in-laboratory replication	Information included in the manuscript?	In which section is the information available? (Reagents and Tools Table, Materials and Methods, Figures, Data Availability Section)
In the figure legends: state number of times the experiment was **replicated** in laboratory.	Yes	Figure legends
In the figure legends: define whether data describe **technical or biological replicates**.	Yes	Figure legends

Ethics

Ethics	Information included in the manuscript?	In which section is the information available? (Reagents and Tools Table, Materials and Methods, Figures, Data Availability Section)
Studies involving **human participants**: State details of **authority granting ethics approval** (IRB or equivalent committee(s), provide reference number for approval.	Yes	Material and Methods
Studies involving **human participants**: Include a statement confirming that **informed consent** was obtained from all subjects and that the experiments conformed to the principles set out in the WMA Declaration of Helsinki and the Department of Health and Human Services Belmont Report.	Yes	Material and Methods
Studies involving **human participants:** For publication of **patient photos**, include a statement confirming that consent to publish was obtained.	Not Applicable	
Studies involving experimental **animals**: State details of **authority granting ethics approval** (IRB or equivalent committee(s), provide reference number for approval. Include a statement of compliance with ethical regulations.	Not Applicable	
Studies involving **specimen and field samples:** State if relevant **permits** obtained, provide details of authority approving study; if none were required, explain why.	Not Applicable	

Dual Use Research of Concern (DURC)	Information included in the manuscript?	In which section is the information available? (Reagents and Tools Table, Materials and Methods, Figures, Data Availability Section)
Could your study fall under dual use research restrictions? Please check biosecurity documents and list of **select agents and toxins** (CDC): https://www.selectagents.gov/sat/list.htm	Not Applicable	
If you used a select agent, is the security level of the lab appropriate and reported in the manuscript?	Not Applicable	
If a study is subject to dual use research of concern regulations, is the name of the **authority granting approval and reference number** for the regulatory approval provided in the manuscript?	Not Applicable	

Reporting

The MDAR framework recommends adoption of discipline-specific guidelines, established and endorsed through community initiatives. Journals have their own policy about requiring specific guidelines and recommendations to complement MDAR.

Adherence to community standards	Information included in the manuscript?	In which section is the information available? (Reagents and Tools Table, Materials and Methods, Figures, Data Availability Section)
State if relevant guidelines or checklists (e.g., **ICMJE, MIBBI, ARRIVE, PRISMA**) have been followed or provided.	Not Applicable	
For **tumor marker prognostic studies,** we recommend that you follow the **REMARK** reporting guidelines (see link list at top right). See author guidelines, under 'Reporting Guidelines'. Please confirm you have followed these guidelines.	Not Applicable	
For **phase II and III randomized controlled trials**, please refer to the **CONSORT** flow diagram (see link list at top right) and submit the CONSORT checklist (see link list at top right) with your submission. See author guidelines, under 'Reporting Guidelines'. Please confirm you have submitted this list.	Not Applicable	

Data Availability

Data availability	Information included in the manuscript?	In which section is the information available? (Reagents and Tools Table, Materials and Methods, Figures, Data Availability Section)
Have **primary datasets** been deposited according to the journal's guidelines (see 'Data Deposition' section) and the respective accession numbers provided in the Data Availability Section?	Not Applicable	
Were **human clinical and genomic datasets** deposited in a public access-controlled repository in accordance to ethical obligations to the patients and to the applicable consent agreement?	Not Applicable	
Are **computational models** that are central and integral to a study available without restrictions in a machine-readable form? Were the relevant accession numbers or links provided?	Not Applicable	
If publicly available data were reused, provide the respective **data citations in the reference list**.	Yes	References
